# Coinage Metal-Catalyzed Divergent Heterocyclizations of Underexplored *N*‑Propargyl Hydrazides

**DOI:** 10.1021/acs.orglett.5c03069

**Published:** 2025-08-27

**Authors:** Daniel Diez-Iriepa, Mireia Toledano-Pinedo, Lorena Herrera-Hernández, Abdelouahid Samadi, Yasir S. Raouf, M. Mercedes Rodríguez-Fernández, Paula Flores-Galán, Hikaru Yanai, José M. Alonso, José Marco-Contelles, Pedro Almendros

**Affiliations:** ‡ Department of Chemistry, College of Science, 11239 United Arab Emirates University, Al Ain 15551, United Arab Emirates; § Instituto de Química Orgánica General (IQOG), 16379 Consejo Superior de Investigaciones Científicas (CSIC), Juan de la Cierva 3, 28006 Madrid, Spain; ∥ Departamento de Química Orgánica, 16722 Universidad Autónoma de Madrid, Cantoblanco, 28049 Madrid, Spain; ⊥ Centro de Investigaciones Biológicas Margarita Salas, Consejo Superior de Investigaciones Científicas (CSIC), Ramiro de Maeztu 9, 28040 Madrid, Spain; # School of Pharmacy, 13115 Tokyo University of Pharmacy and Life Sciences, 1432-1 Horinouchi, Hachioji, Tokyo 192-0392, Japan; ∇ Grupo de Lactamas y Heterociclos Bioactivos, Unidad Asociada al CSIC por el IQOG, Departamento de Química Orgánica, Facultad de Química, 16734 Universidad Complutense de Madrid, 28040 Madrid, Spain

## Abstract

Herein, we disclose metal-catalyzed tunable annulations of uncharted alkyne-tethered hydrazides for the divergent preparation of diazacyclic frameworks. Cationic gold facilitates the O-cyclization, providing valuable [1,3,4]­oxadiazine scaffolds with total selectivity, whereas silver catalysis promotes controlled N-cyclization to access *N*-acyl pyrazoles. Furthermore, density-functional-theory-based theoretical investigations support two different pathways (ionic versus radical) operating in the catalytic heterocyclization reaction of the same *N*-propargyl hydrazide substrate.

## Graphical abstract

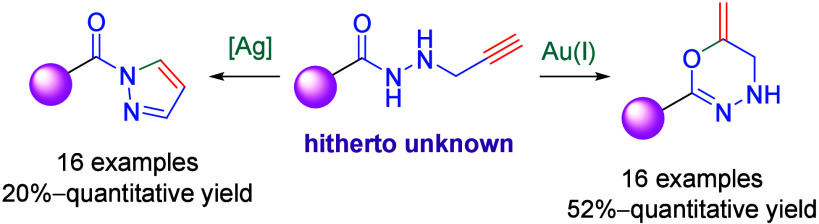

Azaheterocycles are ubiquitous compounds in drugs, agrochemicals, and advanced materials. Among heterocyclic cores, [1,3,4]­oxadiazines can be found in many bioactive molecules and *N*-acyl pyrazoles are important building blocks in organic synthesis (Scheme 1, top).^1,2^ Recent strategies for the preparation of the [1,3,4]­oxadiazine framework include the organocatalyzed cycloaddition of *N*-acyldiazenes with ketenes^3^ or allenoates.^4^ Despite hydrazides being of widespread use to prepare several heterocycles,^5^ propargyl hydrazides^6^ are hitherto unknown, and consequently, its reactivity remains uncharted. Gold and silver catalyses are often utilized for the cyclization of functionalized alkynes due to the π acidity of the metallic salts.^7,8^ Although the gold-catalyzed reaction of *N*-propargylcarboxamides to give 5-methyloxazoles is a well-known process (Scheme 1a and b),^9^ similar reactivity for silver or acyl derivatives of prop-2-yn-1-ylhydrazine has not yet been described.^10^ New synthetic protocols that provide rapid and selective access to either one heterocyclic motif or a skeleton different from the same starting material are highly desirable. Herein, we report a synthetic strategy based on noble metal-catalyzed cyclization of unexplored *N*-propargyl hydrazides, to enable divergent access to a series of [1,3,4]­oxadiazines and *N*-acyl pyrazoles depending on the nature of the metal (Scheme 1c). Relevant aspects of this approach embrace: (a) ambident nucleophilicity of novel alkyne-tethered hydrazides reacting as either *O*-nucleophile or *N*-nucleophile reagents, (b) divergent heterocyclization and convenient synthesis of both [1,3,4]­oxadiazines and *N*-acyl pyrazoles, (c) high atom economy (leaving or protective groups are not necessary), and (d) metal-controlled chemo- and regioselectivity without noticing competing cyclizations. First of all, we targeted a straightforward and cost-effective protocol for the preparation of alkyne-tethered hydrazides. It was found that the hexafluorophosphate azabenzotriazole tetramethyl uronium (HATU)-promoted coupling reaction of commercially available propargyl hydrazine hydrochloride with a variety of carboxylic acids resulted in the convenient preparation of *N*-propargyl hydrazides **1** (for details, see the Supporting Information).^11^ Our initial investigation focused on the evaluation of various reaction conditions by using *N*-propargyl hydrazide **1a** as a model substrate.

**Scheme 1:**
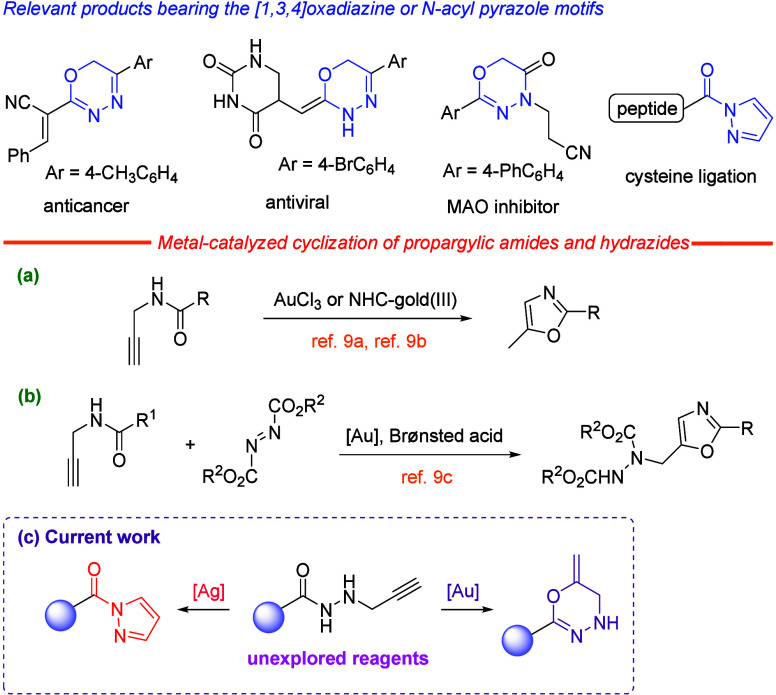
Background and Context of the Work

Different gold-based precatalysts, such as AuCl_3_, [AuClIPr] [IPr = 1,3-bis­(2,6-diisopropylphenyl)­imidazol-2-ylidene]/AgOTf, [(XPhos)­AuNTf_2_], and Au/TiO_2_ resulted in complex reaction mixtures (entries 1–3, Table S1; see the Supporting Information). Fortunately, the use of [(Ph_3_P)­AuCl]/AgOTf provided [1,3,4]­oxadiazine **2a** in a high 88% yield (entry 4, Table S1), while [(Ph_3_P)­AuNTf_2_] proved to be the optimal catalyst and procured oxadiazine **2a** in a great 96% isolated yield (entry 6, Table S1). The acidic media generated under the gold-catalyzed conditions partially decomposed oxadiazine **2a** in the absence of an inorganic base. The addition of stoichiometric amounts of K_2_CO_3_ granted reproducible results on the formation of heterocycle **2a** without impacting either chemo- or regioselectivity. Besides, no isomerization of the exocyclic double bond in **2a** toward endocyclic alkene was detected. Unreacted precursor **1a** was recovered to a considerable extent (80%) with the introduction of platinum or different Lewis acids as promoters. Screening of different solvents revealed that cyclic ethers are best suited for the directed reaction, while chlorinated solvents, alcohols, and aromatic hydrocarbons resulted in significant deterioration of the unsaturated hydrazide precursor **1a**. The heterocyclization reaction can also be conducted in ethyl acetate (entry 9, Table S1), although purification of oxadiazine **2a** is problematic because the reaction is more complicated. Noteworthy, silver-based complexes exert opposing activating effects and were able to tune the chemo- and regioselectivities of the process for the specific formation of *N*-acyl pyrazole **3a** (Table S2; see the Supporting Information).^12^ Nanometallic AgNO_3_·SiO_2_ demonstrated superior performance compared to different silver salts under homogeneous conditions and was selected as the catalyst^13^ of choice (entry 6, Table S2). With optimized reaction conditions in hand, the scope of the reactions was studied. As previously observed for model substrate **1a**, a totally distinct reaction outcome was noticed when gold- or silver-based complexes operated as precatalysts during the transformation of different alkyne-tethered hydrazides **1** (Schemes 2 and 3).

**Scheme 2:**
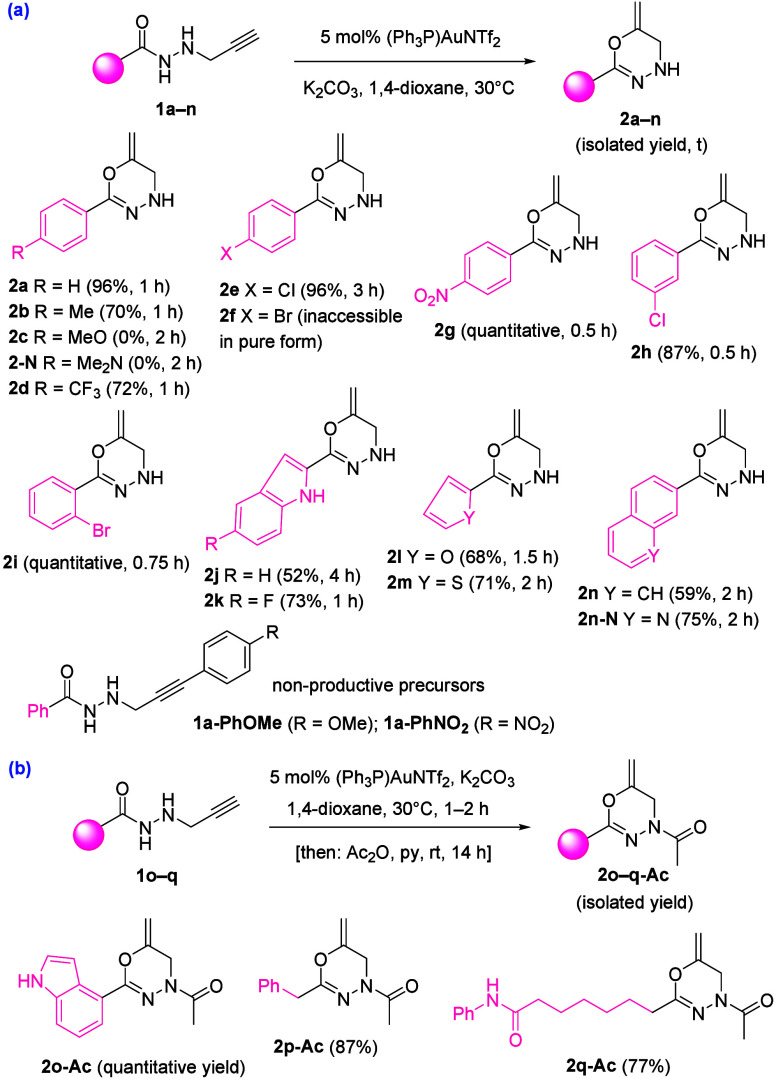
Gold-Catalyzed Controlled Preparation of Oxadiazines **2a**–**2n** and **2o-Ac**–**2q-Ac** ^a^

a Yield of pure, isolated product with correct analytical and spectral data.

**Scheme 3:**
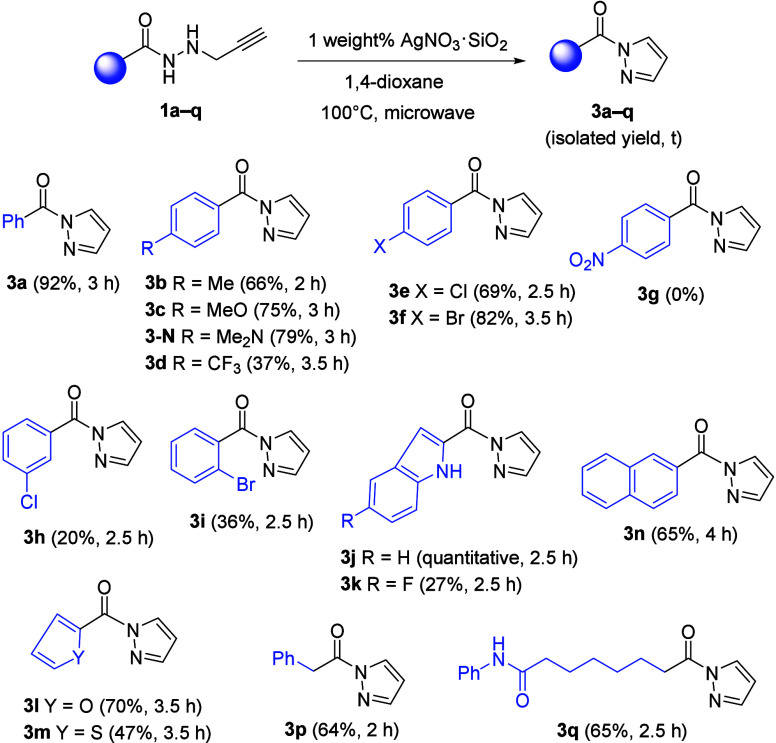
Silver-Catalyzed Synthesis of Pyrazoles **3a**–**3q** ^a^

a Yield of pure, isolated product with correct analytical and spectral data.

The Au­(I)-catalyzed cycloisomerization of *N*-propargyl hydrazides forged the [1,3,4]­oxadiazine skeleton through a 6-*exo*-*dig* oxycyclization, while its AgNP-catalyzed counterpart reaction formed the pyrazole scaffold via a 5-*endo*-*dig* azacyclization. Our method is not compatible with free OH, NH_2_, and COOH groups because the appropriate hydrazide precursors **1** are not achievable through the HATU-promoted coupling reaction. Both a mild electron-donating group (Me) and electron-withdrawing groups (Cl, CF_3_, and NO_2_) could be present in different positions (*ortho*, *meta*, and *para*) of the aryl ring, leading exclusively to [1,3,4]­oxadiazines **2** in good yields. However, electron-withdrawing groups (Cl, CF_3_, and NO_2_) gave better yields (72%, quantitative yield) than those with an electron-donating group (Me, 70%). Indeed, under otherwise identical conditions, the gold-catalyzed reaction of *N*-propargyl hydrazide **1c** bearing a more electron-rich 4-MeOC_6_H_4_ moiety did not occur to form the corresponding oxadiazine. The failure of **1c** may be ascribed to the resonance effect of the 4-methoxy substituent in the 4-methoxyphenyl group through the formation of quinone methide, which may be difficult for the nucleophilic attack of amide oxygen. Similarly, the cyclization of **1-N** bearing a 4-Me_2_NC_6_H_4_ group was unproductive under gold catalysis. Bromoderivative **2f** was generated, but we were unable to isolate it in its pure form. Per contra, when the protocol was applied to internal alkyne-tethered hydrazides **1a-PhOMe** and **1a-PhNO**_**2**_, oxadiazine formation was not attained (Scheme 2a). Interestingly, heterocycles **2o**–**2q**, which were elusive to chromatographic purification, were isolated as their corresponding acetylated derivatives **2o-Ac**, **2p-Ac**, and **2q-Ac** in a gold-catalyzed/acetylation one-pot process (Scheme 2b). By contrast, reverse electronic effects were observed for the conversion of alkynes **1** into *N*-acyl pyrazoles **3**, because the electron-donating group furnished five-membered azacycles **3** (e.g., **3c** and **3-N** versus **3d**) in increased yields (Scheme 3). To our delight, the indole nucleus was well-tolerated in *N*-propargyl hydrazides **1**, and both O-cyclization products **2j** and **2k** and N-cyclization products **3j** and **3k** were efficiently built in the presence of [(Ph_3_P)­AuNTf_2_] and AgNO_3_·SiO_2_, respectively. It should be noted that no protection for the NH-free indole was required for either the gold-catalyzed cycloisomerization reaction or the silver-catalyzed process. Besides, other heterocyclic rings, such as furan, thiophene, and quinoline, were also well-accommodated. When alkyl-substituted *N*-propargyl hydrazides **1p** and **1q** were used as precursors, both metal-catalyzed reactions delivered the products. However, while oxadiazines **2p** and **2q** decomposed during chromatographic purification, pyrazoles **3p** and **3q** were isolated in 64 and 65% yields, respectively. The bulky 2-naphthalene nucleus was tolerated, affording oxadiazine **2n** and pyrazole **3n** in 59 and 65% yields, respectively. The above results point to a notable dependence of the effectiveness of both sequences related to the electronic nature of the substituent attached to the carbonyl group, while little influence due to steric hindrance is observed (e.g., **2e** versus **2h** versus **2i**). Complete conversion was observed by TLC and ^1^H NMR analysis of the crude reaction mixtures of precursors **1**. The unwanted side products, the volatility of several pyrazoles **3**, and some decomposition perceived during purification by flash chromatography may be the cause of the moderate isolated yields.

Besides unprotected NH-free hydrazides **1**, di-*tert*-butyl 1-(prop-2-yn-1-yl)­hydrazine-1,2-dicarboxylate **1-Boc** was also compatible with the above gold catalysis regime. Indeed, the gold-catalyzed heterocyclization was also operative starting from alkyne-tethered bis­(carbamate) **1-Boc**, with 2-oxo-[1,3,4]­oxadiazine **4** being smoothly formed in a selective way (Scheme 4). The value of the gold-catalyzed reaction of **1-Boc** was not just that *N*-Boc-protected hydrazine can work as a NC­(O)O nucleophile but rather that the gold-catalyzed reaction of **1-Boc** proceeded chemoselectively through O-cyclization of the distal carbamate motif, allowing the transfer of the carbonyl group inside the six-membered ring **4**. To inspect the behavior of *N*-homopropargyl hydrazides, we tested *N*′-(but-3-yn-1-yl)­benzohydrazide (homologue of **1a**), but it was not very rewarding because it remained unreacted under gold catalysis and very little conversion was observed after the silver treatment.

**Scheme 4:**
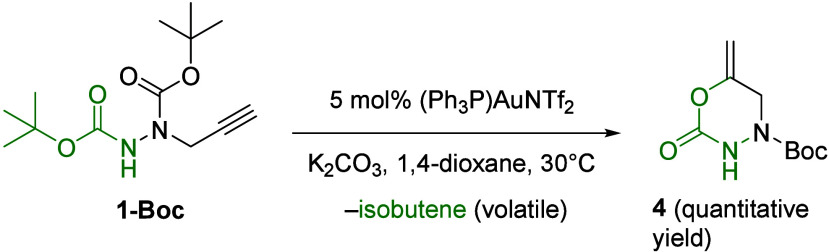
Gold-Catalyzed Cyclization of Hydrazide **1-Boc**

Post-synthetic transformations of heterocycles **2** under both oxidative conditions (epoxidation using MCPBA and dihydroxylation using OsO_4_) and palladium catalysis [4-iodotoluene, catalyzed by either Pd­(OAc)_2_ or Pd­(PPh_3_)_4_ in the presence of SPhos and K_2_CO_3_] resulted in decomposition. The acetylation and bromobenzoylation of [1,3,4]­oxadiazine **2g** were smoothly accomplished to provide acylated derivatives **2g-Ac** and **2g-BrBz** (Scheme 5a). Taking into account the utility of vicinal dihalides in pharmacology and material chemistry,^14^ we decided to explore the dibromination of selected oxadiazines **2-Ac**. The reaction of [1,3,4]­oxadiazine **2g** under several dihalogenation conditions resulted in decomposition. Interestingly, the selective formation of 1,2-dibrominated derivatives **4g** and **5g** was attained after exposure of **2g-Ac** and **2g-BrBz** to NBS in nitromethane^15^ (Scheme 5b). Adduct **5g** was formed along with its rotamer **5g-rot**, which were easily separable by flash column chromatography.^16^ When a scale-up reaction was run starting from 2 mmol of *N*-propargyl hydrazide **1a** (Scheme 5c), oxadiazine **2a** was obtained in a similar yield (92% versus 96%), which revealed the practicability of our method.

**Scheme 5:**
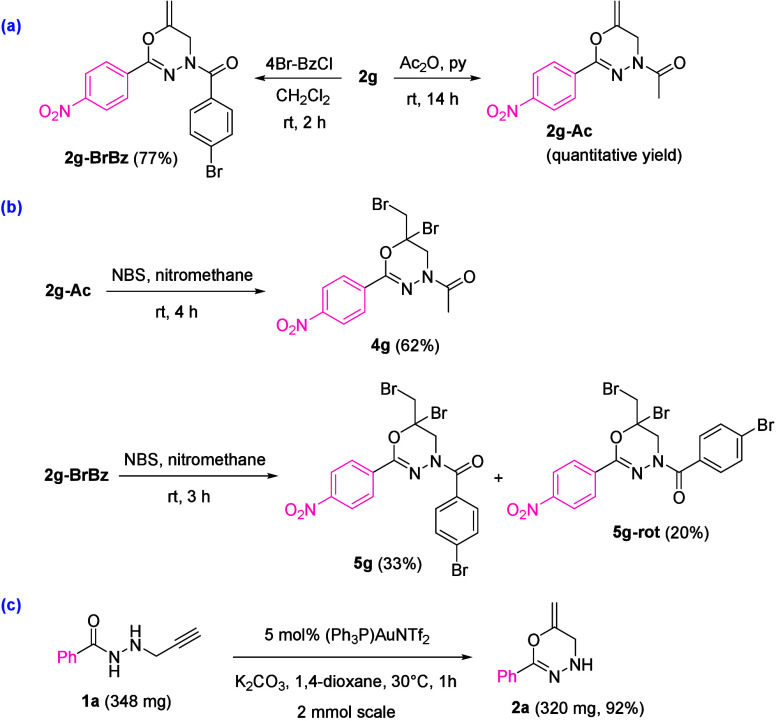
Transformations of Oxadiazines **2** and Scale-Up^a^

a Yield of pure, isolated product with correct analytical and spectral data.

In order to enlighten the mechanism of the considered reactions, we incorporated the established radical scavenger 2,2,6,6-tetramethyl-piperidinium-1-oxyl radical (TEMPO) into the standard reaction conditions of propargyl hydrazide **1a** under both gold and silver catalyses. The addition of TEMPO did not exert influence in the yield of [1,3,4]­oxadiazine **2a** but resulted in both a reduction of the yield of pyrazole **3a** (from 92 to 58%) and a considerable diminution in the reaction rate. In a similar way, a decrease in the yield (from 75 to 29%) was noticed during the silver-catalyzed formation of target product **3c** starting from cyclization precursor **2c** in the presence of TEMPO. Likewise, the use of butylated hydroxytoluene (BHT) as a radical-trapping reagent had a considerable effect on the silver-catalyzed reaction of **2c** and inhibited it in great extension. Besides, a TEMPO-trapped adduct from **2a** was identified by HPLC–MS, displaying a mass-to-charge ratio (*m*/*z*) of 330.19 (see the Supporting Information). The above results firmly suggest that the silver-catalyzed sequence progresses via a free radical pathway. A tentative mechanism for the formation of 6-methylene-2-aryl-5,6-dihydro-4*H*-1,3,4-oxadiazines **2** should start with the η coordination of cationic gold to the triple bond in *N*′-(prop-2-yn-1-yl)­arenehydrazides **1**, which should result in complexes **1-Au**. Next, chemoselective 6-*exo*-*dig* oxyauration by nucleophilic attack of the carbonyl moiety to form cationic 4*H*-1,3,4-oxadiazin-3-ium intermediate **INT-I** followed by deprotonation should construct neutral alkenylgold species **INT-II**. Finally, protonolysis of the carbon–gold bond should liberate heterocycles **2** with concomitant regeneration of the Au­(I) catalytic species (Scheme 6).

**Scheme 6:**
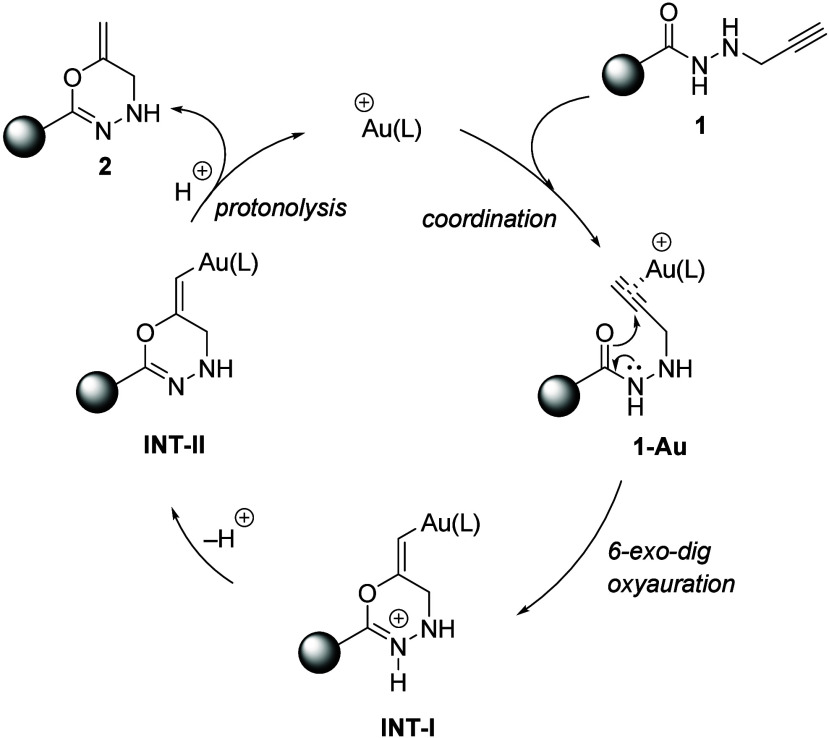
Tentative Mechanism for the Formation of Oxadiazines **2**

Based on previous literature,^17^ a plausible path for the formation of aryl­(1*H*-pyrazol-1-yl)­methanones **3** is depicted in Scheme 7. The reaction should be initiated by molecular-oxygen-promoted oxidation of silver(0) to silver­(I). Next, substitution of the proton in *N*-propargyl hydrazides **1** with silver­(I) should generate silver amide **INT-B**, which via homolytic cleavage of the N–[Ag­(I)] bond should form nitrogen-centered hydrazidyl radical **INT-C**, while oxygen-mediated oxidation of silver(0) to silver­(I) closes the catalytic cycle. An ensuing 5-*endo* azacyclization should occur, giving rise to 2,3-dihydro-1*H*-pyrazole radical **INT-D**. Then, two consecutive hydrogen atom transfers (HATs) assisted by the solvent should build radical **INT-F**, via neutral pyrazoline **INT-E**. Oxidation of species **INT-F** to form pyrazolium intermediate **INT-G** followed by proton release should liberate final products **3**.

**Scheme 7:**
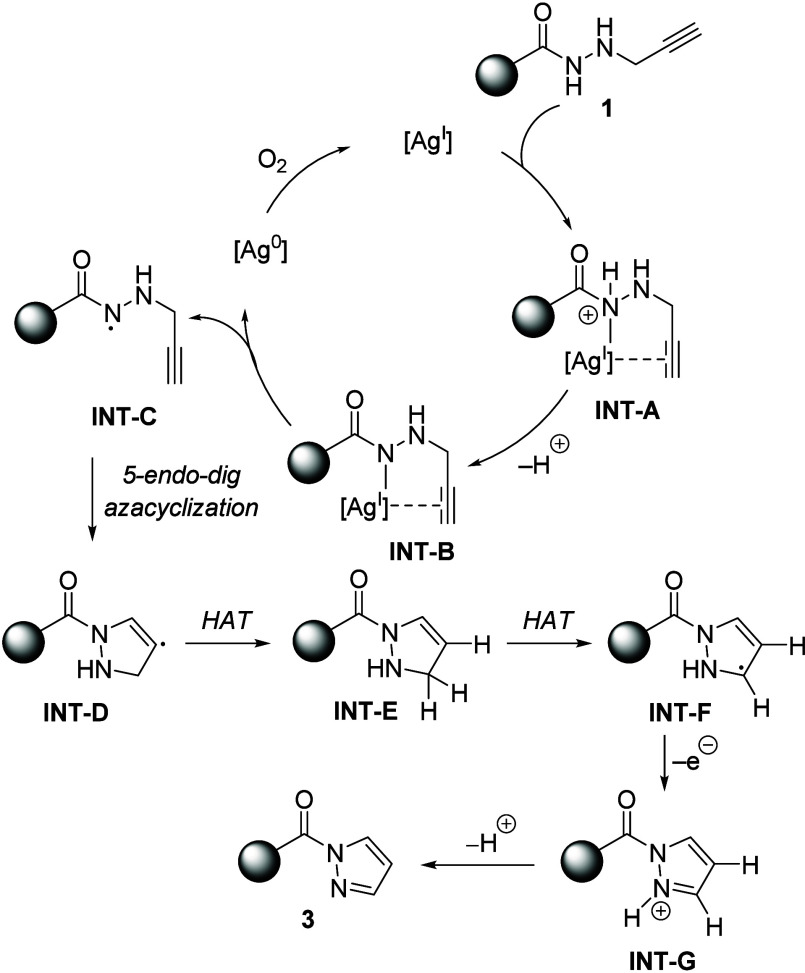
Plausible Pathway for the Formation of Pyrazoles **3**

To support the proposed mechanisms for both heterocyclizations, density functional theory (DFT) calculations were conducted (see the Supporting Information). Interestingly, in both Au- and Ag-mediated reactions, the 6-*exo*-*dig* ring closure yielding a [1,3,4]­oxadiazine product was found to be energetically more favorable than the 5-*endo*-*dig* mode. This implies that, regardless of the kind of noble metal center, the ionic cyclization triggered by π-Lewis acidic activation of the alkyne moiety predominantly gives [1,3,4]­oxadiazines. On the other hand, a selective formation of *N*-acylpyrazole product **3** was successfully simulated in the cyclization of amidyl radical **INT-C**. That is, the activation energy of radical 5-*endo*-*dig* cyclization (+30.6 kcal mol^–1^) was much lower than that of radical 6-*exo*-*dig* cyclization (+32.4 kcal mol^–1^). The following HAT process between **INT-D** and a 1,4-dioxane molecule, which was explicitly considered as a solvent molecule, has a very low barrier (+5.5 kcal mol^–1^), and the formation of **INT-E** and a dioxane radical is exergonic. These data support the irreversible formation of **INT-E**. Although there are several possibilities for the formation of **3** from **INT-E**, our computation proposes the generation of **INT-F** through the HAT reaction by the dioxane radical and the following oxidation to yield **INT-G**, which easily gives **3** through deprotonation.

To summarize, the coinage metal-catalyzed chemo- and regiodivergent cyclization of unexplored *N*-propargyl hydrazides having multiple reactive sites makes it possible to direct access to structurally diverse heterocycles. The controlled oxycyclization provides [1,3,4]­oxadiazines with the assistance of gold complexes, while silver-catalyzed fine-tuned reactivity delivers *N*-acyl pyrazoles using the same starting materials. These reactions present high atom and step economies with good functional group tolerance. DFT studies corroborated that two different pathways, namely, ionic (Au) versus radical (Ag), are operating.

## Supplementary Material



## Data Availability

The data underlying this study are available in the published article and its Supporting Information.

## Notes

Experimental procedures, characterization data of new compounds, copies of NMR spectra, and computational details (PDF)
