# Supplementary material for: Coinage Metal-Catalyzed Divergent Heterocyclizations of Underexplored N‑Propargyl Hydrazides
Source: Org Lett. 2025 Aug 27;27(35):9753–8. doi: 10.1021/acs.orglett.5c03069 (PMC12418500; doi:10.1021/acs.orglett.5c03069)

Supporting Information for the Paper

Coinage Metal-Catalyzed Divergent Heterocyclizations of Underexplored *N*-Propargyl Hydrazides

Daniel Diez-Iriepa,[†,a] Mireia Toledano-Pinedo,[‡,a] Lorena Herrera-Hernández,[‡] Abdelouahid Samadi,[†] Yasir S. Raouf, [†] M. Mercedes Rodríguez-Fernández,[#] Paula Flores-Galán,[‖] Hikaru Yanai,[⊥] José M. Alonso,[*,§] José Marco-Contelles,[*,‡] and Pedro Almendros[*,‡]

[†]Department of Chemistry, College of Science, United Arab Emirates University, Al Ain 15551, UAE

[‡]Instituto de Química Orgánica General, IQOG, CSIC, Juan de la Cierva 3, 28006 Madrid, Spain

[#]Departamento de Química Orgánica, Universidad Autónoma de Madrid, Cantoblanco, 28049 Madrid, Spain

[‖]Centro de Investigaciones Biológicas Margarita Salas, CSIC, Ramiro de Maeztu 9, 28040 Madrid, Spain

[⊥]School of Pharmacy, Tokyo University of Pharmacy and Life Sciences, 1432-1 Horinouchi, Hachioji, Tokyo 192-0392, Japan

[§]Grupo de Lactamas y Heterociclos Bioactivos, Unidad Asociada al CSIC por el IQOG, Departamento de Química Orgánica, Facultad de Química, Universidad Complutense de Madrid, 28040-Madrid, Spain

[a]These authors have equally contributed to this work

E-mails: josalo08@ucm.es; iqoc21@iqog.csic.es; palmendros@iqog.csic.es

Table of Contents

1.- General Methods S2

2.- Table S1 and Table S2 S2–S3

3.- DFT calculations S3–S19

4.- Experimental Section S19–S54

5.- NMR Spectra S55–S149

1.- General Methods: ^{1}H NMR and ^{13}C NMR spectra were recorded on a Bruker Avance-DPX 300, Bruker Avance III HD-400, JEOL JNM-ECZ 400R, or Bruker Avance AVIII-600. NMR spectra were recorded in $CDCl_3$, DMSO-d_6, or acetone-d_6 solutions, except otherwise stated. Chemical shifts are given in ppm relative to TMS (^{1}H, 0.0 ppm), or $CDCl_3$ (^{1}H, 7.27 ppm; ^{13}C, 76.9 ppm), or DMSO-d_6 (^{1}H, 2.50 ppm; ^{13}C, 39.5 ppm), or acetone-d_6 (^{1}H, 2.05 ppm; ^{13}C, 206.3 ppm). Low- and high-resolution mass spectra were taken on an AGILENT 6520 Accurate-Mass QTOF LC/MS spectrometer using the electrospray mode (ES) unless otherwise stated. IR spectra were recorded on a Bruker Tensor 27 spectrometer. Microwave irradiation was carried out in a Monowave 300 from Anton Paar GmbH. The reaction temperatures during microwave heating were measured with an internal infrared sensor. Syntheses in the microwave reactor were conducted in sealed reaction vessels. Column chromatography was carried out using silica gel 60, 0.04-0.06 mm, for flash chromatography (230-400 mesh ASTM) provided by Scharlau. For reactions that require heating, a heating-on block was used. All commercially available compounds were used without further purification. Structural assignments were made with additional information from gCOSY, gHSQC, and gHMBC experiments.

2.- Table S1 and Table S2

Table S1. Synthesis of Oxadiazine 2a under Modified Metal-Catalyzed Conditions

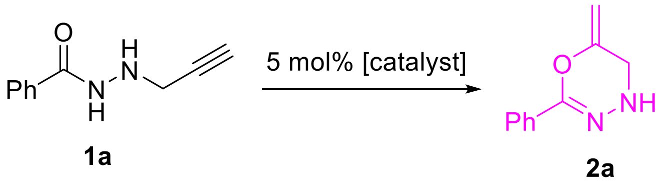

entry	[Catalyst]	Conditions[b]	yield **2a** (%)[c]
1	$AuCl_3$	K_2CO_3, 1,4-dioxane, rt, 1 h	Complex mixture
2	[AuClIPr]/AgOTf	K_2CO_3, 1,4-dioxane, rt, 1 h	Complex mixture
3	[(XPhos)AuNTf$_2$]	K_2CO_3, 1,4-dioxane, rt, 1 h	Complex mixture
4	[(Ph$_3$P)AuCl]/AgOTf	K_2CO_3, 1,4-dioxane, 30°C, 1 h	88
5	[(Ph$_3$P)AuNTf$_2$]	1,4-dioxane, rt, 1 h	Complex mixture
**6**	**[(Ph$_3$P)AuNTf$_2$]**	**K_2CO_3, 1,4-dioxane, 30°C, 1 h**	**96**

7	[$(Ph_3P)AuNTf_2$]	K_2CO_3, DCE, rt, 16 h	Complex mixture
8	[$(Ph_3P)AuNTf_2$]	K_2CO_3, ethanol, rt, 16 h	Complex mixture
9	[$(Ph_3P)AuNTf_2$]	K_2CO_3, ethyl acetate, rt, 1 h	38
10	Au/TiO_2[a]	1,4-dioxane, 100°C, 6 h	Complex mixture
11	$PtCl_2$	K_2CO_3, 1,4-dioxane, rt, 16 h	Recovered **1a**

[a]10 weight% Au/TiO_2 (1 weight% Au on anatase) was used. [b]DCE = 1,2-dichloroethane. [c]Yield of pure, isolated product with correct analytical and spectral data.

Table S2. Synthesis of Pyrazole 3a under Modified Metal-Catalyzed Conditions

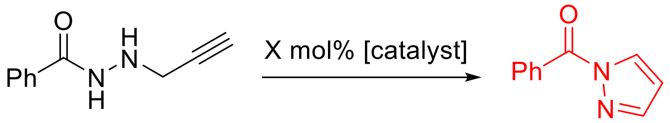

1a **3a**

entry	[Catalyst]	Conditions	yield **3a** (%)[c]
1	$Sc(OTf)_3$	K_2CO_3, 1,4-dioxane, rt, 16 h	Recovered **1a**
2	$AgNO_3$[a]	K_2CO_3, 1,4-dioxane, 100°C, 3 h	87
3	$AgNO_3$[a]	1,4-dioxane, 100°C, 3 h	89
4	$AgNO_3$[a]	ethyl acetate, 100°C, 3 h	85
5	AgOTf	K_2CO_3, 1,4-dioxane, 100°C, 3 h	76
**6**	**$AgNO_3 \cdot SiO_2$[b]**	**1,4-dioxane, 100°C, 3 h**	**92**

[a]15 mol% was used. [b]1 weight% $AgNO_3 \cdot SiO_2$ (1 weight% $AgNO_3$ on silica) was used. [c]Yield of pure, isolated product with correct analytical and spectral data. Silver-catalyzed reactions were carried out in a microwave reactor.

3.- DFT Calculations

All calculations were conducted by using a *Gaussian 09* program package, revision D.01.[1] Molecular geometries were optimized at PCM(1,4-dioxane)-B3LYP-D3/def2-SVP level of theory. Thermochemical properties were obtained by single point calculations with frequency analysis using def2-TZVPP basis instead of def2-SVP. Free energies ΔG are calculated as the values at 403 K. Single imaginary frequency was obtained in all transition states, which were supported by the intrinsic reaction coordinate (IRC) calculations using the local quadratic approximation (lqa) algorithm. Each geometry of reaction intermediates was obtained by structural optimization of the IRC geometries.

Au(I)-catalyzed reaction paths

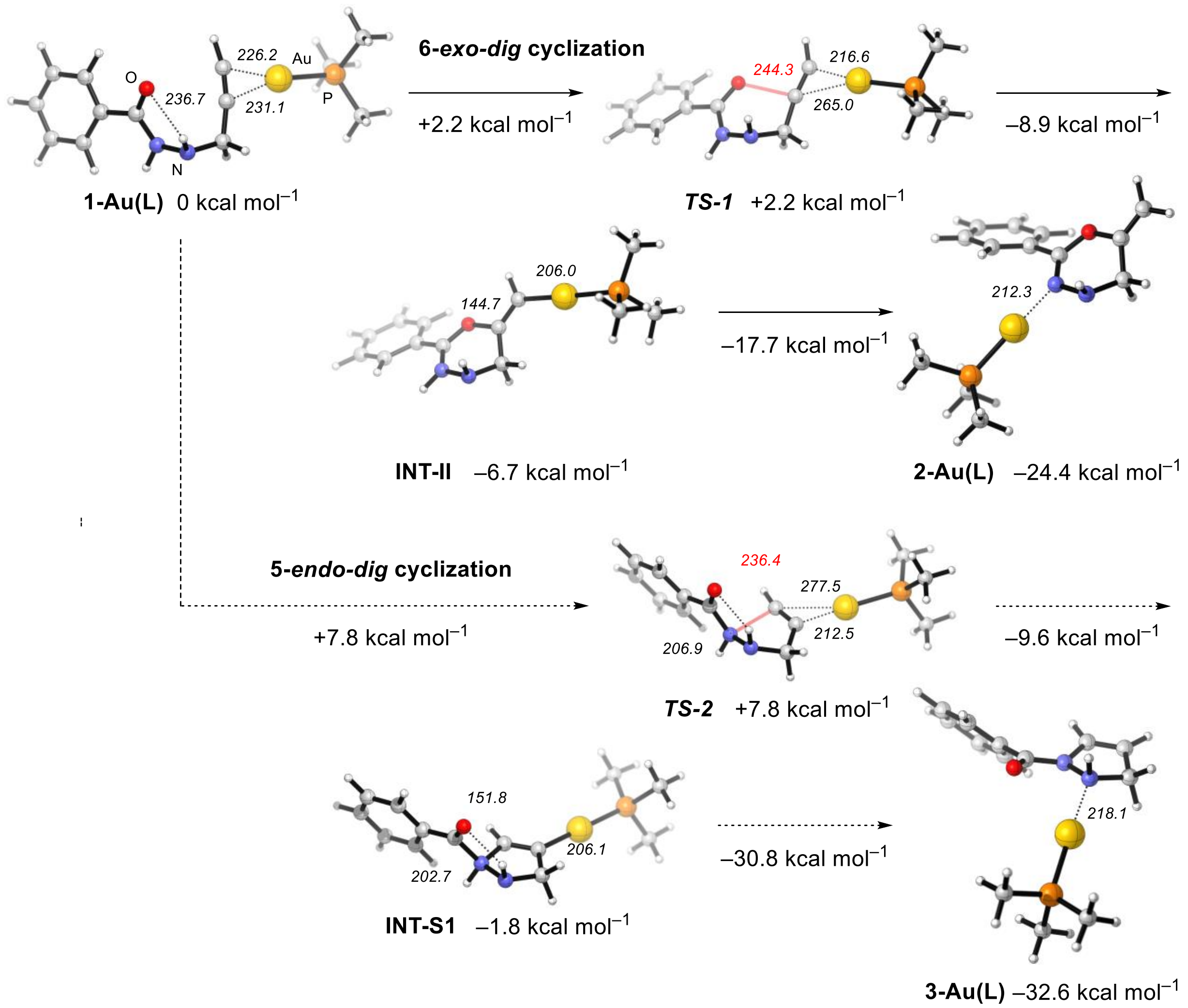

Figure S1. Reaction profiles for Au(I)-catalyzed reaction paths

Table S1. Molecular geometries and energies

**1-Au(L)** (L = PMe_3)					***TS-1***				
Center Number	Atomic Number	Coordinates (Angstroms) X	Y	Z	Center Number	Atomic Number	Coordinates (Angstroms) X	Y	Z
1	6	-0.859305	1.030902	0.023334	1	6	1.856614	1.804785	-3.185214
2	6	-0.218391	0.704790	1.025324	2	6	1.986878	0.989033	-2.252245
3	6	0.270351	0.350495	2.381961	3	6	2.402917	0.053898	-1.195501
4	7	-0.768710	0.363838	3.402289	4	7	1.962938	0.396646	0.154293
5	7	-1.770924	-0.585977	3.217103	5	7	0.677999	-0.009382	0.477848
6	6	-2.966114	-0.227304	2.638615	6	6	-0.408353	0.336468	-0.276561
7	8	-3.090235	0.860130	2.089260	7	8	-0.266715	0.988540	-1.309969
8	6	-4.068679	-1.235664	2.700729	8	6	-1.738375	-0.135615	0.203062
9	6	-4.103231	-2.298777	3.618127	9	6	-1.994882	-0.435210	1.551310
10	6	-5.172827	-3.195644	3.615270	10	6	-3.264134	-0.863839	1.942534
11	6	-6.216167	-3.039310	2.697984	11	6	-4.282469	-0.994146	0.993281
12	6	-6.193648	-1.976287	1.788968	12	6	-4.034849	-0.684300	-0.348496
13	6	-5.129479	-1.076051	1.795208	13	6	-2.770096	-0.249899	-0.741813
14	1	-1.610180	1.340114	-0.688473	14	1	1.143855	2.418443	-3.726568
15	1	1.052026	1.065731	2.681803	15	1	3.505876	0.014340	-1.209010
16	1	0.737473	-0.645466	2.352262	16	1	2.048932	-0.960960	-1.433128
17	1	-1.207323	1.285261	3.458797	17	1	2.075204	1.394151	0.338011
18	1	-1.712888	-1.392583	3.828364	18	1	0.606913	-0.699834	1.217033
19	1	-3.316472	-2.427717	4.364908	19	1	-1.219513	-0.304136	2.309302
20	1	-5.195795	-4.013934	4.338034	20	1	-3.461566	-1.087434	2.992987
21	1	-7.051945	-3.742663	2.697653	21	1	-5.274800	-1.330737	1.301850
22	1	-7.012031	-1.847422	1.077253	22	1	-4.832697	-0.779119	-1.088180
23	1	-5.099237	-0.232962	1.103579	23	1	-2.560287	0.005832	-1.781502
24	79	1.228037	0.687697	-0.777110	24	79	3.917269	1.849331	-3.850256
25	15	3.135023	0.492219	-2.096781	25	15	6.026382	2.111891	-4.786090

26	6	4.662550	0.393833	-1.102050
27	1	5.538305	0.332713	-1.765991
28	1	4.746552	1.287773	-0.468009
29	1	4.627244	-0.498215	-0.460735
30	6	3.127760	-0.991190	-3.158376
31	1	2.251353	-0.965042	-3.821282
32	1	4.045377	-1.022558	-3.765277
33	1	3.071782	-1.891163	-2.529817
34	6	3.352864	1.914996	-3.218392
35	1	3.438024	2.838781	-2.628649
36	1	4.262619	1.778847	-3.822819
37	1	2.481026	1.995283	-3.883171

E(RB3LYP) = -1168.89233253
Zero-point correction = 0.297918 (Hartree/Particle)
Sum of electronic and thermal Energies = -1168.574090
Sum of electronic and thermal Enthalpies = -1168.573145
Sum of electronic and thermal Free Energies = -1168.644479

26	6	7.327312	2.470592	-3.555684
27	1	8.295176	2.610002	-4.060913
28	1	7.066870	3.385326	-3.004385
29	1	7.402399	1.635701	-2.844398
30	6	6.597888	0.642429	-5.706240
31	1	5.891154	0.424534	-6.519653
32	1	7.598351	0.824988	-6.127549
33	1	6.637471	-0.222768	-5.029596
34	6	6.077148	3.493615	-5.978670
35	1	5.809771	4.429443	-5.467611
36	1	7.086480	3.585153	-6.407866
37	1	5.352463	3.308859	-6.784307

E(RB3LYP) = -1168.88776054
Zero-point correction = 0.297575 (Hartree/Particle)
Sum of electronic and thermal Energies = -1168.569805
Sum of electronic and thermal Enthalpies = -1168.568861
Sum of electronic and thermal Free Energies = -1168.640897

INT-II

Center Number	Atomic Number	Coordinates (Angstroms) X	Y	Z
1	6	-0.618970	0.212414	-0.209503
2	6	-0.875057	-0.553204	0.848985
3	6	0.014455	-1.377055	1.726399
4	7	-0.266769	-1.132416	3.146162
5	7	-1.638414	-1.252937	3.396046
6	6	-2.571000	-1.072571	2.471518
7	8	-2.251160	-0.670651	1.279795
8	6	-3.987932	-1.322336	2.737579
9	6	-4.484755	-1.406361	4.052987
10	6	-5.838280	-1.648749	4.268233
11	6	-6.704088	-1.805918	3.179523
12	6	-6.216662	-1.714090	1.871785
13	6	-4.864359	-1.469903	1.646008
14	1	-1.501985	0.694288	-0.650303
15	1	1.065921	-1.136634	1.531753
16	1	-0.130655	-2.455075	1.538760
17	1	0.029544	-0.194375	3.424547
18	1	-1.889970	-1.691132	4.278619
19	1	-3.833041	-1.253822	4.915763
20	1	-6.222652	-1.705488	5.288117
21	1	-7.765402	-1.995655	3.353093
22	1	-6.893871	-1.834481	1.024292
23	1	-4.475268	-1.399503	0.630194
24	79	1.227283	0.518308	-1.069835
25	15	3.306208	0.898135	-2.105912
26	6	4.669877	1.295719	-0.948464
27	1	5.609063	1.462655	-1.497895
28	1	4.413088	2.201927	-0.380887
29	1	4.804087	0.464604	-0.240985
30	6	3.927015	-0.526103	-3.075519
31	1	3.199196	-0.773944	-3.861674
32	1	4.897937	-0.288916	-3.536680
33	1	4.038993	-1.397964	-2.415124
34	6	3.296741	2.290029	-3.295687
35	1	3.015987	3.215633	-2.772780
36	1	4.289346	2.414379	-3.754904
37	1	2.552938	2.091756	-4.080738

E(RB3LYP) = -1168.90827814
Zero-point correction = 0.301189 (Hartree/Particle)
Sum of electronic and thermal Energies = -1168.588144
Sum of electronic and thermal Enthalpies = -1168.587199
Sum of electronic and thermal Free Energies = -1168.655105

2-Au(L)

Center Number	Atomic Number	Coordinates (Angstroms) X	Y	Z
1	6	-0.616878	0.299752	0.324220
2	6	-0.912556	-0.611014	1.247232
3	6	-0.010207	-1.532101	2.004766
4	7	-0.326660	-1.506681	3.428339
5	7	-1.703898	-1.631405	3.703712
6	6	-2.575424	-1.312873	2.788332
7	8	-2.251276	-0.789926	1.605690
8	6	-4.028172	-1.495579	2.973344
9	6	-4.643806	-1.178942	4.196420
10	6	-6.017776	-1.359095	4.350745
11	6	-6.783966	-1.853983	3.289910
12	6	-6.176898	-2.150685	2.065885
13	6	-4.804395	-1.962702	1.900751
14	1	-1.396132	0.910323	-0.135084
15	1	1.043925	-1.262309	1.868436
16	1	-0.145213	-2.567470	1.645369
17	1	0.032248	-0.682549	3.912046
18	1	0.418252	0.434460	0.008979
19	1	-4.052863	-0.762194	5.013453
20	1	-6.495646	-1.097347	5.297113
21	1	-7.859743	-1.995780	3.413967
22	1	-6.775465	-2.526988	1.233953
23	1	-4.328432	-2.186026	0.944755
24	79	-2.111404	-2.774950	5.445334
25	15	-2.529713	-4.057635	7.293140
26	6	-3.486976	-3.166435	8.567818
27	1	-3.674279	-3.821802	9.432113
28	1	-2.924860	-2.280437	8.896166
29	1	-4.445638	-2.839774	8.139951
30	6	-3.507275	-5.547342	6.894521
31	1	-2.947335	-6.170937	6.183235
32	1	-3.712011	-6.127060	7.807522
33	1	-4.456582	-5.245548	6.429575
34	6	-1.024803	-4.661704	8.130829
35	1	-0.409568	-3.806868	8.445201
36	1	-1.297580	-5.261855	9.012115
37	1	-0.442790	-5.278677	7.431538

E(RB3LYP) = -1168.93619788
Zero-point correction = 0.300414 (Hartree/Particle)
Sum of electronic and thermal Energies = -1168.616823
Sum of electronic and thermal Enthalpies = -1168.615879
Sum of electronic and thermal Free Energies = -1168.683408

TS-2

Center	Atomic	Coordinates (Angstroms)

INT-S1

Center	Atomic	Coordinates (Angstroms)

Number	Number	X	Y	Z	Number	Number	X	Y	Z
1	6	-0.747187	-0.752780	1.023572	1	6	-1.020673	-0.398506	1.102791
2	6	0.156026	0.051536	1.380155	2	6	0.076762	0.354063	1.022507
3	6	0.252726	0.787385	2.697613	3	6	0.184452	1.171629	2.285707
4	7	-1.080440	0.903268	3.287031	4	7	-1.070772	0.952786	3.051573
5	7	-1.896151	-0.179292	3.007844	5	7	-1.766768	-0.125502	2.395766
6	6	-3.175794	0.107956	2.488558	6	6	-3.245217	0.235221	2.119896
7	8	-3.429654	1.240029	2.125002	7	8	-3.503741	1.401699	2.093670
8	6	-4.122200	-1.034307	2.409831	8	6	-4.140635	-0.901298	1.908715
9	6	-3.730821	-2.373609	2.586566	9	6	-3.729099	-2.247552	1.988487
10	6	-4.673891	-3.396408	2.487667	10	6	-4.653270	-3.268331	1.781555
11	6	-6.010691	-3.091806	2.211036	11	6	-5.987059	-2.958025	1.494246
12	6	-6.404002	-1.762075	2.026274	12	6	-6.402645	-1.623470	1.412182
13	6	-5.464228	-0.737397	2.120693	13	6	-5.486945	-0.597548	1.617813
14	1	-1.375707	-1.455765	0.497768	14	1	-1.468979	-1.143083	0.449338
15	1	0.678752	1.794646	2.602729	15	1	0.316457	2.246581	2.089787
16	1	0.925504	0.204549	3.348327	16	1	1.054378	0.838789	2.876996
17	1	-1.577164	1.718595	2.913671	17	1	-1.694911	1.756766	2.936496
18	1	-1.822133	-0.946250	3.674040	18	1	-1.742695	-0.947249	3.013366
19	1	-2.689356	-2.637860	2.783076	19	1	-2.695672	-2.525091	2.202894
20	1	-4.364510	-4.434702	2.622875	20	1	-4.332931	-4.309597	1.844236
21	1	-6.747334	-3.894753	2.136368	21	1	-6.707620	-3.762756	1.333479
22	1	-7.447273	-1.525036	1.808164	22	1	-7.444116	-1.386368	1.187780
23	1	-5.750148	0.305569	1.977648	23	1	-5.792578	0.447903	1.560028
24	79	1.567378	0.159976	-0.204459	24	79	1.444838	0.403235	-0.517903
25	15	3.182003	0.405978	-1.866263	25	15	3.005404	0.500797	-2.274734
26	6	4.816647	-0.253121	-1.389157	26	6	4.653287	-0.168382	-1.842055
27	1	5.534801	-0.115375	-2.211908	27	1	5.341552	-0.086132	-2.697053
28	1	5.177674	0.275656	-0.495634	28	1	5.062285	0.390815	-0.988363
29	1	4.727152	-1.323689	-1.156480	29	1	4.552775	-1.224695	-1.553661
30	6	2.746038	-0.443978	-3.424035	30	6	2.507136	-0.427432	-3.771966
31	1	1.801637	-0.036556	-3.812397	31	1	1.550945	-0.033053	-4.145122
32	1	3.542914	-0.298883	-4.169408	32	1	3.273683	-0.332904	-4.556291
33	1	2.617043	-1.518634	-3.231376	33	1	2.374077	-1.488746	-3.517185
34	6	3.470628	2.151476	-2.320451	34	6	3.331389	2.200009	-2.872279
35	1	3.809664	2.707966	-1.435098	35	1	3.721365	2.810676	-2.045253
36	1	4.234909	2.218978	-3.109636	36	1	4.062325	2.186603	-3.695149
37	1	2.531976	2.596187	-2.680317	37	1	2.391494	2.647909	-3.225809

E(RB3LYP) = -1168.87892788	E(RB3LYP) = -1168.90044518
Zero-point correction = 0.297185 (Hartree/Particle)	Zero-point correction = 0.301428 (Hartree/Particle)
Sum of electronic and thermal Energies = -1168.562036	Sum of electronic and thermal Energies = -1168.580004
Sum of electronic and thermal Enthalpies = -1168.561092	Sum of electronic and thermal Enthalpies = -1168.579059
Sum of electronic and thermal Free Energies = -1168.632020	Sum of electronic and thermal Free Energies = -1168.647293

3-Au(L)

Center	Atomic	Coordinates (Angstroms)		
Number	Number	X	Y	Z
1	6	-1.543473	-0.526433	0.837426
2	6	-0.230075	-0.284375	0.792006
3	6	0.311527	-0.274302	2.197515
4	7	-0.932037	-0.201937	3.040110
5	7	-1.995875	-0.636070	2.171284
6	6	-3.277795	-0.605044	2.700243
7	8	-3.447419	-0.186783	3.837740
8	6	-4.377717	-1.111427	1.837068
9	6	-4.214385	-2.198227	0.961802
10	6	-5.299452	-2.660992	0.215990
11	6	-6.546926	-2.040520	0.335075
12	6	-6.716078	-0.966878	1.216264
13	6	-5.639365	-0.510317	1.974617
14	1	-2.259670	-0.634605	0.027241
15	1	0.957484	0.580204	2.443759
16	1	0.863361	-1.198597	2.435195
17	1	-1.129657	0.784227	3.253253
18	1	0.371234	-0.164486	-0.106391
19	1	-3.250323	-2.704190	0.883983
20	1	-5.172902	-3.513398	-0.454679
21	1	-7.392799	-2.400382	-0.254825

22	1	-7.692333	-0.487658	1.314447
23	1	-5.759365	0.316079	2.677070
24	79	-0.898612	-1.221541	4.967717
25	15	-0.786404	-2.295831	6.981561
26	6	-2.220632	-1.940421	8.051695
27	1	-2.121912	-2.488595	9.001373
28	1	-2.271902	-0.860985	8.252407
29	1	-3.143774	-2.248409	7.540385
30	6	-0.735706	-4.112731	6.815390
31	1	0.144383	-4.404811	6.225610
32	1	-0.683542	-4.579500	7.810640
33	1	-1.642214	-4.456812	6.297381
34	6	0.687911	-1.841509	7.956833
35	1	0.694169	-0.755715	8.128305
36	1	0.673565	-2.365122	8.924747
37	1	1.595532	-2.119321	7.402177

E(RB3LYP) = -1168.94990173
Zero-point correction = 0.301432 (Hartree/Particle)
Sum of electronic and zero-point Energies = -1168.648470
Sum of electronic and thermal Energies = -1168.629563
Sum of electronic and thermal Enthalpies = -1168.628619
Sum of electronic and thermal Free Energies = -1168.696391

Ag(I)-catalyzed reaction paths

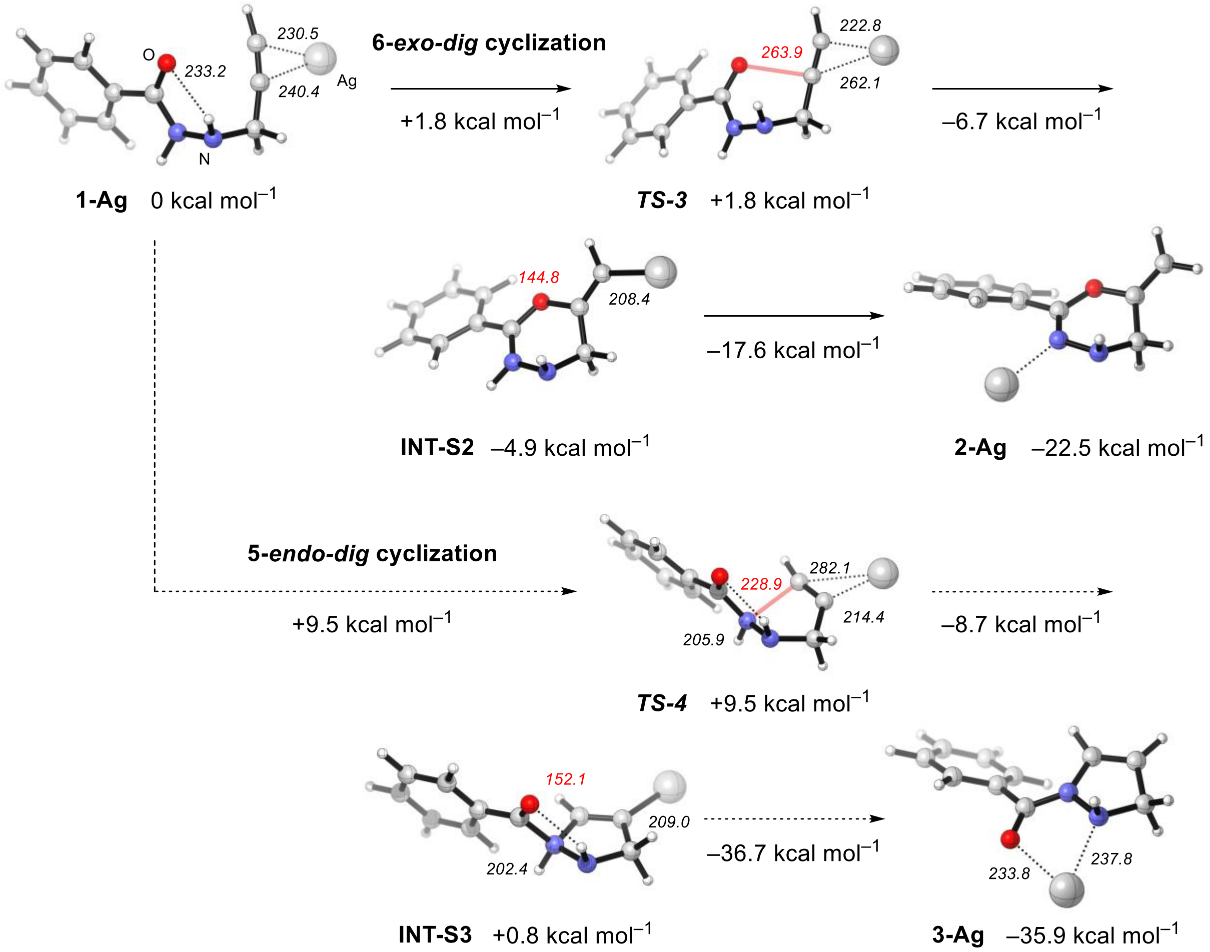

Figure S2. Reaction profile for Ag(I)-catalyzed reaction paths

Table S2. Molecular geometries and energies

**1-Ag**					***TS-3***				
Center Number	Atomic Number	Coordinates (Angstroms) X	Y	Z	Center Number	Atomic Number	Coordinates (Angstroms) X	Y	Z
1	6	0.921192	0.841933	-1.222593	1	6	1.830491	1.666534	-3.229239

2	6	1.304884	0.829643	-0.052370
3	6	1.474522	0.891607	1.421715
4	7	0.223683	1.122299	2.128743
5	7	-0.704707	0.090444	2.001893
6	6	-1.718831	0.206057	1.068411
7	8	-1.722097	1.128945	0.265302
8	6	-2.779309	-0.842703	1.117370
9	6	-2.592040	-2.088217	1.739748
10	6	-3.621419	-3.030629	1.743610
11	6	-4.841584	-2.737029	1.126682
12	6	-5.029110	-1.502241	0.496074
13	6	-4.000700	-0.561087	0.485727
14	1	0.375230	0.922238	-2.151599
15	1	2.166662	1.710757	1.672846
16	1	1.927784	-0.043032	1.784374
17	1	-0.213003	1.993565	1.818212
18	1	-0.893535	-0.407359	2.866956
19	1	-1.634843	-2.341463	2.200543
20	1	-3.469290	-3.999644	2.223660
21	1	-5.646976	-3.474918	1.133333
22	1	-5.980869	-1.274383	0.011342
23	1	-4.124611	0.405571	-0.004803
24	47	3.122617	0.217879	-1.502077

E(RB3LYP) = -718.859054966
Zero-point correction = 0.182256 (Hartree/Particle)
Sum of electronic and thermal Energies = -718.663498
Sum of electronic and thermal Enthalpies = -718.662554
Sum of electronic and thermal Free Energies = -718.718762

2	6	2.089512	0.948003	-2.256942
3	6	2.501915	0.139954	-1.092993
4	7	1.915314	0.541332	0.182268
5	7	0.653942	0.042847	0.450412
6	6	-0.453858	0.424824	-0.261371
7	8	-0.339173	1.150133	-1.243825
8	6	-1.767147	-0.104803	0.210173
9	6	-1.981751	-0.562349	1.520920
10	6	-3.237986	-1.037270	1.900522
11	6	-4.287433	-1.056612	0.976929
12	6	-4.082529	-0.590621	-0.326234
13	6	-2.830108	-0.111328	-0.706622
14	1	1.171136	2.265531	-3.845595
15	1	3.600010	0.199878	-1.006198
16	1	2.254679	-0.917812	-1.271059
17	1	1.934950	1.554069	0.304038
18	1	0.598256	-0.633994	1.202554
19	1	-1.184727	-0.522603	2.266514
20	1	-3.400473	-1.383839	2.923187
21	1	-5.269839	-1.428807	1.276189
22	1	-4.904225	-0.598715	-1.045580
23	1	-2.652955	0.265042	-1.715132
24	47	3.865593	1.466312	-4.112723

E(RB3LYP) = -718.854974129
Zero-point correction = 0.181649 (Hartree/Particle)
Sum of electronic and thermal Energies = -718.659788
Sum of electronic and thermal Enthalpies = -718.658844
Sum of electronic and thermal Free Energies = -718.715856

INT-S2

Center	Atomic	Coordinates (Angstroms)		
Number	Number	X	Y	Z
1	6	0.532846	0.807292	-1.283219
2	6	0.368311	0.066762	-0.193492
3	6	1.334675	-0.684612	0.668696
4	7	1.113587	-0.391330	2.088258
5	7	-0.235475	-0.562529	2.415036
6	6	-1.220794	-0.462397	1.534832
7	8	-0.979169	-0.099077	0.310711
8	6	-2.608847	-0.760105	1.884679
9	6	-3.031096	-0.805706	3.227829
10	6	-4.358527	-1.096081	3.527467
11	6	-5.272338	-1.339154	2.495078
12	6	-4.859755	-1.284808	1.159687
13	6	-3.534003	-0.992838	0.849414
14	1	-0.377937	1.240831	-1.712502
15	1	2.364710	-0.412452	0.412434
16	1	1.223609	-1.773823	0.529299
17	1	1.382485	0.568529	2.315361
18	1	-0.420930	-0.968470	3.329348
19	1	-2.340966	-0.586119	4.045019
20	1	-4.685681	-1.123419	4.568221
21	1	-6.313178	-1.566890	2.734476
22	1	-5.575130	-1.472030	0.356969
23	1	-3.203703	-0.950974	-0.188558
24	47	2.347079	1.165319	-2.244251

E(RB3LYP) = -718.872288232
Zero-point correction = 0.185665 (Hartree/Particle)
Sum of electronic and thermal Energies = -718.674660
Sum of electronic and thermal Enthalpies = -718.673716
Sum of electronic and thermal Free Energies = -718.726593

2-Ag

Center	Atomic	Coordinates (Angstroms)		
Number	Number	X	Y	Z
1	6	0.474021	1.025802	-1.185938
2	6	0.302455	0.171164	-0.181477
3	6	1.317129	-0.620563	0.582447
4	7	1.097081	-0.495436	2.021130
5	7	-0.244242	-0.630416	2.409269
6	6	-1.192109	-0.475699	1.532880
7	8	-0.995427	-0.078999	0.273929
8	6	-2.607123	-0.733445	1.869857
9	6	-3.113018	-0.388979	3.137202
10	6	-4.439745	-0.673431	3.461173
11	6	-5.270963	-1.293698	2.521379
12	6	-4.778551	-1.609832	1.252116
13	6	-3.452467	-1.324829	0.920098
14	1	-0.375157	1.540221	-1.639121
15	1	2.338179	-0.296763	0.348724
16	1	1.230630	-1.688996	0.316222
17	1	1.476275	0.363437	2.421222
18	1	1.475644	1.210353	-1.575731
19	1	-2.485077	0.159057	3.845724
20	1	-4.833639	-0.389692	4.439162
21	1	-6.309688	-1.515372	2.774888
22	1	-5.430527	-2.081190	0.514100
23	1	-3.065512	-1.568895	-0.070179
24	47	-0.630235	-1.659584	4.310591

E(RB3LYP) = -718.899460799
Zero-point correction = 0.184703 (Hartree/Particle)
Sum of electronic and thermal Energies = -718.702712
Sum of electronic and thermal Enthalpies = -718.701768
Sum of electronic and thermal Free Energies = -718.754542

TS-4

Center	Atomic	Coordinates (Angstroms)		
Number	Number	X	Y	Z
1	6	-0.775952	-0.741096	1.048028
2	6	0.147402	0.059540	1.364593

INT-S3

Center	Atomic	Coordinates (Angstroms)		
Number	Number	X	Y	Z
1	6	0.323544	-0.316467	0.168554
2	6	1.419093	0.417524	-0.005895

3	6	0.258807	0.792147	2.684661
4	7	-1.071859	0.880558	3.288412
5	7	-1.876880	-0.202039	2.981466
6	6	-3.178172	0.098053	2.502403
7	8	-3.433326	1.238751	2.173429
8	6	-4.125149	-1.039924	2.418034
9	6	-3.742708	-2.379791	2.612070
10	6	-4.689286	-3.398557	2.509673
11	6	-6.020702	-3.089641	2.212129
12	6	-6.405464	-1.759736	2.010736
13	6	-5.462581	-0.738800	2.109517
14	1	-1.403233	-1.450333	0.528055
15	1	0.665909	1.807905	2.596025
16	1	0.947370	0.215295	3.323972
17	1	-1.578862	1.701072	2.940642
18	1	-1.791950	-0.983343	3.630655
19	1	-2.706696	-2.649734	2.827894
20	1	-4.387457	-4.436893	2.660004
21	1	-6.760171	-3.889697	2.135066
22	1	-7.444587	-1.519838	1.776908
23	1	-5.742516	0.304170	1.955409
24	47	1.533558	0.208247	-0.264799

E(RB3LYP) = -718.845592430
Zero-point correction = 0.182567 (Hartree/Particle)
Sum of electronic and thermal Energies = -718.650738
Sum of electronic and thermal Enthalpies = -718.649794
Sum of electronic and thermal Free Energies = -718.703668

3	6	1.581306	1.338083	1.174032
4	7	0.361570	1.177605	2.011437
5	7	-0.381262	0.074687	1.458677
6	6	-1.859892	0.458837	1.189300
7	8	-2.085289	1.628490	1.097928
8	6	-2.786416	-0.663552	1.060486
9	6	-2.411619	-2.011741	1.234612
10	6	-3.362699	-3.019186	1.097813
11	6	-4.687661	-2.693447	0.786994
12	6	-5.067469	-1.356976	0.613280
13	6	-4.124730	-0.344062	0.749204
14	1	-0.151849	-1.110284	-0.402875
15	1	1.696877	2.393426	0.882961
16	1	2.477964	1.058936	1.752968
17	1	-0.257899	1.984309	1.890858
18	1	-0.363386	-0.699715	2.135315
19	1	-1.386691	-2.300063	1.474286
20	1	-3.071100	-4.061775	1.234575
21	1	-5.429557	-3.487709	0.680421
22	1	-6.102400	-1.108222	0.372046
23	1	-4.402718	0.702605	0.619173
24	47	2.738343	0.290694	-1.621523

E(RB3LYP) = -718.863609793
Zero-point correction = 0.186129 (Hartree/Particle)
Sum of electronic and thermal Energies = -718.665525
Sum of electronic and thermal Enthalpies = -718.664580
Sum of electronic and thermal Free Energies = -718.717551

3-Ag

Center Number	Atomic Number	Coordinates (Angstroms) X	Y	Z
1	6	-0.073456	-0.216157	0.012333
2	6	1.242976	0.010910	0.001075
3	6	1.596935	0.878283	1.185471
4	7	0.269140	1.390689	1.638476
5	7	-0.682982	0.470562	1.094068
6	6	-1.912690	0.342502	1.674198
7	8	-2.263229	1.107590	2.588157
8	6	-2.821562	-0.721856	1.174776
9	6	-2.382566	-2.038008	0.951378
10	6	-3.294876	-3.018297	0.558811
11	6	-4.641930	-2.690345	0.378812
12	6	-5.083923	-1.383793	0.613133
13	6	-4.181629	-0.404321	1.024577
14	1	-0.688966	-0.808954	-0.658209
15	1	2.248683	1.730941	0.950883
16	1	2.076869	0.294440	1.989703
17	1	0.098648	2.282483	1.160716
18	1	1.957459	-0.391556	-0.714121
19	1	-1.338197	-2.310150	1.115108
20	1	-2.953391	-4.043159	0.400194
21	1	-5.351892	-3.458293	0.064265
22	1	-6.137647	-1.130135	0.481215
23	1	-4.519817	0.612944	1.228045
24	47	-0.460005	1.908697	3.841989

E(RB3LYP) = -718.922072064
Zero-point correction = 0.185772 (Hartree/Particle)
Sum of electronic and thermal Energies = -718.724400
Sum of electronic and thermal Enthalpies = -718.723456
Sum of electronic and thermal Free Energies = -718.775941

Radical-mediated reaction paths

Radical 6-*exo-dig* cyclization (disfavored)

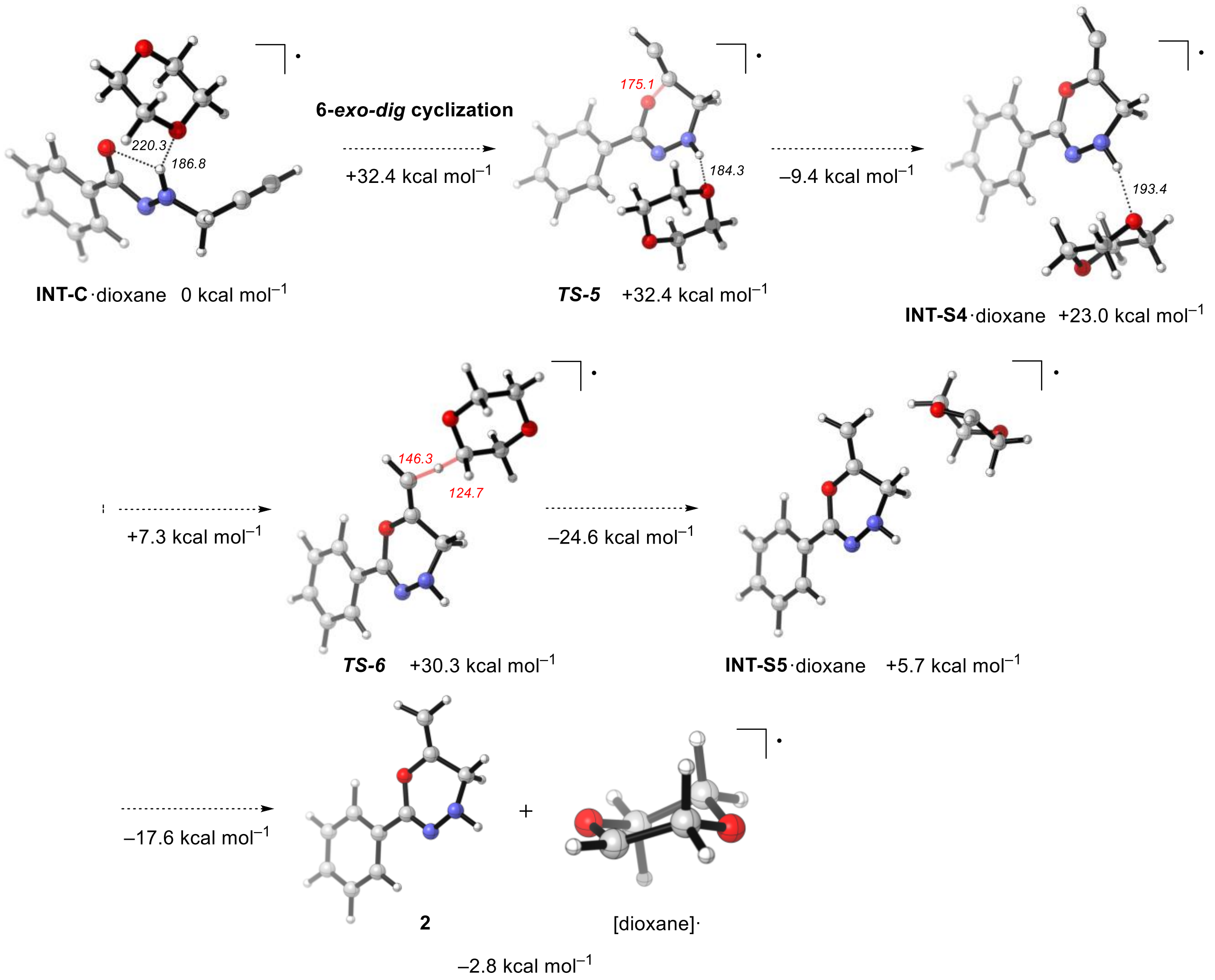

Figure S3. Reaction profile of radical 6-*endo*-*dig* cyclization

Table S3. Molecular geometries and energies

**INT-C**·dioxane					***TS-5***				
Center Number	Atomic Number	Coordinates (Angstroms) X	Y	Z	Center Number	Atomic Number	Coordinates (Angstroms) X	Y	Z
1	6	-3.317548	2.910341	0.768486	1	6	0.230963	-3.252353	0.798726
2	6	-1.643408	1.433803	2.250649	2	6	-1.686391	-1.489555	0.774688
3	7	-0.765323	0.613932	1.439196	3	7	-1.465806	-0.155966	1.321519
4	7	0.365139	1.105368	1.014121	4	7	-0.386902	0.582166	1.121413
5	6	1.006403	0.276783	0.107147	5	6	0.537177	0.146220	0.282279
6	8	0.525521	-0.793495	-0.283296	6	8	0.411677	-0.960351	-0.395157
7	6	2.324191	0.781362	-0.368348	7	6	1.786196	0.928468	0.154877
8	6	2.858527	2.005135	0.070539	8	6	1.966442	2.142620	0.844476
9	6	4.097543	2.437934	-0.403183	9	6	3.152213	2.861113	0.701707
10	6	4.813464	1.656283	-1.316456	10	6	4.175061	2.380609	-0.125018
11	6	4.285869	0.436898	-1.757014	11	6	4.002957	1.173619	-0.809532
12	6	3.047979	0.001806	-1.285859	12	6	2.816146	0.451980	-0.673512
13	1	-3.984959	3.502814	0.170041	13	1	1.132947	-3.828084	0.645012
14	1	-2.226233	0.762726	2.901231	14	1	-2.313339	-2.050335	1.482961
15	1	-1.019431	2.078384	2.889065	15	1	-2.231443	-1.426924	-0.185971
16	1	-1.147554	-0.269422	1.050797	16	1	-2.032897	0.173781	2.114138
17	1	2.290678	2.605194	0.781475	17	1	1.172672	2.509891	1.495212
18	1	4.508326	3.390287	-0.059351	18	1	3.282239	3.801674	1.242468
19	1	5.783724	1.997668	-1.685708	19	1	5.103630	2.946166	-0.233772
20	1	4.843658	-0.174456	-2.470462	20	1	4.797949	0.792769	-1.455319
21	1	2.616804	-0.944760	-1.615635	21	1	2.668646	-0.489521	-1.204035
22	6	-2.560382	2.251518	1.446013	22	6	-0.399785	-2.189330	0.551239
23	1	-1.721696	-2.993759	1.793822	23	1	-3.618897	2.455171	2.391097
24	6	-2.377984	-2.815085	0.929081	24	6	-2.942395	2.484186	3.258174
25	6	-1.732737	-3.303587	-0.360642	25	6	-1.682358	3.279439	2.946761

26	1	-3.338785	-3.339860	1.094837
27	6	-3.389888	-1.047217	-0.270321
28	1	-0.726875	-2.857668	-0.457547
29	1	-1.644496	-4.400820	-0.350637
30	6	-2.722342	-1.549954	-1.541734
31	1	-3.473140	0.049652	-0.263534
32	1	-4.403239	-1.483311	-0.178346
33	1	-3.355501	-1.340029	-2.417486
34	1	-1.748874	-1.039507	-1.670035
35	8	-2.616683	-1.410079	0.872944
36	8	-2.535808	-2.953746	-1.477766

E(UB3LYP) = -879.180602343
Zero-point correction = 0.291561 (Hartree/Particle)
Sum of electronic and thermal Energies = -878.871209
Sum of electronic and thermal Enthalpies = -878.870264
Sum of electronic and thermal Free Energies = -878.936664

26	1	-3.477812	2.938636	4.113422
27	6	-1.690754	1.067982	4.665583
28	1	-1.214933	2.877424	2.029437
29	1	-1.928598	4.340439	2.788619
30	6	-0.437010	1.873001	4.352137
31	1	-1.450952	0.005842	4.822908
32	1	-2.178811	1.462091	5.577236
33	1	0.227706	1.901283	5.228823
34	1	0.100204	1.400825	3.509799
35	8	-2.613583	1.132130	3.577767
36	8	-0.769691	3.215418	4.031868

E(UB3LYP) = -879.128912641
Zero-point correction = 0.290014 (Hartree/Particle)
Sum of electronic and thermal Energies = -878.822017
Sum of electronic and thermal Enthalpies = -878.821073
Sum of electronic and thermal Free Energies = -878.885063

INT-S4·dioxane

Center Number	Atomic Number	Coordinates (Angstroms) X	Y	Z
1	6	-0.409701	-4.196964	-1.669585
2	6	-1.684895	-2.294935	-0.617435
3	7	-1.171859	-1.525034	0.498575
4	7	-0.169627	-0.653871	0.205736
5	6	0.611801	-0.907534	-0.778249
6	8	0.522397	-2.039292	-1.562732
7	6	1.728687	-0.018698	-1.137161
8	6	1.896523	1.210155	-0.469573
9	6	2.951519	2.054362	-0.807473
10	6	3.853471	1.693087	-1.816449
11	6	3.689130	0.477692	-2.484970
12	6	2.634479	-0.375003	-2.150257
13	1	0.275878	-4.870831	-2.176509
14	1	-2.384062	-3.061479	-0.259658
15	1	-2.213658	-1.654332	-1.357002
16	1	-1.867091	-1.069157	1.096676
17	1	1.187998	1.494182	0.309051
18	1	3.070260	3.004879	-0.281787
19	1	4.678909	2.358789	-2.079558
20	1	4.387594	0.187814	-3.273751
21	1	2.507659	-1.324803	-2.669718
22	6	-0.502571	-2.933665	-1.304144
23	1	-3.684517	1.087372	0.590445
24	6	-3.120440	1.421439	1.474458
25	6	-1.889564	2.219661	1.067303
26	1	-3.784966	2.044982	2.103727
27	6	-1.960355	0.587697	3.341926
28	1	-1.293810	1.632257	0.345309
29	1	-2.188073	3.168246	0.595226
30	6	-0.729137	1.381742	2.929981
31	1	-1.676393	-0.358691	3.825597
32	1	-2.576183	1.178671	4.047381
33	1	-0.175301	1.718810	3.819428
34	1	-0.072180	0.742196	2.314239
35	8	-2.746303	0.253643	2.199637
36	8	-1.102136	2.544827	2.202543

E(UB3LYP) = -879.147174208
Zero-point correction = 0.292971 (Hartree/Particle)
Sum of electronic and thermal Energies = -878.837417
Sum of electronic and thermal Enthalpies = -878.836473
Sum of electronic and thermal Free Energies = -878.900060

TS-8

Center Number	Atomic Number	Coordinates (Angstroms) X	Y	Z
1	6	-1.072901	1.017536	1.732717
2	6	-0.024913	0.304252	2.546106
3	7	-0.646286	-0.091346	3.801119
4	7	-1.797021	-0.821646	3.684961
5	6	-2.549179	-0.595133	2.671981
6	8	-2.278430	0.341824	1.699119
7	6	-3.830465	-1.294355	2.473496
8	6	-4.267062	-2.244344	3.415318
9	6	-5.475261	-2.912287	3.231756
10	6	-6.266670	-2.646755	2.106992
11	6	-5.838086	-1.705080	1.168524
12	6	-4.628176	-1.030266	1.347767
13	1	0.826773	0.965407	2.748358
14	1	0.339339	-0.573776	1.969612
15	1	-0.014049	-0.542808	4.454855
16	1	-3.642366	-2.443492	4.286783
17	1	-5.804522	-3.646786	3.970802
18	1	-7.213624	-3.173034	1.965251
19	1	-6.449821	-1.491338	0.288702
20	1	-4.295186	-0.292906	0.617527
21	6	-0.917702	2.150495	1.060134
22	1	-1.636902	2.689285	0.442342
23	1	0.413892	2.755484	1.110911
24	6	1.535585	3.296150	1.041795
25	6	2.450847	2.378008	0.258447
26	1	1.868691	3.445554	2.088399
27	1	1.951654	2.084041	-0.686848
28	1	2.685900	1.467441	0.829468
29	6	2.551372	5.167916	0.083300
30	6	3.464641	4.247583	-0.715638
31	1	2.302845	6.075410	-0.487045
32	1	3.055987	5.464704	1.023868
33	1	4.448351	4.715461	-0.873431
34	1	3.006814	4.044916	-1.705173
35	8	1.324672	4.507237	0.379537
36	8	3.680443	3.033103	-0.018664

E(UB3LYP) = -879.129699150
Zero-point correction = 0.287134 (Hartree/Particle)
Sum of electronic and thermal Energies = -878.826161
Sum of electronic and thermal Enthalpies = -878.825217
Sum of electronic and thermal Free Energies = -878.888300

INT-S5·dioxane

Center Number	Atomic Number	Coordinates (Angstroms) X	Y	Z
1	6	-0.329620	1.216661	0.430698

2	6	0.928625	0.529180	0.879066
3	7	0.562977	-0.388642	1.948805
4	7	-0.428701	-1.279642	1.640493
5	6	-1.337902	-0.896815	0.821507
6	8	-1.387186	0.356118	0.253129
7	6	-2.471044	-1.759219	0.445855
8	6	-2.571814	-3.058988	0.975550
9	6	-3.637946	-3.882018	0.621355
10	6	-4.619350	-3.425231	-0.267587
11	6	-4.524218	-2.136235	-0.797284
12	6	-3.458321	-1.305059	-0.444454
13	1	1.659935	1.263460	1.234729
14	1	1.372536	-0.011008	0.014138
15	1	1.350075	-0.878858	2.362408
16	1	-1.800677	-3.404413	1.665040
17	1	-3.705828	-4.889094	1.040001
18	1	-5.454141	-4.073505	-0.544445
19	1	-5.286180	-1.771616	-1.490470
20	1	-3.386058	-0.297899	-0.854651
21	6	-0.478015	2.517627	0.169554
22	1	-1.440286	2.913209	-0.160413
23	1	0.375297	3.185765	0.280396
24	6	4.014717	2.781999	0.027048
25	6	4.794039	1.642161	-0.552004
26	1	4.134430	3.053682	1.079816
27	1	4.485709	0.679256	-0.072235
28	1	5.868606	1.771696	-0.351406
29	6	2.561348	2.771958	-1.828848
30	6	3.249659	1.497349	-2.286452
31	1	1.476812	2.727491	-1.998826
32	1	2.983801	3.637663	-2.368324
33	1	3.171274	1.396164	-3.379195
34	1	2.758924	0.616930	-1.821974
35	8	2.741502	2.976610	-0.422875
36	8	4.622085	1.529864	-1.953939

E(UB3LYP) = -879.172786273

Zero-point correction = 0.292371 (Hartree/Particle)

Sum of electronic and thermal Energies = -878.863357

Sum of electronic and thermal Enthalpies = -878.862413

Sum of electronic and thermal Free Energies = -878.927573

2

Center	Atomic	Coordinates (Angstroms)		
Number	Number	X	Y	Z
1	6	-0.330456	1.216363	0.433977
2	6	0.929983	0.528945	0.876597
3	7	0.568174	-0.393290	1.945077
4	7	-0.423909	-1.283532	1.634456
5	6	-1.335154	-0.899152	0.818891
6	8	-1.385493	0.355252	0.252657
7	6	-2.469717	-1.760159	0.444438
8	6	-2.574602	-3.057326	0.979630
9	6	-3.642192	-3.879141	0.627058
10	6	-4.620741	-3.423640	-0.265665
11	6	-4.521374	-2.137237	-0.800848
12	6	-3.454098	-1.307184	-0.449599
13	1	1.660077	1.261354	1.244280
14	1	1.373487	-0.004576	0.007744
15	1	1.357590	-0.886210	2.351265
16	1	-1.805666	-3.401717	1.672088
17	1	-3.713496	-4.884162	1.050033
18	1	-5.456653	-4.070992	-0.541290
19	1	-5.280867	-1.773848	-1.497355
20	1	-3.378349	-0.302216	-0.864494
21	6	-0.482453	2.516767	0.174769
22	1	-1.442539	2.911639	-0.161790
23	1	0.360606	3.197840	0.291503

E(UB3LYP) = -572.023023305

[dioxane]·

Center	Atomic	Coordinates (Angstroms)		
Number	Number	X	Y	Z
1	6	-3.783129	-4.238592	0.175785
2	6	-4.369652	-4.684042	-1.128410
3	1	-3.127549	-3.363216	0.209174
4	6	-5.317607	-5.564547	1.360874
5	1	-5.062115	-3.901974	-1.529441
6	1	-3.575578	-4.826106	-1.877674
7	6	-6.021798	-5.832495	0.040616
8	1	-6.037427	-5.437136	2.182280
9	1	-4.645375	-6.409048	1.597052
10	1	-6.554482	-6.794643	0.081153
11	1	-6.764175	-5.031266	-0.157393
12	8	-4.547679	-4.362519	1.297081
13	8	-5.080272	-5.905619	-1.009987

E(UB3LYP) = -307.144517496

Zero-point correction = 0.183359 (Hartree/Particle)<br>Sum of electronic and thermal Energies = -571.829672<br>Sum of electronic and thermal Enthalpies = -571.828728<br>Sum of electronic and thermal Free Energies = -571.875316	Zero-point correction = 0.107732 (Hartree/Particle)<br>Sum of electronic and thermal Energies = -307.031629<br>Sum of electronic and thermal Enthalpies = -307.030685<br>Sum of electronic and thermal Free Energies = -307.065751

Radical 5-*endo-dig* cyclization (favored)

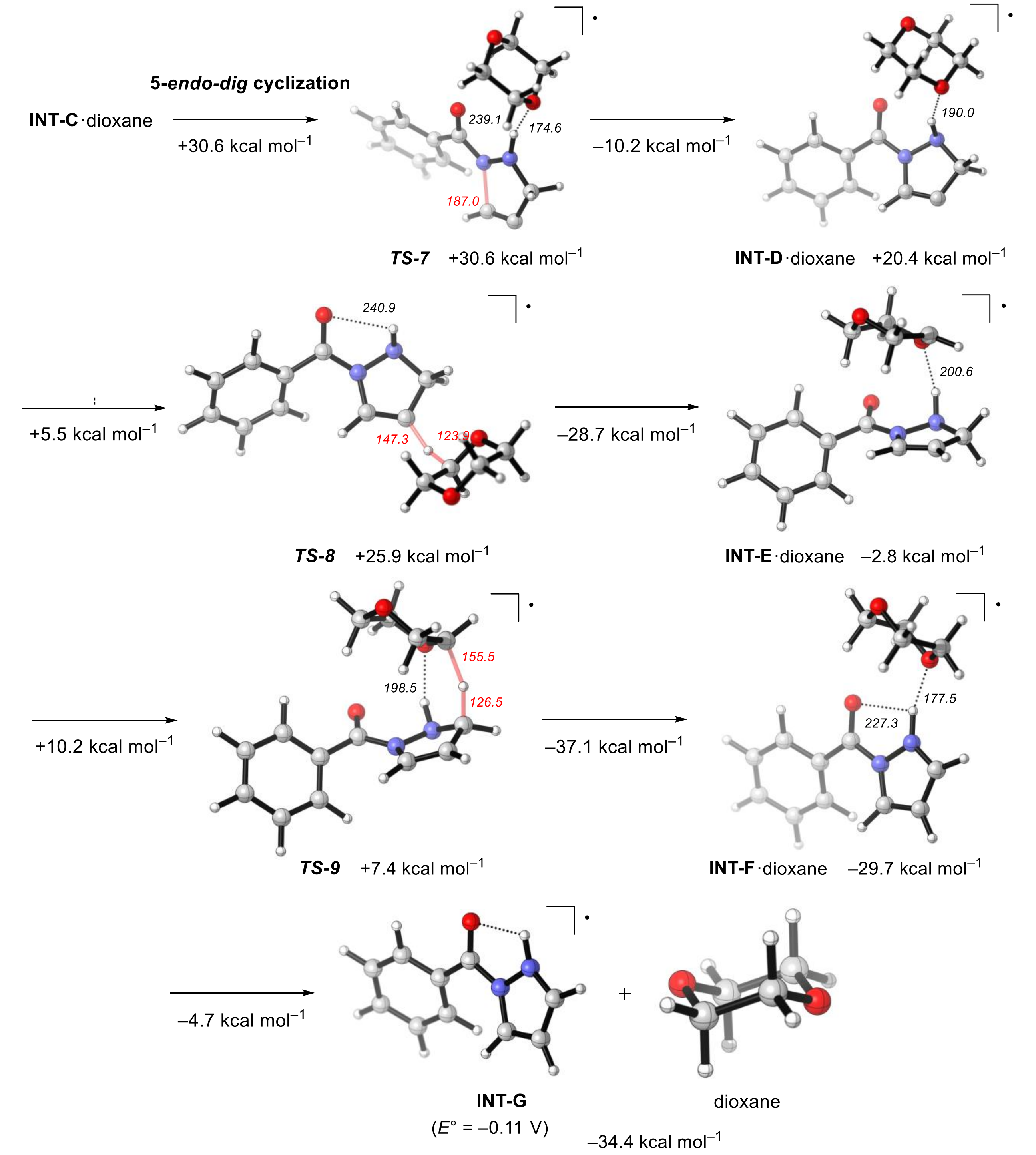

Figure S4. Reaction profile of radical 5-*endo-dig* cyclization

Table S4. Molecular geometries and energies

TS-7

Center Number	Atomic Number	Coordinates (Angstroms) X	Y	Z
1	6	0.793320	2.021847	2.582709
2	6	-1.375031	1.244663	2.743389
3	7	-0.912682	0.652101	1.460874
4	7	0.296882	0.978535	1.112333
5	6	0.896482	0.281485	0.070282
6	8	0.391241	-0.736383	-0.401877
7	6	2.187913	0.839791	-0.411923
8	6	2.531325	2.193430	-0.258270
9	6	3.742605	2.670574	-0.762135

INT-D·dioxane

Center Number	Atomic Number	Coordinates (Angstroms) X	Y	Z
1	6	0.518016	1.605394	1.909592
2	6	-1.825550	1.659904	2.042878
3	7	-1.417059	1.031297	0.765980
4	7	0.001685	1.177312	0.662896
5	6	0.627115	0.646285	-0.431301
6	8	0.010438	-0.056818	-1.229024
7	6	2.084917	0.942455	-0.596316
8	6	2.642462	2.197169	-0.303357
9	6	3.997967	2.436353	-0.540710

10	6	4.620336	1.802385	-1.418555
11	6	4.277616	0.455386	-1.585025
12	6	3.064135	-0.021745	-1.093176
13	1	1.818798	2.325256	2.421527
14	1	-2.276686	1.851510	2.538940
15	1	-1.705557	0.413576	3.393765
16	1	-1.470522	-0.051883	0.933295
17	1	1.837625	2.878140	0.230431
18	1	3.999536	3.725976	-0.646899
19	1	5.570136	2.176967	-1.807559
20	1	4.959724	-0.222639	-2.103057
21	1	2.773410	-1.065318	-1.224075
22	6	-0.260059	2.025128	3.315395
23	1	-2.256523	-2.430237	1.906462
24	6	-2.714677	-2.445202	0.906118
25	6	-1.891602	-3.274708	-0.068478
26	1	-3.737909	-2.857943	0.988018
27	6	-3.294619	-0.985735	-0.867907
28	1	-0.847942	-2.911443	-0.064115
29	1	-1.907272	-4.335426	0.224909
30	6	-2.468642	-1.840209	-1.819286
31	1	-3.249846	0.078448	-1.143454
32	1	-4.351077	-1.314158	-0.881879
33	1	-2.920234	-1.835438	-2.823070
34	1	-1.442644	-1.434532	-1.881021
35	8	-2.794421	-1.088980	0.465829
36	8	-2.440163	-3.186327	-1.373165

E(UB3LYP) = -879.131896163
Zero-point correction = 0.290431 (Hartree/Particle)
Sum of electronic and thermal Energies = -878.824614
Sum of electronic and thermal Enthalpies = -878.823670
Sum of electronic and thermal Free Energies = -878.887842

10	6	4.806275	1.426112	-1.069875
11	6	4.251781	0.179169	-1.379243
12	6	2.896032	-0.057725	-1.154661
13	1	1.591582	1.646103	2.074851
14	1	-2.365549	2.609506	1.869786
15	1	-2.504216	0.983101	2.588191
16	1	-1.636943	0.025715	0.768686
17	1	2.011231	2.998860	0.084117
18	1	4.421892	3.418649	-0.319880
19	1	5.867229	1.613583	-1.251466
20	1	4.878891	-0.608633	-1.803004
21	1	2.444532	-1.017473	-1.411623
22	6	-0.505278	1.895496	2.713395
23	1	-1.347355	-2.874236	1.568468
24	6	-2.140035	-2.820647	0.806902
25	6	-1.580371	-3.051499	-0.590798
26	1	-2.905954	-3.586936	1.036634
27	6	-3.718446	-1.315885	-0.097189
28	1	-0.745182	-2.350864	-0.775651
29	1	-1.213289	-4.084939	-0.689039
30	6	-3.132183	-1.554280	-1.481083
31	1	-4.068895	-0.278879	0.012096
32	1	-4.568108	-2.003312	0.080288
33	1	-3.914990	-1.469896	-2.250360
34	1	-2.344373	-0.804166	-1.677351
35	8	-2.725555	-1.525320	0.906648
36	8	-2.594196	-2.865909	-1.564369

E(UB3LYP) = -879.151678673
Zero-point correction = 0.293976 (Hartree/Particle)
Sum of electronic and thermal Energies = -878.840762
Sum of electronic and thermal Enthalpies = -878.839818
Sum of electronic and thermal Free Energies = -878.904191

TS-8

Center Number	Atomic Number	Coordinates (Angstroms) X	Y	Z
1	6	-1.620471	-1.414348	0.813473
2	6	-3.787671	-0.562298	0.907599
3	7	-2.878966	0.477845	0.360366
4	7	-1.558734	-0.066404	0.399719
5	6	-0.571086	0.618268	-0.262593
6	8	-0.852163	1.583135	-0.966324
7	6	0.830955	0.123019	-0.095740
8	6	1.325242	-0.361029	1.125857
9	6	2.661213	-0.753210	1.237184
10	6	3.512112	-0.666652	0.131468
11	6	3.028424	-0.169219	-1.083755
12	6	1.698175	0.234569	-1.193557
13	1	-0.717109	-2.019481	0.805937
14	1	-4.260466	-0.205157	1.841045
15	1	-4.601865	-0.811328	0.205582
16	1	-3.071393	0.712832	-0.616025
17	1	0.672752	-0.404932	1.999769
18	1	3.041160	-1.118760	2.194035
19	1	4.555944	-0.977289	0.219221
20	1	3.693941	-0.090951	-1.946614
21	1	1.310556	0.644358	-2.127848
22	6	-2.874942	-1.729232	1.151894
23	1	-3.445086	-3.085951	1.209323
24	6	-4.130995	-4.071978	0.906754
25	6	-3.340414	-4.935663	-0.053309
26	1	-4.375502	-4.585660	1.857065
27	6	-6.100451	-4.518400	-0.281361
28	1	-2.924104	-4.297980	-0.859065
29	1	-2.510761	-5.442532	0.461054
30	6	-5.299557	-5.372329	-1.254946
31	1	-6.923073	-3.995869	-0.791849
32	1	-6.525229	-5.161741	0.513947

INT-E·dioxane

Center Number	Atomic Number	Coordinates (Angstroms) X	Y	Z
1	6	-0.305951	0.251270	1.855552
2	6	-2.482575	0.972110	2.154447
3	7	-2.058616	1.317223	0.781179
4	7	-0.667480	0.997982	0.720419
5	6	-0.002645	1.178002	-0.465071
6	8	-0.604513	1.466727	-1.494727
7	6	1.484831	0.992974	-0.444109
8	6	2.292668	1.437984	0.614169
9	6	3.680250	1.291955	0.549545
10	6	4.271986	0.699560	-0.569791
11	6	3.473509	0.272023	-1.636229
12	6	2.089124	0.430149	-1.578974
13	1	0.693759	-0.167954	1.930348
14	1	-2.661309	1.887017	2.754171
15	1	-3.436746	0.419754	2.128441
16	1	-2.513412	0.685085	0.114652
17	1	1.839274	1.924673	1.479581
18	1	4.302082	1.650348	1.373146
19	1	5.357108	0.581197	-0.616363
20	1	3.933781	-0.179948	-2.517850
21	1	1.456355	0.126135	-2.414323
22	6	-1.330601	0.169680	2.714112
23	1	-1.320490	-0.344160	3.673659
24	6	-2.856583	-2.207863	0.207793
25	6	-1.654625	-3.089854	0.334614
26	1	-3.480644	-1.998125	1.080886
27	6	-2.076758	-1.398386	-1.876633
28	1	-0.898229	-2.606597	1.001182
29	1	-1.931645	-4.049206	0.798034
30	6	-0.791102	-2.162371	-1.612149
31	1	-1.869686	-0.413258	-2.313772
32	1	-2.732678	-1.985376	-2.541972

33	1	-5.912376	-6.200863	-1.641369	33	1	-0.305691	-2.421659	-2.564952
34	1	-4.972544	-4.745752	-2.109679	34	1	-0.088081	-1.533884	-1.030386
35	8	-5.264377	-3.521153	0.298444	35	8	-2.783830	-1.148931	-0.650675
36	8	-4.176108	-5.942048	-0.607719	36	8	-1.057038	-3.365710	-0.919926

E(UB3LYP) = -879.136818328	E(UB3LYP) = -879.188157329
Zero-point correction = 0.287703 (Hartree/Particle)	Zero-point correction = 0.293143 (Hartree/Particle)
Sum of electronic and thermal Energies = -878.832506	Sum of electronic and thermal Energies = -878.878071
Sum of electronic and thermal Enthalpies = -878.831562	Sum of electronic and thermal Enthalpies = -878.877127
Sum of electronic and thermal Free Energies = -878.895438	Sum of electronic and thermal Free Energies = -878.941058

TS-9 / **INT-F**·dioxane

Center	Atomic	Coordinates (Angstroms)			Center	Atomic	Coordinates (Angstroms)		
Number	Number	X	Y	Z	Number	Number	X	Y	Z
1	6	-0.312172	0.958261	2.261668	1	6	0.545163	1.689453	2.112941
2	6	-2.599781	1.038352	2.100959	2	6	-1.659143	1.306637	2.188340
3	7	-2.023935	1.502378	0.856199	3	7	-1.158615	0.754357	1.049387
4	7	-0.638738	1.226328	0.926579	4	7	0.185950	0.977921	0.981906
5	6	0.036873	0.968637	-0.243827	5	6	0.883248	0.395878	-0.091606
6	8	-0.578560	0.878846	-1.305054	6	8	0.247412	-0.404944	-0.809227
7	6	1.517875	0.802565	-0.157037	7	6	2.295818	0.737637	-0.301173
8	6	2.330034	1.610189	0.654986	8	6	2.915430	1.944343	0.095053
9	6	3.716193	1.439365	0.653327	9	6	4.258160	2.184727	-0.197478
10	6	4.302037	0.461736	-0.156114	10	6	5.018053	1.235496	-0.888856
11	6	3.498793	-0.333187	-0.981318	11	6	4.408579	0.045743	-1.310210
12	6	2.115823	-0.155262	-0.991495	12	6	3.067496	-0.196130	-1.031549
13	1	0.723494	0.809902	2.550387	13	1	1.582153	1.918406	2.321096
14	1	-3.449496	1.650384	2.439985	14	1	-2.718354	1.232504	2.417424
15	1	-3.110729	-0.096397	1.876074	15	1	-3.972913	-0.957214	1.229432
16	1	-2.401388	1.022287	0.030047	16	1	-1.631043	0.173613	0.331569
17	1	1.882314	2.395775	1.266414	17	1	2.339037	2.726243	0.589167
18	1	4.341888	2.079489	1.279597	18	1	4.712055	3.130939	0.108044
19	1	5.386164	0.326651	-0.152759	19	1	6.071146	1.425268	-1.108649
20	1	3.954479	-1.090276	-1.623613	20	1	4.987915	-0.698021	-1.863347
21	1	1.478714	-0.751891	-1.646616	21	1	2.580680	-1.112385	-1.367873
22	6	-1.439136	0.896647	3.002377	22	6	-0.609051	1.905167	2.873300
23	1	-1.487851	0.695671	4.070656	23	1	-0.663223	2.432105	3.822359
24	6	-3.400276	-1.361645	1.019223	24	6	-3.748911	-1.542711	0.324320
25	6	-2.339137	-2.337139	1.446882	25	6	-3.065885	-2.857949	0.672811
26	1	-4.452061	-1.647961	1.185381	26	1	-4.697619	-1.742953	-0.209439
27	6	-2.700323	-1.657880	-1.258400	27	6	-2.489000	-1.441186	-1.676195
28	1	-1.420765	-1.790752	1.750003	28	1	-2.172754	-2.648670	1.297037
29	1	-2.684265	-2.926353	2.308700	29	1	-3.747107	-3.506784	1.244815
30	6	-1.582588	-2.548201	-0.742625	30	6	-1.830479	-2.759889	-1.303919
31	1	-2.342462	-1.009866	-2.069845	31	1	-1.773992	-0.787092	-2.189640
32	1	-3.538460	-2.283406	-1.617976	32	1	-3.374347	-1.617820	-2.315998
33	1	-1.303305	-3.287486	-1.509068	33	1	-1.598727	-3.342789	-2.207960
34	1	-0.689870	-1.931707	-0.512822	34	1	-0.890032	-2.540739	-0.766951
35	8	-3.180316	-0.785549	-0.228565	35	8	-2.904205	-0.743816	-0.497017
36	8	-1.999834	-3.256070	0.407908	36	8	-2.697179	-3.550620	-0.501233

E(UB3LYP) = -879.169593733	E(UB3LYP) = -879.232199661
Zero-point correction = 0.288762 (Hartree/Particle)	Zero-point correction = 0.293567 (Hartree/Particle)
Sum of electronic and thermal Energies = -878.864939	Sum of electronic and thermal Energies = -878.922202
Sum of electronic and thermal Enthalpies = -878.863995	Sum of electronic and thermal Enthalpies = -878.921258
Sum of electronic and thermal Free Energies = -878.924901	Sum of electronic and thermal Free Energies = -878.983970

INT-G / 1,4-dioxane

Center	Atomic	Coordinates (Angstroms)			Center	Atomic	Coordinates (Angstroms)		
Number	Number	X	Y	Z	Number	Number	X	Y	Z
1	6	-0.330857	1.080384	2.375570	1	1	-3.534890	-2.956629	0.042970
2	6	-2.556727	1.304115	2.161073	2	6	-3.853091	-4.006188	0.138067
3	7	-1.994881	1.228891	0.916865	3	6	-4.452754	-4.511908	-1.167853
4	7	-0.641243	1.059078	1.025295	4	1	-2.965980	-4.620887	0.395413
5	6	0.030601	0.784284	-0.176489	5	6	-5.310861	-5.381042	1.339683
6	8	-0.684489	0.613396	-1.183311	6	1	-5.265842	-3.827204	-1.485834
7	6	1.497709	0.697072	-0.178554	7	1	-3.691842	-4.543064	-1.962771
8	6	2.349416	1.353711	0.735330	8	6	-5.910524	-5.886763	0.033764

9	6	3.735412	1.235934	0.623991
10	6	4.303561	0.465890	-0.395718
11	6	3.468049	-0.169981	-1.323616
12	6	2.085516	-0.048860	-1.224349
13	1	0.681168	0.911539	2.719788
14	1	-3.629982	1.418489	2.281246
15	1	-2.372733	1.029555	-0.008413
16	1	1.937512	2.002292	1.508385
17	1	4.377765	1.761982	1.334640
18	1	5.388992	0.371573	-0.474981
19	1	3.902046	-0.763111	-2.132472
20	1	1.425764	-0.527549	-1.949326
21	6	-1.525871	1.236075	3.088805
22	1	-1.621584	1.289325	4.170361

E(UB3LYP) = -571.410764626
Zero-point correction = 0.170194 (Hartree/Particle)
Sum of electronic and thermal Energies = -571.230599
Sum of electronic and thermal Enthalpies = -571.229654
Sum of electronic and thermal Free Energies = -571.276803

9	1	-6.071771	-5.349887	2.134602
10	1	-4.497771	-6.065745	1.657663
11	1	-6.228724	-6.936322	0.128861
12	1	-6.797637	-5.272065	-0.223581
13	8	-4.808423	-4.065902	1.182481
14	8	-4.955193	-5.827047	-1.010652

E(UB3LYP) = -307.807779725
Zero-point correction = 0.121350 (Hartree/Particle)
Sum of electronic and thermal Energies = -307.681395
Sum of electronic and thermal Enthalpies = -307.680451
Sum of electronic and thermal Free Energies = -307.714672

Calculations of redox potentials

Redox potentials were obtained by Eqn. S1 using calculated energies E_{ox} and E_{red} at UB3LYP-D3/def2- TZTVPP//UB3LYP-D3/def2-SVP level of theory.

$$E°_{calc}\ (vs.\ \mathrm{SCE}) = \frac{E_A}{nF} - 4.44 - 0.241 \quad \cdots \text{(S1)}$$

where E_A is electorn affinity ($E_{ox} - E_{red}$)
$n = 1$
$F = 23.061$ kcal mol^{-1} V^{-1}

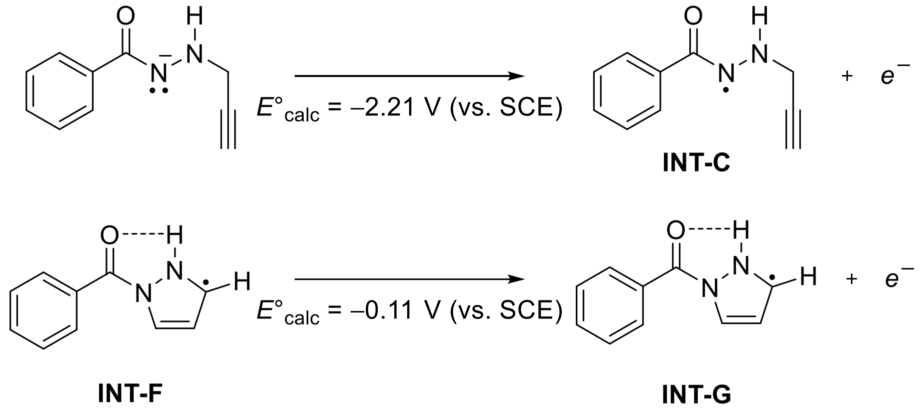

References

(1) For *Gaussian 09*, Revision D.01, Frisch, M. J.; Trucks, G. W.; Schlegel, H. B.; Scuseria, G. E.; Robb, M. A.; Cheeseman, J. R.; Scalmani, G.; Barone, V.; Mennucci, B.; Petersson, G. A.; Nakatsuji, H.; Caricato, M.; Li, X.; Hratchian, H. P.; Izmaylov, A. F.; Bloino, J.; Zheng, G.; Sonnenberg, J. L.; Hada, M.; Ehara, M.; Toyota, K.; Fukuda, R.; Hasegawa, J.; Ishida, M.; Nakajima, T.; Honda, Y.; Kitao, O.; Nakai, H.; Vreven, T.; Montgomery, Jr., J. A.; Peralta, J. E.; Ogliaro, F.; Bearpark, M.; Heyd, J. J.; Brothers, E.; Kudin, K. N.; Staroverov, V. N.; Keith, T.; Kobayashi, R.; Normand, J.; Raghavachari, K.; Rendell, A.; Burant, J. C.; Iyengar, S. S.; Tomasi, J.; Cossi, M.; Rega, N.; Millam, J. M.; Klene, M.; Knox, J. E.; Cross, J. B.; Bakken, V.; Adamo, C.; Jaramillo, J.; Gomperts, R.; Stratmann, R. E.; Yazyev, O.; Austin, A. J.; Cammi, R.; Pomelli, C.; Ochterski, J. W.; Martin, R. L.; Morokuma, K.; Zakrzewski, V. G.; Voth, G. A.; Salvador, P.; Dannenberg, J. J.; Dapprich, S.; Daniels, A. D.; Farkas, O.; Foresman, J. B.; Ortiz, J. V.; Cioslowski, J.; Fox, D. J. Gaussian, Inc., Wallingford CT, 2013.

4.- Experimental Section

General procedure for the synthesis of propargyl hydrazides 1. *N,N*-Diisopropylethylamine (DIPEA) (323 mg, 2.5 mmol, 2.5 equiv) and hexafluorophosphate azabenzotriazole tetramethyl uronium (HATU) (382 mg, 1.0 mmol, 1.0 equiv) were sequentially added at room temperature to a solution of the corresponding carboxylic acid (1.0 mmol, 1.0 equiv) in dry DMF (4 mL). The reaction was stirred for 15 min and then prop-2-yn-1-ylhydrazine hydrochloride (160 mg, 1.5 mmol, 1.5 equiv) and DIPEA (194 mg, 1.5 mmol, 1.5 equiv) were sequentially added. DCM (20 mL) was added, and the organic layer was separated, washed with brine (3 x 20 mL) and dried over anhydrous $MgSO_4$. After the removal of $MgSO_4$ by filtration, the filtrate was concentrated in vacuo. The residue was purified by flash chromatography on silica gel eluting with ethyl acetate/hexanes mixtures to afford the corresponding propargyl hydrazide. Spectroscopic and analytical data for compounds **1** follow.

Propargyl hydrazide 1a

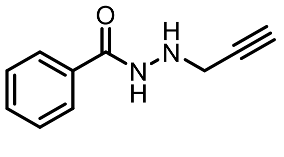

Following the general procedure and starting from 300 mg (2.46 mmol) of benzoic acid, compound **1a** (68%, 290 mg, 1.66 mmol) was obtained as a colorless solid, after purification by column chromatography using *n*-hexane/AcOEt (7:1 → 4:1) as eluent; mp 76-78 ºC; ^{1}H NMR (400 MHz, $CDCl_3$, 25 ºC): δ 7.92 (1H, s, NH), 7.79 (2H, dd, *J* = 8.1, 1.4 Hz, $2CH_{Ar}$), 7.58-7.51 (1H, m, CH_{Ar}), 7.49-7.43 (2H, m, $2CH_{Ar}$), 3.78 (2H, d, *J* = 2.4 Hz, CH_2), 3.40 (1H, s, NH), 2.30 (1H, t, *J* = 2.5 Hz, CH); ^{13}C {^{1}H} NMR (101 MHz, $CDCl_3$, 25 ºC): δ 167.4 (C=O), 132.4 (C_{Ar}), 132.0 (CH_{Ar}), 128.6 ($2CH_{Ar}$), 127.0 ($2CH_{Ar}$), 79.8 (C), 72.6 (CH), 41.2 (CH_2); IR (cm^{-1}): ν 3286 (N-H), 3226 (N-H), 1626 (C=O), 1540 (N-C=O); HRMS (ESI) m/z: $[M+H]^+$ calcd for $C_{10}H_{11}N_2O$: 175.0866; found 175.0860.

Propargyl hydrazide 1b

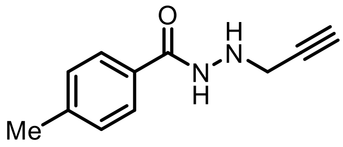

Following the general procedure and starting from 300 mg (2.20 mmol) of *p*-toluic acid, compound **1b** (87%, 360.8 mg, 1.92 mmol) was obtained as a colorless solid, after purification by column chromatography using *n*-hexane/AcOEt (7:1 → 5:1) as eluent; mp 80-82 ºC; ^{1}H NMR (400 MHz, $CDCl_3$, 25 ºC): δ 7.69 (1H, s, NH), 7.69-7.64 (2H, m, $2CH_{Ar}$), 7.26 (2H, d, *J* = 7.4 Hz, $2CH_{Ar}$), 5.01 (1H, s, NH), 3.75 (2H, d, *J* = 2.6 Hz, CH_2), 2.41 (3H, s, CH_3), 2.28 (1H, t, *J* = 2.5 Hz, CH); ^{13}C {^{1}H} NMR (101 MHz, $CDCl_3$, 25 ºC): δ 167.4 (C=O), 142.6 (C_{Ar}), 129.6 (C_{Ar}), 129.4 ($2CH_{Ar}$), 126.9 ($2CH_{Ar}$), 79.8 (C), 72.5 (CH), 41.3 (CH_2), 21.5 (CH_3); IR (cm^{-1}): ν 3310 (N-H), 3281 (N-H), 1625 (C=O), 1538 (N-C=O); HRMS (ESI) m/z: $[M+H]^+$ calcd for $C_{11}H_{13}N_2O$: 189.1022; found 189.1020.

Propargyl hydrazide 1c

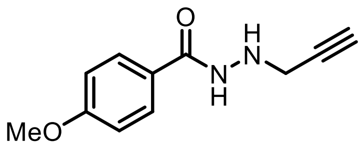

Following the general procedure and starting from 300 mg (1.97 mmol) of *p*-anisic acid, compound **1c** (88%, 355.5 mg, 1.74 mmol) was obtained as a colorless solid, after purification by column chromatography using *n*-hexane/AcOEt (7:1 → 2:1) as eluent; mp 135-137 ºC; ^{1}H NMR (400 MHz,

$CDCl_3$, 25 ºC): δ 7.81-7.72 (2H, m, $2CH_{Ar}$), 7.70 (1H, s, NH), 7.01-6.89 (2H, m, 2HAr), 5.00 (1H, s, NH), 3.86 (3H, s, CH_3), 3.74 (2H, d, J = 2.5 Hz, CH_2), 2.28 (1H, t, J = 2.5 Hz, CH); ^{13}C {^1H} NMR (101 MHz, $CDCl_3$, 25 ºC): δ 167.1 (C=O), 162.6 (C_{Ar}-O), 128.79 ($2CH_{Ar}$), 124.7 (C_{Ar}), 114.0 ($2CH_{Ar}$), 79.9 (C), 72.5 (CH), 55.4 (CH_3-O), 41.3 (CH_2); IR (cm^{-1}): ν 3278 (N-H), 3227 (N-H), 1613 (C=O), 1511 (N-C=O), 1249 (C-O-C); HRMS (ESI) m/z: $[M+Na]^+$ calcd for $C_{11}H_{12}N_2NaO_2$: 227.0791; found 227.0785.

Propargyl hydrazide 1-N

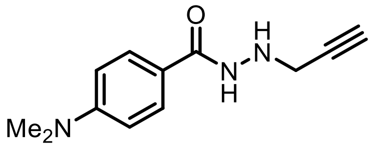

Following the general procedure and starting from 300 mg (1.82 mmol) of 4-(dimethylamino)benzoic acid, compound **1-N** (59%, 231 mg, 1.06 mmol) was obtained as a yellow solid, after purification by column chromatography using *n*-hexane/AcOEt (5:1 → 1:1) as eluent; mp 85-87 ºC; ^1H NMR (300 MHz, $CDCl_3$, 25 ºC): δ 7.68 (d, J = 9.1 Hz, 2H, $2CH_{Ar}$), 7.66 (s, 1H, NH), 6.68 (d, J = 9.0 Hz, 2H, $2CH_{Ar}$), 3.73 (d, J = 2.5 Hz, 2H, CH_2), 3.03 (s, 6H, $2CH_3$), 3.02 (s, 1H, NH), 2.27 (t, J = 2.5 Hz, 1H, CH); ^{13}C {^1H} NMR (75 MHz, $CDCl_3$, 25 ºC): δ 167.6 (C=O), 152.8 (C_{Ar}), 128.4 ($2CH_{Ar}$), 118.9 (C_{Ar}), 111.1 ($2CH_{Ar}$), 80.1 (C), 72.3 (CH), 41.4 (CH_2), 40.1 ($2CH_3$); IR (cm^{-1}): ν 3281 (N-H), 1610 (C=O), 1515 (N-C=O) ; HRMS (ESI) m/z: $[M+Na]^+$ calcd for $C_{12}H_{15}N_3NaO^+$: 240.1107; found 240.1109.

Propargyl hydrazide 1d

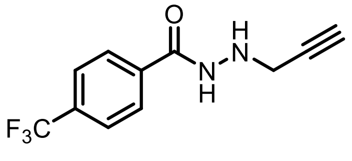

Following the general procedure and starting from 40 mg (0.21 mmol) of 4-(trifluoromethyl)benzoic acid, compound **1d** (34%, 17.5 mg, 0.07 mmol) was obtained as a withe solid, after purification by column chromatography using *n*-hexane/AcOEt (6:4) as eluent; mp 118-120 ºC; ^1H NMR (600 MHz, $CDCl_3$, 25 ºC): δ 7.96 (s, 1H, NH), 7.89 (d, J = 8.2 Hz, 2H, CH_{Ar}), 7.72 (d, J = 8.2 Hz, 2H, CH_{Ar}),

3.77 (d, *J* = 1.9 Hz, 2H, CH_2), 2.29 (t, *J* = 2.5 Hz, 1H, CH); ^{13}C {^1H} (151 MHz, $CDCl_3$): δ 166.32 (C=O), 135.93 (C_{Ar}), 133.97 (d, J = 32.7 Hz, CF_3), 127.67 (2 CH_{Ar}), 125.98 (2 CH_{Ar}), 122.78 (C_{Ar}), 79.62 (C), 73.04 (CH) , 41.38 (CH_2); IR (cm^{-1}): ν 3307, 1640, 1330, 1117; HRMS m/z (ESI): $[M-H]^-$ calc. for $C_{11}H_8F_3N_2O$: 241.0594; found 241.0594.

Propargyl hydrazide 1e

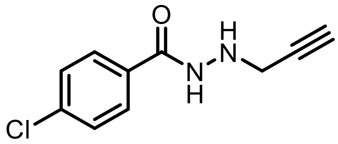

Following the general procedure and starting from 300 mg (1.92 mmol) of *p*-chlorobenzoic acid, compound **1e** (59%, 237.9 mg, 1.14 mmol) was obtained as a yellow solid, after purification by column chromatography using *n*-hexane/AcOEt (7:1 → 3:1) as eluent; mp 110-112 ºC; ^1H NMR (400 MHz, $CDCl_3$, 25 ºC): δ 7.85 (1H, s, NH), 7.75-7.69 (2H, m, 2CH_{Ar}), 7.47-7.41 (2H, m, 2CH_{Ar}), 3.78 (2H, s, CH_2), 2.29 (1H, t, *J* = 2.2 Hz, CH); ^{13}C {^1H} NMR (101 MHz, $CDCl_3$, 25 ºC): δ 166.5 (C=O), 138.6 (2C_{Ar}), 129.2 (2CH_{Ar}), 128.7 (2CH_{Ar}), 79.8 (C), 73.0 (CH), 41.3 (CH_2); IR (cm^{-1}): ν 3277 (N-H), 3158 (N-H), 1630 (C=O), 1527 (N-C=O); HRMS (ESI) m/z: $[M+Na]^+$ calcd for $C_{10}H_9ClN_2NaO$: 231.0296; found 231.0294.

Propargyl hydrazide 1f

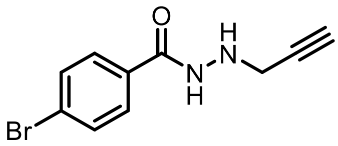

Following the general procedure and starting from 161.8 mg (0.80 mmol) of *p*-bromobenzoic acid, compound **1f** (82%, 165.8 mg, 0.66 mmol) was obtained as a yellow solid, after purification by column chromatography using *n*-hexane/AcOEt (7:1 → 3:1) as eluent; mp 118-120 ºC; ^1H NMR (400 MHz, $CDCl_3$, 25 ºC): δ 7.77 (1H, s, NH), 7.67-7.63 (2H, m, 2CH_{Ar}), 7.63-7.58 (2H, m, 2CH_{Ar}), 5.01 (1H, s, NH), 3.75 (2H, d, *J* = 2.9 Hz, CH_2), 2.29 (1H, t, *J* = 2.5 Hz, CH); ^{13}C {^1H} NMR (101 MHz, $CDCl_3$, 25 ºC): δ 166.5 (C=O), 132.0 (2CH_{Ar}), 131.3 (C_{Ar}), 128.5 (2CH_{Ar}), 126.9 (C_{Ar}), 79.6 (C),

72.7 (CH), 41.3 (CH_2); IR (cm^{-1}): ν 3278 (N-H), 3158 (N-H), 1632 (C=O), 1527 (N-C=O); HRMS (ESI) m/z: $[M+H]^+$ calcd for $C_{10}H_{10}BrN_2O$: 252.9971; found 252.9970.

Propargyl hydrazide 1g

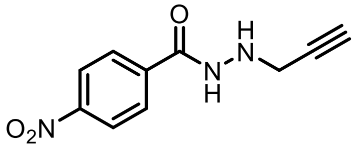

Following the general procedure and starting from 600 mg (3.59 mmol) of *p*-nitrobenzoic acid, compound **1g** (31%, 239.4 mg, 1.09 mmol) was obtained as a yellow solid, after purification by column chromatography using *n*-hexane/AcOEt (7:1 → 4:1) as eluent; mp 123-125 ºC; ^{1}H NMR (400 MHz, $CDCl_3$, 25 ºC): δ 10.44 (1H, d, J = 6.4 Hz, NH), 8.34-8.30 (2H, m, $2CH_{Ar}$), 8.07-8.04 (2H, m, $2CH_{Ar}$), 5.59 (1H, q, J = 5.7 Hz, NH), 3.63 (2H, dd, J = 5.5, 2.5 Hz, CH_2), 3.12 (1H, t, J = 2.5 Hz, CH); ^{13}C {^{1}H} NMR (101 MHz, $CDCl_3$, 25 ºC): δ 163.7 (C=O), 149.1 (C_{Ar}), 138.7 (C_{Ar}), 128.6 ($2CH_{Ar}$), 123.6 ($2CH_{Ar}$), 81.1 (C), 74.6 (CH), 39.5 (CH_2); IR (cm^{-1}): ν 3290 (N-H), 3187 (N-H), 1632 (C=O), 1594 (N-C=O), 1519 (N=O), 1347 (N=O); HRMS (ESI) m/z: $[M+Na]^+$ calcd for $C_{10}H_9N_3NaO_3$: 242.0536; found 242.0536.

Propargyl hydrazide 1h

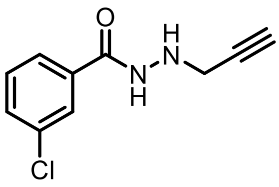

Following the general procedure and starting from 300 mg (1.92 mmol) of *m*-chlorobenzoic acid, compound **1h** (56%, 222 mg, 1.06 mmol) was obtained as a yellow solid, after purification by column chromatography using *n*-hexane/AcOEt (7:1 → 5:1) as eluent; mp 110-112 ºC; ^{1}H NMR (400 MHz, $CDCl_3$, 25 ºC): δ 7.99 (1H, s, NH), 7.79 (1H, d, J = 1.9 Hz, CH_{Ar}), 7.66 (1H, d, J = 7.7 Hz, CH_{Ar}), 7.52 (1H, dd, J = 7.9, 2.2 Hz, CH_{Ar}), 7.41 (1H, t, J = 7.9 Hz, CH_{Ar}), 3.77 (2H, d, J = 2.4 Hz, CH_2), 3.60 (1H, s, NH), 2.31 (1H, t, J = 2.5 Hz, CH); ^{13}C {^{1}H} NMR (101 MHz, $CDCl_3$, 25 ºC): δ 166.1 (C=O), 135.0 (C_{Ar}), 134.2 (C_{Ar}), 132.2 (CH_{Ar}), 130.1 (CH_{Ar}), 127.4 (CH_{Ar}), 125.1 (CH_{Ar}), 79.4 (C),

73.0 (CH), 41.2 (CH_2); IR (cm^{-1}): ν 3286 (N-H), 3226 (N-H), 1638 (C=O), 1524 (N-C=O); HRMS (ESI) m/z: $[M+H]^+$ calcd for $C_{10}H_{10}ClN_2O$: 209.0476; found 209.0473.

Propargyl hydrazide 1i

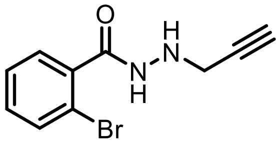

Following the general procedure and starting from 300 mg (1.49 mmol) of *o*-bromobenzoic acid, compound **1i** (75%, 284.3 mg, 1.12 mmol) was obtained as a colorless solid, after purification by column chromatography using *n*-hexane/AcOEt (7:1 → 3:1) as eluent; mp 118-120 ºC; ^1H NMR (500 MHz, $CDCl_3$, 25 ºC): δ 7.62 (1H, dd, J = 8.0, 1.2 Hz, CH_{Ar}), 7.60 (1H, s, NH), 7.54 (1H, dd, J = 7.6, 1.8 Hz, CH_{Ar}), 7.39 (1H, td, J = 7.5, 1.2 Hz, CH_{Ar}), 7.32 (1H, ddd, J = 8.0, 7.4, 1.8 Hz, CH_{Ar}), 3.80 (2H, d, J = 2.5 Hz, CH_2), 2.30 (1H, t, J = 2.5 Hz, CH); ^{13}C {^1H} NMR (126 MHz, $CDCl_3$, 25 ºC): δ 167.4 (C=O), 135.7 (C_{Ar}), 133.7 (CH_{Ar}), 132.0 (CH_{Ar}), 129.9 (CH_{Ar}), 127.7 (CH_{Ar}), 119.8 (C_{Ar}), 79.7 (C), 73.0 (CH), 41.3 (CH_2); IR (cm^{-1}): ν 3272 (N-H), 3240 (N-H), 1628 (C=O), 1545 (N-C=O); HRMS (ESI) m/z: $[M+H]^+$ calcd for $C_{10}H_{10}BrN_2O$: 252.9971; found 252.9974.

Propargyl hydrazide 1j

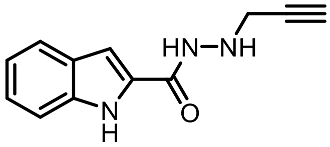

Following the general procedure and starting from 300 mg (1.86 mmol) of 1*H*-indole-2-carboxylic acid, compound **1j** (32%, 125.7 mg, 0.59 mmol) was obtained as a yellow solid, after purification by column chromatography using *n*-hexane/AcOEt (5:1 → 1:1) as eluent; mp 116-118 ºC; ^1H NMR (400 MHz, dmso-d_6, 25 ºC): δ 11.62 (1H, s, NH), 10.10 (1H, d, J = 6.0 Hz, NH), 7.59 (1H, d, J = 8.0 Hz, H4), 7.42 (1H, d, J = 8.2 Hz, H7), 7.17 (1H, ddd, J = 8.2, 6.9, 1.1 Hz, H5), 7.12 (1H, d, J = 1.4 Hz, H3), 7.03 (1H, ddd, J = 8.0, 6.9, 1.0 Hz, H6), 5.46 (1H, q, J = 5.5 Hz, NH), 3.64 (2H, dd, J = 5.3, 2.5 Hz, CH_2), 3.12 (1H, t, J = 2.5 Hz, CH); ^{13}C {^1H} NMR (101 MHz, dmso-d_6, 25 ºC): δ 160.8 (C=O), 136.4 (C3a), 130.0 (C2), 127.0 (C7a), 123.3 (C5), 121.5 (C4), 119.8 (C6), 112.3 (C7), 102.5 (C3),

81.3 (C), 74.5 (CH), 39.5 (CH_2); IR (cm^{-1}): ν 3305 (NH), 3293 (N-H), 3248 (N-H), 1617 (C=O), 1524 (N-C=O); HRMS (ESI) m/z: $[M+H]^+$ calcd for $C_{12}H_{12}N_3O$: 214.0975; found 214.0971.

Propargyl hydrazide 1k

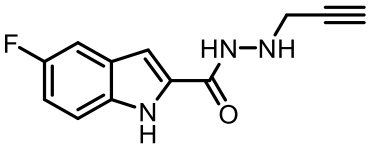

Following the general procedure and starting from 300 mg (1.67 mmol) of 5-fluoroindole-2-carboxylic acid, compound **1k** (34%, 131.5 mg, 0.57 mmol) was obtained as a yellow solid, after purification by column chromatography using *n*-hexane/AcOEt (5:1 → 3:1) as eluent; mp 155-157 ºC; ^1H NMR (400 MHz, dmso-d_6, 25 ºC): δ 11.74 (1H, s, NH), 10.15 (1H, d, *J* = 6.1 Hz, NH-CO), 7.60-7.27 (2H, m, H4, H7), 7.10 (1H, dd, *J* = 2.2, 0.9 Hz, H3), 7.04 (1H, td, *J* = 9.3, 2.6 Hz, H6), 5.48 (1H, q, *J* = 5.5 Hz, N*H*-CH_2), 3.64 (2H, dd, *J* = 5.2, 2.5 Hz, CH_2), 3.12 (1H, t, *J* = 2.5 Hz, CH); ^{13}C {^1H} NMR (101 MHz, dmso-d_6, 25 ºC): δ 160.5 (C=O), 157.2 (d, *J* = 232.6 Hz, C5), 133.2 (C7a), 131.8 (C2), 127.1 (d, *J* = 10.7 Hz, C3a), 113.4 (d, *J* = 9.8 Hz, C7), 112.1 (d, *J* = 26.7 Hz, C6), 105.7 (d, *J* = 23.1 Hz, C4), 102.5 (d, *J* = 5.1 Hz, C3), 81.3 (CH*C*CH_2), 74.5 (*C*HCCH_2), 39.5 (CHCC*H*$_2$); ^{19}F NMR (376 MHz, dmso-d_6, 25ºC): δ -124.8 (CF); IR (cm^{-1}): ν 3288 (N-H), 3187 (N-H), 1634 (C=O), 1515 (N-C=O); HRMS (ESI) m/z: $[M+H]^+$ calcd for $C_{12}H_{11}FN_3O$: 232.0881; found 232.0887.

Propargyl hydrazide 1l

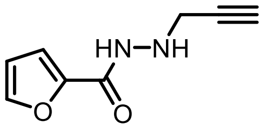

Following the general procedure and starting from 300 mg (2.68 mmol) of furan-2-carboxylic acid, compound **1l** (35%, 153.5 mg, 0.94 mmol) was obtained as a brown solid, after purification by column chromatography using *n*-hexane/AcOEt (6:1 → 3:1) as eluent; mp 98-100 ºC; ^1H NMR (400 MHz, $CDCl_3$, 25 ºC): δ 7.93 (1H, d, *J* = 5.9 Hz, NH), 7.47 (1H, dd, *J* = 1.8, 0.8 Hz, H5), 7.17 (1H, dd, *J* = 3.5, 0.8 Hz, H3), 6.53 (1H, dd, *J* = 3.5, 1.8 Hz, H4), 4.88 (1H, q, *J* = 5.6 Hz, NH), 3.74 (2H, dd, *J* = 5.1, 2.5 Hz, CH_2), 2.29 (1H, t, *J* = 2.5 Hz, CH); ^{13}C {^1H} NMR (101 MHz, $CDCl_3$, 25 ºC): δ 158.3 (C=O), 146.6 (C2), 144.6 (C5), 115.4 (C3), 112.3 (C4), 79.7 (C), 72.9 (CH), 41.5 (CH_2); IR (cm^{-1}):

ν 3284 (N-H), 3244 (N-H), 1652 (C=O), 1589 (N-C=O), 1309 (C-O-C); HRMS (ESI) m/z: $[M+H]^+$ calcd for $C_8H_9N_2O_2$: 165.0659; found 165.0657.

Propargyl hydrazide 1m

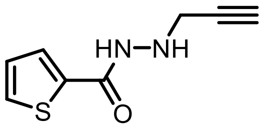

Following the general procedure and starting from 200 mg (1.56 mmol) of thiophene-2-carboxylic acid, compound **1m** (36%, 99.5 mg, 0.55 mmol) was obtained as a colorless solid, after purification by column chromatography using *n*-hexane/AcOEt (7:1 → 3:1) as eluent; mp 70-72 ºC; ^{1}H NMR (400 MHz, dmso-d_6, 25 ºC): δ 10.11 (1H, d, J = 6.0 Hz, NH), 7.78 (1H, dd, J = 5.0, 1.1 Hz, H3), 7.75 (1H, dd, J = 3.8, 1.2 Hz, H5), 7.14 (1H, dd, J = 5.0, 3.7 Hz, H4), 5.40 (1H, q, J = 5.4 Hz, NH), 3.60 (2H, dd, J = 5.2, 2.5 Hz, CH_2), 3.11 (1H, t, J = 2.5 Hz, CH); ^{13}C {^{1}H} NMR (101 MHz, dmso-d_6, 25 ºC): δ 160.8 (C=O), 137.9 (C2), 130.9 (C3), 128.1 (C5), 128.0 (C4), 81.2 (C), 74.5 (CH), 39.5 (CH_2); IR (cm^{-1}): ν 3277 (N-H), 3238 (N-H), 1642 (C=O), 1536 (N-C=O); HRMS (ESI) m/z: $[M+H]^+$ calcd for $C_8H_9N_2OS$: 181.0430; found 181.0431.

Propargyl hydrazide 1n

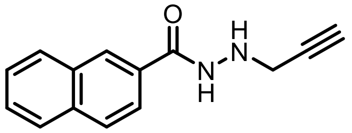

Following the general procedure and starting from 200 mg (1.16 mmol) of 2-naphthoic acid, compound **1n** (89%, 232 mg, 1.04 mmol) was obtained as a colorless solid, after purification by column chromatography using *n*-hexane/AcOEt (5:1 → 4:1) as eluent; mp 117-119 ºC; ^{1}H NMR (300 MHz, $CDCl_3$, 25 ºC): δ 8.35–8.25 (m, 1H, CH_{Ar}), 8.03–7.77 (m, 5H, NH, 4CH_{Ar}), 7.66–7.50 (m, 2H, 2CH_{Ar}), 5.12 (d, J = 5.9 Hz, 1H, NH), 3.81 (dd, J = 4.9, 2.5 Hz, 2H, CH_2), 2.31 (t, J = 2.5 Hz, 1H, CH); ^{13}C {^{1}H} NMR (75 MHz, $CDCl_3$, 25 ºC): δ 167.6 (C=O), 135.0 (C_{Ar}), 132.6 (C_{Ar}), 129.7 (C_{Ar}), 129.0 (CH_{Ar}), 128.7 (CH_{Ar}), 127.9 (CH_{Ar}), 127.8 (CH_{Ar}), 127.7 (CH_{Ar}), 126.9 (CH_{Ar}), 123.2 (CH_{Ar}), 79.8(C), 72.7 (CH), 41.4 (CH_2); IR (cm^{-1}): ν 3283 (N-H), 3230 (N-H), 1620 (C=O), 1544 (N-C=O); HRMS (ESI) m/z: $[M+H]^+$ calcd for $C_{14}H_{13}N_2O^+$: 225.1022; found 225.1023.

Propargyl hydrazide 1n-N

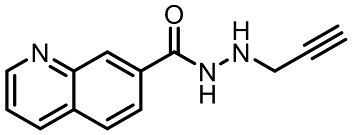

Following the general procedure and starting from 80 mg (0.46 mmol) of quinoline-7-carboxylic acid, compound **1n-N** (46%, 47.8 mg, 0.21 mmol) was obtained as a orange syrup, after purification by column chromatography using AcOEt as eluent; ^{1}H NMR (600 MHz, acetone-d$_6$, 25ºC) δ 9.72 (s, 1H, NH), 8.99 (dd, *J* = 4.2, 1.7 Hz, 1H, CH$_{Ar}$), 8.59 (d, *J* = 1.5 Hz, 1H, CH$_{Ar}$), 8.43–8.36 (m, 1H, CH$_{Ar}$), 8.10–8.05 (m, 2H, CH$_{Ar}$), 7.60 (dd, *J* = 8.2, 4.1 Hz, 1H, CH$_{Ar}$), 5.36 (s, 1H, NH), 3.77 (d, *J* = 2.6 Hz, 2H, CH$_2$), 2.71 (t, J = 2.6 Hz, 1H, CH); ^{13}C {^{1}H} NMR (151 MHz, acetone-d$_6$) δ 168.65(C=O), 154.16 (CH$_{Ar}$), 150.45 (C$_{Ar}$), 138.34 (CH$_{Ar}$), 136.60 (C$_{Ar}$), 132.57 (C$_{Ar}$), 131.21 (CH$_{Ar}$), 131.07 (CH$_{Ar}$), 127.27 (CH$_{Ar}$), 125.30 (CH$_{Ar}$), 83.07 (<u>C</u>≡CH), 75.26 (CH), 43.07 (CH$_2$); IR (cm^{-1}): ν 3294 (N-H), 1656 (C=O), 1570 (N-C=O); HRMS m/z (ESI): [M+H]$^+$ calc. for $C_{13}H_{12}N_3O$: 226.0975; found 226.0976.

Propargyl hydrazide 1o

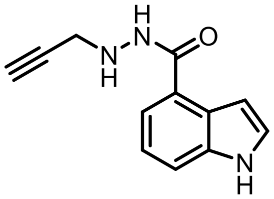

Following the general procedure and starting from 300 mg (1.86 mmol) of 1*H*-indole-4-carboxylic acid, compound **1o** (34%, 320.4 mg, 0.50 mmol) was obtained as a green solid, after purification by column chromatography using *n*-hexane/AcOEt (4:1 → 1:1) as eluent; mp 114-116 ºC; ^{1}H NMR (400 MHz, dmso-d_6, 25 ºC): δ 11.28 (1H, s, NH), 9.82 (1H, d, *J* = 6.3 Hz, NH-CO), 7.54 (1H, dt, *J* = 8.1, 1.0 Hz, H3), 7.43 (1H, t, *J* = 2.8 Hz, H2), 7.39 (1H, dd, *J* = 7.3, 0.9 Hz, H5), 7.16-7.08 (1H, m, H6), 6.86 (1H, t, *J* = 2.1 Hz, H7), 5.42 (1H, q, *J* = 5.7 Hz, N*H*-CH$_2$), 3.63 (2H, dd, *J* = 5.4, 2.5 Hz, CH$_2$), 3.12 (1H, t, *J* = 2.5 Hz, CH); ^{13}C {^{1}H} NMR (101 MHz, dmso-d_6, 25 ºC): δ 167.2 (C=O), 136.5 (C4), 126.5 (C2), 125.9 (C7a), 124.9 (C3a), 120.1 (C6), 118.5 (C5), 114.3 (C3), 101.7 (C7), 81.4

(CHCCH$_2$), 74.4 (CHCCH$_2$), 39.5 (CHCCH_2); IR (cm^{-1}): ν 3271 (N-H), 3240 (N-H), 1631 (C=O), 1511 (N-C=O); HRMS (ESI) m/z: $[M+H]^+$ calcd for $C_{12}H_{12}N_3O$: 214.0975; found 214.0977.

Propargyl hydrazide 1p

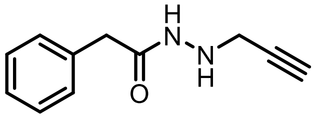

Following the general procedure and starting from 300 mg (2.20 mmol) of 2-phenylacetic acid, compound **1p** (36%, 147.7 mg, 0.78 mmol) was obtained as a white solid, after purification by column chromatography using *n*-hexane/AcOEt (7:1 → 1:1) as eluent; mp 78-80 ºC; ^{1}H NMR (400 MHz, $CDCl_3$, 25 ºC): δ 7.38-7.25 (5H, m, 5CH$_{Ar}$), 7.09 (1H, s, NH), 3.58 (4H, d, J = 3.9 Hz, 2CH$_2$), 2.18 (1H, t, J = 2.5 Hz, CH); ^{13}C {^{1}H} NMR (101 MHz, $CDCl_3$, 25 ºC): δ 170.7 (C=O), 133.9 (C$_{Ar}$), 129.5 (2CH$_{Ar}$), 129.2 (2CH$_{Ar}$), 127.7 (CH$_{Ar}$), 79.64(C), 72.7 (CH), 42.2 (CH$_2$-C$_{Ar}$), 41.1 (CH$_2$-C); IR (cm^{-1}): ν 3265 (N-H), 3078 (N-H), 1634 (C=O), 1548 (N-C=O); HRMS (ESI) m/z: $[M+H]^+$ calcd for $C_{11}H_{13}N_2O$: 189.1022; found 189.1021.

Propargyl hydrazide 1q

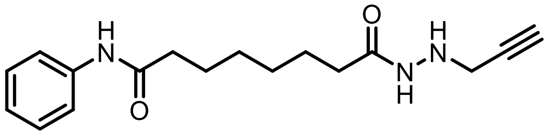

Following the general procedure and starting from 300 mg (1.20 mmol) of 8-oxo-8-(phenylamino)octanoic acid, compound **1q** (60%, 219 mg, 0.73 mmol) was obtained as a colorless solid, after purification by column chromatography using *n*-hexane/AcOEt (4:1 → 1:1) as eluent; mp 130-132 ºC; ^{1}H NMR (400 MHz, dmso-d_6, 25 ºC): δ 9.83 (1H, s, NH), 9.33 (1H, d, J = 6.3 Hz, NH), 7.60-7.53 (2H, m, 2CH$_{Ar}$), 7.32-7.24 (2H, m, 2CH$_{Ar}$), 7.01 (1H, tt, J = 7.4, 1.2 Hz, 1CH$_{Ar}$), 5.14-5.04 (1H, m, NH), 3.50-3.41 (2H, m, CH$_2$), 3.07 (1H, t, J = 2.5 Hz, CH), 2.28 (2H, t, J = 7.5 Hz, CH$_2$), 2.02 (2H, t, J = 7.4 Hz, CH$_2$), 1.63-1.43 (4H, m, 4CH$_2$), 1.34-1.22 (4H, m, 4CH$_2$); ^{13}C {^{1}H} NMR (101 MHz, $CDCl_3$, 25 ºC): δ 171.4 (C=O), 171.2 (C=O), 139.3 (C$_{Ar}$), 128.6 (2CH$_{Ar}$), 122.9 (CH$_{Ar}$), 119.0 (2CH$_{Ar}$), 81.4 (C), 74.2 (CH), 39.5 (CH$_2$), 36.4 (CH$_2$), 33.4 (CH$_2$), 28.4 (CH$_2$), 28.4 (CH$_2$),

25.1 (CH_2), 25.0 (CH_2); IR (cm^{-1}): ν 3326 (N-H), 3295 (N-H), 3282 (N-H), 1642 (C=O), 1539 (N-C=O); HRMS (ESI) m/z: $[M+H]^+$ calcd for $C_{17}H_{24}N_3O_2$: 302.1863; found 302.1850.

Propargyl hydrazide 1-Boc. It was prepared following the procedure reported in Savelson, E.; Selewski, K. A.; Tepe, J. J. A Tetrazine-Based Synthesis for Accessing Underutilized Aza-Indole Analogues. *Chem. Eur. J.* **2025**, *31*, e202500751.

General procedure for the synthesis of internal propargyl hydrazides 1a-PhOMe and 1a-PhNO$_2$. A solution of the appropriate terminal *N*-propargyl hydrazide **1** (1 mmol, 1.0 equiv) in dry THF (3 mL) was added dropwise at room temperature under argon atmosphere to a stirred mixture of the corresponding yodoarene (1 mmol, 240 mg, 1.0 equiv), bis(triphenylphosphine)palladium(II) dichloride (0.187 mmol, 144 mg, 0.187 equiv), copper(I) iodide (0.125 mmol, 19.4 mg, 0.125 equiv), and triethylamine (0.94 mL, 688 mg, 6.80 mmol, 6.80 equiv) in dry THF (9.4 mL). The reaction mixture was stirred at room temperature until completion of the reaction (monitored by TLC, tipycally 3 h). After evaporation of the solvents under reduce pressure, the residue was purified by flash chromatography on silica gel eluting with ethyl acetate/hexanes mixtures to afford the corresponding internal propargyl hydrazides. Spectroscopic and analytical data for compounds **1-Ar** follow.

Internal propargyl hydrazide 1a-PhOMe

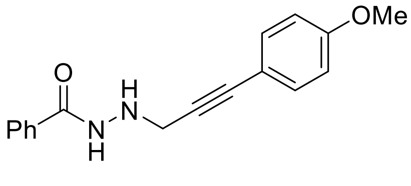

Following the general procedure and starting from 30 mg (0.17 mmol) of *N'*-(prop-2-yn-1-yl)benzohydrazide **1a**, compound **1a-PhOMe** (49%, 23 mg, 0.08 mmol) was obtained as a yellow oil, after purification by column chromatography using *n*-hexane/AcOEt (1:1) as eluent; ^{1}H NMR (300 MHz, acetone-d$_6$, 25 ºC): δ 7.79 (dd, *J* = 8.3, 1.5 Hz, 2H, CH_{Ar}), 7.43–7.27 (m, 4H, CH_{Ar}), 7.24–7.17 (m, 2H, CH_{Ar}), 6.78–6.72 (m, 2H, CH_{Ar}), 3.66 (s, 3H, CH_3), 1.15 (s, 2H, CH_2); ^{13}C {^{1}H} NMR (75 MHz, acetone-d$_6$, 25ºC): δ 205.3 (C=O), 159.7 (C_{Ar}), 132.9 (2 CH_{Ar}), 131.4 (CH_{Ar}), 129.5 (C_{Ar}),

128.4 (2 CH_{Ar}), 127.1 (2 CH_{Ar}), 125.3 (C_{Ar}), 115.2 (C), 114.0 (2 CH_{Ar}), 83.3 (C), 54.7 (CH_3), 41.3 (CH_2); IR (cm^{-1}): ν 3279 (N-H), 3221 (N-H), 1624 (C=O), 1537 (N-C=O); HRMS (ESI) m/z: $[M+H]^+$ calc. for: $C_{17}H_{17}N_2O_2$; 281.1285; found 281.1284.

Internal propargyl hydrazide 1a-PhNO₂

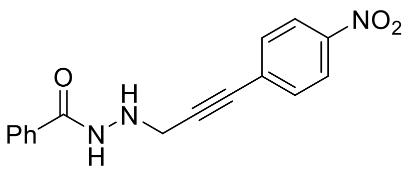

Following the general procedure and starting from 80 mg (0.46 mmol) of *N'*-(prop-2-yn-1-yl)benzohydrazide **1a**, compound **1a-PhNO₂** (81%, 110 mg, 0.37 mmol) was obtained as a yellow solid, after purification on column chromatography using DCM/methanol (0.3%) as eluent; mp 132-134 ºC; ^{1}H NMR (300 MHz, $CDCl_3$, 25 ºC): δ 8.17 (d, *J* = 9.0 Hz, 2H, 2CH_{Ar}), 7.87–7.69 (m, 3H, 2CH_{Ar}, NH), 7.60–7.52 (m, 3H, 3CH_{Ar}), 7.52–7.40 (m, 2H, 2CH_{Ar}), 5.21 (s, 1H, NH), 4.02 (s, 2H, CH_2); ^{13}C {^{1}H} NMR (101 MHz, $CDCl_3$): δ 167.7 (C=O), 165.4 (C_{Ar}), 147.2 (C_{Ar}), 132.5 (2CH_{Ar}), 132.2 (CH_{Ar}), 129.7 (C_{Ar}), 128.8 (2CH_{Ar}), 126.9 (2CH_{Ar}), 123.6 (2CH_{Ar}), 90.8 (C), 82.7 (C), 42.2 (CH_2); IR (cm^{-1}): ν 3333 (N-H), 3279 (N-H), 1627 (C=O), 1592 (N-C=O); HRMS (ESI) m/z: $[M+Na]^+$ calcd for $C_{16}H_{13}N_3NaO_3^+$: 318.0849; found 318.0847.

General procedure for the synthesis of oxadiazines 2. The appropriate alkyne **1** (1 mmol, 1 equiv) was solved in 1,4-dioxane (20 mL) at 30°C. Then, K_2CO_3 (553 mg, 4 mmol, 4 equiv) and [$Ph_3PAuNTf_2$] (37 mg, 5% mol, 0.05 mmol, 0.05 equiv) were sequentially added to the solution and stirred at 30°C. After completion of the reaction (monitored by TLC), the solvent was evaporated in vacuo. The residue was purified by flash chromatography on silica gel eluting with ethyl acetate/hexanes mixtures to afford the corresponding oxadiazines. Spectroscopic and analytical data for compounds **2** follow.

Oxadiazine 2a

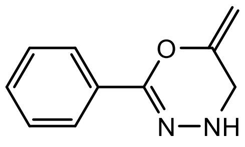

Following the general procedure, the reaction of (prop-2-yn-1-yl)benzohydrazide (**1a**) (0.07 mmol, 12 mg), K_2CO_3 (0.21 mmol, 29.4 mg), and [$Ph_3PAuNTf_2$] (5% mol, 4.2 mg) in 1,4-dioxane (1.4 mL) at 30 ºC for 1 h and after flash chromatography using *n*-hexane/AcOEt (8:2) as eluent, afforded product **2a** (96%, 11 mg, 0.069 mmol) as a yellow oil; ^{1}H NMR (300 MHz, $CDCl_3$, 25 ºC): δ 7.82 (m, 2H, CH_{Ar}), 7.37 (m, 3H, CH_{Ar}), 5.30 (br s, 1H, NH), 4.74 (d, *J* = 2.0 Hz, 1H, =CH_2), 4.27 (m, 1H, =CH_2), 3.67 (br s, 2H, CH_2); ^{13}C {^{1}H} (75 MHz, $CDCl_3$, 25ºC): δ 151.0 (O*C*=CH_2), 145.8 (C=N), 131.2 (C_{Ar}), 129.5 (CH_{Ar}), 128.2 (2 CH_{Ar}), 125.3 (2 CH_{Ar}), 89.6 (=CH_2), 43.5 (CH_2); IR (cm^{-1}): ν 2925, 1676, 1326, 1101; HRMS m/z (ESI): $[M+H]^+$ calc. for: $C_{10}H_{11}N_2O$; 175.0788; found 175.0858.

2 mmol synthetic method example: Following the general procedure and starting from 348 mg (2.0 mmol) of propargyl hydrazide **1a**, 320 mg (1.84 mmol) of compound **2a** (92%) were obtained.

Oxadiazine 2b

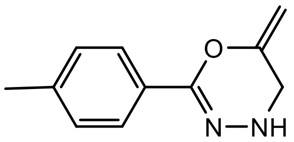

Following the general procedure, the reaction of 4-methyl-*N'*-(prop-2-yn-1-yl)benzohydrazide (**1b**) (0.05 mmol, 10 mg), K_2CO_3 (0.21 mmol, 29.4 mg) and [$Ph_3PAuNTf_2$] (5% mol, 4.2 mg) in 1,4-dioxane (1 mL) at 30 ºC for 1 h and after flash chromatography using *n*-hexane/AcOEt (8:2) as eluent, afforded product **2b** (70%, 7 mg, 0.04 mmol) as a yellow oil; ^{1}H NMR (600 MHz, $CDCl_3$, 25 ºC): δ 7.72 (d, *J* = 8.3 Hz, 2H, CH_{Ar}), 7.19 (d, *J* = 8.0 Hz, 2H, CH_{Ar}), 4.74 (d, *J* = 2.0 Hz, 1H, =CH_2), 4.27 (d, *J* = 2.0 Hz, 1H, =CH_2), 3.68 (s, 2H, CH_2), 2.38 (s, 3H, CH_3); ^{13}C {^{1}H} (151 MHz, $CDCl_3$, 25ºC): δ 153.9 (C=), 148.5 (C=N), 142.3 (C_{Ar}), 131.6 (2 CH_{Ar}), 131.1 (C_{Ar}), 128.0 (2 CH_{Ar}), 92.2 (=CH_2), 46.2 (CH_2), 24.0 (CH_3); IR (cm^{-1}): ν 2924, 2854, 1676, 1324, 1097; HRMS m/z (ESI): $[M+H]^+$ calc. for $C_{11}H_{13}N_2O$: 189.1022; found 189.1022.

Oxadiazine 2d

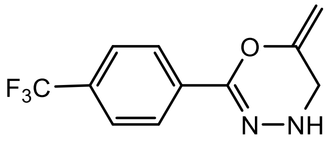

Following the general procedure, the reaction of *N'*-(prop-2-yn-1-yl)-4-(trifluoromethyl)benzohydrazide (**1d**) (0.04 mmol, 9 mg), K_2CO_3 (0.15 mmol, 20.5 mg) and [$Ph_3PAuNTf_2$] (5% mol, 3.0 mg) in 1,4-dioxane (0.8 mL) at 30 ºC for 1 h and after flash chromatography using *n*-hexane/AcOEt (8:2) as eluent, afforded product **2d** (72%, 6.5 mg, 0.03 mmol) as a yellow oil; ^{1}H NMR (600 MHz, $CDCl_3$, 25 ºC): δ 7.94 (d, J = 8.1 Hz, 2H, CH_{Ar}), 7.63 (d, J = 8.2 Hz, 2H, CH_{Ar}), 5.40 (s, 1H, NH), 4.79 (d, J = 2.1 Hz, 1H, =CH_2), 4.33 (d, J = 2.1 Hz, 1H, =CH_2), 3.72 (s, 2H, CH_2); ^{13}C {^{1}H} (151 MHz, $CDCl_3$, 25ºC): 153.3 (C=), 146.8 (C=N), 137.3 (C_{Ar}), 133.9 (CF_3), 128.2 (2 CH_{Ar}), 127.9 (2 CH_{Ar}), 125.8 (C_{Ar}), 92.9 (=CH_2), 46.0 (CH_2); IR (cm^{-1}): ν 3349, 2927, 1678, 1324, 1105; HRMS m/z (ESI): [M+H]$^+$ calc. for $C_{11}H_{10}F_3N_2O$: 243.0740; found 243.0741.

Oxadiazine 2e

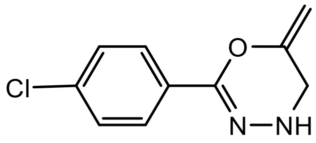

Following the general procedure, the reaction of 4-chloro-*N'*-(prop-2-yn-1-yl)benzohydrazide (**1e**) (0.05 mmol, 10 mg), K_2CO_3 (0.19 mmol, 26.5 mg) and [$Ph_3PAuNTf_2$] (5% mol, 3.8 mg) in 1,4-dioxane (1 mL) at 30 ºC for 3 h and after flash chromatography using *n*-hexane/AcOEt (8:2) as eluent, afforded product **2e** (96%, 9.6 mg, 0.05 mmol) as a yellow oil; ^{1}H NMR (600 MHz, $CDCl_3$, 25 ºC): δ 7.75 (d, J = 8.7 Hz, 2H, CH_{Ar}), 7.33 (d, J = 8.7 Hz, 2H, CH_{Ar}), 4.74 (d, J = 2.1 Hz, 1H, =CH_2), 4.29 (d, J = 2.1 Hz, 1H, =CH_2), 3.67 (s, 2H, CH_2); ^{13}C {^{1}H} (151 MHz, $CDCl_3$, 25ºC): δ 151.0 (C=), 144.9 (C=N), 135.6 (C_{Ar}), 129.9 (C_{Ar}), 128.6 (2 CH_{Ar}), 126.8 (2 CH_{Ar}), 90.1 (=CH_2), 43.5 (CH_2); IR (cm^{-1}): ν 2962, 1674, 1259, 1070; HRMS m/z (ESI): [M+H]$^+$ calc. for $C_{10}H_{10}ClN_2O$: 209.0476; found 209.0474.

Oxadiazine 2g

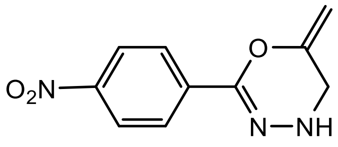

Following the general procedure, the reaction of 4-nitro-*N*'-(prop-2-yn-1-yl)benzohydrazide (**1g**) (0.05 mmol, 10 mg), K_2CO_3 (0.18 mmol, 25.2 mg) and [$Ph_3PAuNTf_2$] (5% mol, 3.7 mg) in 1,4-dioxane (1 mL) at 30 ºC for 30 min and after flash chromatography using *n*-hexane/AcOEt (8:2) as eluent, afforded product **2g** (quantitative yield, 10 mg, 0.05 mmol) as an orange solid; mp 148-150 ºC; ^{1}H NMR (600 MHz, $CDCl_3$, 25 ºC): δ 8.22 (d, *J* = 9.1 Hz, 2H, CH_{Ar}), 7.97 (d, *J* = 9.1 Hz, 2H, CH_{Ar}), 4.80 (d, *J* = 2.3 Hz, 1H, =CH_2), 4.34 (d, *J* = 2.2 Hz, 1H, =CH_2), 3.72 (s, 2H, CH_2); ^{13}C {^{1}H} (151 MHz, $CDCl_3$, 25ºC): δ 150.4 (C=), 148.3 (C=N), 143.4 (C_{Ar}), 137.3 (C_{Ar}), 126.0 (2 CH_{Ar}), 123.7 (2 CH_{Ar}), 90.8 (=CH_2), 43.4 (CH_2); IR (cm^{-1}): ν 3320, 2920, 1596, 1515, 1309, 1070; HRMS m/z (ESI): $[M-H]^-$ calc. for $C_{10}H_8N_3O_3$: 218.0571; found 218.0569.

Oxadiazine 2h

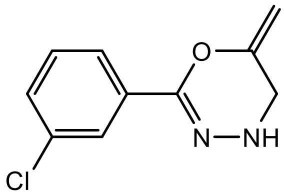

Following the general procedure, the reaction of 3-chloro-*N*'-(prop-2-yn-1-yl)benzohydrazide (**1h**) (0.05 mmol, 10 mg), K_2CO_3 (0.19 mmol, 26.5 mg) and [$Ph_3PAuNTf_2$] (5% mol, 3.8 mg) in 1,4-dioxane (1 mL) at 30 ºC for 30 min and after flash chromatography using *n*-hexane/AcOEt (8:2) as eluent, afforded product **2h** (87%, 8.7 mg, 0.04 mmol) as a yellow oil; ^{1}H NMR (600 MHz, $CDCl_3$, 25 ºC): δ 7.81 (s, 1H, CH_{Ar}), 7.70 (d, *J* = 7.8 Hz, 1H, CH_{Ar}), 7.34 (ddd, *J* = 8.0, 2.2, 1.1 Hz, 1H, CH_{Ar}), 7.29 (t, *J* = 7.8 Hz, 1H, CH_{Ar}), 4.77 (d, *J* = 2.1 Hz, 1H, =CH_2), 4.30 (d, *J* = 2.2 Hz, 1H, =CH_2), 3.68 (s, 2H, CH_2); ^{13}C {^{1}H} (151 MHz, $CDCl_3$, 25ºC): δ 150.8 (C=), 144.4 (C=N), 134.5 (C_{Ar}), 133.1

(C_{Ar}), 129.6 (2 CH_{Ar}), 125.6 (CH_{Ar}), 123.5 (CH_{Ar}), 90.3 (=CH_2), 43.5 (CH_2); IR (cm^{-1}): ν 2924, 1676, 1254, 1114; HRMS m/z (ESI): $[M+H]^+$ calc. for $C_{10}H_{10}ClN_2O$: 209.0476; found 208. 209.0477.

Oxadiazine 2i

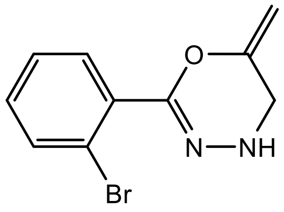

Following the general procedure, the reaction of 2-bromo-*N'*-(prop-2-yn-1-yl)benzohydrazide (**1i**) (0.08 mmol, 20 mg), K_2CO_3 (0.40 mmol, 54.6 mg) and [$Ph_3PAuNTf_2$] (5% mol, 6.2 mg) in 1,4-dioxane (1.6 mL) at 30 ºC for 45 min and after flash chromatography using *n*-hexane/AcOEt (8:2) as eluent, afforded product **2i** (quantitative yield, 20 mg, 0.4 mmol) as a yellow oil; ^{1}H NMR (600 MHz, $CDCl_3$, 25 ºC): δ 7.64 (d, *J* = 8.0 Hz, 1H, CH_{Ar}), 7.58 (dd, *J* = 7.9, 1.6 Hz, 1H, CH_{Ar}), 7.36 (t, *J* = 7.5 Hz, 1H, CH_{Ar}), 7.28 (d, *J* = 5.7 Hz, 1H, CH_{Ar}), 4.72 (d, *J* = 2.2 Hz, 1H, =CH_2), 4.30 (s, 1H, =CH_2), 3.74 (s, 2H, CH_2); ^{13}C {^{1}H} (151 MHz, $CDCl_3$, 25ºC): δ 154.1 (C=), 149.0 (C=N), 136.1 (CH_{Ar}), 135.7 (C_{Ar}), 133.5 (CH_{Ar}), 133.6 (CH_{Ar}), 129.9 (CH_{Ar}), 124.5 (C_{Ar}), 92.5 (=CH_2), 46.3 (CH_2); IR (cm^{-1}): ν 2928, 1681, 1241, 1106; HRMS m/z (ESI): $[M+H]^+$ calc. for $C_{10}H_{10}BrN_2O$: 252.9971; found 252.9957.

Oxadiazine 2j

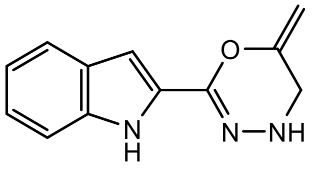

Following the general procedure, the reaction of *N'*-(prop-2-yn-1-yl)-1*H*-indole-2-carbohydrazide (**1j**) (0.07 mmol, 15 mg), K_2CO_3 (0.28 mmol, 38.9 mg) and [$Ph_3PAuNTf_2$] (5% mol, 5.5 mg) in 1,4-dioxane (1.5 mL) at 30 ºC for 4 h and after flash chromatography using *n*-hexane/AcOEt (7:3) as eluent, afforded product **2j** (52%, 7.2 mg, 0.04 mmol) as a yellow oil; ^{1}H NMR (600 MHz, $CDCl_3$, 25 ºC): δ 8.69 (s, 1H, NH), 7.62 (d, *J* = 8.0 Hz, 1H, CH_{Ar}), 7.35 (d, *J* = 8.2 Hz, 1H, CH_{Ar}), 7.22 (t, *J* = 7.4 Hz, 1H, CH_{Ar}), 7.10 (t, *J* = 7.4 Hz, 1H, CH_{Ar}), 6.89 (s, 1H, CH_{Ar}), 4.79 (d, *J* = 2.0 Hz, 1H,

$=CH_2$), 4.33 (d, J = 2.3 Hz, 1H, $=CH_2$), 3.73 (s, 2H, CH_2); ^{13}C {^1H} (151 MHz, $CDCl_3$, 25ºC): δ 150.7 (C=), 141.7 (C=N), 136.5 (C_{Ar}), 129.0 (C_{Ar}), 128.3 (C_{Ar}), 123.6 (CH_{Ar}), 121.5 (CH_{Ar}), 120.3 (CH_{Ar}), 111.1 (CH_{Ar}), 102.4 (CH_{Ar}), 90.5 ($=CH_2$), 44.0 (CH_2); IR (cm^{-1}): ν 3321, 2926, 1676, 1307, 1256; HRMS m/z (ESI): $[M-H]^-$ calc. for $C_{12}H_{10}N_3O$: 212.0829; found 212.0828.

Oxadiazine 2k

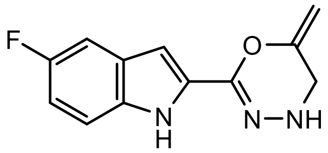

Following the general procedure, the reaction of 5-fluoro-*N'*-(prop-2-yn-1-yl)-1*H*-indole-2-carbohydrazide (**1k**) (0.06 mmol, 15 mg), K_2CO_3 (0.26 mmol, 35.9 mg) and [$Ph_3PAuNTf_2$] (5% mol, 5.2 mg) in 1,4-dioxane (1.5 mL) at 30 ºC for 1 h and after flash chromatography using *n*-hexane/AcOEt (7:3) as eluent, afforded product **2k** (73%, 11.0 mg, 0.04 mmol) as a yellow oil; ^1H NMR (600 MHz, $CDCl_3$, 25 ºC): δ 8.70 (s, 1H, NH), 7.26 (s, 1H, CH_{Ar}), 7.24 (d, *J* = 3.3 Hz, 1H, CH_{Ar}), 6.96 (td, *J* = 9.2, 2.4 Hz, 1H, CH_{Ar}), 6.83 (dd, *J* = 2.2, 0.9 Hz, 1H, CH_{Ar}), 4.78 (d, *J* = 2.2 Hz, 1H, $=CH_2$), 4.32 (d, *J* = 2.2 Hz, 1H, $=CH_2$), 3.71 (s, 2H, CH_2); ^{13}C {^1H} (151 MHz, $CDCl_3$, 25ºC): δ 158.2 (d, *J* = 235.2 Hz, C_{Ar-F}), 150.6 (C_{Ar}), 141.4 (C=), 133.1 (C=N), 130.7 (C_{Ar}), 128.6 (C_{Ar}), 112.1 (d, *J* = 26.8 Hz, CH_{Ar}), 111.8 (d, *J* = 9.5 Hz, CH_{Ar}), 106.1 (d, *J* = 23.6 Hz, CH_{Ar}), 102.4 (CH_{Ar}), 90.7 ($=CH_2$), 43.9 (CH_2); IR (cm^{-1}): ν 3341, 2925, 1700, 1526, 1261; HRMS m/z (ESI): $[M-H]^-$ calc. for $C_{12}H_9FN_3O$: 230.0735; found 230.0734.

Oxadiazine 2l

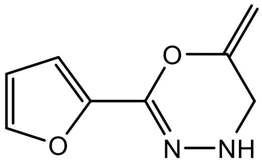

Following the general procedure, the reaction of *N'*-(prop-2-yn-1-yl)furan-2-carbohydrazide (**1l**) (0.09 mmol,15 mg), K_2CO_3 (0.37 mmol, 50.5 mg) and [$Ph_3PAuNTf_2$] (5% mol, 7.2 mg) in 1,4-

dioxane (1.5 mL) at 30 ºC for 1.5 h and after flash chromatography using *n*-hexane/AcOEt (8:2) as eluent, afforded product **2l** (68%, 10.2 mg, 0.6 mmol) as a yellow oil; ^{1}H NMR (600 MHz, $CDCl_3$, 25 ºC): δ 7.45 (dd, *J* = 1.8, 0.9 Hz, 1H, CH_{Ar}), 6.72 (dd, *J* = 3.4, 0.9 Hz, 1H, CH_{Ar}), 6.44 (dd, *J* = 3.4, 1.8 Hz, 1H, CH_{Ar}), 4.73 (d, *J* = 2.2 Hz, 1H, $=CH_2$), 4.28 (d, *J* = 2.2 Hz, 1H, $=CH_2$), 3.68 (s, 2H, CH_2); ^{13}C {^{1}H} (151 MHz, $CDCl_3$, 25ºC): δ 150.7 (C=), 145.3 (C=N), 143.8 (CH_{Ar}), 140.1 (C_{Ar}), 111.3 (CH_{Ar}), 109.9 (CH_{Ar}), 90.5 ($=CH_2$), 43.8 (CH_2); IR (cm^{-1}): ν 2926, 1678, 1259, 1171, 1112; HRMS m/z (ESI): $[M+H]^+$ calc. for $C_8H_9N_2O_2$: 165.0659; found 165.0660.

Oxadiazine 2m

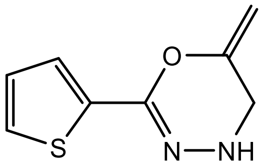

Following the general procedure, the reaction of *N'*-(prop-2-yn-1-yl)thiophene-2-carbohydrazide (**1m**) (0.08 mmol, 15 mg), K_2CO_3 (0.42 mmol, 57.5 mg) and [$Ph_3PAuNTf_2$] (5% mol, 6.5 mg) in 1,4-dioxane (1.5 mL) at 30 ºC for 2 h and after flash chromatography using *n*-hexane/AcOEt (8:2) as eluent, afforded product **2m** (71%, 10.7 mg, 0.06 mmol) as a yellow oil; ^{1}H NMR (600 MHz, $CDCl_3$, 25 ºC): δ 7.41 (dd, *J* = 3.6, 1.3 Hz, 1H, CH_{Ar}), 7.29 (dd, *J* = 5.0, 1.3 Hz, 1H, CH_{Ar}), 7.01 (dd, *J* = 5.0, 3.6 Hz, 1H, CH_{Ar}), 4.74 (d, *J* = 2.2 Hz, 1H, $=CH_2$), 4.28 (d, *J* = 2.2 Hz, 1H, $=CH_2$), 3.68 (s, 2H, CH_2); ^{13}C {^{1}H} (151 MHz, $CDCl_3$, 25ºC): δ 150.9 (C=), 143.3 (C=N), 134.8 (C_{Ar}), 127.3 (CH_{Ar}), 127.0 (CH_{Ar}), 126.1 (CH_{Ar}), 90.2 ($=CH_2$), 43.7 (CH_2); IR (cm^{-1}): ν 2827, 1674, 1438, 1365, 1259; HRMS m/z (ESI): $[M+H]^+$ calc. for $C_8H_9N_2OS$: 181.0430; found 181.0432.

Oxadiazine 2n

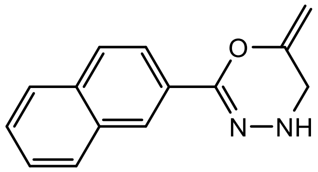

Following the general procedure and starting from 10 mg (0.05 mmol) of propargyl hydrazide **1n-N**, 6 mg (0.02 mmol) of compound **2n-M** (59%) were obtained as a green oil, after purification by column chromatography using *n*-hexane/AcOEt (9:1 → 8:1) as eluent; ^{1}H NMR (700 MHz, $CDCl_3$) δ 8.29 (s, 1H, CH_{Ar}), 7.94 (dd, *J* = 8.6, 1.7 Hz, 1H, CH_{Ar}), 7.92–7.88 (m, 1H, CH_{Ar}), 7.86–7.81 (m, 2H, 2CH_{Ar}), 7.52–7.47 (m, 2H, CH_{Ar}), 5.38 (s, 1H, NH), 4.83 (d, *J* = 2.0 Hz, 1H, C*H*H), 4.33 (d, *J* = 2.1 Hz, 1H, CH*H*), 3.74 (s, 2H, CH_2); ^{13}C NMR (176 MHz, $CDCl_3$) δ 151.1 (C), 145.6 (C_{Ar}), 133.8 (C_{Ar}), 132.9 (C_{Ar}), 128.6 (CH_{Ar}), 128.5 (C_{Ar}), 127.9 (CH_{Ar}), 127.7 (CH_{Ar}), 126.7 (CH_{Ar}), 126.3 (CH_{Ar}), 124.8 (CH_{Ar}), 122.8 (CH_{Ar}), 89.8 (CH_2), 43.6 (CH_2); IR (cm^{-1}): ν 3346 (N-H), 1674 (N=C-O) ; HRMS (ESI) m/z: $[M+H]^+$ calcd for $C_{14}H_{13}N_2O^+$: 225.1022; found 225.1022.

Oxadiazine 2n-N

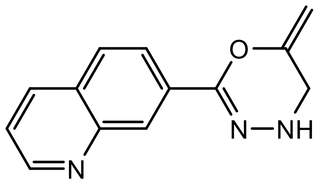

Following the general procedure, the reaction of *N*'-(prop-2-yn-1-yl)quinoline-7-carbohydrazide (**1n-N**) (0.04 mmol, 10 mg), K_2CO_3 (0.18 mmol, 24.5 mg) and [$Ph_3PAuNTf_2$] (5% mol, 3.5 mg) in 1,4-dioxane (0.8 mL) at 30 ºC for 2 h and after flash chromatography using *n*-hexane/AcOEt (4:6) as eluent, afforded product **2n-N** (75%, 7.5 mg, 0.03 mmol) as a yellow oil; ^{1}H NMR (600 MHz, $CDCl_3$, 25 ºC): δ 8.94 (dd, *J* = 4.2, 1.9 Hz, 1H, CH_{Ar}), 8.54 (s, 1H), 8.15 (d, *J* = 8.1 Hz, 1H, CH_{Ar}), 8.03 (dd, *J* = 8.6, 1.4 Hz, 1H, CH_{Ar}), 7.79 (d, *J* = 8.6 Hz, 1H, CH_{Ar}), 7.41 (dd, *J* = 8.2, 4.2 Hz, 1H, CH_{Ar}), 4.81 (d, *J* = 2.1 Hz, 1H, =CH_2), 4.32 (d, *J* = 2.0 Hz, 1H, =CH_2), 3.74 (s, 2H, CH_2); ^{13}C {^{1}H} (151 MHz, $CDCl_3$, 25ºC): δ 151.0 (CH_{Ar}), 150.9 (C=), 148.2 (C=N), 145.1 (C_{Ar}), 136.0 (CH_{Ar}), 132.5 (C_{Ar}), 128.9 (C_{Ar}), 127.8 (CH_{Ar}), 126.1 (CH_{Ar}), 123.8 (CH_{Ar}), 121.7 (CH_{Ar}), 90.2 (=CH_2), 43.6 (CH_2); IR (cm^{-1}): ν 2926, 1675, 1261, 1188; HRMS m/z (ESI): $[M+H]^+$ calc. for $C_{13}H_{12}N_3O$: 226.0975; found 226.0978.

AgNPs were prepared according to a literature (Luna, A.; Herrera, F.; Higuera, S.; Murillo, A.; Fernández, I.; Almendros, P. *J. Catal.* 2020, *389*, 432–439) procedure: 1 weight% $AgNO_3$•SiO_2 was prepared by adding $AgNO_3$ (100 mg, 0.59 mmol, 0.59 equiv) to a slurry of Scharlau silica gel 60 (9.90 g, 164 mmol, 164 equiv, 0.04-0.06 mm, 230-400 mesh) in deionized water (27 ml). The mixture was stirred for 15 minutes, concentrated under vacuum at 60 ºC to form a free-flowing powder and dried by heating to 140 ºC under high vacuum for 6-7 hours. Nanoparticles were kept in the dark at room temperature under argon atmosphere and can be stored for 1.5-month period.

General procedure for the synthesis of pyrazoles 3. The appropriate alkyne **1** (1 mmol, 1 equiv) was solved in 1,4-dioxane (20 mL) in an oven-dried microwave vessel. Then, AgNPs supported on silica gel (1 weight% $AgNO_3$•SiO_2, 0.0059 mmol, 0.0059 equiv) were added to the solution portionwise. Next, the reaction mixture was heated under microwave irradiation at 100 ºC. After completion of the reaction (monitored by TLC), the solvent was evaporated, and the residue was purified by flash chromatography using silica gel and *n*-hexane/AcOEt mixtures as eluent, to afford the corresponding pyrazoles. Spectroscopic and analytical data for compounds **3** follow.

Pyrazole 3a

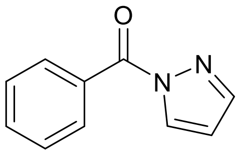

Following the general procedure, the reaction of (prop-2-yn-1-yl)benzohydrazide (**1a**) (0.05 mmol, 10 mg) and AgNPs supported on silica gel (1% Ag) in 1,4-dioxane (1 mL) at 100 ºC for 2 h in the microwave and after flash chromatography using *n*-hexane/AcOEt (9:1) as eluent, afforded product **3a** (92%, 8.6 mg, 0.046 mmol) as a pale yellow oil; ^{1}H NMR (300 MHz, $CDCl_3$, 25 ºC): δ 8.38 (dd, *J* = 4.8, 2.9 Hz, 1H, CH), 8.06 (m, 2H, CHAr), 7.73 (br s, 1H, CH), 7.58–7.52 (m, 1H, CHAr), 7.47–7.41 (m, 2H, CH), 6.46 (dd, J = 2.9, 1.5 Hz, 1H, CHAr); ^{13}C {1H} (75 MHz, $CDCl_3$, 25ºC): δ 166.7 (C=O), 144.5 (CH), 133.0 (CAr), 131.5 (2 CHAr), 130.5 (CAr), 128.1 (2 CHAr), 109.5 (CH); IR (cm^{-1}): ν 2921, 2854, 1672 (C=O), 1284; HRMS m/z (ESI): [M+Na]$^+$ calc. for $C_{10}H_8N_2NaO$: 195.0529;

found 195.0522; **3a** has ben previously reported using a different procedure (Alam, T.; Rakshit, A.; Dhara, H. N.; Palai, A.; Patel, B. K. Electrochemical Amidation: Benzoyl Hydrazine/Carbazate and Amine as Coupling Partners. *Org. Lett.* **2022**, *24*, 6619–6624).

Pyrazole 3b

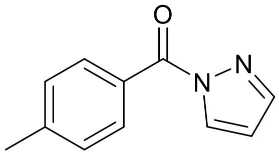

Following the general procedure, the reaction of 4-methyl-*N'*-(prop-2-yn-1-yl)benzohydrazide (**1b**) (0.05 mmol, 10 mg) and AgNPs supported on silica gel (1% Ag) in 1,4-dioxane (1 mL) at 100 ºC for 2 h in the microwave and after flash chromatography using *n*-hexane/AcOEt (9:1) as eluent, afforded product **3b** (66%, 6.6 mg, 0.03 mmol) as a pale yellow oil; ^{1}H NMR (600 MHz, $CDCl_3$, 25 ºC): δ 8.43 (d, J = 2.8 Hz, 1H, CH), 8.04 (d, J = 8.3 Hz, 2H, CH_{Ar}), 7.80 (s, 1H, CH), 7.31 (d, J = 8.0 Hz, 2H, CH_{Ar}), 6.52 (d, J = 1.3 Hz, 1H, CH), 2.44 (s, 3H, CH_3); ^{13}C {^{1}H} (151 MHz, $CDCl_3$, 25ºC): δ 166.5 (C=O), 144.5 (CH), 144.1 (C_{Ar}), 131.8 (2 CH_{Ar}), 130.6 (C_{Ar}), 129.0 (2 CH_{Ar}), 128.8 (CH), 109.4 (CH), 21.9 (CH_3); IR (cm^{-1}): ν 2923, 2853, 1670 (C=O), 1283; HRMS m/z (ESI): $[M+Na]^+$ calc. for $C_{11}H_{10}N_2NaO$: 209.0685; found 209.0684. **3b** has ben previously reported using a different procedure (Boudry, E.; Bourdreux, F.; Marrot, J.; Moreau, X.; Ghiazza, C. Dearomatization of Pyridines: Photochemical Skeletal Enlargement for the Synthesis of 1,2-Diazepines. *J. Am. Chem. Soc.* **2024**, *146*, 2845–2854).

Pyrazole 3c

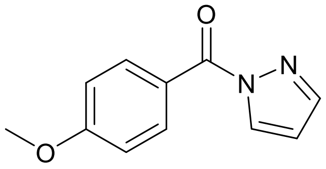

Following the general procedure, the reaction of 4-methoxy-*N'*-(prop-2-yn-1-yl)benzohydrazide (**1c**) (0.05 mmol, 10 mg) and AgNPs supported on silica gel (1% Ag) in 1,4-dioxane (1 mL) at 100 ºC for 3 h in the microwave and after flash chromatography using *n*-hexane/AcOEt (9:1) as eluent, afforded

product **3c** (75%, 7.5 mg, 0.04 mmol) as a colorless oil; ^{1}H NMR (600 MHz, acetone-d$_6$, 25ºC): δ 8.49 (dd, *J* = 2.8, 0.8 Hz, 1H, CH), 8.32–8.24 (m, 2H, CH$_{Ar}$), 7.87 (s, 1H, CH), 7.18–6.99 (m, 2H, CH$_{Ar}$), 6.63 (dd, *J* = 2.8, 1.5 Hz, 1H, CH), 3.94 (s, 3H, CH$_3$); ^{13}C {^{1}H} (151 MHz, acetone-d$_6$, 25ºC): δ 165.7 (C=O), 164.7 (C$_{Ar}$), 144.9 (CH), 135.2 (2 CH$_{Ar}$), 131.3 (CH), 124.4 (C$_{Ar}$), 114.3 (2 CH$_{Ar}$), 109.9 (CH), 56.0 (CH$_3$); IR (cm^{-1}): ν 2925, 2855, 1672 (C=O), 1285; HRMS m/z (ESI): [M+H]$^+$ calc. for $C_{11}H_{11}N_2O_2$: 203.0815; found 203.0818. **3c** has ben previously reported using a different procedure (Nandi, J.; Ovian, J. M.; Kelly, C. B.; Leadbeater, N. E. Oxidative Functionalisation of Alcohols and Aldehydes via the Merger of Oxoammonium Cations and Photoredox Catalysis. *Org. Biomol. Chem.* **2017**, *15*, 8295–8301).

Pyrazole 3-N

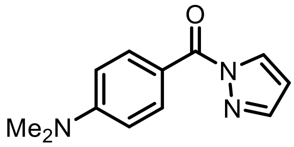

Following the general procedure and starting from 20 mg (0.09 mmol) of propargyl hydrazide **1-N**, 15.6 mg (0.07 mmol) of compound **3-N** (79%) were obtained as a brown oil, after purification on column chromatography using *n*-hexane/AcOEt (10:1) as eluent; ^{1}H NMR (300 MHz, CDCl$_3$, 25 ºC): δ 8.42 (dd, *J* = 2.8, 0.8 Hz, 1H, CH$_{Ar}$), 8.22 (d, *J* = 9.3 Hz, 2H, 2CH$_{Ar}$), 7.78 (dd, *J* = 1.5, 0.8 Hz, 1H, CH$_{Ar}$), 6.72 (d, *J* = 9.2 Hz, 2H, 2CH$_{Ar}$), 6.48 (dd, *J* = 2.8, 1.5 Hz, 1H, CH$_{Ar}$), 3.09 (s, 6H, 2CH$_3$); ^{13}C NMR {^{1}H} (75 MHz, CDCl$_3$, 25ºC): δ 165.4 (CO), 153.6 (C$_{Ar}$), 143.4 (CH$_{Ar}$), 134.3 (2CH$_{Ar}$), 130.5(CH$_{Ar}$), 117.4 (C$_{Ar}$), 110.5 (2CH$_{Ar}$), 108.3 (CH$_{Ar}$), 40.0 (2CH$_3$); IR (cm^{-1}): ν 1668 (C=O), 1599 (N-C=O); HRMS m/z (ESI): [M+Na]$^+$ calc. for $C_{12}H_{13}N_3NaO$: 238.0951; found 238.0953.

Pyrazole 3d

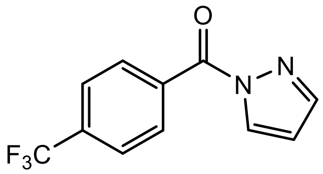

Following the general procedure, the reaction of *N'*-(prop-2-yn-1-yl)-4-(trifluoromethyl)benzohydrazide (**1d**) (0.04 mmol, 9 mg) and AgNPs supported on silica gel (1% Ag) in 1,4-dioxane (0.8 mL) at 100 ºC for 3.5 h in the microwave and after flash chromatography using *n*-hexane/AcOEt (95:5) as eluent, afforded product **3d** (37%, 3.7 mg, 0.01 mmol) as a colourless oil; ^{1}H NMR (600 MHz, acetone-d_6, 25ºC): δ 8.56 (dd, *J* = 3.0, 0.8 Hz, 1H, CH), 8.32 (dt, *J* = 8.1, 0.8 Hz, 2H, CH_{Ar}), 8.04–7.85 (m, 3H, $CH_{Ar,}$ CH), 6.71 (dd, *J* = 2.9, 1.5 Hz, 1H, CH); ^{13}C {^{1}H} (151 MHz, acetone-d_6, 25ºC): δ 167.8 (C=O), 147.6 (CH), 138.3 (C_{Ar}), 135.3 (C_{Ar}), 135.8 (CF_3), 134.7 (2 CH_{Ar}), 132.9 (CH), 127.5 (2 CH_{Ar}), 112.8 (CH); IR (cm^{-1}): ν 2921, 2852, 1668 (C=O), 1280; HRMS m/z (ESI): $[M+H]^+$ calc. for $C_{11}H_8F_3N_2O$: 241.0583; found 241.0589. **3d** has ben previously reported using a different procedure (Nandi, J.; Ovian, J. M.; Kelly, C. B.; Leadbeater, N. E. Oxidative Functionalisation of Alcohols and Aldehydes via the Merger of Oxoammonium Cations and Photoredox Catalysis. *Org. Biomol. Chem.* **2017**, *15*, 8295–8301).

Pyrazole 3e

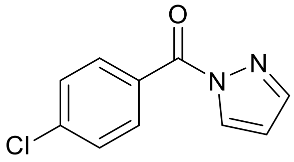

Following the general procedure, the reaction of 4-chloro-*N'*-(prop-2-yn-1-yl)benzohydrazide (**1e**) (0.03 mmol, 5.8 mg) and AgNPs supported on silica gel (1% Ag) in 1,4-dioxane (0.6 mL) at 100 ºC for 2.5 h in the microwave and after flash chromatography using *n*-hexane/AcOEt (9:1) as eluent, afforded product **3e** (69%, 4 mg, 0.02 mmol) as a pale yellow oil; ^{1}H NMR (600 MHz, $CDCl_3$, 25 ºC): δ 8.44 (dd, *J* = 2.8, 0.8 Hz, 1H, CH), 8.15–8.10 (m, 2H, CH_{Ar}), 7.80 (d, *J* = 0.8 Hz, 1H, CH), 7.52–7.46 (m, 2H, CH_{Ar}), 6.54 (dd, *J* = 2.8, 1.5 Hz, 1H, CH); ^{13}C {^{1}H} (151 MHz, $CDCl_3$, 25ºC): δ 168.0 (C=O), 147.4 (CH), 142.3 (CH), 135.7 (2 CH_{Ar}), 133.1 (C_{Ar}), 132.4 (C_{Ar}), 131.2 (2 CH_{Ar}), 112.4 (CH); IR (cm^{-1}): ν 2928, 2855, 1670 (C=O), 1286; HRMS m/z (ESI): $[M+H]^+$ calc. for $C_{10}H_8ClN_2O$: 207.0320; found 207.0312. **3e** has ben previously reported using a different procedure

(Gong, N.; Zhao, Z.; James Young, D.; Cao, X.; Ren, Z. G.; Li, H. X. Catalyst-Free Photooxidative *N*-Acylation of Azoles with Aldehydes. *Chem. Eur. J.* **2025**, *31*, e202404225).

Pyrazole 3f

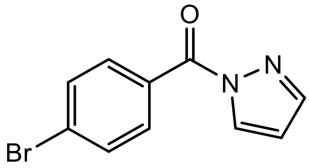

Following the general procedure, the reaction of 4-bromo-*N'*-(prop-2-yn-1-yl)benzohydrazide (**1f**) (0.04 mmol, 10 mg) and AgNPs supported on silica gel (1% Ag) in 1,4-dioxane (0.8 mL) at 100 ºC for 3.5 h in the microwave and after flash chromatography using *n*-hexane/AcOEt (9:1) as eluent, afforded product **3f** (82%, 8.2 mg) as a pale yellow oil; ^{1}H NMR (600 MHz, $CDCl_3$, 25 ºC): δ 8.44 (dd, *J* = 2.9, 0.7 Hz, 1H, CH), 8.07–8.01 (m, 2H, CH_{Ar}), 7.80 (dd, *J* = 1.4, 0.8 Hz, 1H, CH), 7.70–7.63 (m, 2H, CH_{Ar}), 6.54 (dd, *J* = 2.8, 1.5 Hz, 1H, CH); ^{13}C {^{1}H} (151 MHz, $CDCl_3$, 25ºC): δ 165.6 (C=O), 144.8 (CH), 133.3 (2 CH_{Ar}), 131.6 (2 CH_{Ar}), 130.6 (CH), 130.4 (C_{Ar}), 128.5 (C_{Ar}), 109.8 (CH); IR (cm^{-1}): ν 2930, 2856, 1671 (C=O), 1284; HRMS m/z (ESI): [M+H]$^{+}$ calc. for $C_{10}H_8BrN_2O$: 250.9815; found 250.9822. **3f** has ben previously reported using a different procedure (Boudry, E.; Bourdreux, F.; Marrot, J.; Moreau, X.; Ghiazza, C. Dearomatization of Pyridines: Photochemical Skeletal Enlargement for the Synthesis of 1,2-Diazepines. *J. Am. Chem. Soc.* **2024**, *146*, 2845–2854).

Pyrazole 3h

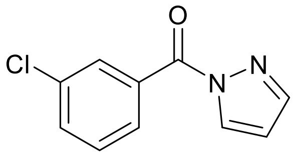

Following the general procedure, the reaction of 3-chloro-*N'*-(prop-2-yn-1-yl)benzohydrazide (**1h**) (0.05 mmol, 10 mg) and AgNPs supported on silica gel (1% Ag) in 1,4-dioxane (1 mL) at 100 ºC for 2.5 h in the microwave and after flash chromatography using *n*-hexane/AcOEt (9:1) as eluent, afforded product **3h** (20%, 2 mg, 0.01 mmol) as a pale yellow oil; ^{1}H NMR (600 MHz, $CDCl_3$, 25

ºC): δ 8.44 (dd, J = 2.9, 1.0 Hz, 1H, CH), 8.24–8.10 (m, 1H, CH_{Ar}), 8.03 (dt, J = 7.8, 1.4 Hz, 1H, CH), 7.82 (s, 1H, CH), 7.59 (ddd, J = 8.0, 2.1, 1.1 Hz, 1H, CH_{Ar}), 7.45 (t, J = 7.9 Hz, 1H, CH_{Ar}), 6.55 (tq, J = 2.8, 1.3 Hz, 1H, CH); ^{13}C {^{1}H} (151 MHz, $CDCl_3$, 25ºC): δ 165.2 (C=O), 145.0 (CH), 134.4 (C_{Ar}), 133.3 (C_{Ar}), 133.2 (CH_{Ar}), 131.7 (CH_{Ar}), 130.6 (CH_{Ar}), 129.8 (CH_{Ar}), 129.5 (CH), 110.0 (CH); IR (cm^{-1}): ν 2931, 2857, 1673 (C=O), 1285; HRMS m/z (ESI): $[M+H]^+$ calc. for $C_{10}H_8ClN_2O$: 207.0320; found 207.0325. **3h** has ben previously reported using a different procedure (Gong, N.; Zhao, Z.; James Young, D.; Cao, X.; Ren, Z. G.; Li, H. X. Catalyst-Free Photooxidative N-Acylation of Azoles with Aldehydes. *Chem. Eur. J.* **2025**, *31*, e202404225).

Pyrazole 3i

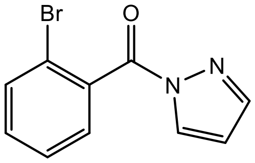

Following the general procedure, the reaction of 2-bromo-*N'*-(prop-2-yn-1-yl)benzohydrazide (**1i**) (0.04 mmol, 10 mg) and AgNPs supported on silica gel (1% Ag) in 1,4-dioxane (0.8 mL) at 100 ºC for 2.5 h in the microwave and after flash chromatography using *n*-hexane/AcOEt (9:1) as eluent, afforded product **3i** (36%, 3.6 mg, 0.01 mmol) as a colorless oil; ^{1}H NMR (600 MHz, $CDCl_3$, 25 ºC): δ 8.39 (d, J = 2.9 Hz, 1H, CH), 7.79–7.75 (m, 1H, CH), 7.70–7.65 (m, 1H, CH_{Ar}), 7.50 (dd, J = 7.6, 1.8 Hz, 1H, CH_{Ar}), 7.48–7.43 (m, 1H, CH_{Ar}), 7.40 (td, J = 7.7, 1.8 Hz, 1H, CH_{Ar}), 6.55 (dd, J = 2.9, 1.5 Hz, 1H, CH); ^{13}C {^{1}H} (151 MHz, $CDCl_3$, 25ºC): δ 166.4 (C=O), 145.4 (CH), 135.5 (C_{Ar}), 133.2 (CH_{Ar}), 132.1 (CH_{Ar}), 129.7 (CH_{Ar}), 129.5 (CH_{Ar}), 127.2 (CH), 120.4 (C_{Ar}), 110.6 (CH); IR (cm^{-1}): ν 2925, 1712 (C=O), 1380, 1028; HRMS m/z (ESI): $[M+H]^+$ calc. for $C_{10}H_8BrN_2O$: 250.9815; found 250.9809. **3i** has ben previously reported using a different procedure (Nandi, J.; Ovian, J. M.; Kelly, C. B.; Leadbeater, N. E. Oxidative Functionalisation of Alcohols and Aldehydes via the Merger of Oxoammonium Cations and Photoredox Catalysis. *Org. Biomol. Chem.* **2017**, *15*, 8295–8301).

Pyrazole 3j

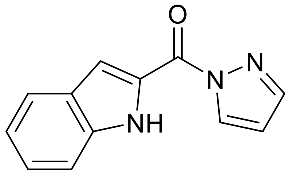

Following the general procedure, the reaction of *N'*-(prop-2-yn-1-yl)-1*H*-indole-2-carbohydrazide (**1j**) (0.05 mmol, 10 mg) and AgNPs supported on silica gel (1% Ag) in 1,4-dioxane (1 mL) at 100 ºC for 2.5 h in the microwave and after flash chromatography 1% Ag using *n*-hexane/AcOEt (9:1) as eluent, afforded product **3j** (quantitative yield, 10 mg, 0.05 mmol) as a colorless oil; ^{1}H NMR (600 MHz, $CDCl_3$, 25 ºC): δ 10.92 (s, 1H, NH), 8.51 (dd, *J* = 2.8, 0.8 Hz, 1H, CH), 7.88 (ddd, *J* = 15.9, 1.9, 1.0 Hz, 2H, 2CH), 7.76 (dd, *J* = 8.1, 1.0 Hz, 1H, CH_{Ar}), 7.50 (dd, *J* = 8.3, 1.0 Hz, 1H, CH_{Ar}), 7.38 (ddd, *J* = 8.2, 6.9, 1.2 Hz, 1H, CH_{Ar}), 7.18 (ddd, *J* = 8.0, 6.9, 1.0 Hz, 1H, CH_{Ar}), 6.55 (dd, *J* = 2.8, 1.5 Hz, 1H, CH_{Ar}); ^{13}C {^{1}H} (151 MHz, $CDCl_3$, 25ºC): δ 157.0 (C=O), 144.5 (CH), 138.0 (C_{Ar}), 130.5 (CH), 128.0 (C_{Ar}), 127.1 (C_{Ar}), 126.7 (CH_{Ar}), 123.2 (CH_{Ar}), 121.2 (CH_{Ar}), 115.4 (CH), 112.6 (CH_{Ar}), 109.2 (CH_{Ar}); IR (cm^{-1}): ν 3348, 2926, 1681 (C=O), 1518, 1308; HRMS m/z (ESI): $[M-H]^-$ calc. for $C_{12}H_8N_3O$: 210.0673; found 210.0673. **3j** has ben previously reported using a different procedure (Hou, L.; Yang, L.; Yang, G.; Luo, Z.; Xiao, W.; Yang, L.; Wang, F.; Gong, L. Z.; Liu, X.; Cao, W.; *et al.* Catalytic Asymmetric Dearomative [2+2] Photocycloaddition/Ring-Expansion Sequence of Indoles with Diversified Alkenes. *J. Am. Chem. Soc.* **2024**, *146*, 23457–23466).

Pyrazole 3k

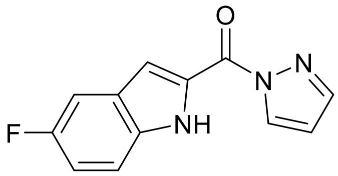

Following the general procedure, the reaction of 5-fluoro-*N'*-(prop-2-yn-1-yl)-1*H*-indole-2-carbohydrazide (**1k**) (0.06 mmol, 15 mg) and AgNPs supported on silica gel (1% Ag) in 1,4-dioxane (1.5 mL) at 100 ºC for 2.5 h in the microwave and after flash chromatography using *n*-hexane/AcOEt (9:1) as eluent, afforded product **3k** (27%, 4 mg, 0.02 mmol) as a pale yellow oil; ^{1}H NMR (600

MHz, $CDCl_3$, 25 ºC): δ 11.0 (s, 1H, NH), 8.51 (dd, *J* = 2.9, 0.7 Hz, 1H, CH), 7.89 (dd, *J* = 1.5, 0.6 Hz, 1H, CH), 7.80 (dd, *J* = 2.2, 1.0 Hz, 1H, CH), 7.44 (ddt, *J* = 9.0, 4.4, 0.8 Hz, 1H, CH_{Ar}), 7.38 (dd, *J* = 9.1, 2.4 Hz, 1H, CH_{Ar}), 7.15 (td, *J* = 9.1, 2.6 Hz, 1H, CH_{Ar}), 6.55 (dd, *J* = 2.8, 1.5 Hz, 1H, CH_{Ar}); ^{13}C {^1H} (151 MHz, $CDCl_3$, 25ºC): δ 159.2 (C=O), 156.7 (C_{Ar}), 144.6 (CH), 134.6 (C_{Ar}), 130.6 (CH), 129.5 (C_{Ar}), 127.1 (C_{Ar}), 116.1 (d, *J* = 27.2 Hz) (CH_{Ar}), 114.9 (CH), 113.7 (d, *J* = 9.5 Hz) (CH_{Ar}), 109.3 (CH_{Ar}), 107.0 (d, *J* = 23.6 Hz) (CH_{Ar}); IR (cm^{-1}): ν 3341, 2925, 1700 (C=O), 1526, 1261; HRMS m/z (ESI): $[M-H]^-$ calc. for $C_{12}H_7FN_3O$: 228.0579; found 228.0579. **3k** has ben previously reported using a different procedure (Ma, J.; Schäfers, F.; Daniliuc, C.; Bergander, K.; Strassert, C. A.; Glorius, F. Gadolinium Photocatalysis: Dearomative [2+2] Cycloaddition/Ring-Expansion Sequence with Indoles. *Angew. Chem. Int. Ed.* **2020**, *59*, 9639–9645).

Pyrazole 3l

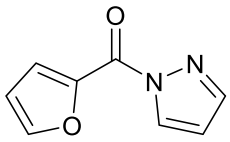

Following the general procedure, the reaction of *N'*-(prop-2-yn-1-yl)furan-2-carbohydrazide (**1l**) (0.06 mmol, 10 mg) and AgNPs supported on silica gel (1% Ag) in 1,4-dioxane (1.2 mL) at 100 ºC for 3.5 h in the microwave and after flash chromatography using *n*-hexane/AcOEt (9:1) as eluent, afforded product **3l** (70%, 7 mg, 0.4 mmol) as a colorless oil; ^1H-NMR (300 MHz, $CDCl_3$): δ 8.43 (dd, *J* = 2.9, 0.7 Hz, 1H), 8.08 (dd, *J* = 3.7, 0.8 Hz, 1H), 7.78 (ddd, *J* = 6.9, 1.6, 0.8 Hz, 2H), 6.63 (dd, *J* = 3.6, 1.7 Hz, 1H), 6.49 (dd, *J* = 2.9, 1.5 Hz, 1H); ^{13}C {^1H} (75 MHz, $CDCl_3$, 25ºC): δ 154.7 (C=O), 148.3 (CH), 144.7 (C_{Ar}), 144.6 (CH_{Ar}), 130.0 (CH_{Ar}), 124.8 (CH_{Ar}), 112.6 (CHAr), 109.3 (CHAr); IR (cm^{-1}): ν 3090, 1688 (C=O), 924; HRMS m/z (ESI): $[M+Na]^+$ calc. for $C_8H_6N_2NaO_2$: 185.0321; found 185.0316; **3l** has ben previously reported using a different procedure (Ovian, J. M.; Kelly, C. B.; Pistritto, V. A.; Leadbeater, N. E. Accessing *N*-Acyl Azoles via Oxoammonium Salt-Mediated Oxidative Amidation. *Org. Lett.* **2017**, *19*, 1286–1289).

Pyrazole 3m

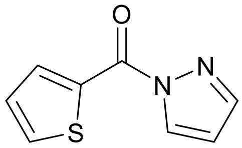

Following the general procedure, the reaction of *N'*-(prop-2-yn-1-yl)thiophene-2-carbohydrazide (**1m**) (0.08 mmol, 15 mg) and AgNPs supported on silica gel (1% Ag) in 1,4-dioxane (1.6 mL) at 100 ºC for 3.5 h in the microwave and after flash chromatography using *n*-hexane/AcOEt (9:1) as eluent, afforded product **3m** (47%, 7 mg, 0.04 mmol) as a colorless oil; ^{1}H NMR (600 MHz, $CDCl_3$, 25 ºC): δ 8.45 (dd, *J* = 3.9, 1.3 Hz, 1H, CH_{Ar}), 8.43 (dd, *J* = 2.8, 0.7 Hz, 1H, CH), 7.82 (d, *J* = 0.7 Hz, 1H, CH), 7.80 (dd, *J* = 5.0, 1.3 Hz, 1H, CH_{Ar}), 7.20 (dd, *J* = 5.0, 3.9 Hz, 1H, CH_{Ar}), 6.51 (dd, *J* = 2.8, 1.5 Hz, 1H, CH); ^{13}C {^{1}H} (151 MHz, $CDCl_3$, 25ºC): δ 159.0 (C=O), 144.2 (CH), 138.7 (CH_{Ar}), 137.5 (CH_{Ar}), 132.6 (C_{Ar}), 129.9 (CH_{Ar}), 127.6 (CH), 109.8 (CH); IR (cm^{-1}): ν 3105, 1683 (C=O), 875; HRMS m/z (ESI): $[M+Na]^+$ calc. for $C_8H_6N_2NaOS$: 201.0093; found 201.0100. **3m** has ben previously reported using a different procedure (Wang, X.; Gao, S.; Yang, J.; Gao, Y.; Wang, L.; Tang, X. Synthesis and Antifungal Activity Evaluation of New Heterocycle Containing Amide Derivatives. *Nat. Prod. Res.* **2016**, *30*, 682–688).

Pyrazole 3n

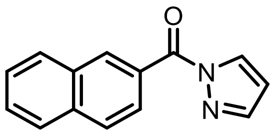

Following the general procedure and starting from 20 mg (0.09 mmol) of propargyl hydrazide **1n**, 12.9 mg (0.06 mmol) of compound **3n** (65%) were obtained as a green oil, after purification by column chromatography using *n*-hexane/AcOEt (10:1) as eluent; ^{1}H NMR (300 MHz, $CDCl_3$, 25 ºC): δ 8.78 (s, 1H, CH_{Ar}), 8.51 (dd, *J* = 2.9, 0.7 Hz, 1H, CH_{Ar}), 8.15 (dd, *J* = 8.6, 1.8 Hz, 1H, CH_{Ar}), 8.01 (d, *J* = 8.0 Hz, 1H, CH_{Ar}), 7.96 (d, *J* = 8.8 Hz, 1H, CH_{Ar}), 7.91 (d, *J* = 8.2 Hz, 1H, CH_{Ar}), 7.86 (dd, *J* = 1.5, 0.7 Hz, 1H, CH_{Ar}), 7.70–7.48 (m, 2H, 2CH_{Ar}), 6.57 (dd, *J* = 2.9, 1.5 Hz, 1H, CH_{Ar}); ^{13}C NMR {^{1}H} (75 MHz, $CDCl_3$, 25ºC): δ 166.4 (CO), 144.5 (CH_{Ar}), 135.4 (C_{Ar}), 133.7 (CH_{Ar}), 132.2 (C_{Ar}),

130.6 (CH_{Ar}), 129.7(CH_{Ar}), 128.7 (CH_{Ar}), 128.6 (C_{Ar}), 127.8 (CH_{Ar}), 127.7 (CH_{Ar}), 126.8 (CH_{Ar}), 126.7 (CH_{Ar}), 109.4 (CH_{Ar}); IR (cm^{-1}): ν 1696 (C=O), 1628 (N-C=O); HRMS m/z (ESI): $[M+Na]^+$ calc. for $C_{14}H_{10}N_2NaO$: 245.0685; found 245.0684.

Pyrazole 3p

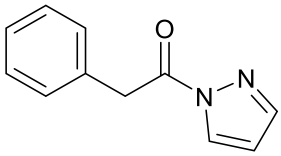

Following the general procedure, the reaction of 2-phenyl-*N'*-(prop-2-yn-1-yl)acetohydrazide (**1p**) (0.05 mmol, 10 mg) and AgNPs supported on silica gel (1% Ag) in 1,4-dioxane (1 mL) at 100 ºC for 2 h in the microwave and after flash chromatography using *n*-hexane/AcOEt (9:1) as eluent, afforded product **3p** (64%, 6.4 mg, 0.03 mmol) as a colorless oil; ^{1}H NMR (600 MHz, $CDCl_3$, 25 ºC): δ 8.27 (dd, *J* = 2.8, 0.6 Hz, 1H, CH), 7.79–7.71 (m, 1H, CH), 7.39 (dd, *J* = 2.1, 0.5 Hz, 1H, CH_{Ar}), 7.37 (dq, *J* = 1.4, 0.6 Hz, 1H, CH_{Ar}), 7.37–7.31 (m, 2H, CH_{Ar}), 7.30–7.27 (m, 1H, CH_{Ar}), 6.46 (dd, *J* = 2.8, 1.5 Hz, 1H, CH), 4.47 (s, 2H, CH_2); ^{13}C {^{1}H} (151 MHz, $CDCl_3$, 25ºC): δ 170.2 (C=O), 144.3 (CH), 133.4 (C_{Ar}), 129.9 (2 CH_{Ar}), 128.8 (2 CH_{Ar}), 128.7 (CH_{Ar}), 127.4 (CH), 110.1 (CH), 40.6 (CH_2); IR (cm^{-1}): ν 2929, 1680 (C=O); HRMS m/z (ESI): $[M+H]^+$ calc. for $C_{11}H_{11}N_2O$: 187.0866; found 187.0860. **3m** has ben previously reported using a different procedure (Li, T. Z.; Wang, X. B.; Sha, F.; Wu, X. Y. Organocatalyzed Enantioselective Mannich Reaction of Pyrazoleamides with Isatin-Derived Ketimines. *J. Org. Chem.* **2014**, *79*, 4332–4339).

Pyrazole 3q

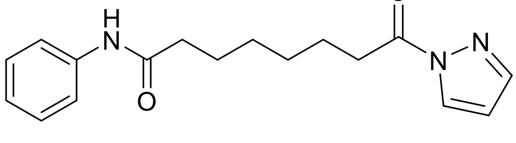

Following the general procedure, the reaction of 8-oxo-*N*-phenyl-8-(2-(prop-2-yn-1-yl)hydrazineyl)octanamide (**1q**) (0.03 mmol, 8.9 mg) and AgNPs supported on silica gel (1% Ag) in 1,4-dioxane (0.6 mL) at 100 ºC for 2.5 h in the microwave and after flash chromatography using *n*-

hexane/AcOEt (1:1) as eluent, afforded product **3q** (65%, 5.8 mg, 0.02) as a colorless oil; ^{1}H NMR (600 MHz, $CDCl_3$, 25 ºC): δ 8.27 (dd, *J* = 2.8, 0.8 Hz, 1H, CH), 7.76–7.69 (m, 1H, CH), 7.53 (d, *J* = 7.4 Hz, 2H, CH_{Ar}), 7.33 (t, *J* = 8.0 Hz, 2H, CH_{Ar}), 7.19 (s, 1H, NH), 7.11 (t, *J* = 7.4 Hz, 1H, CH_{Ar}), 6.45 (dd, *J* = 2.8, 1.4 Hz, 1H, CH), 3.16 (d, *J* = 7.4 Hz, 2H, CH_2), 2.38 (d, *J* = 7.3 Hz, 2H, CH_2), 1.84–1.77 (m, 4H, CH_2), 1.50–1.46 (m, 4H, CH_2); ^{13}C {^{1}H} (151 MHz, $CDCl_3$, 25ºC): δ 172.4 (C=O), 171.3 (C=O), 144.0 (CH), 138.1 (C_{Ar}), 129.1 (2 CH_{Ar}), 128.4 (CH), 124.3 (CH_{Ar}), 119.9 (2 CH_{Ar}), 109.6 (CH), 37.8 (CH_2), 34.0 (CH_2), 28.9 (CH_2), 28.9 (CH_2), 25.5 (CH_2), 24.3 (CH_2); IR (cm^{-1}): ν 3300, 2927, 1736 (C=O), 1541, 1441; HRMS m/z (ESI): [M+Na]$^+$ calc. for $C_{17}H_{21}N_3NaO_2$: 322.1526; found 322.1528.

Oxadiazine 4

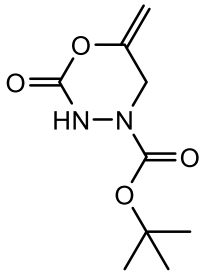

Following the general procedure for the preparation of oxadiazines **2**, the reaction of di-*tert*-butyl 1-(prop-2-yn-1-yl)hydrazine-1,2-dicarboxylate (**1-Boc**) (0.07 mmol, 20 mg), K_2CO_3 (0.30 mmol, 40.9 mg) and [$Ph_3PAuNTf_2$] (3% mol, 3.5 mg) in 1,4-dioxane (1.4 mL) at 30 ºC for 2.5 h and after flash chromatography using *n*-hexane/AcOEt (9:1) as eluent, afforded product **4** (quantitative yield, 15.8 mg, 0.07 mmol) as a yellow syrup; ^{1}H NMR (600 MHz, $CDCl_3$, 25 ºC): δ 6.60 (s, 1H, NH), 4.82 (q, *J* = 2.7 Hz, 1H, =CH), 4.37 (br s, 3H, =CH, CH_2), 1.48 (s, 9H, *t*-Bu); ^{13}C {^{1}H} (151 MHz, $CDCl_3$, 25ºC): δ 155.4 (C=O), 153.9 (C=O), 147.6 (C=), 88.6 (=CH_2), 83.0 (C-*t*-Bu), 49.6 (CH_2), 28.2 (3C, *t*-Bu); IR (cm^{-1}): ν 3306, 2981, 1798, 1682, 1250; HRMS m/z (ESI): [M-H]$^-$ calc. for $C_9H_{13}N_2O_4$: 213.0881; found 213.0881.

Oxadiazine 2g-BrBz

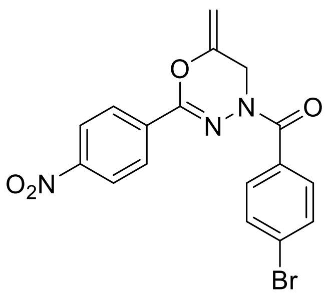

4-Bromobenzoyl chloride (0.06 mmol, 12.6 mg) was addet to a stirred solution of 6-methylene-2-(4-nitrophenyl)-5,6-dihydro-4*H*-1,3,4-oxadiazine (**2g**) (0.04 mmol, 8.4 mg) in dicloromethane (1 mL) and stirred at rt for 2 h. After that, the solvent was evaporated under reduced pressure and the mixture was purified by flash chromatography using *n*-hexane/AcOEt (8:2) as eluent, affording product **2g-BrBz** (77%, 11.8 mg, 0.03 mmol) as a yellow solid; mp 190-192 ºC; ^{1}H NMR (600 MHz, $CDCl_3$, 25 ºC): δ 8.24 (d, *J* = 9.0 Hz, 2H, CH_{Ar}), 7.95 (d, *J* = 8.9 Hz, 2H, CH_{Ar}), 7.71 (d, *J* = 8.6 Hz, 2H, CH_{Ar}), 7.60 (d, *J* = 8.5 Hz, 2H, CH_{Ar}), 5.06 (d, *J* = 2.6 Hz, 1H, CH), 4.74 (d, *J* = 2.7 Hz, 1H, CH), 4.54 (s, 2H, CH_2); ^{13}C {^{1}H} (151 MHz, $CDCl_3$, 25ºC): δ 168.2 (C=O), 149.2 (C_{Ar}), 146.5 (C=), 142.3 (C_{Ar}), 135.8 (C_{Ar}), 132.0 (C=N), 131.9 (2 CH_{Ar}), 131.2 (2 CH_{Ar}), 127.1 (2 CH_{Ar}), 126.3 (C_{Ar}), 123.9 (2 CH_{Ar}), 95.3 (CH), 40.5 (CH_2); IR (cm^{-1}): ν 1663 (C=O), 1522, 1350, 1312; HRMS m/z (ESI): $[M+Na]^+$ calc. for $C_{17}H_{12}BrN_3NaO_4$: 423.9903; found 423.9911.

Oxadiazine 2g-Ac

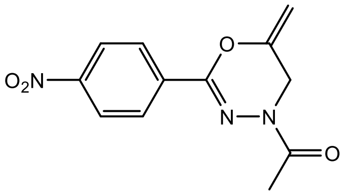

Oxadiazine **2g** (0.04 mmol, 8 mg) was solved in a mixture exceed of pyridine and acetic anhydride (1/1, 1mL) and stirred at rt for 14 h. After completion of the reaction (monitored by TLC), the solvent was evaporated and co-evaporated with toluene without further purification to afford product **2g-Ac** (quantitative yield, 9.5 mg) as a yellow solid; mp 196-198 ºC; ^{1}H NMR (600 MHz, $CDCl_3$, 25 ºC): δ 8.27 (d, *J* = 8.9 Hz, 2H, CH_{Ar}), 8.10 (d, *J* = 8.9 Hz, 2H, CH_{Ar}), 4.98 (d, *J* = 2.6 Hz, 1H, =CH_2), 4.65 (d, *J* = 3.1 Hz, 1H, =CH_2), 4.41 (s, 2H, CH_2), 2.42 (s, 3H, CH_3); ^{13}C {^{1}H} (151 MHz, $CDCl_3$, 25ºC): δ 171.0 (C=O), 149.1 (C_{Ar}), 146.4 (C=), 141.9 (C=N), 136.0 (C_{Ar}), 127.0 (2 CH_{Ar}), 123.8 (2 CH_{Ar}),

94.7 (=CH_2), 39.5 (CH_2), 20.7 (CH_3); IR (cm^{-1}): ν 2922, 1676 (C=O), 1349, 1100; HRMS m/z (ESI): $[M+H]^+$ calc. for $C_{12}H_{12}N_3O_4$: 262.0822; found 262.0815.

General procedure for the synthesis of oxadiazines 2-Ac. The appropriate alkyne **1** (1 mmol, 1 equiv) was solved in 1,4-dioxane (40 mL) at 30°C. Then, K_2CO_3 (4 mmol, 4 equiv) and [$Ph_3PAuNTf_2$] (5% mol, 0.05 mmol, 0.05 equiv) were sequentially added to the solution and stirred at 30°C until consumption of alkyne **1** (monitored by TLC). Next, the 1,4-dioxane was evaporated in vacuo ant the residue was solved in a mixture exceed of pyridine and acetic anhydride (1/1, 20 mL) and stirred at rt for 14 h. The residue was purified by flash chromatography on silica gel eluting with ethyl acetate/hexanes mixtures to afford the corresponding acetylated oxadiazines. Spectroscopic and analytical data for compounds **2-Ac** follow.

Oxadiazine 2o-Ac

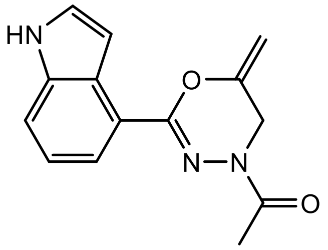

From 8.5 mg (0.04 mmol) of alkyne **1o**, and after column chromatography using *n*-hexane/AcOEt (7:3) as eluent gave compound **2o-Ac** (quantitative yield, 9.5 mg, 0.04 mmol) as a pale brown syrup; ^1H NMR (600 MHz, acetone-d_6, 25°C): δ 10.64 (s, 1H, NH), 7.71 (dd, *J* = 7.5, 1.0 Hz, 1H, CH_{Ar}), 7.63 (dt, *J* = 8.1, 1.0 Hz, 1H, CH_{Ar}), 7.49 (t, *J* = 2.8 Hz, 1H, CH_{Ar}), 7.23–7.19 (m, 1H, CH_{Ar}), 7.17 (tt, *J* = 2.2, 0.9 Hz, 1H, CH_{Ar}), 4.96 (d, *J* = 2.1 Hz, 1H, =CH_2), 4.72 (dt, *J* = 1.9, 0.9 Hz, 1H, = CH_2), 4.48–4.40 (m, 2H, CH_2), 2.41 (s, 3H, CH_3); ^{13}C {^1H} (151 MHz, acetone-d_6, 25°C): δ 172.1 (C=O), 150.8 (C=), 147.3 (C=N), 139.7 (C_{Ar}), 129.0 (CH_{Ar}), 127.7 (CH_{Ar}), 123.8 (CH_{Ar}), 123.2 (CH_{Ar}), 121.4 (CH_{Ar}), 116.9 (CH_{Ar}), 105.7 (CH_{Ar}), 94.6 (=CH_2), 41.5 (CH_2), 22.9 (CH_3); IR (cm^{-1}): ν 3286, 2928, 1654 (C=O), 1415, 1295; HRMS m/z (ESI): $[M+H]^+$ calc. for $C_{14}H_{14}N_3O_2$: 256.1081; found 256.1077.

Oxadiazine 2p-Ac

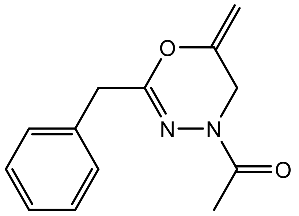

From 7.5 mg (0.04 mmol) of alkyne **1p**, and after column chromatography using *n*-hexane/AcOEt (8:2) as eluent gave compound **2p-Ac** (87%, 8 mg, 0.035 mmol) as a brown oil; ^{1}H NMR (600 MHz, $CDCl_3$, 25 ºC) δ 7.34–7.28 (m, 5H, CH_{Ar}), 4.69 (d, *J* = 2.2 Hz, 1H, = CH_2), 4.41 (d, *J* = 1.0 Hz, 1H, CH_2), 4.22 (s, 2H, CH_2), 3.61 (s, 2H, CH_2), 2.26 (s, 3H, CH_3); ^{13}C {^{1}H} (151 MHz, $CDCl_3$, 25ºC): δ 170.6 (C=O), 147.4 (C=), 147.3 (C=N), 135.3 (C_{Ar}), 129.0 (2 CH_{Ar}), 128.8 (2 CH_{Ar}), 127.3 (CH_{Ar}), 93.3 (C=), 39.1 (CH_2), 39.0 (CH_2), 20.6 (CH_3); IR (cm^{-1}): ν 2926, 1671 (C=O), 1409, 1244; HRMS m/z (ESI): $[M+Na]^+$ calc. for $C_{13}H_{14}N_2NaO_2$: 253.0947; found 253.0949.

Oxadiazine 2q-Ac

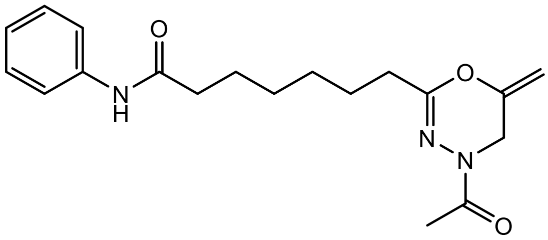

From 9 mg (0.03 mmol) of alkyne **1q**, and after column chromatography using *n*-hexane/AcOEt (2:1) as eluent gave compound **2q-Ac** (77%, 10 mg, 0.023 mmol) as a yellow syrup; ^{1}H NMR (600 MHz, $CDCl_3$, 25 ºC): δ 7.52 (d, *J* = 7.5 Hz, 2H, CH_{Ar}), 7.33 (t, *J* = 7.9 Hz, 2H, CH_{Ar}), 7.12 (t, *J* = 7.3 Hz, 1H, CH_{Ar}), 4.72 (d, *J* = 2.2 Hz, 1H, =CH_2), 4.48–4.37 (m, 1H, =CH_2), 4.24 (s, 2H, CH_2), 2.39–2.31 (m, 4H, 2CH_2), 2.26 (s, 3H, CH_3), 1.79–1.68 (m, 4H, 2 CH_2), 1.46–1.41 (m, 4H, 2 CH_2); ^{13}C {^{1}H} (151 MHz, $CDCl_3$, 25ºC): δ 173.8 (C=O), 173.0 (C=O), 151.2 (C=), 149.9 (C=N), 140.6 (C_{Ar}), 131.7 (2 CH_{Ar}), 126.9 (CH_{Ar}), 122.4 (2 CH_{Ar}), 95.4 (=CH_2), 41.5 (CH_2), 40.4 (CH_2), 34.7 (CH_2), 31.6 (CH_2), 31.3 (CH_2), 28.2 (CH_2), 28.1 (CH_2), 23.1 (CH_3); IR (cm^{-1}): ν 3308, 2929, 1662 (C=O), 1441; HRMS m/z (ESI): $[M+H]^+$ calc. for $C_{19}H_{26}N_3O_3$: 344.1969; found 344.1968.

Oxadiazine 4g

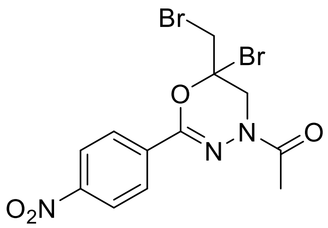

N-Bromosuccinimide (0.05 mmol, 9 mg) was added to a stirred solution of 1-(6-methylene-2-(4-nitrophenyl)-5,6-dihydro-4*H*-1,3,4-oxadiazin-4-yl)ethan-1-one (**2g-Ac**) (0.03 mmol, 6 mg) in nitromethane (1 mL) and the mixture was stirred at rt for 4 h. After that, the solvent was evaporated under reduced pressure and the mixture was purified by flash chromatography using *n*-hexane/AcOEt (8:2) as eluent, affording product **4g** (62%, 6 mg, 0.02 mmol) as a yellow oil; ^{1}H NMR (600 MHz, $CDCl_3$, 25 ºC): δ 8.28 (d, J = 9.1 Hz, 2H, CH_{Ar}), 8.05 (d, J = 9.1 Hz, 2H, CH_{Ar}), 4.95 (d, J = 13.5 Hz, 1H, CH_2), 4.15 (d, J = 11.8 Hz, 1H, CH_2), 3.97 (d, J = 11.8 Hz, 1H, CH_2), 3.70 (d, J = 13.4 Hz, 1H, CH_2), 2.50 (s, 3H, CH_3); ^{13}C {^{1}H} (151 MHz, $CDCl_3$, 25ºC): δ 171.1 (C=O), 149.3 (C_{Ar}), 140.5 (C=N), 135.8 (C_{Ar}), 127.1 (2 CH_{Ar}), 123.9 (2 CH_{Ar}), 91.7 (C-Br), 46.7 (CH_2), 35.5 (CH_2), 20.6 (CH_3); IR (cm^{-1}): ν 2926, 1687 (C=O), 1522, 1308; HRMS m/z (ESI): $[M+H]^+$ calc. for $C_{12}H_{12}Br_2N_3O_4$: 419.9189; found 419.9181.

Reaction of oxadiazine 2g-BrBz with NBS

N-Bromosuccinimide (0.06 mmol, 10.5 mg) was added to a stirred solution of (4-bromophenyl)(6-methylene-2-(4-nitrophenyl)-5,6-dihydro-4*H*-1,3,4-oxadiazin-4-yl)methanone (**2g-BrBz**) (0.03 mmol, 13.8 mg) in nitromethane (1 mL) and the mixture was stirred at rt for 3 h. After that, the solvent was evaporated under reduced pressure and the mixture was purified with flash chromatography using *n*-hexane/AcOEt (8:2) as eluent, affording the corresponding product as to isolable isomers (**5g**: 33%, 4.9 mg; **5g-rot**: 20%, 3 mg; total yield: 53%) as a yellow syrup.

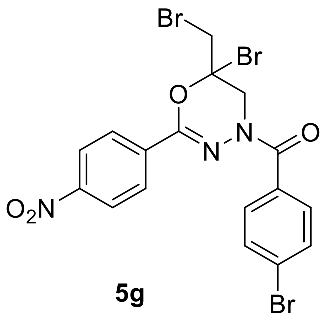

5g

Oxadiazine 5g. ^{1}H NMR (600 MHz, $CDCl_3$, 25 ºC): δ 8.25 (d, *J* = 8.6 Hz, 2H, CH_{Ar}), 7.89 (d, *J* = 8.6 Hz, 2H, CH_{Ar}), 7.74 (d, *J* = 8.2 Hz, 2H, CH_{Ar}), 7.63 (d, *J* = 8.3 Hz, 2H, CH_{Ar}), 5.06 (d, *J* = 13.7 Hz, 1H, CH_2), 4.43 (d, *J* = 11.8 Hz, 1H, CH_2), 4.19 (d, *J* = 11.9 Hz, 1H, CH_2), 3.98 (d, *J* = 13.6 Hz, 1H, CH_2); ^{13}C {^{1}H} (151 MHz, $CDCl_3$, 25ºC): δ 170.6 (C=O), 151.9 (C_{Ar}), 143.2 (C=N), 137.9 (C_{Ar}), 134.4 (2 CH_{Ar}), 134.2 (C_{Ar}), 133.8 (2 CH_{Ar}), 129.6 (2 CH_{Ar}), 129.0 (C_{Ar}), 126.5 (2 CH_{Ar}), 88.1 (C-Br), 51.3 (CH_2), 38.9 (CH_2); IR (cm^{-1}): ν 2925, 1710 (C=O), 1522, 1347; HRMS m/z (ESI): $[M-H]^-$ calc. for $C_{17}H_{11}Br_3N_3O_4$: 557.8305; found 557.8298.

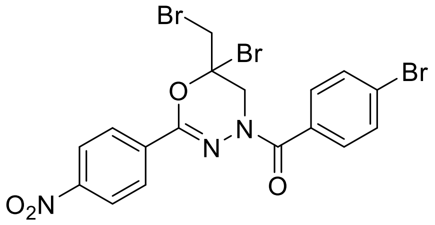

5g-rot

Oxadiazine 5g-rot. ^{1}H NMR (600 MHz, $CDCl_3$, 25 ºC): δ 8.25 (d, *J* = 9.1 Hz, 2H, CH_{Ar}), 7.89 (d, *J* = 9.1 Hz, 2H, CH_{Ar}), 7.70 (d, *J* = 8.6 Hz, 2H, CH_{Ar}), 7.62 (d, *J* = 8.6 Hz, 2H, CH_{Ar}), 5.09 (d, *J* = 14.1 Hz, 1H, CH_2), 4.38 (d, *J* = 12.1 Hz, 1H, CH_2), 3.98 (d, *J* = 12.1 Hz, 1H, CH_2), 3.62 (d, *J* = 14.0 Hz, 1H, CH_2); ^{13}C {^{1}H} (151 MHz, $CDCl_3$, 25ºC): δ 168.2 (C=O), 157.0 (C_{Ar}), 140.6 (C=N), 131.9 (2 CH_{Ar}), 131.7 (C_{Ar}), 131.3 (2 CH_{Ar}), 127.1 (2 CH_{Ar}), 124.3 (C_{Ar}), 124.1 (2 CH_{Ar}), 101.2 (C-Br), 43.4 (CH_2), 29.1 (CH_2); IR (cm^{-1}): ν 2923, 1694 (C=O), 1526, 1347; HRMS m/z (ESI): $[M+Na]^+$ calc. for $C_{17}H_{12}Br_3N_3NaO_4$: 581.8270; found 581.8277.

Radical trapping experiment with TEMPO

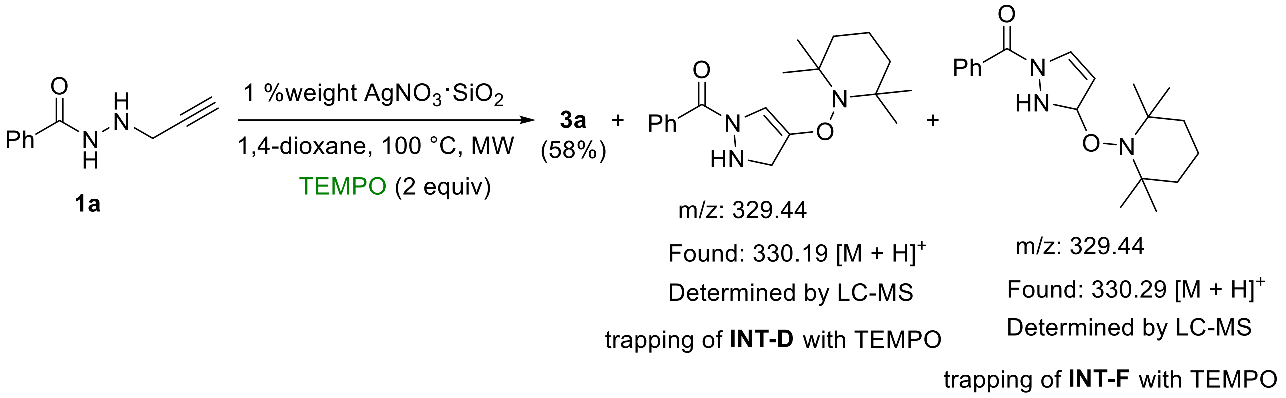
1 %weight $AgNO_3 \cdot SiO_2$
1,4-dioxane, 100 °C, MW
TEMPO (2 equiv)
1a
3a (58%)
m/z: 329.44
Found: 330.19 [M + H]+
Determined by LC-MS
trapping of **INT-D** with TEMPO
m/z: 329.44
Found: 330.29 [M + H]+
Determined by LC-MS
trapping of **INT-F** with TEMPO

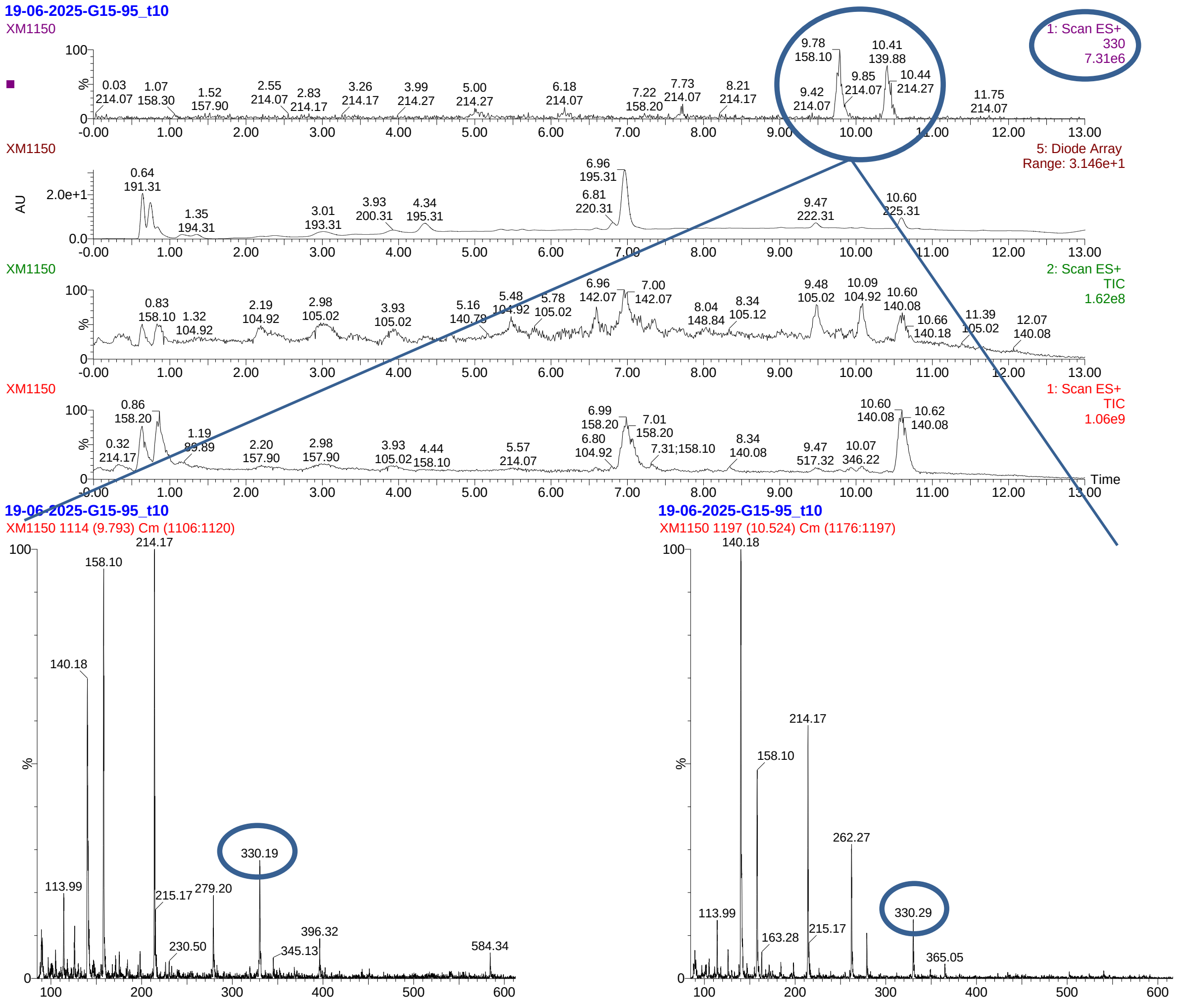
19-06-2025-G15-95_t10
XM1150
1: Scan ES+ 330 7.31e6
5: Diode Array Range: 3.146e+1
2: Scan ES+ TIC 1.62e8
1: Scan ES+ TIC 1.06e9
Time
XM1150 1114 (9.793) Cm (1106:1120)
330.19
XM1150 1197 (10.524) Cm (1176:1197)
330.29

4.- NMR Spectra

^{1}H NMR of compound **1a** (400 MHz, $CDCl_3$, 25 ºC)

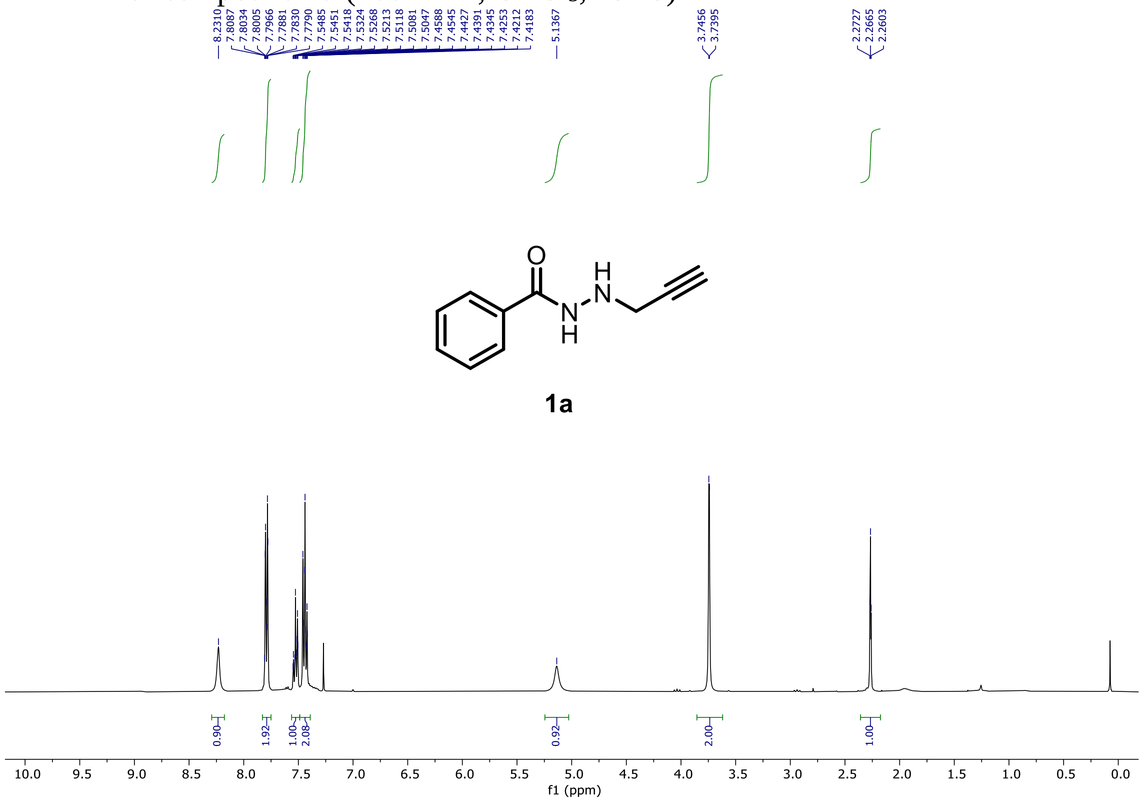

^{13}C NMR of compound **1a** (101 MHz, $CDCl_3$, 25 ºC)

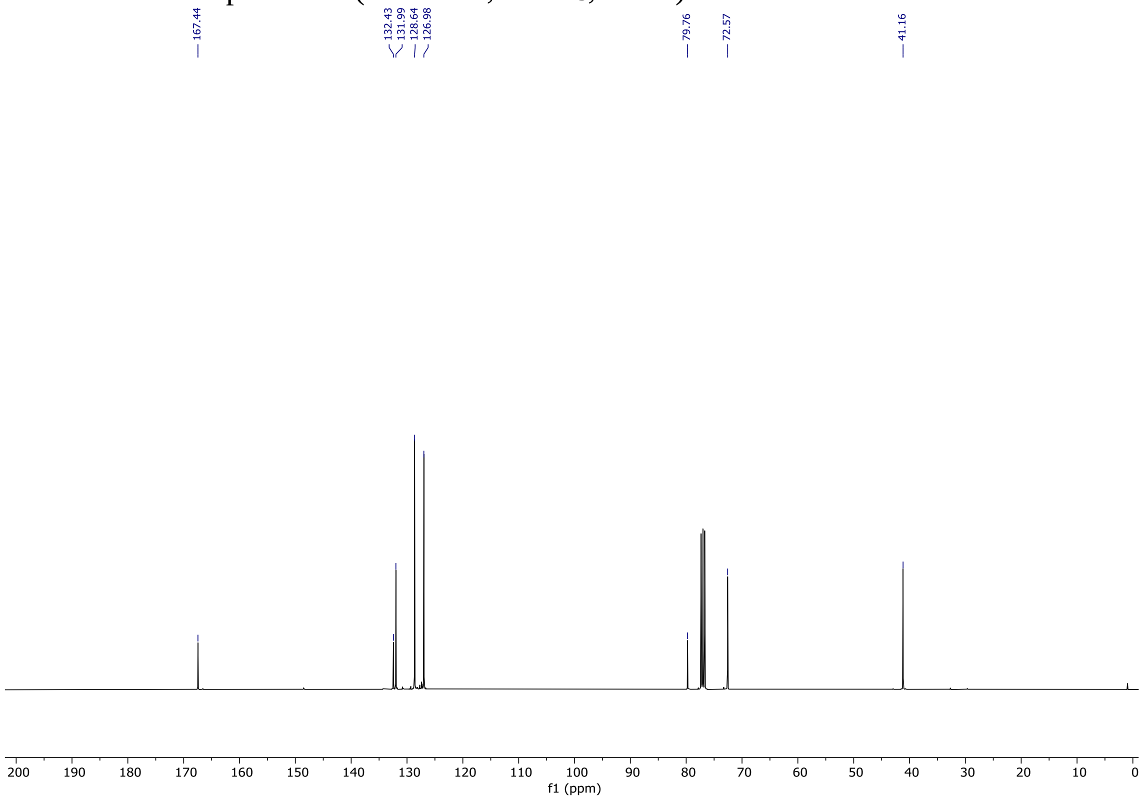

^{1}H NMR of compound **1b** (400 MHz, $CDCl_3$, 25 ºC)

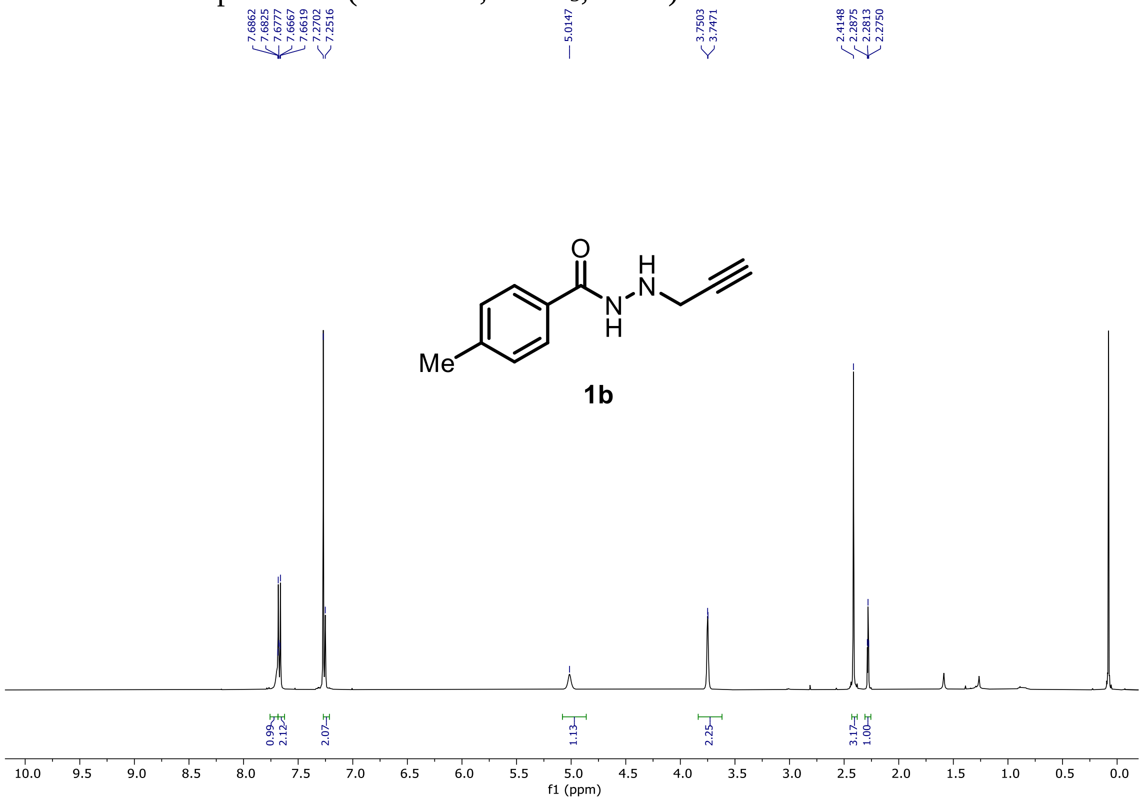

^{13}C NMR of compound **1b** (101 MHz, $CDCl_3$, 25 ºC)

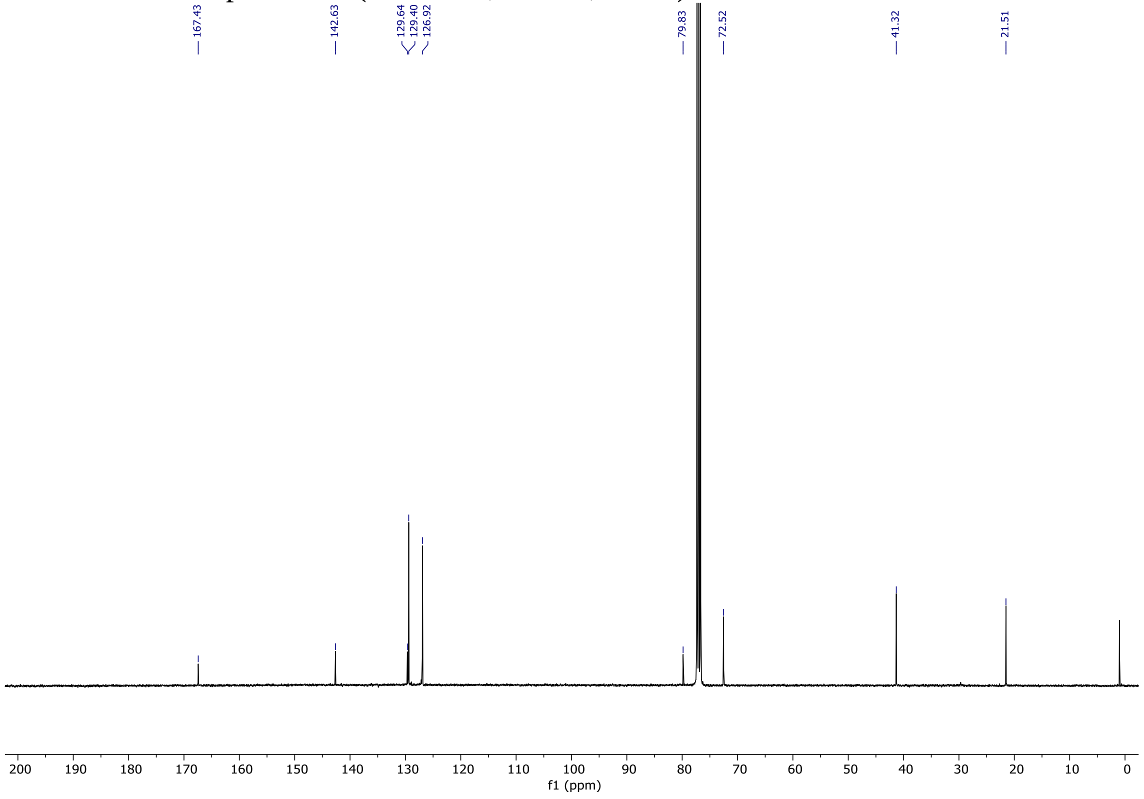

^{1}H NMR of compound **1c** (400 MHz, $CDCl_3$, 25 ºC)

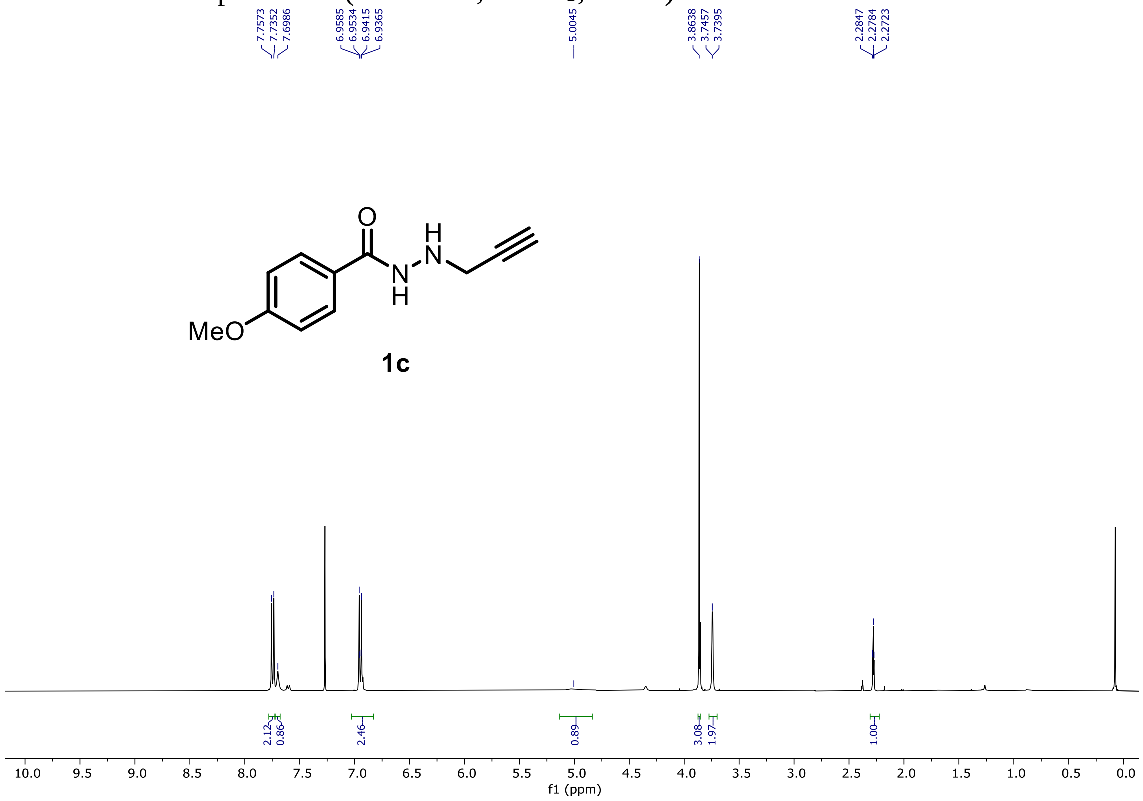

^{13}C NMR of compound **1c** (101 MHz, $CDCl_3$, 25 ºC)

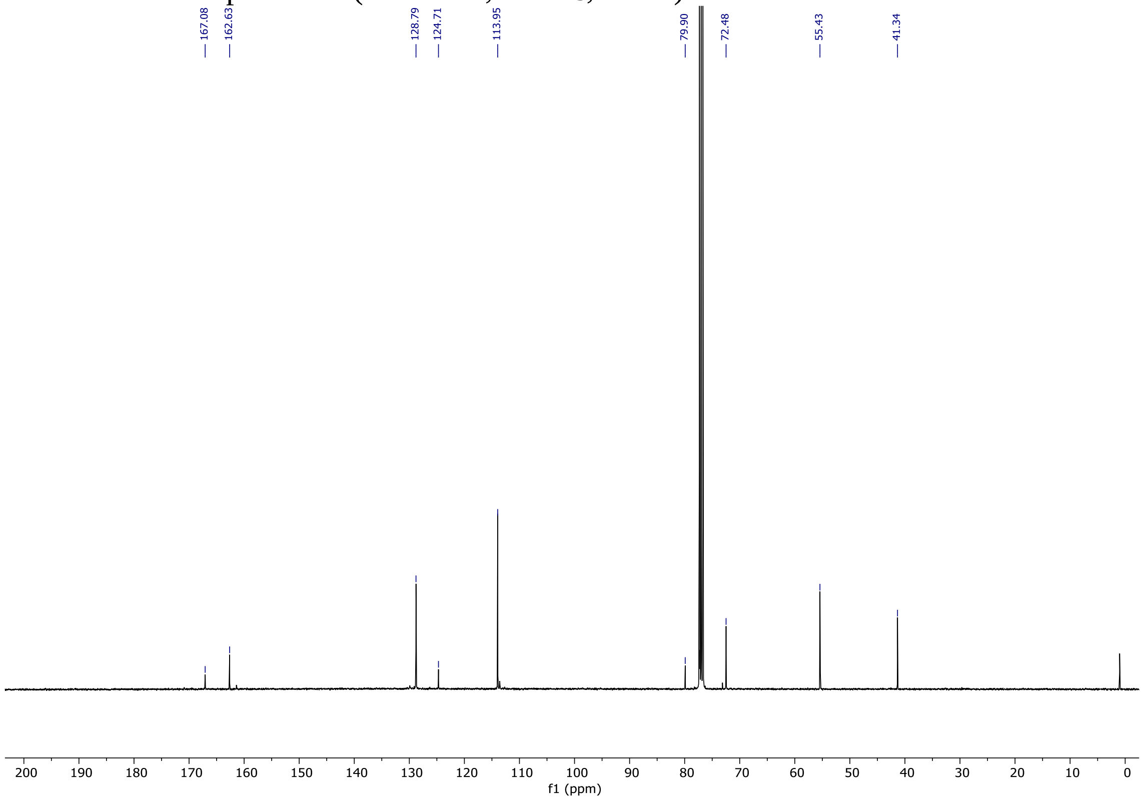

^{1}H NMR of compound **1-N** (300 MHz, $CDCl_3$, 25 ºC)

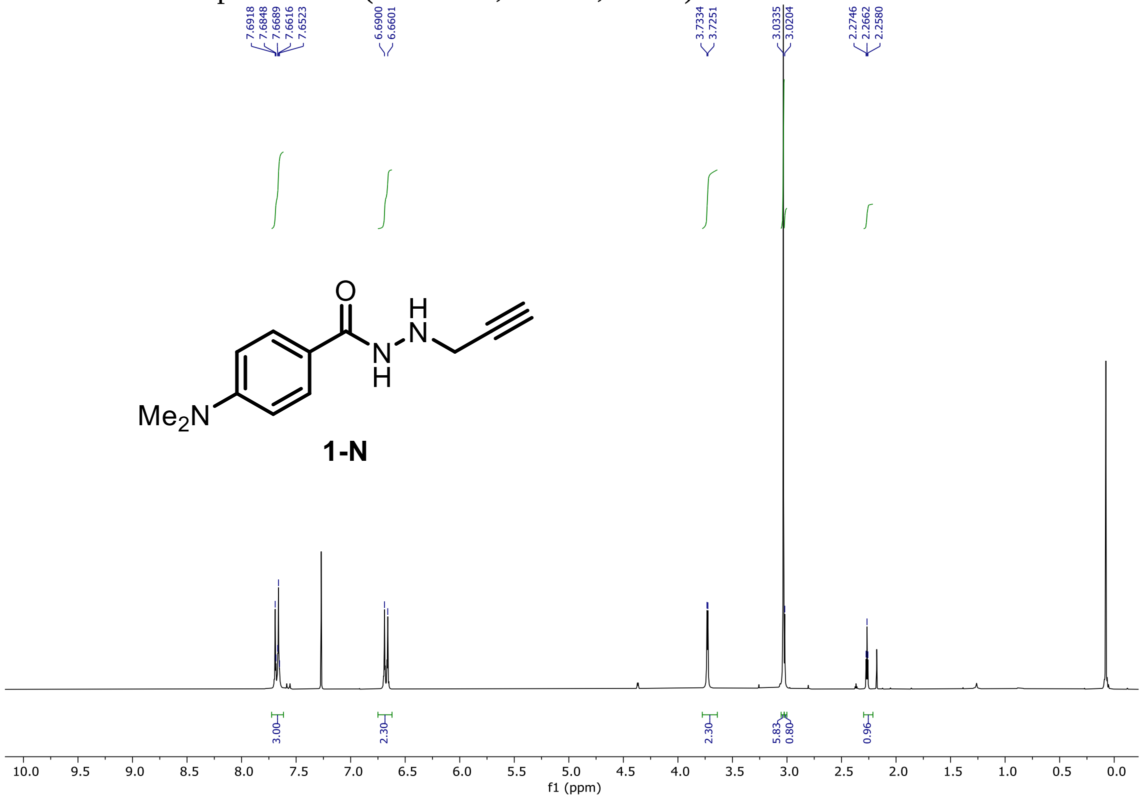

^{13}C NMR of compound **1-N** (75 MHz, $CDCl_3$, 25 ºC)

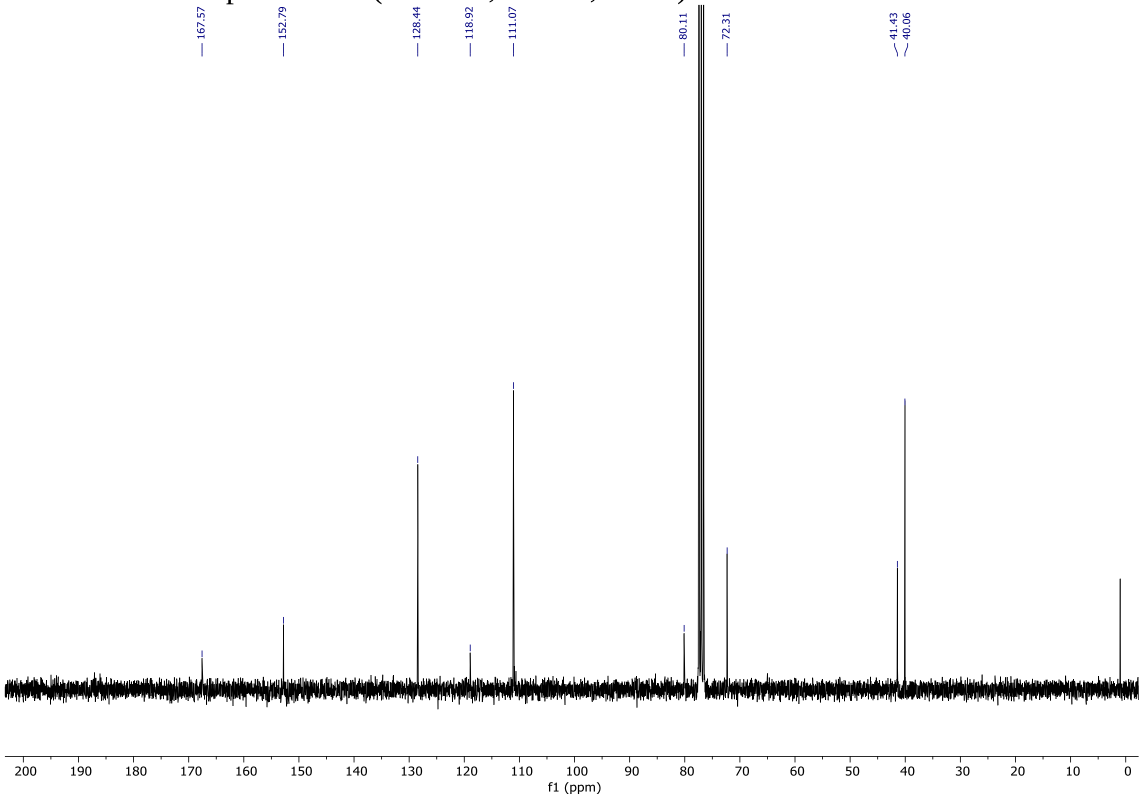

^{1}H NMR of compound **1d** (600 MHz, $CDCl_3$, 25 ºC)

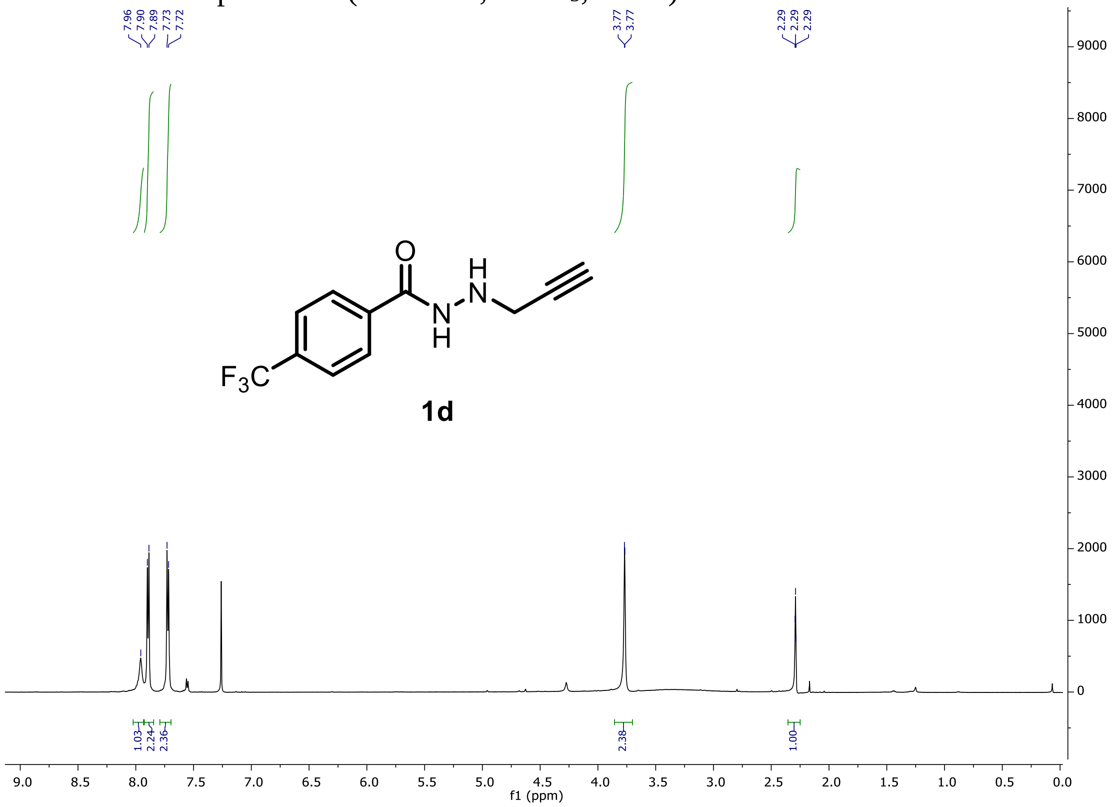

^{13}C NMR of compound **1d** (151 MHz, $CDCl_3$, 25 ºC)

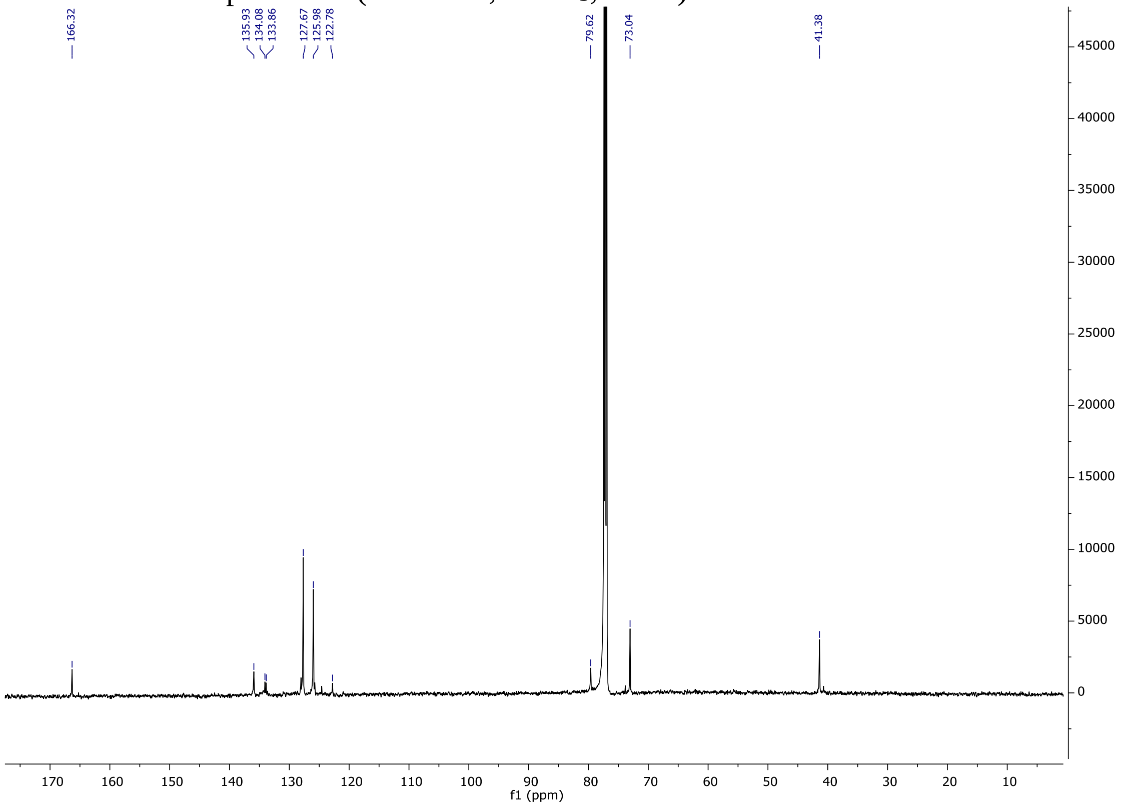

^{1}H NMR of compound **1e** (400 MHz, $CDCl_3$, 25 ºC)

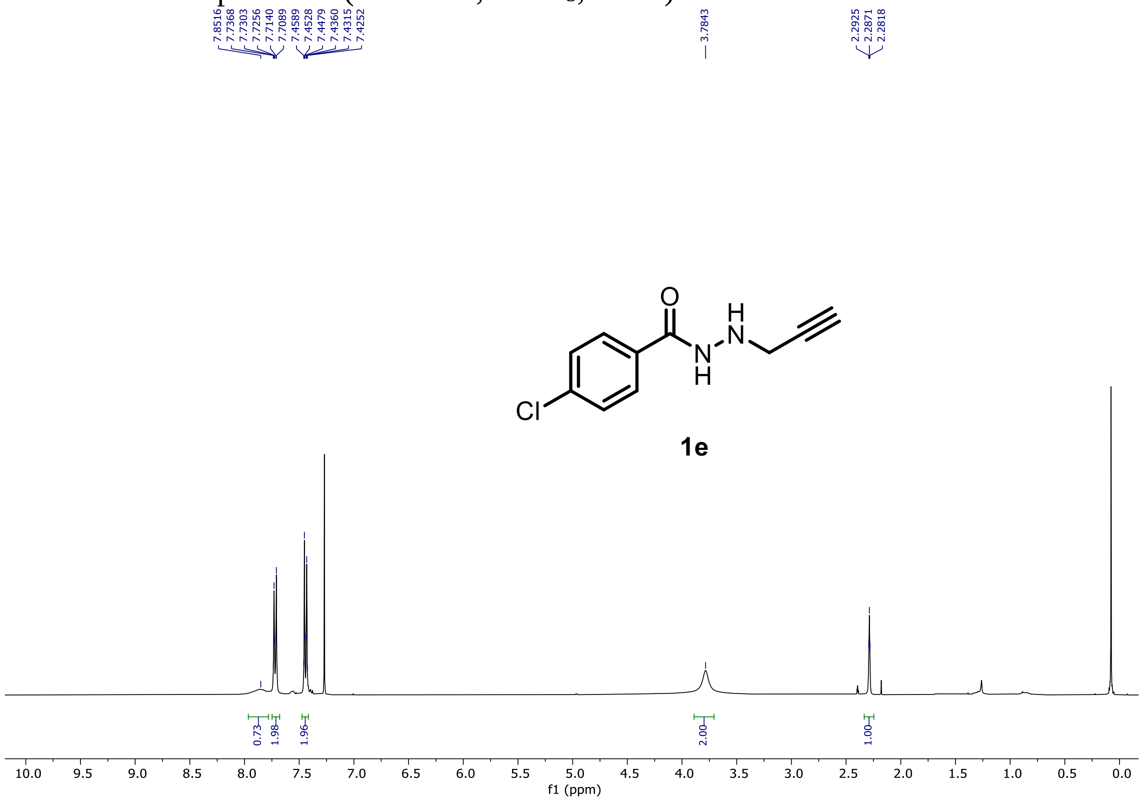

^{13}C NMR of compound **1e** (101 MHz, $CDCl_3$, 25 ºC)

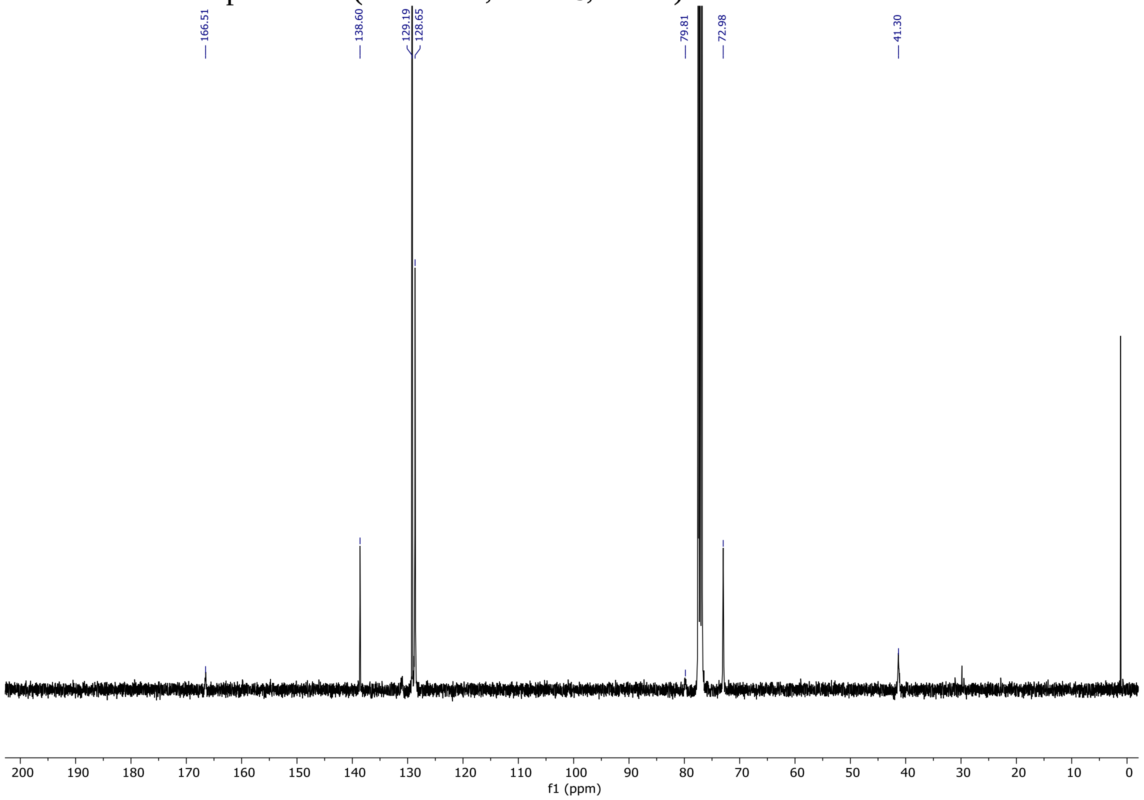

^{1}H NMR of compound **1f** (400 MHz, $CDCl_3$, 25 ºC)

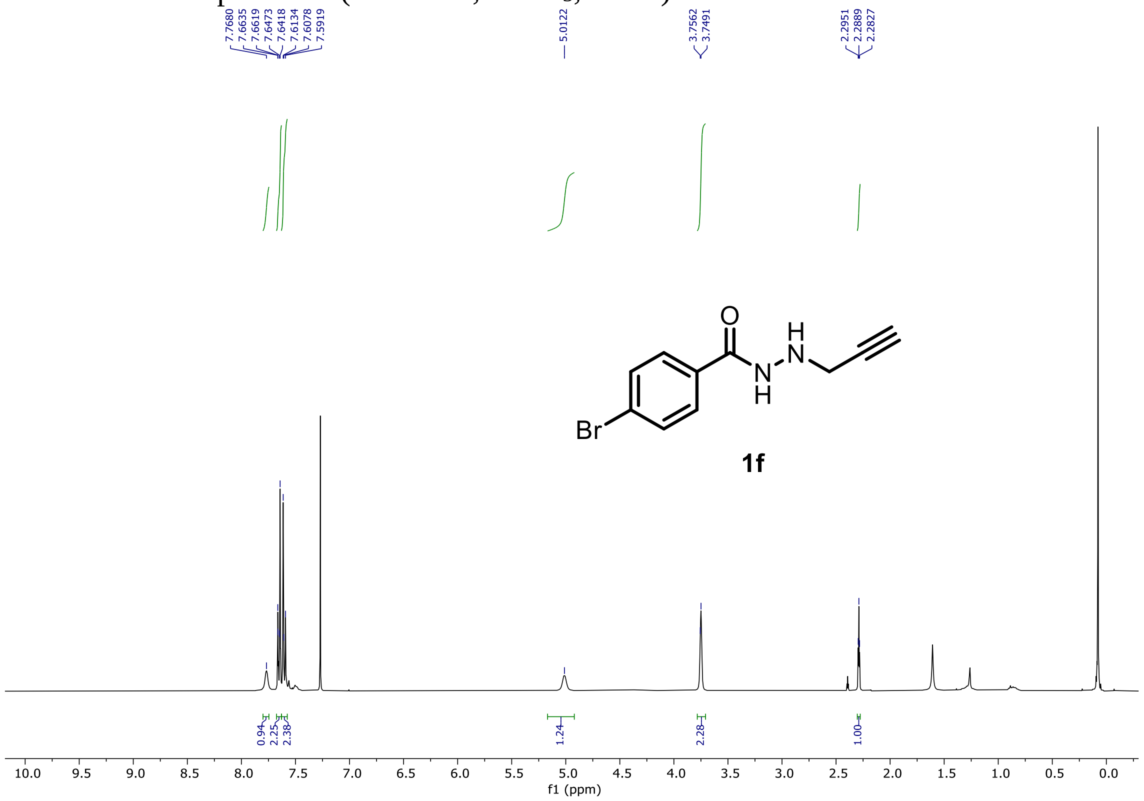

^{13}C NMR of compound **1f** (101 MHz, $CDCl_3$, 25 ºC)

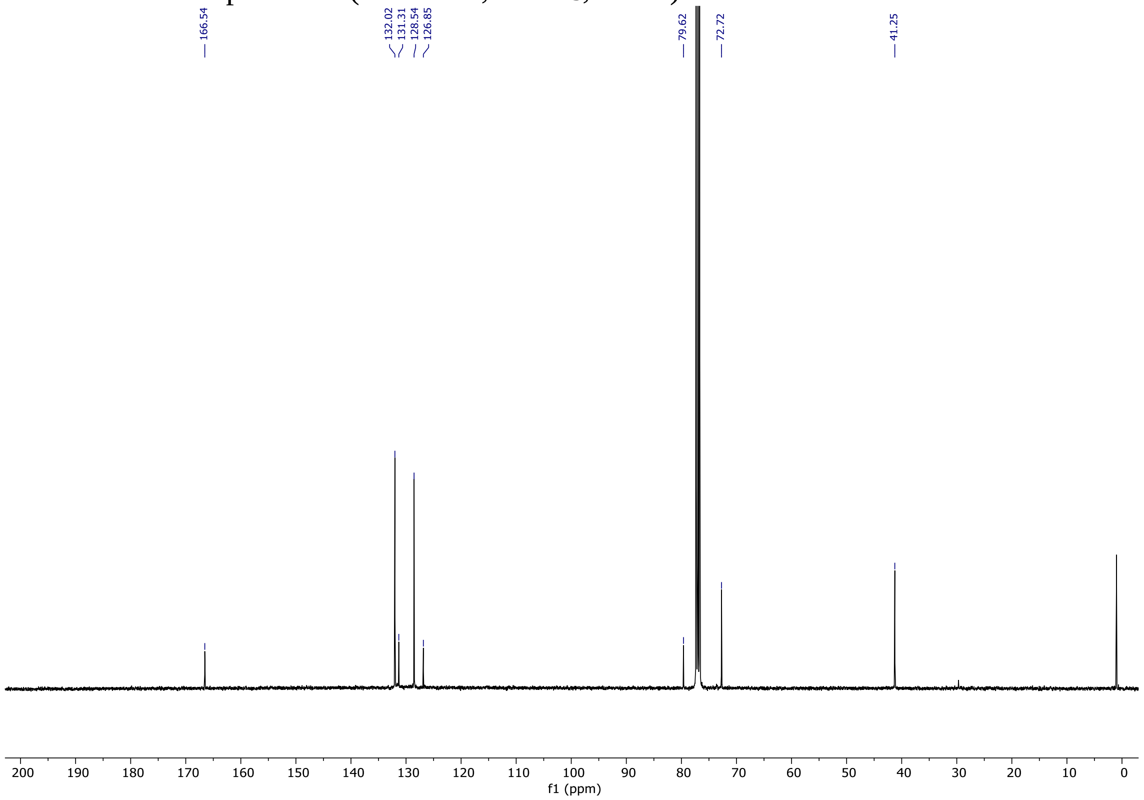

^{1}H NMR of compound **1g** (400 MHz, dmso-d_6, 25 ºC)

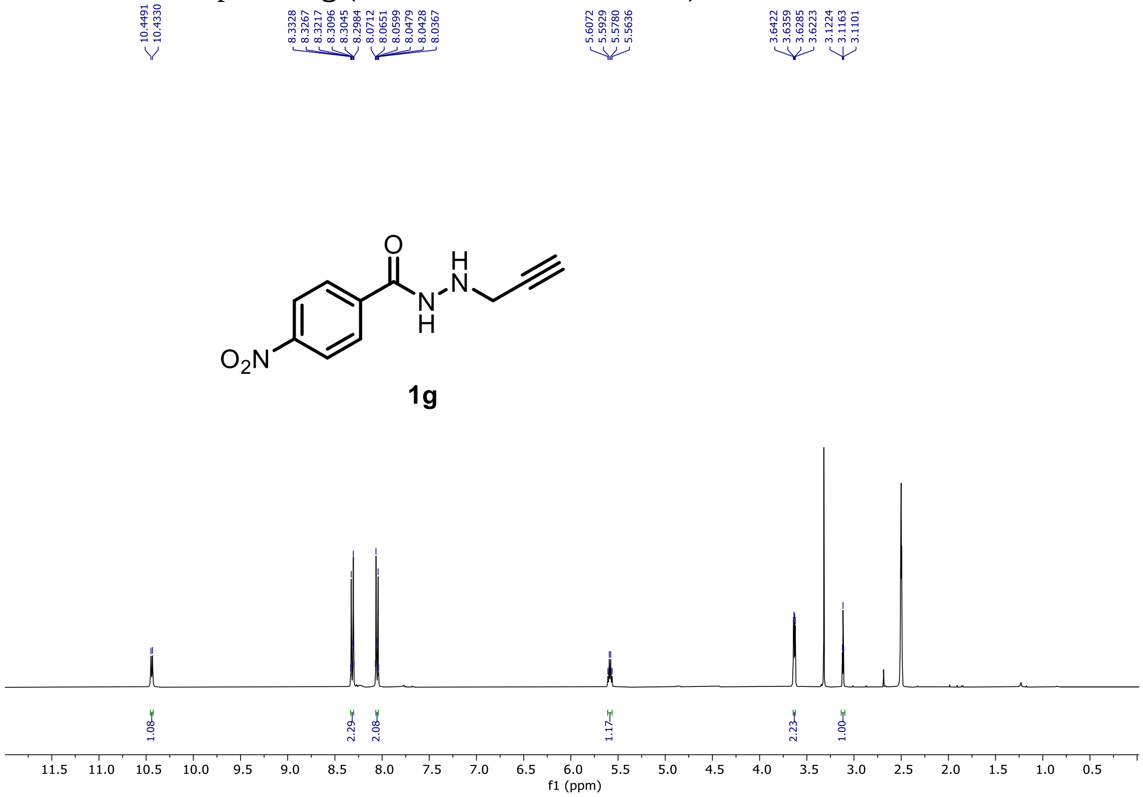

^{13}C NMR of compound **1g** (101 MHz, dmso-d_6, 25 ºC)

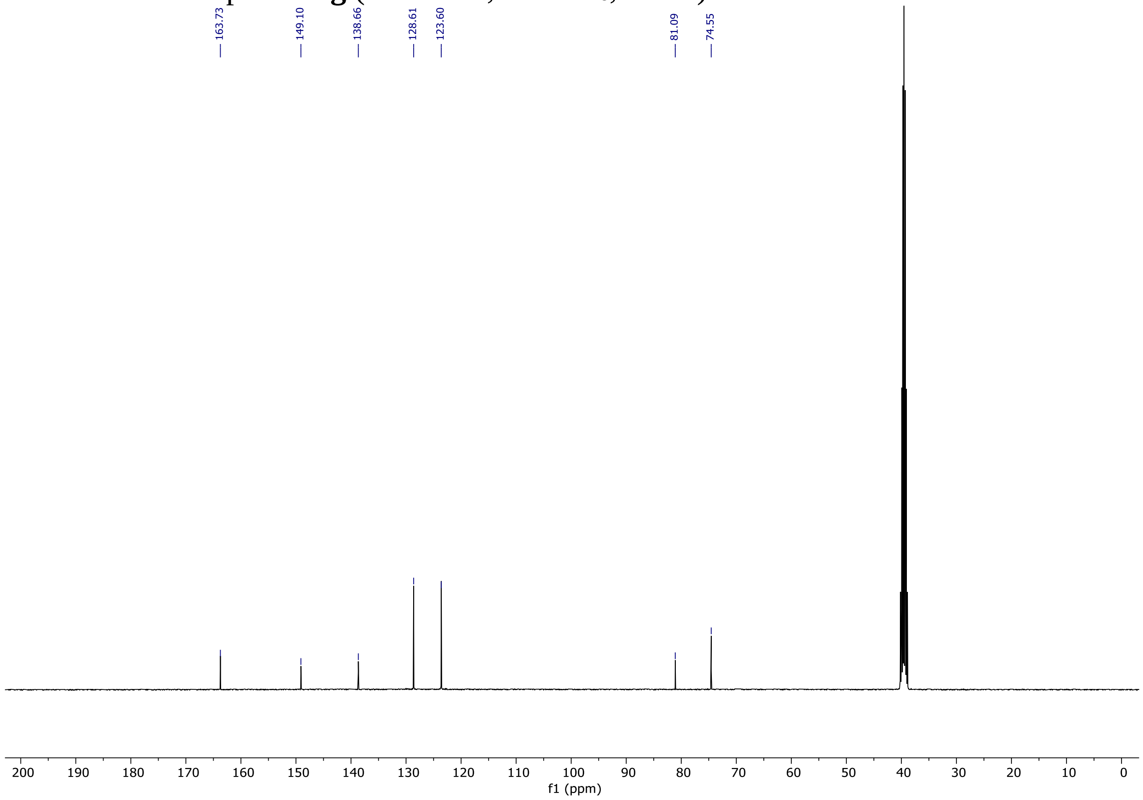

^{1}H NMR of compound **1h** (400 MHz, $CDCl_3$, 25 ºC)

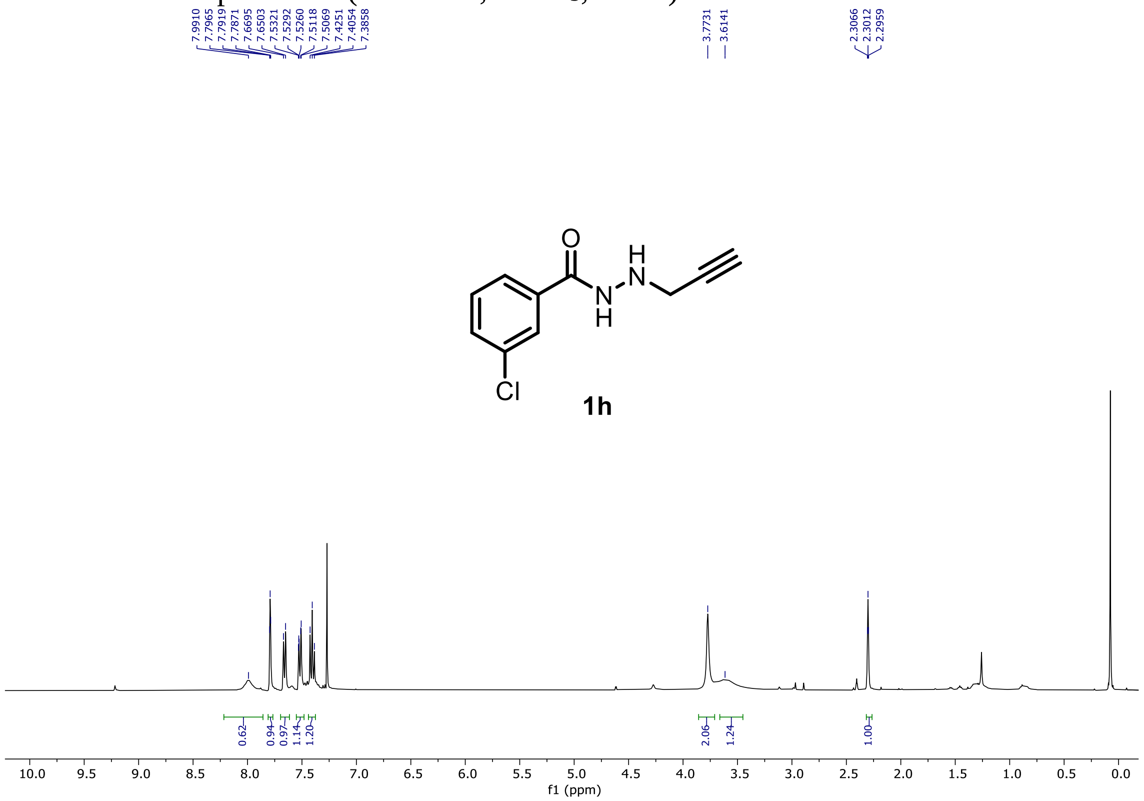

^{13}C NMR of compound **1h** (101 MHz, $CDCl_3$, 25 ºC)

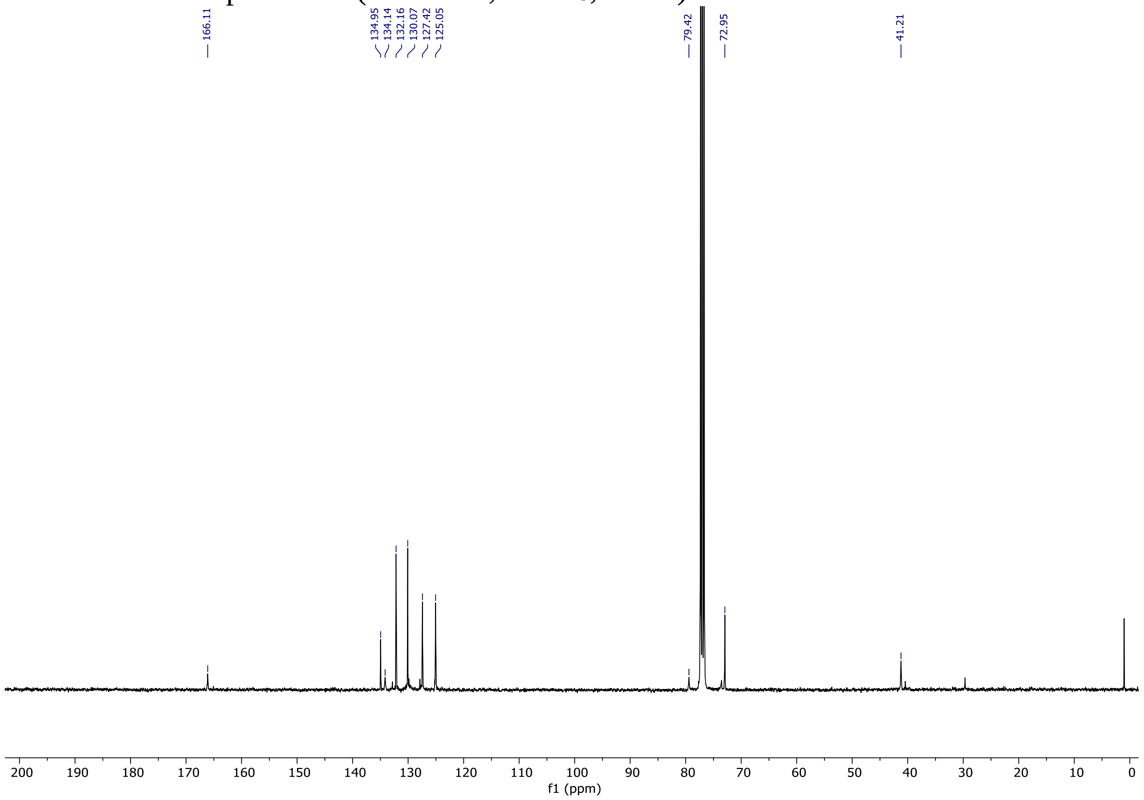

^{1}H NMR of compound **1i** (500 MHz, $CDCl_3$, 25 ºC)

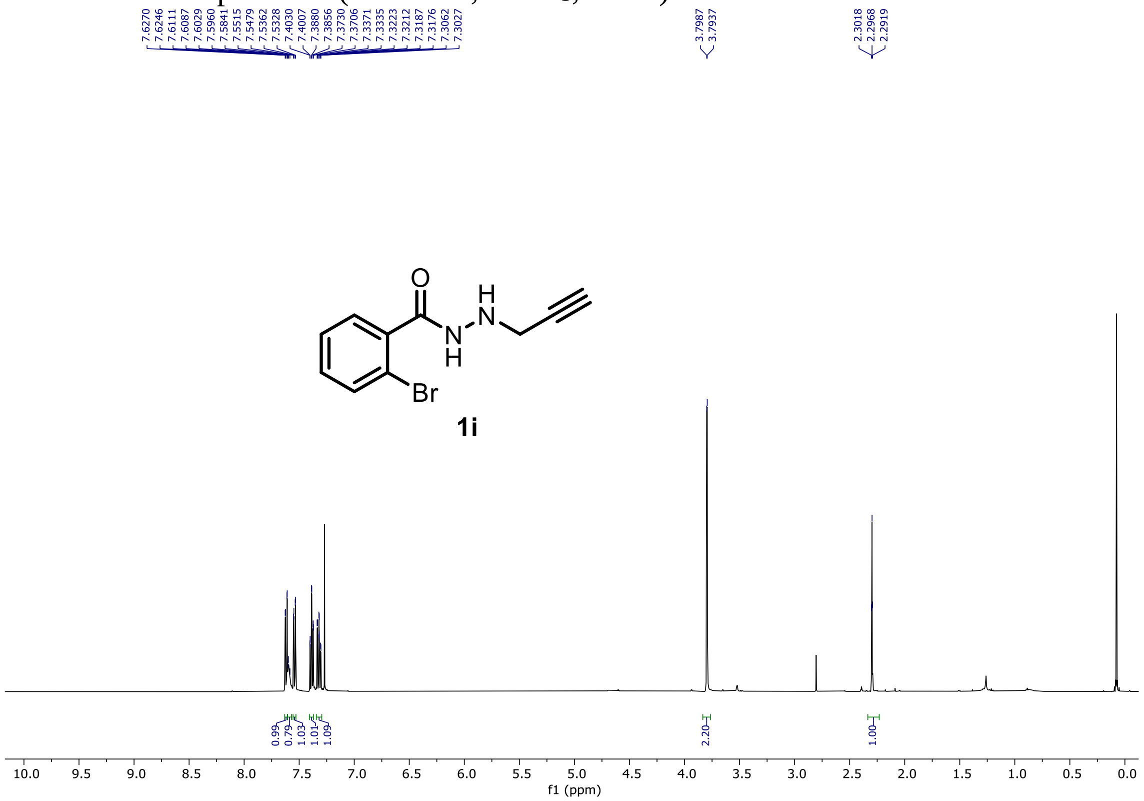

^{13}C NMR of compound **1i** (126 MHz, $CDCl_3$, 25 ºC)

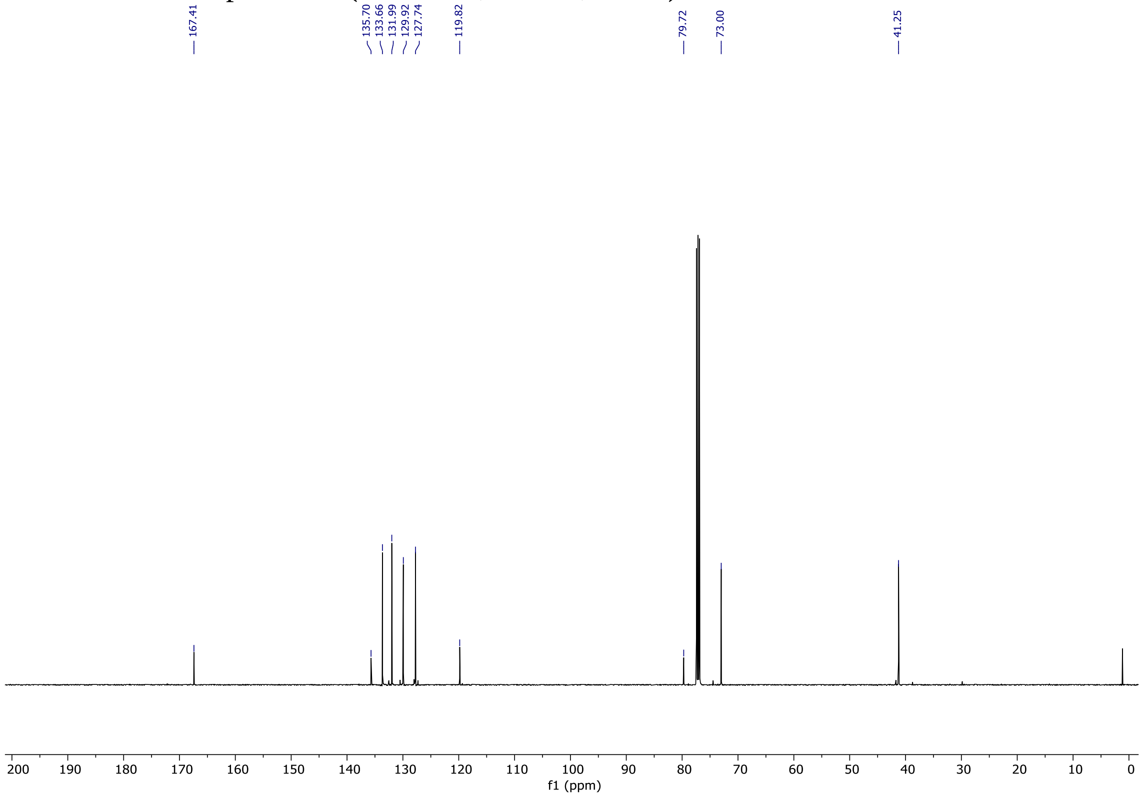

^{1}H NMR of compound **1j** (400 MHz, dmso-d_6, 25 ºC)

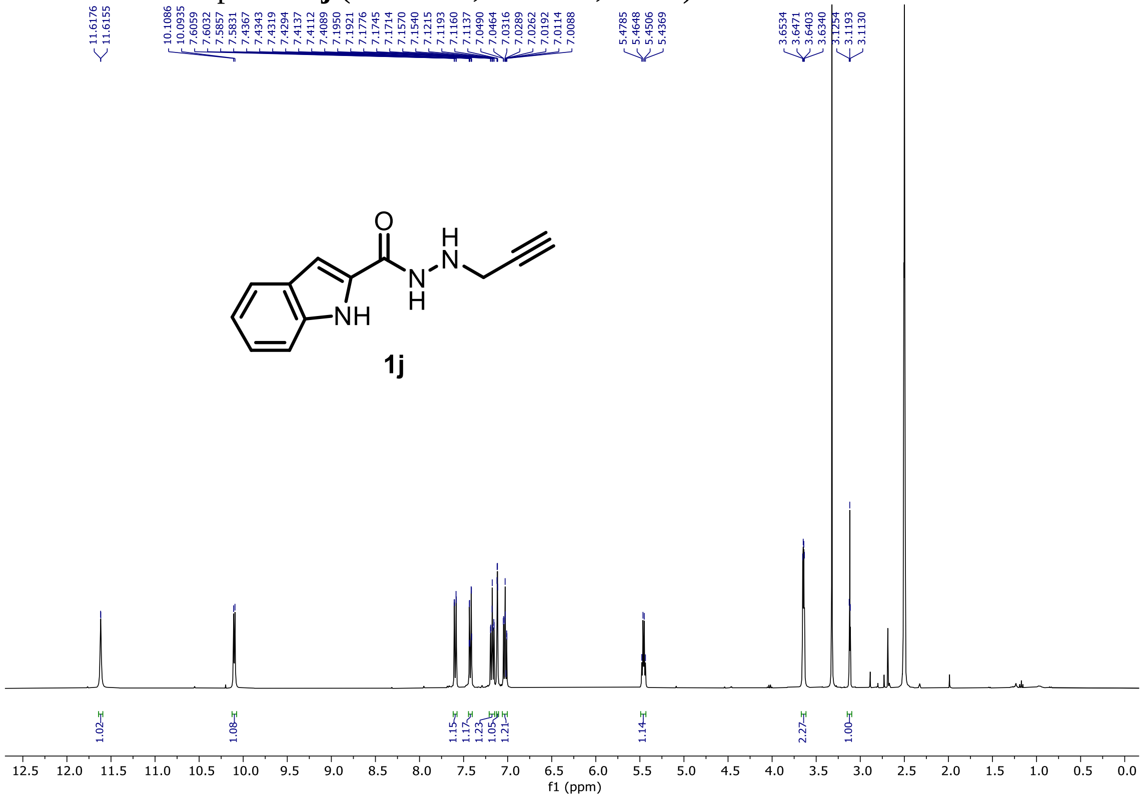

^{13}C NMR of compound **1j** (101 MHz, dmso-d_6, 25 ºC)

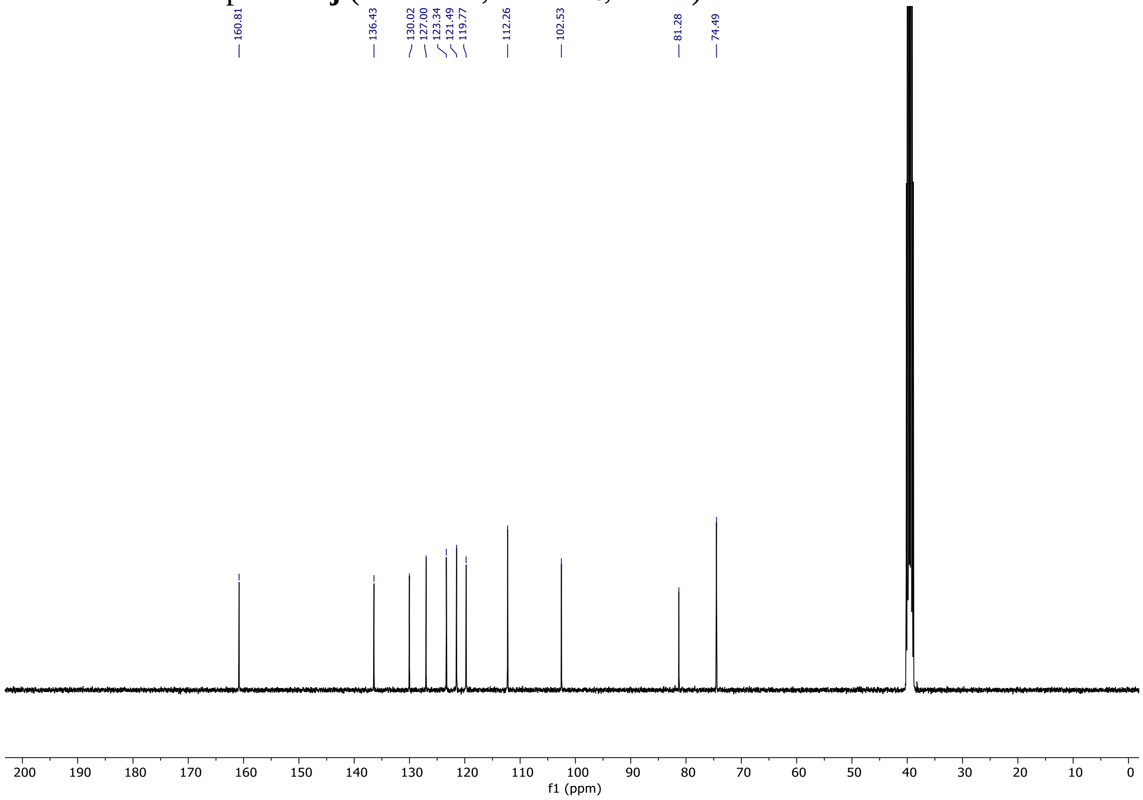

^{1}H NMR of compound **1k** (400 MHz, dmso-*d*$_6$, 25 ºC)

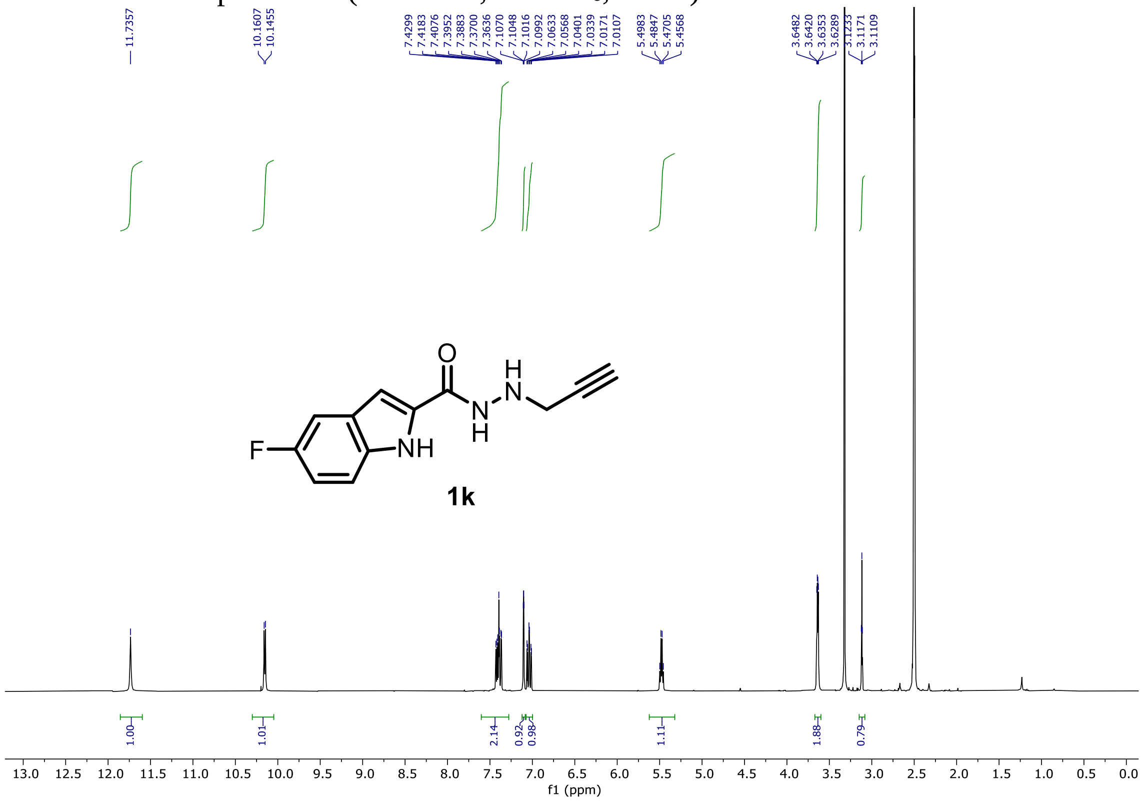

^{13}C NMR of compound **1k** (101 MHz, dmso-*d*$_6$, 25 ºC)

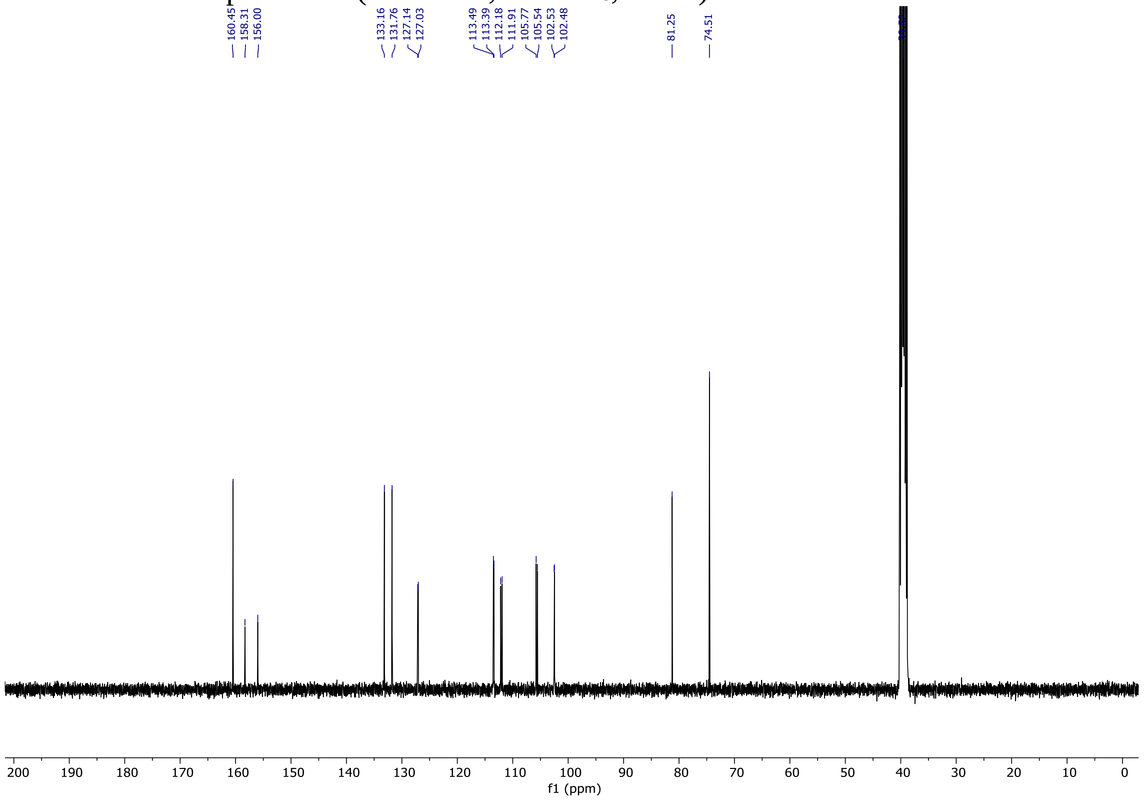

^{1}H NMR of compound **1l** (400 MHz, $CDCl_3$, 25 ºC)

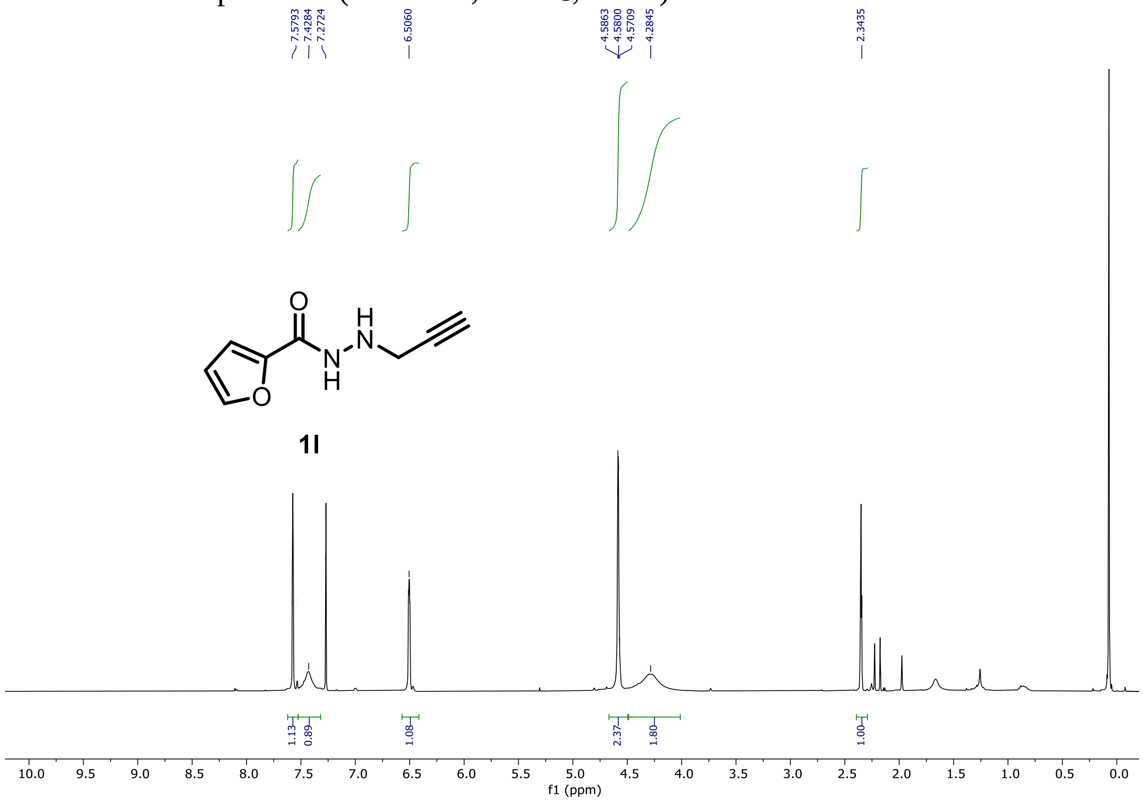

^{13}C NMR of compound **1l** (101 MHz, $CDCl_3$, 25 ºC)

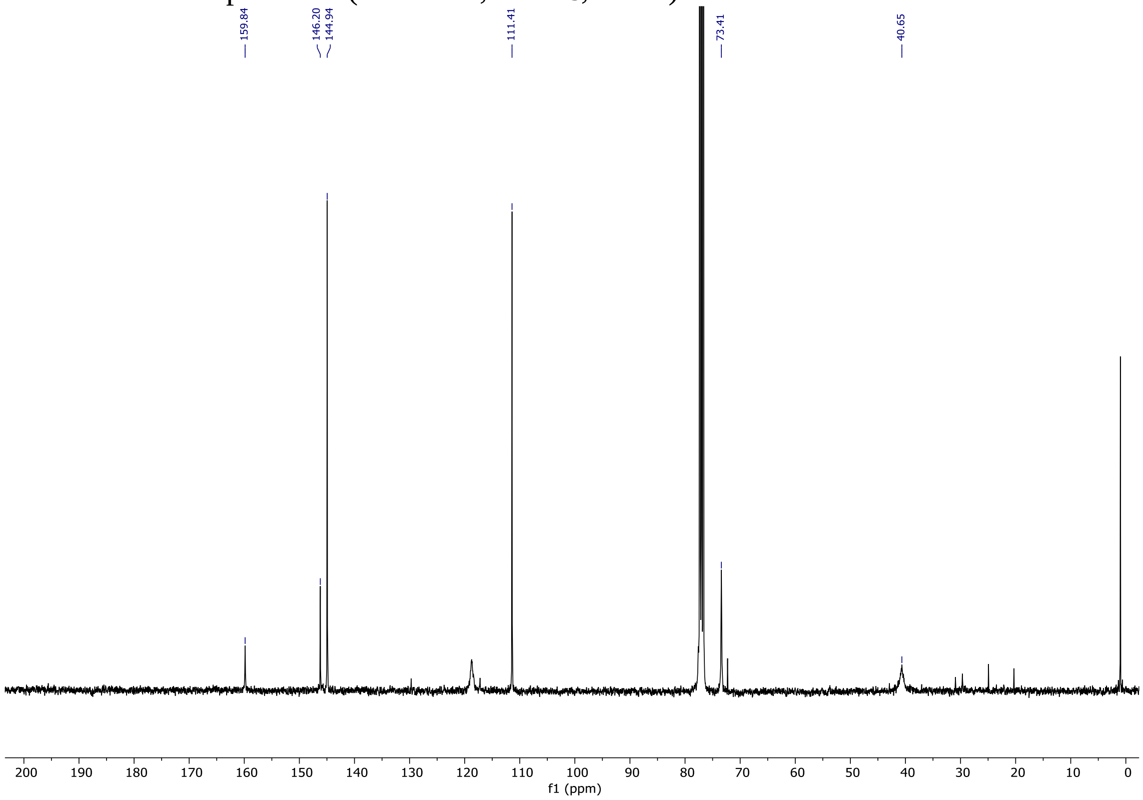

^{1}H NMR of compound **1m** (400 MHz, dmso-d_6, 25 ºC)

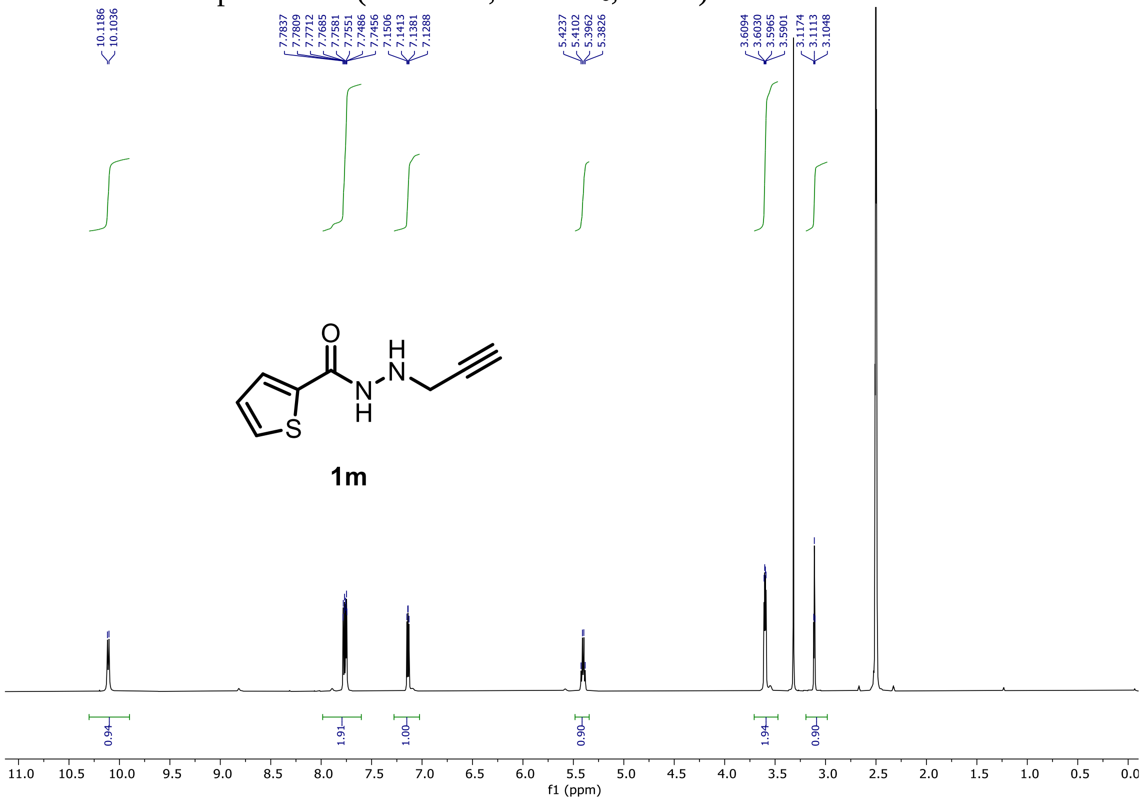

^{13}C NMR of compound **1m** (101 MHz, dmso-d_6, 25 ºC)

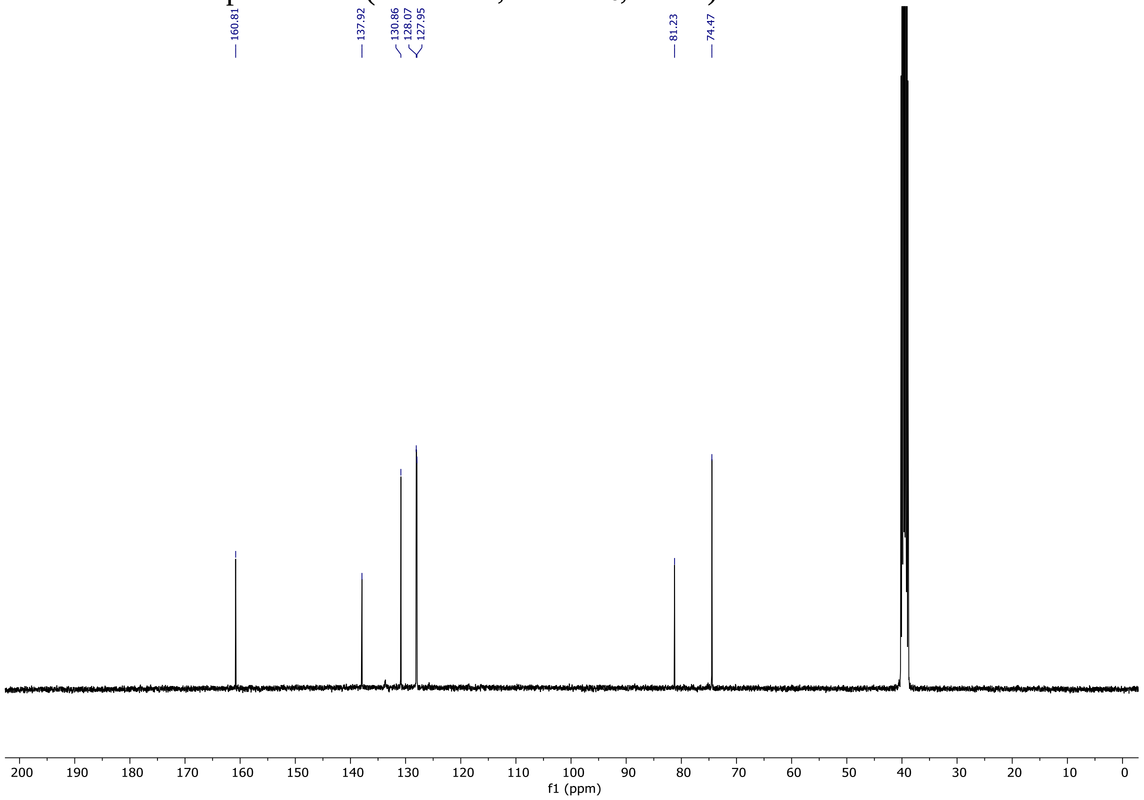

^{1}H NMR of compound **1n** (300 MHz, $CDCl_3$, 25 ºC)

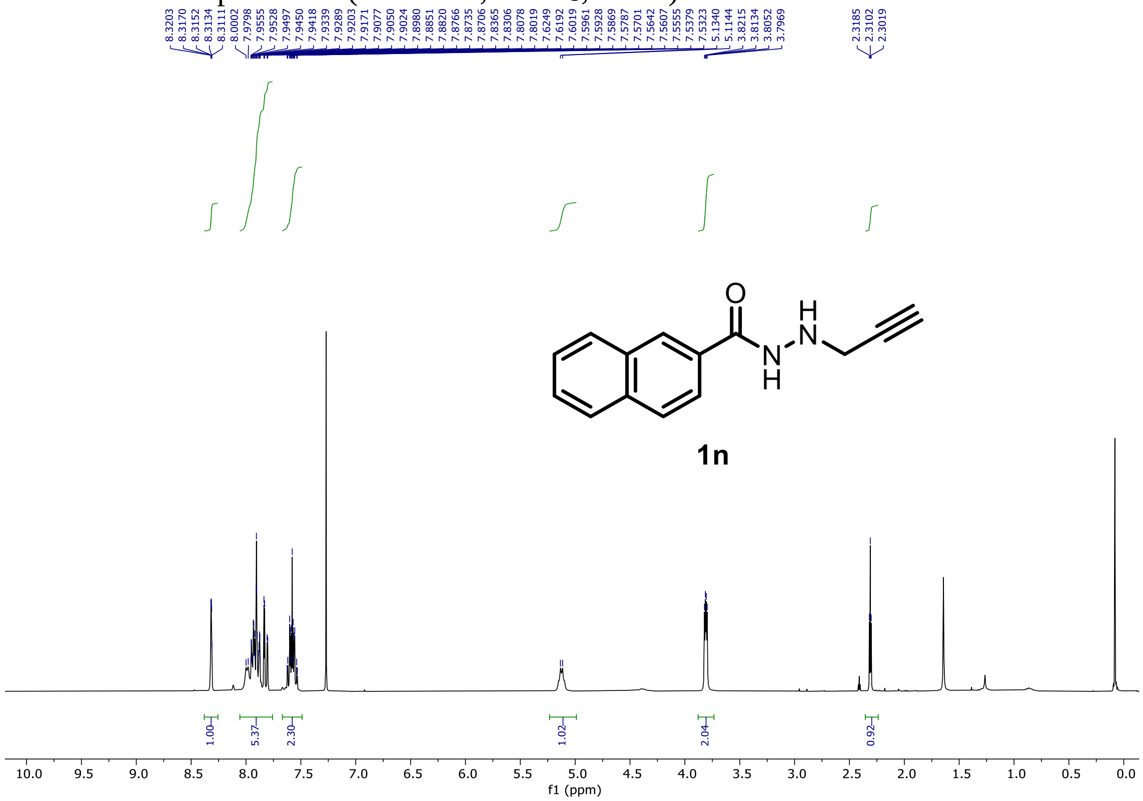

^{13}C NMR of compound **1n** (75 MHz, $CDCl_3$, 25 ºC)

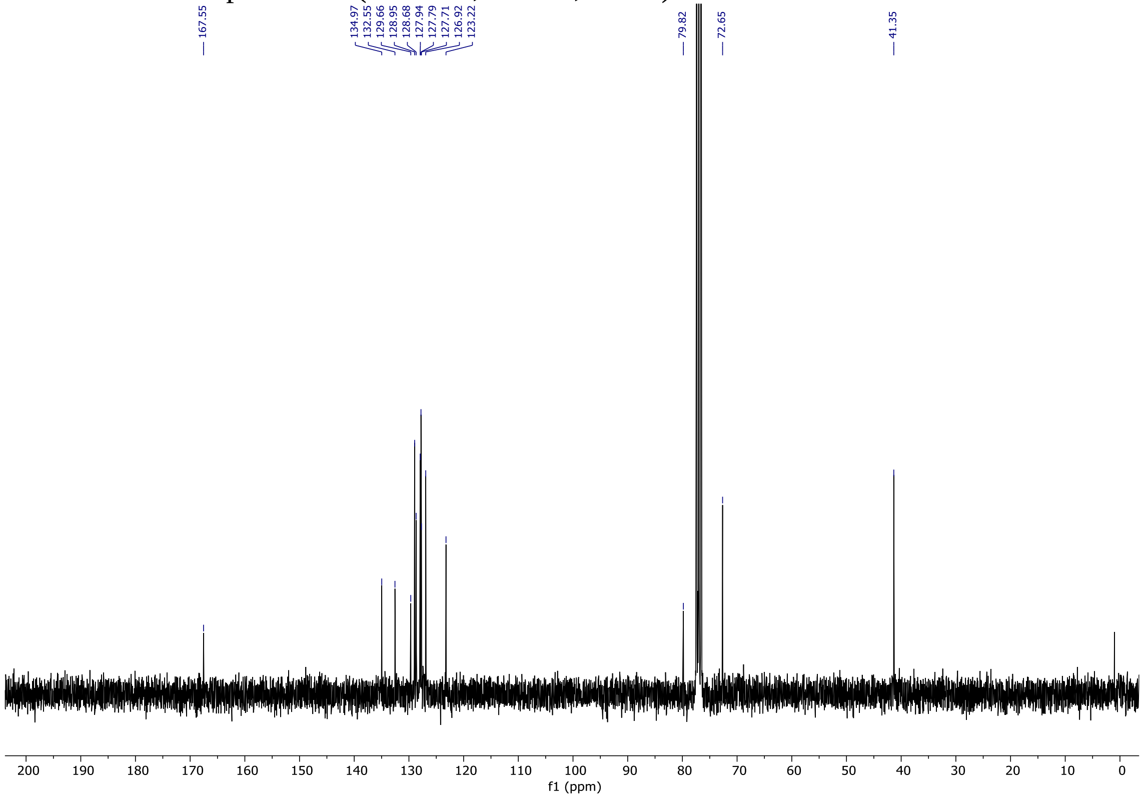

^{1}H NMR of compound **1n-N** (600 MHz, Acetone d$_6$, 25 °C)

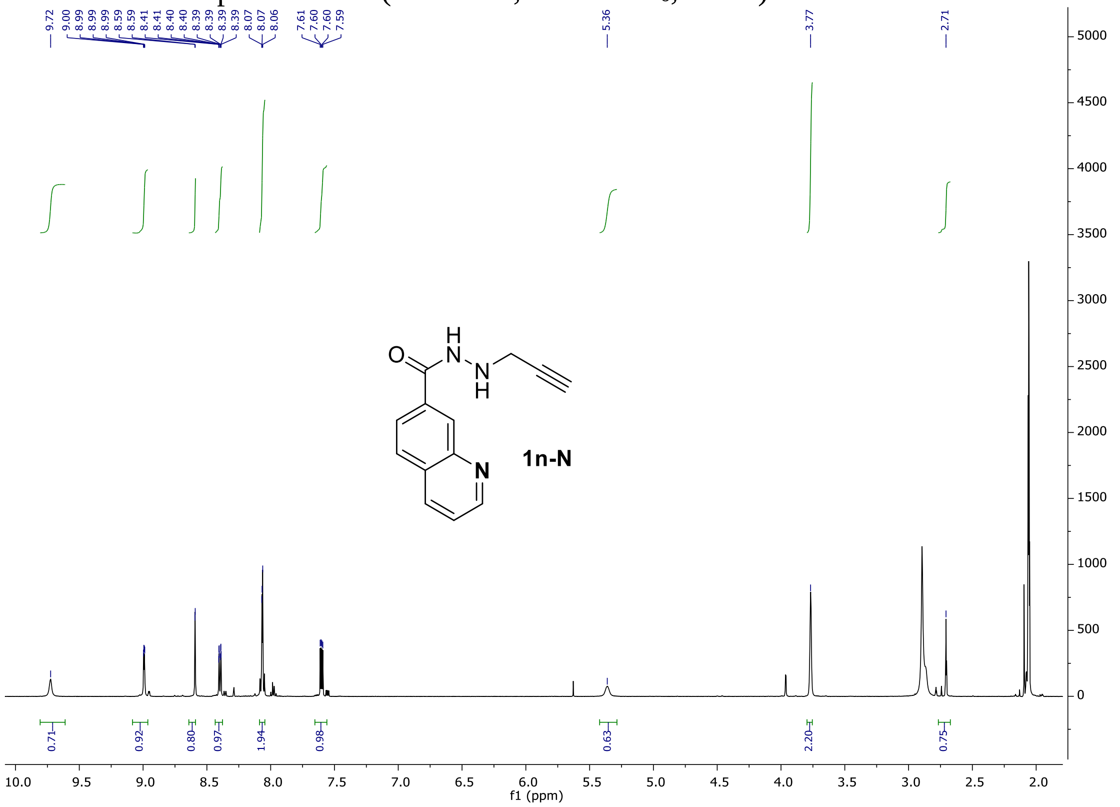

^{13}C NMR of compound **1n-N** (151 MHz, Acetone d$_6$, 25 °C)

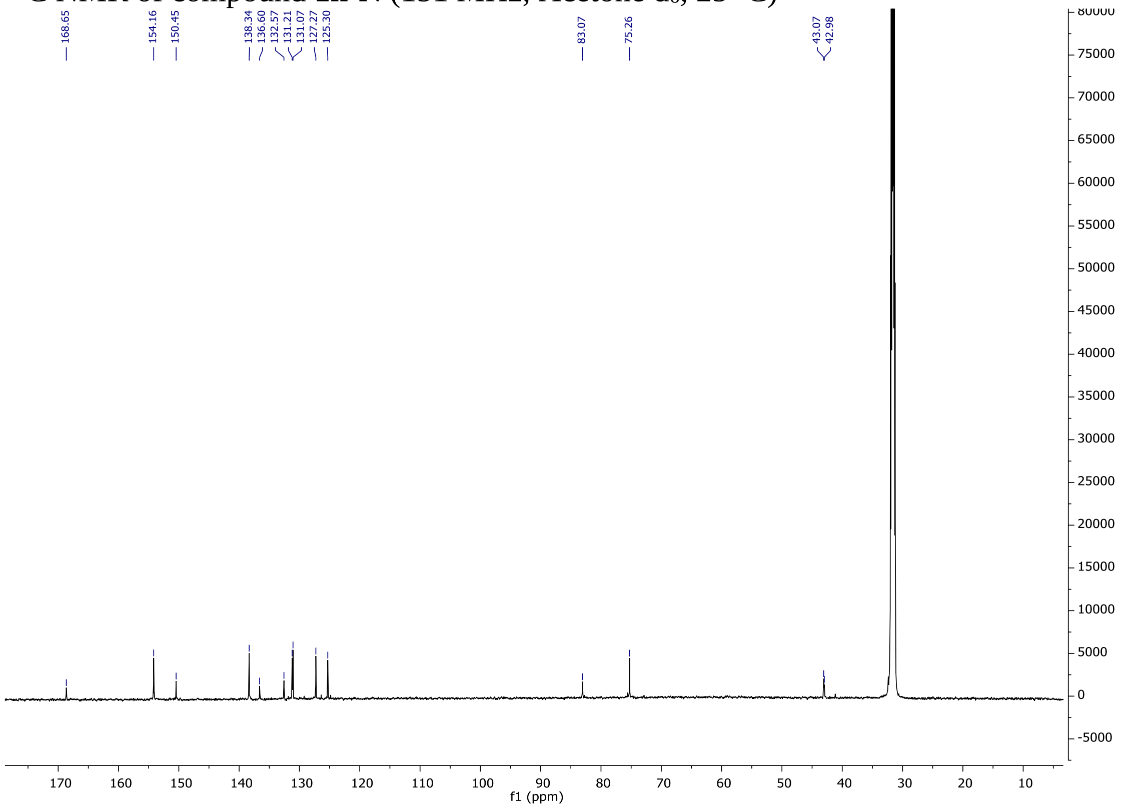

^{1}H NMR of compound **1o** (400 MHz, dmso-d_6, 25 ºC)

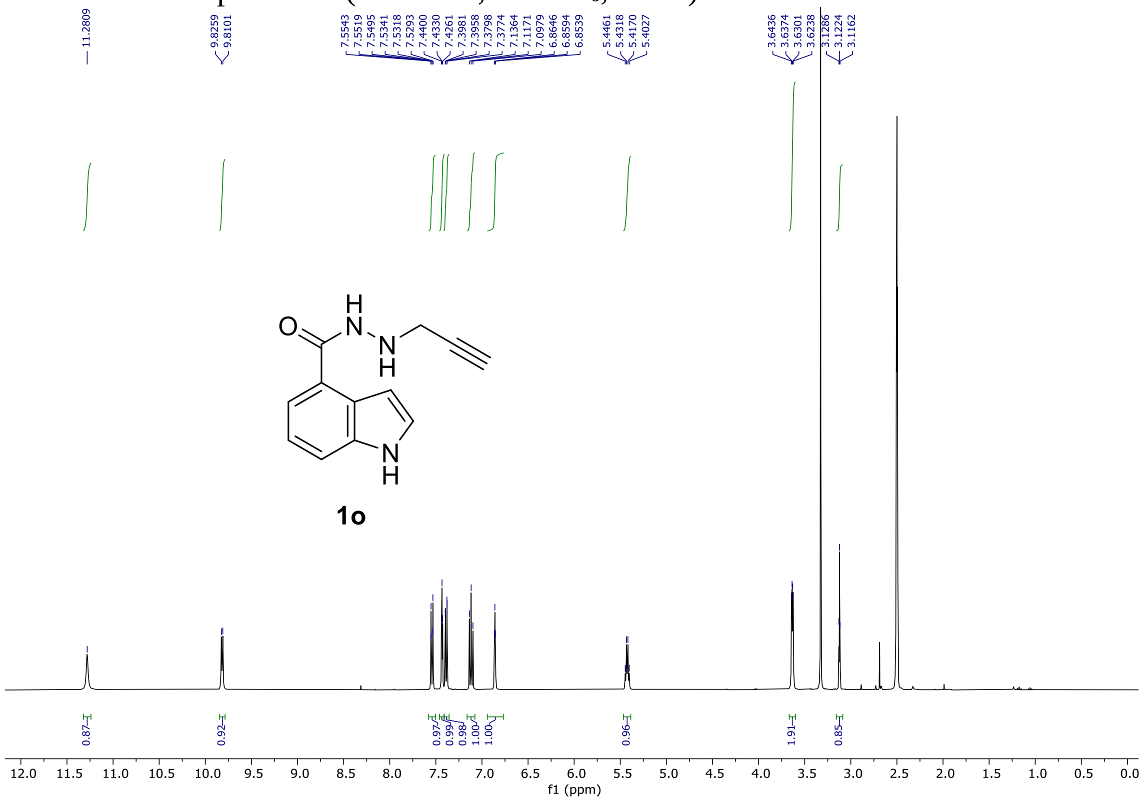

^{13}C NMR of compound **1o** (101 MHz, dmso-d_6, 25 ºC)

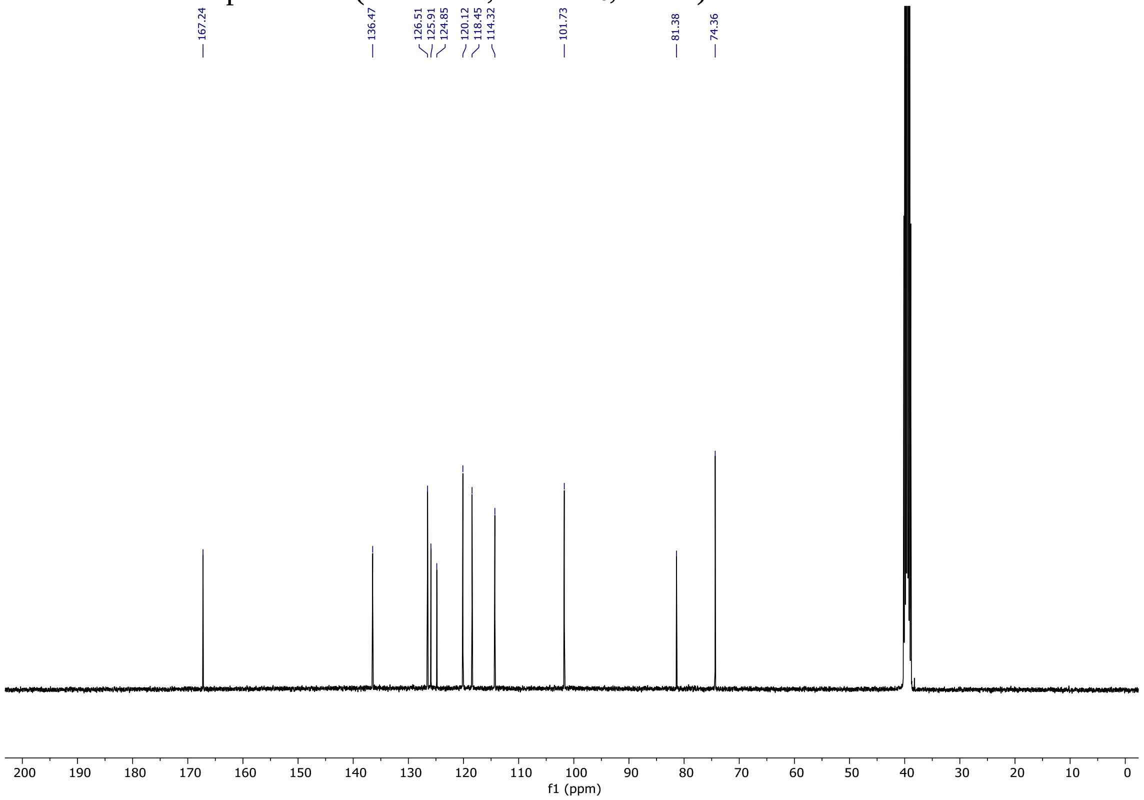

^{1}H NMR of compound **1p** (400 MHz, $CDCl_3$, 25 ºC)

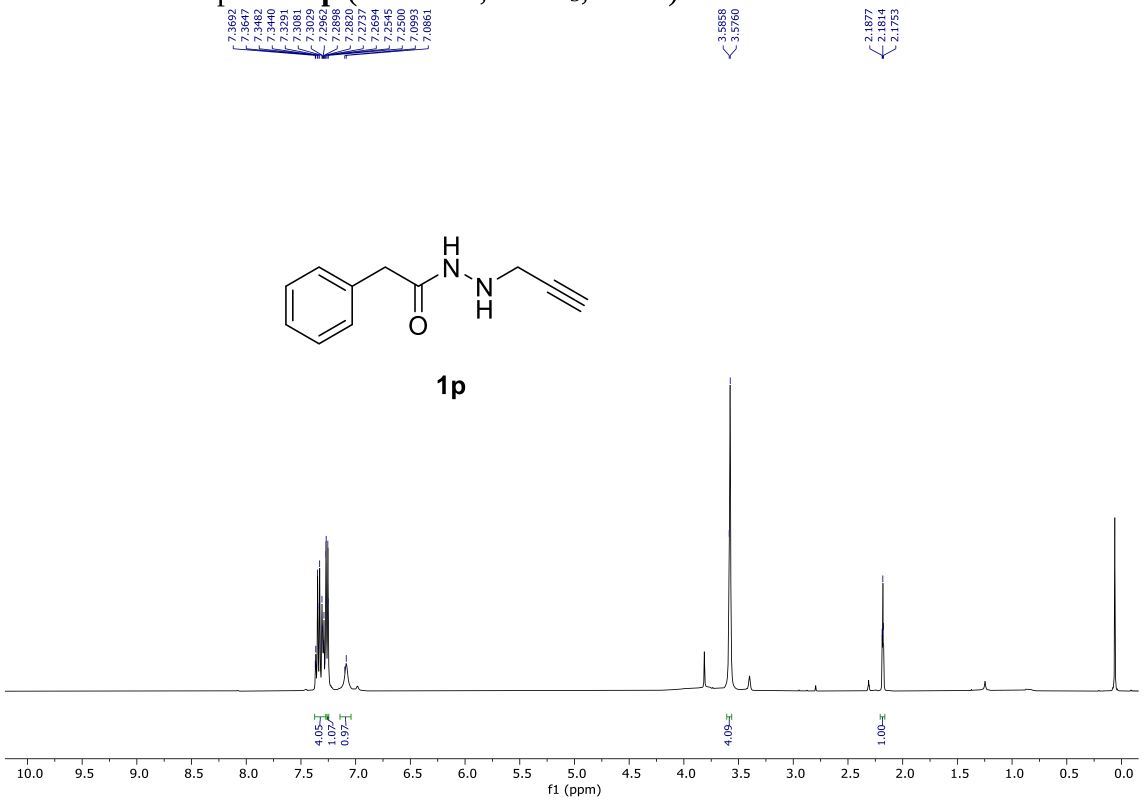

^{13}C NMR of compound **1p** (101 MHz, $CDCl_3$, 25 ºC)

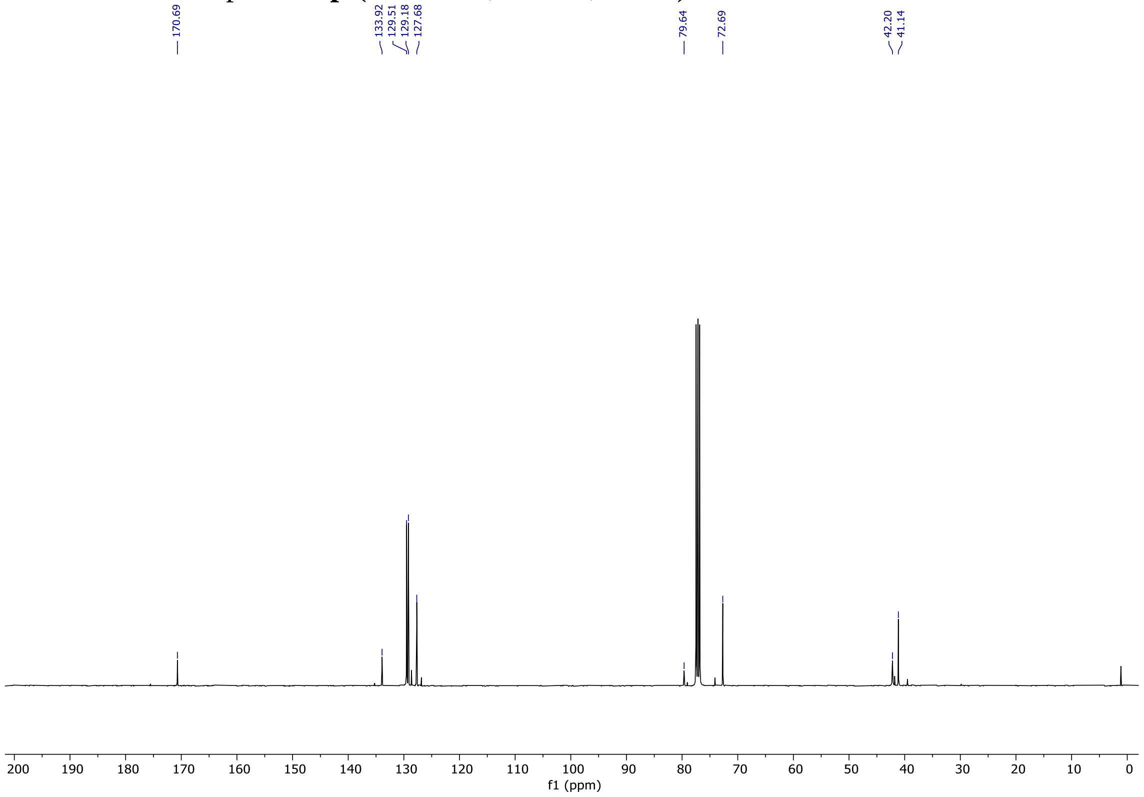

^{1}H NMR of compound **1q** (400 MHz, dmso-d_6, 25 ºC)

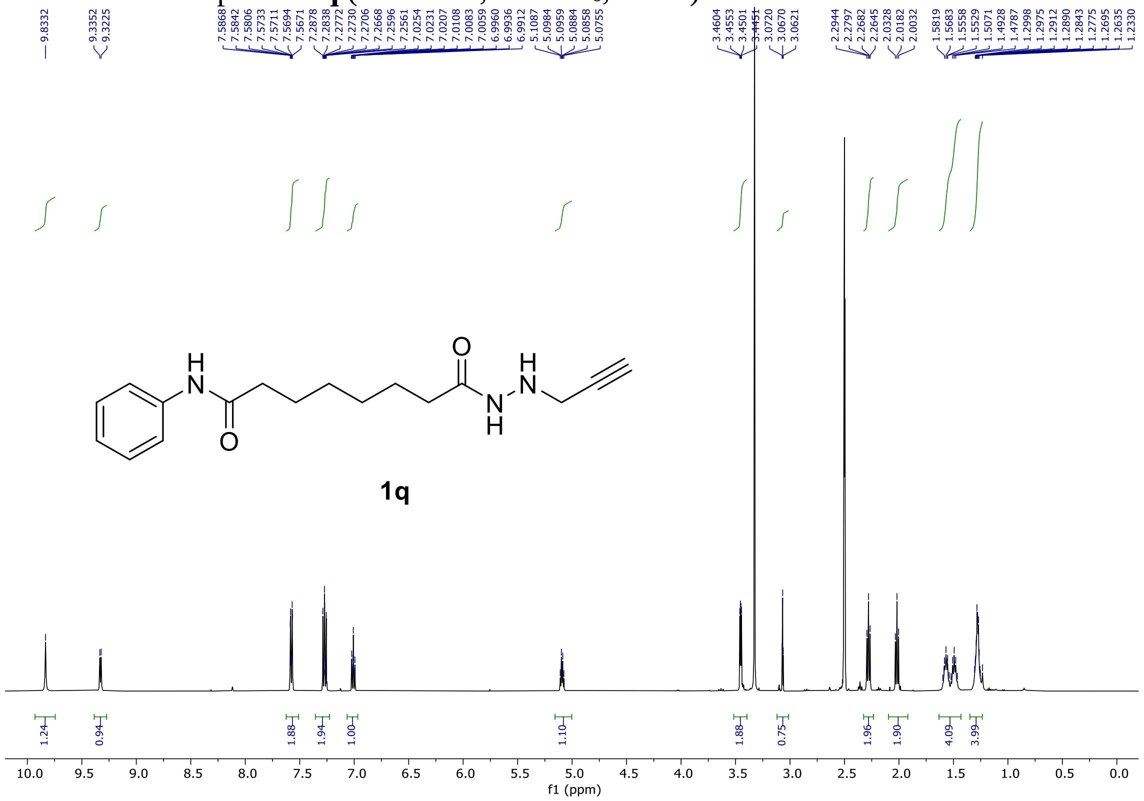

^{13}C NMR of compound **1q** (101 MHz, dmso-d_6, 25 ºC)

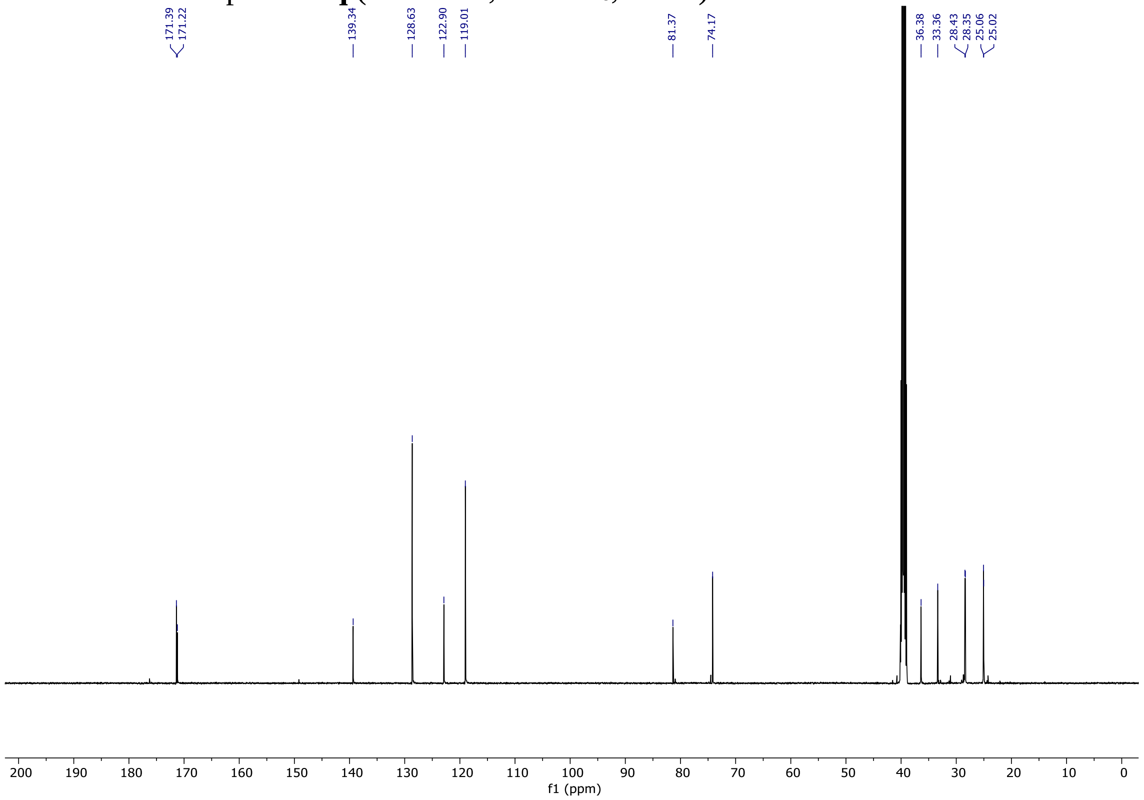

^{1}H NMR of compound **1a-PhOMe** (400 MHz, acetone-d_6, 25 ºC)

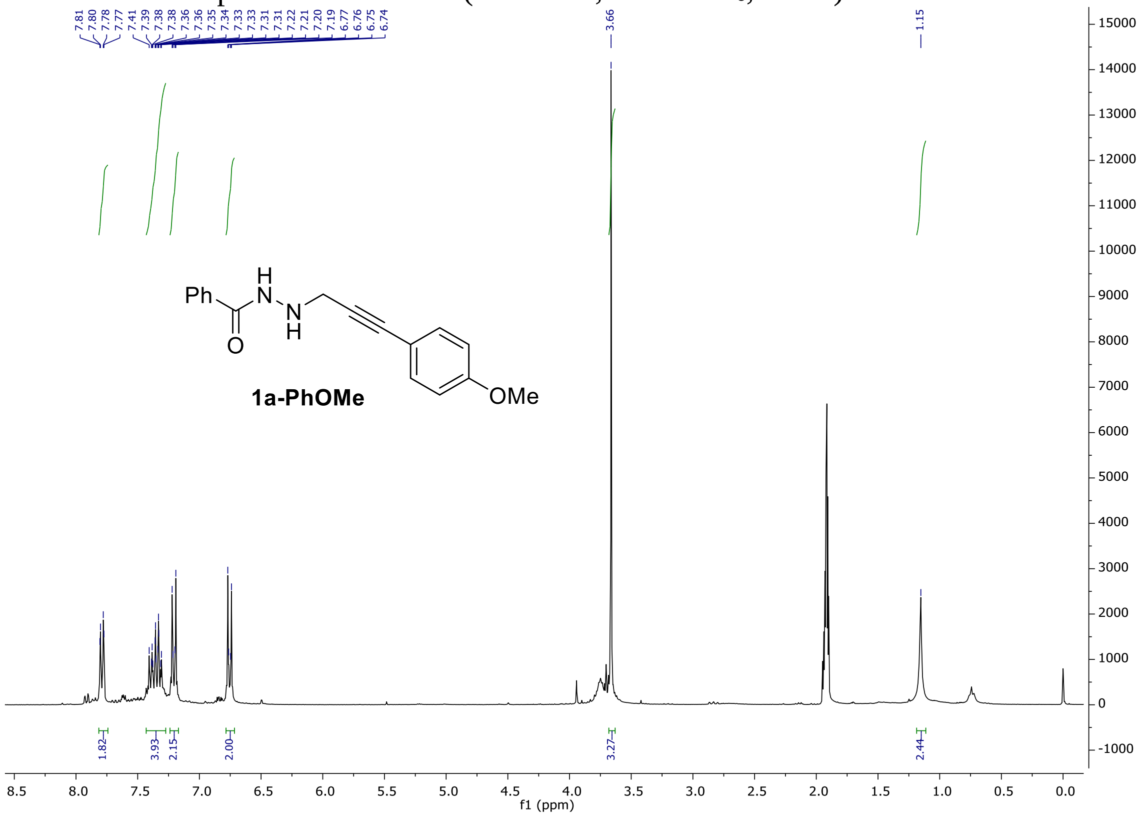

^{13}C NMR of compound **1a-PhOMe** (101 MHz, acetone-d_6, 25 ºC)

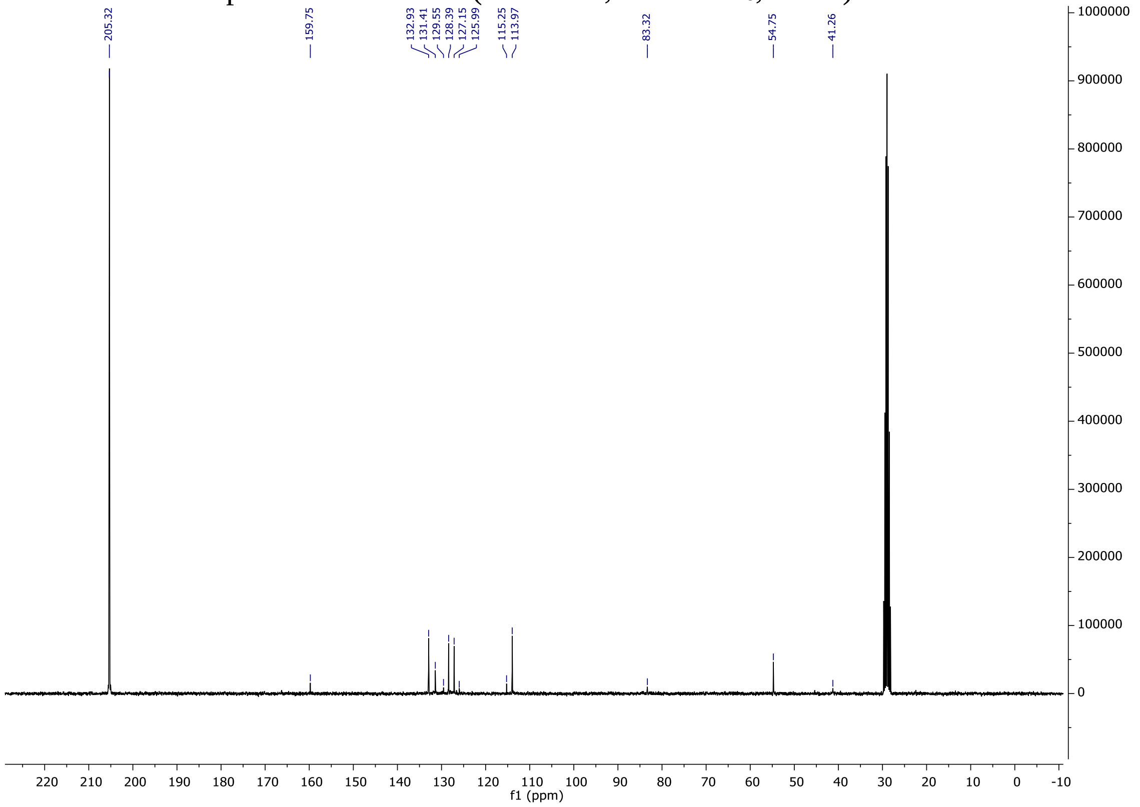

^{1}H NMR of compound **1a-PhNO$_2$** (300 MHz, $CDCl_3$, 25 ºC)

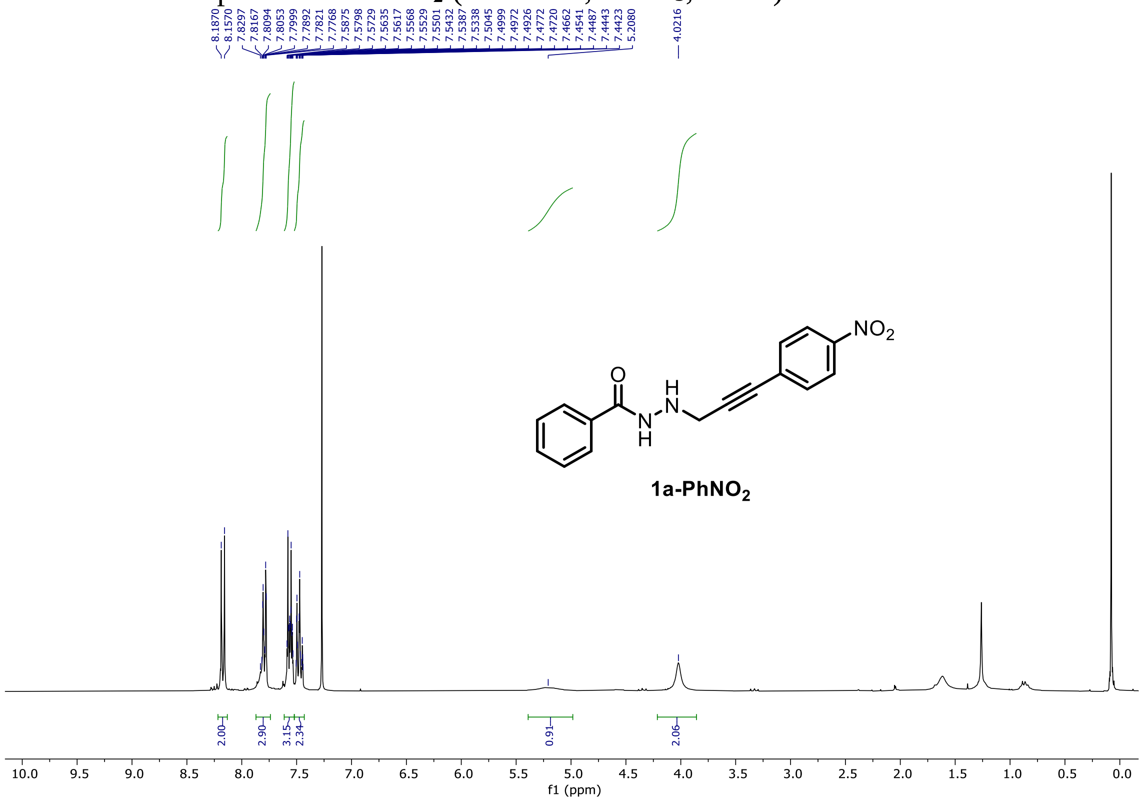

^{13}C NMR of compound **1a-PhNO$_2$** (101 MHz, $CDCl_3$, 25 ºC)

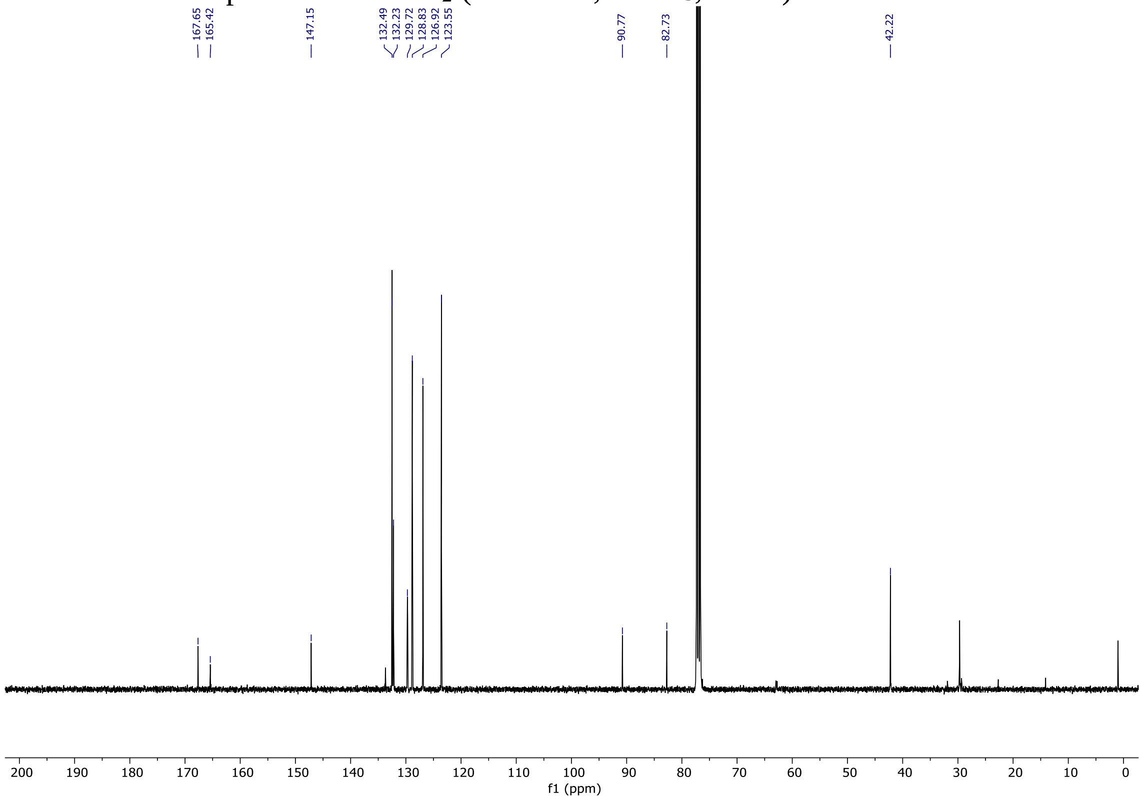

^{1}H NMR of compound **2a** (300 MHz, $CDCl_3$, 25 ºC)

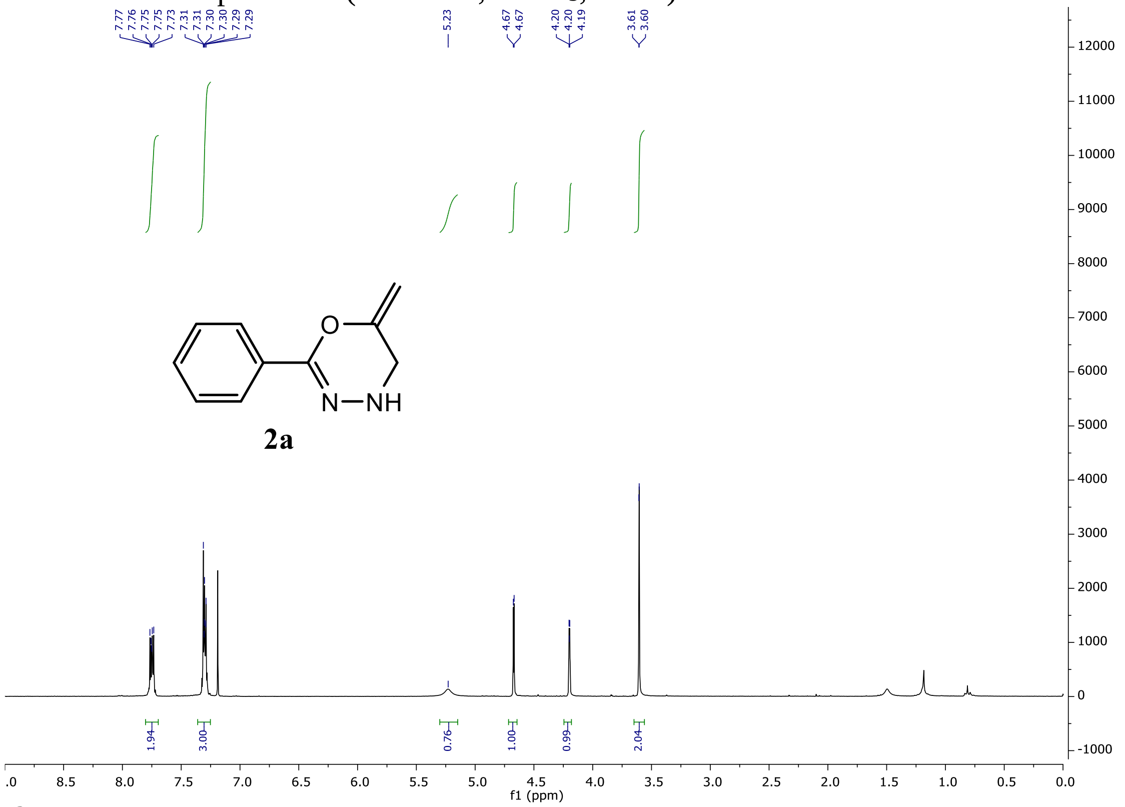

^{13}C NMR of compound **2a** (75 MHz, $CDCl_3$, 25 ºC)

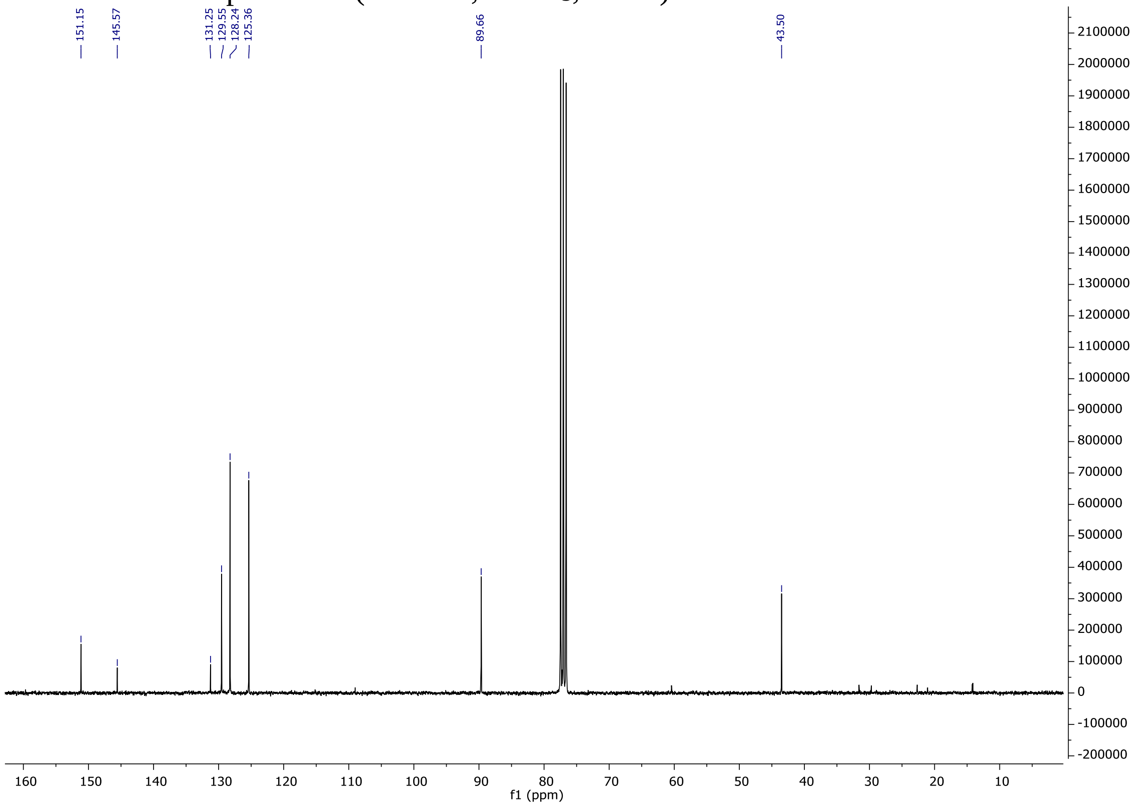

DEPT of compound **2a** (75 MHz, $CDCl_3$, 25 ºC)

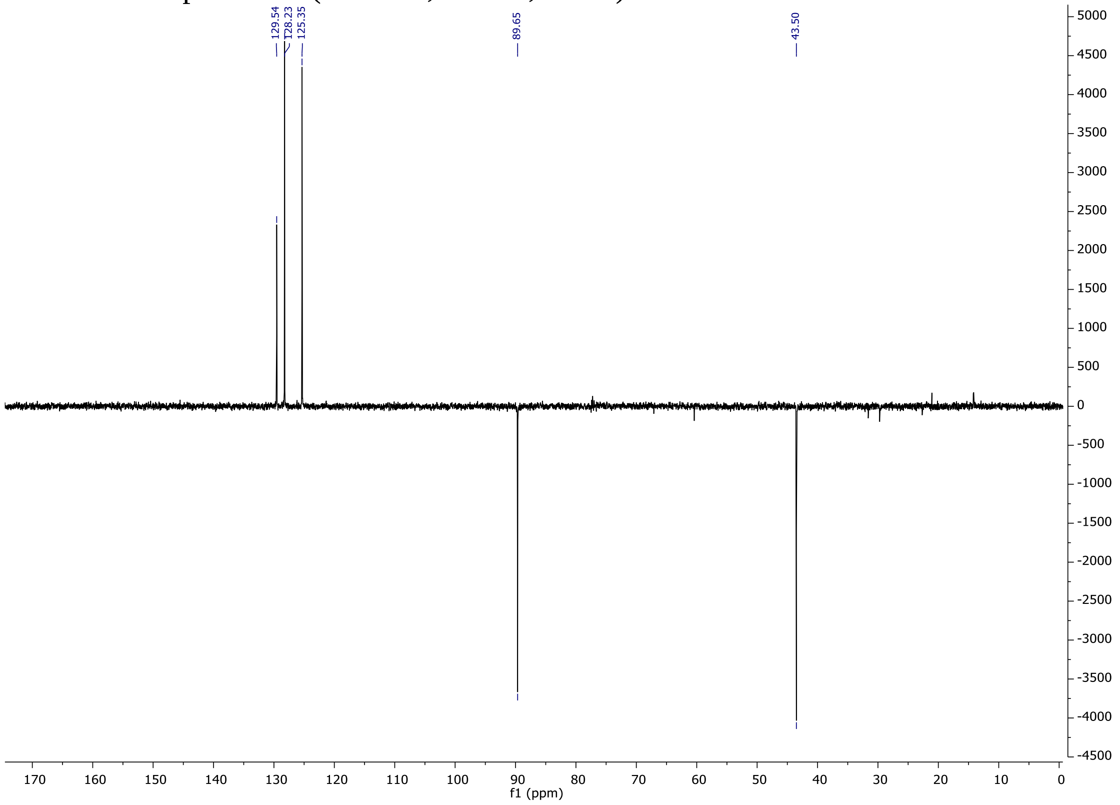

^{1}H NMR of compound **2b** (600 MHz, $CDCl_3$, 25 ºC)

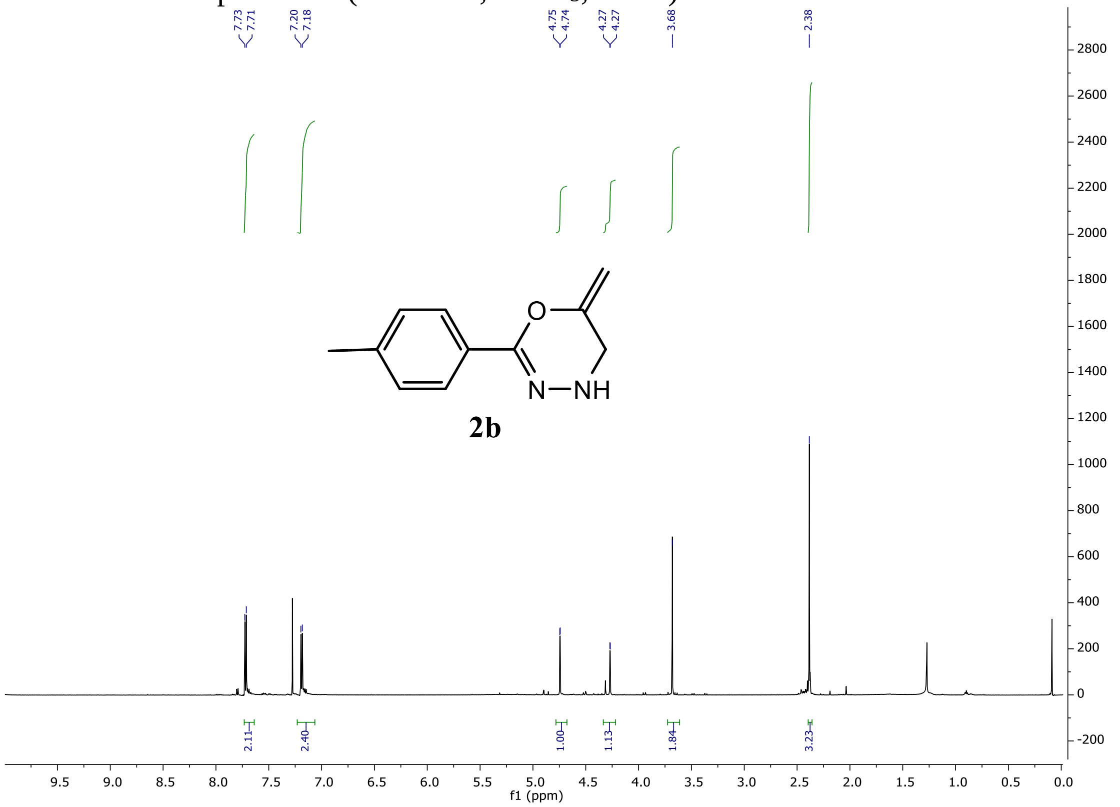

^{13}C NMR of compound **2b** (151 MHz, $CDCl_3$, 25 ºC)

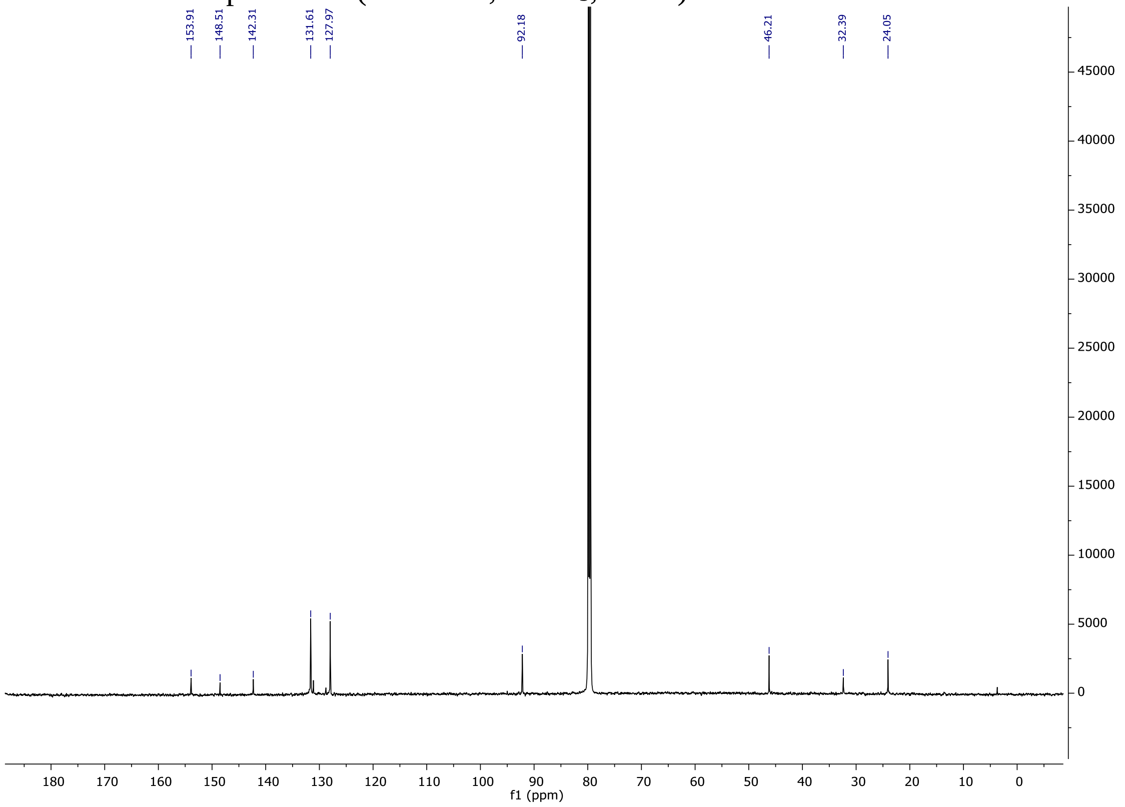

HSQC of compound **2b**

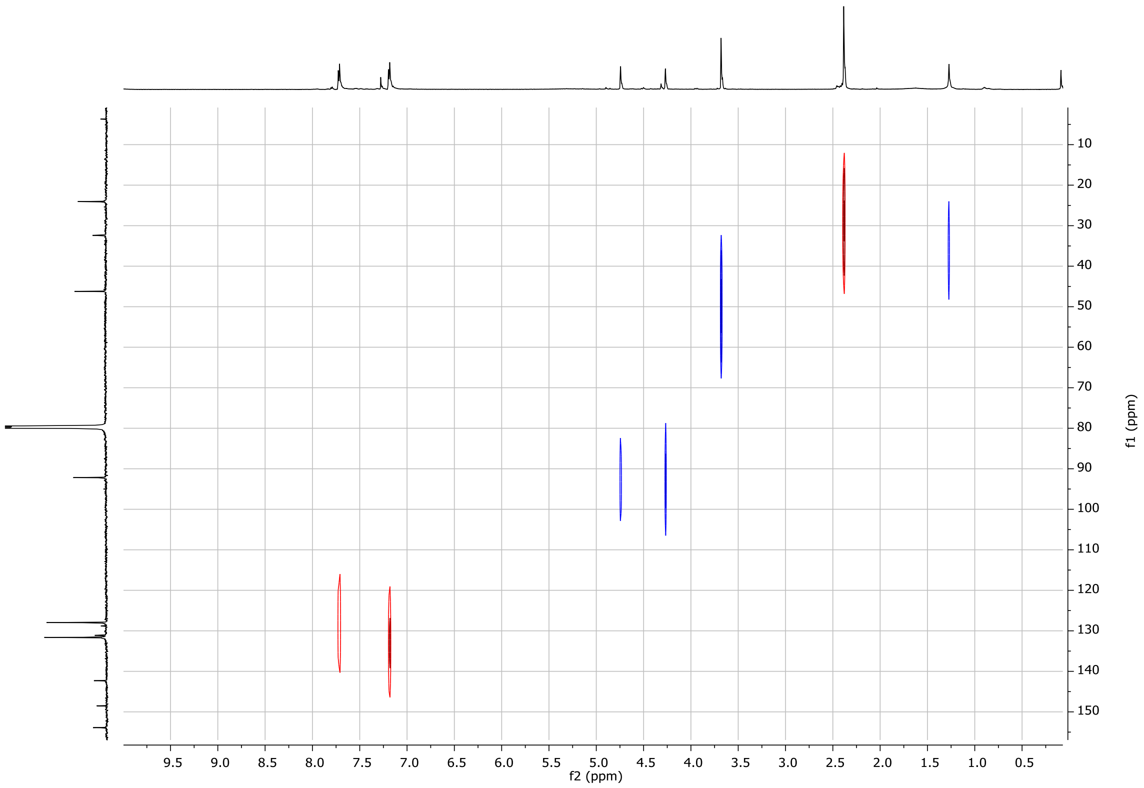

HMBC of compound **2b**

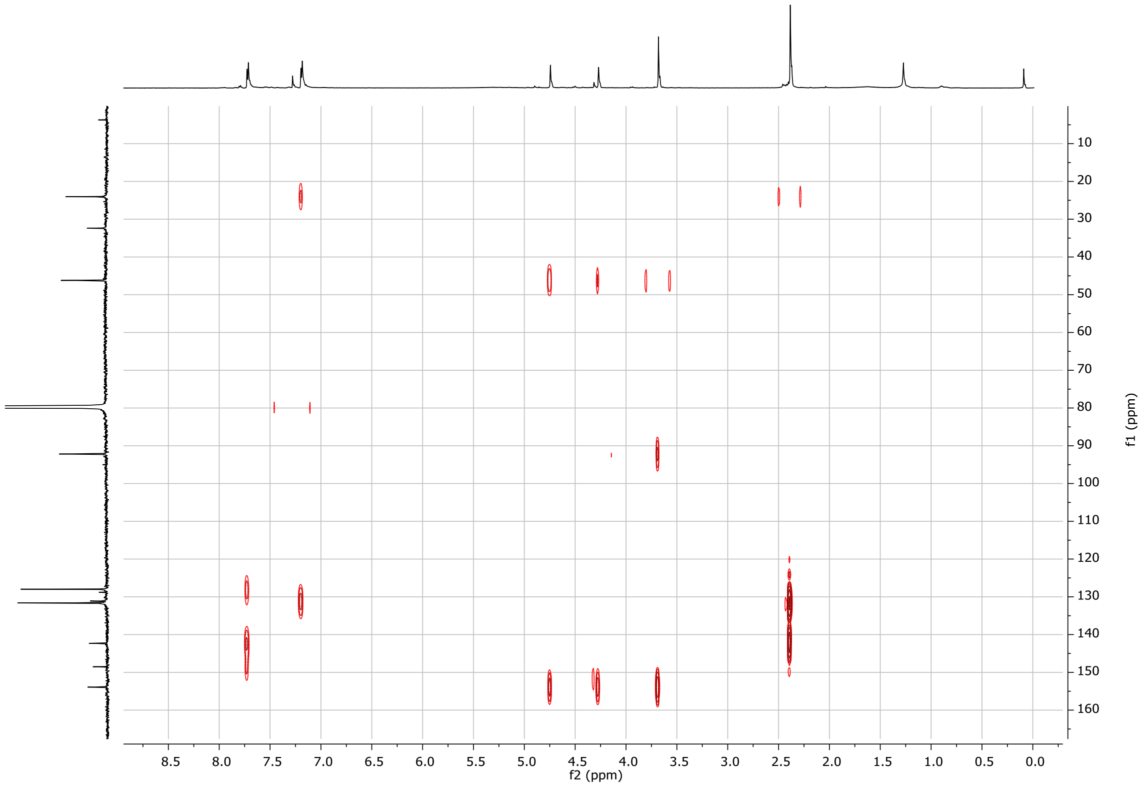

^{1}H NMR of compound **2d** (600 MHz, $CDCl_3$, 25 ºC)

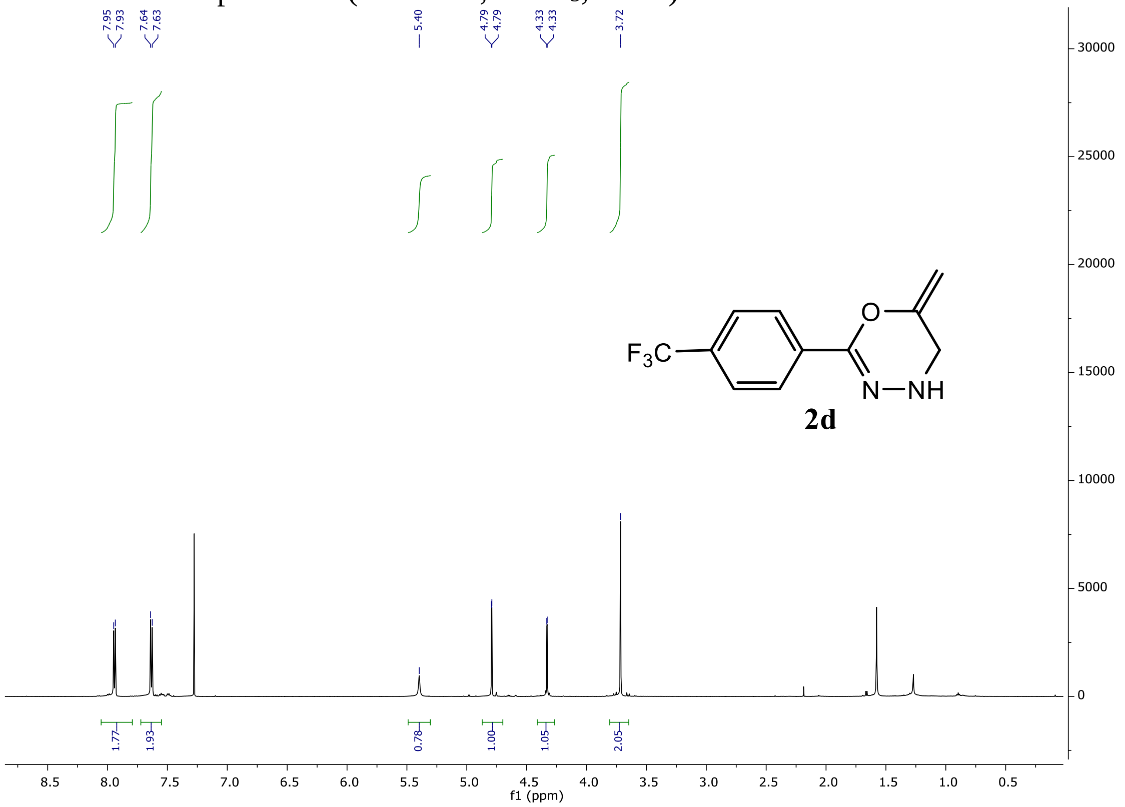

^{13}C NMR of compound **2d** (151 MHz, $CDCl_3$, 25 ºC)

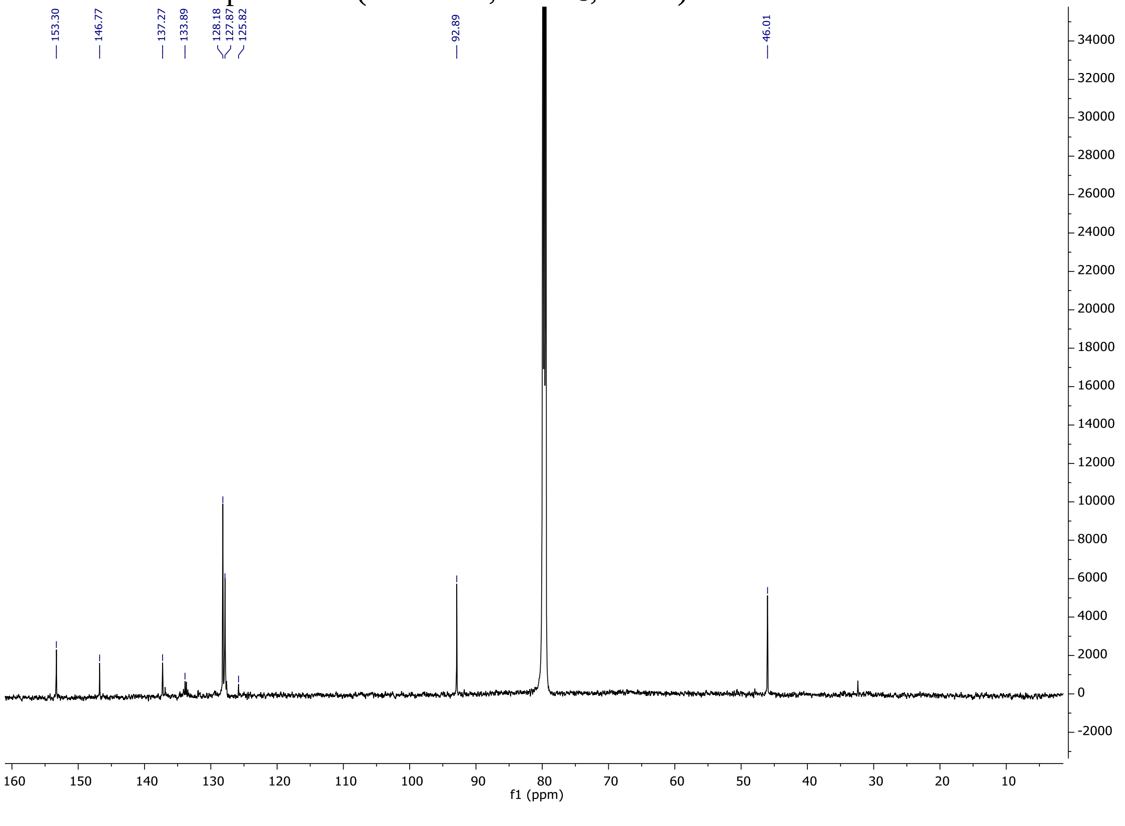

HSQC of compound **2d**

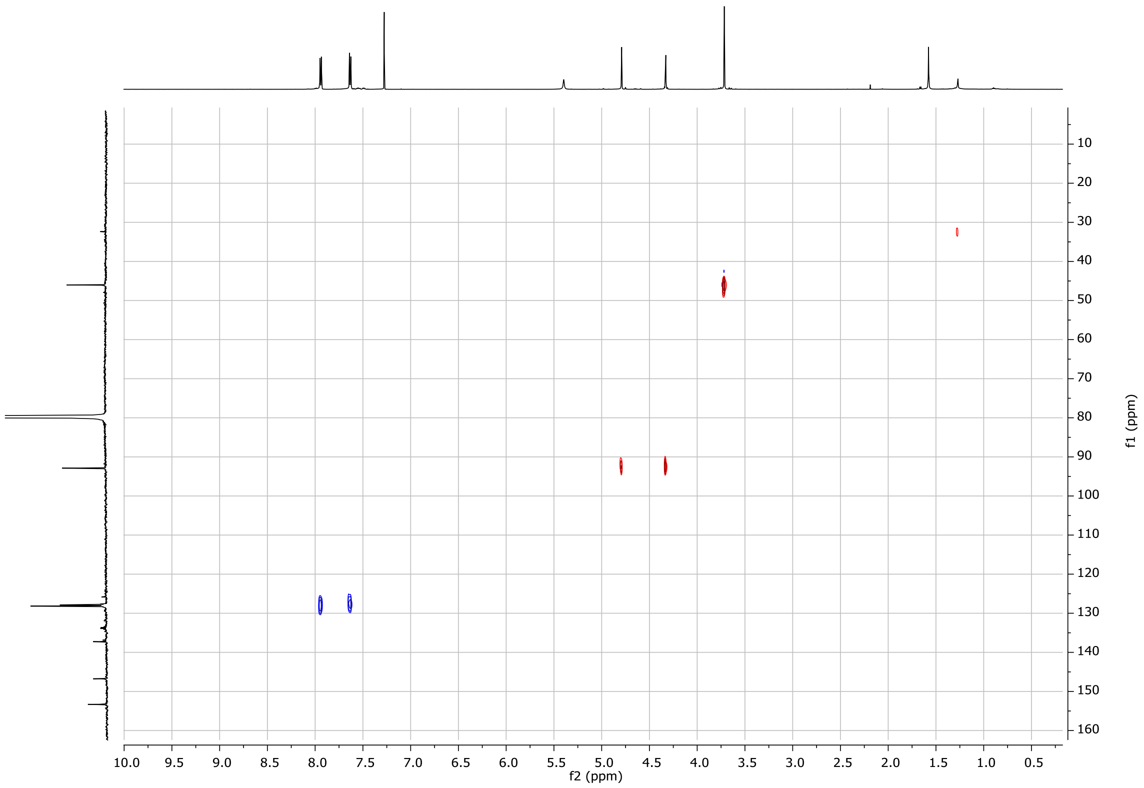

HMBC of compound **2d**

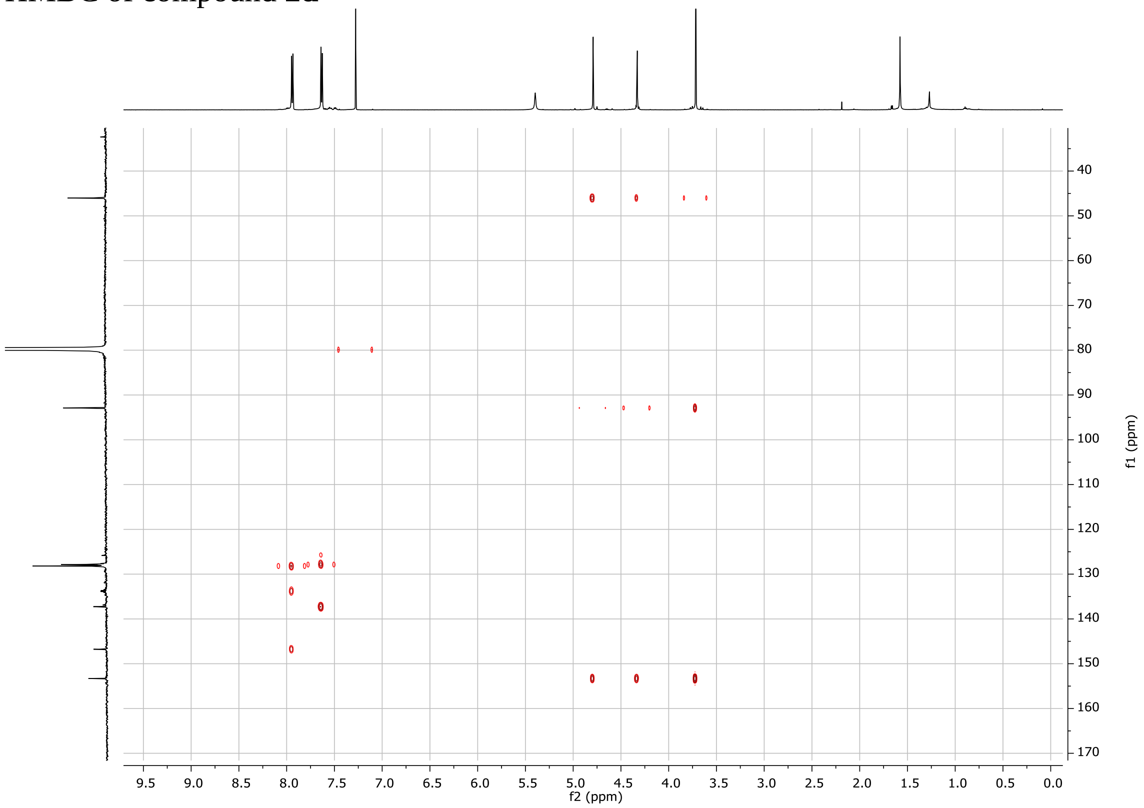

^{1}H NMR of compound **2e** (600 MHz, $CDCl_3$, 25 ºC)

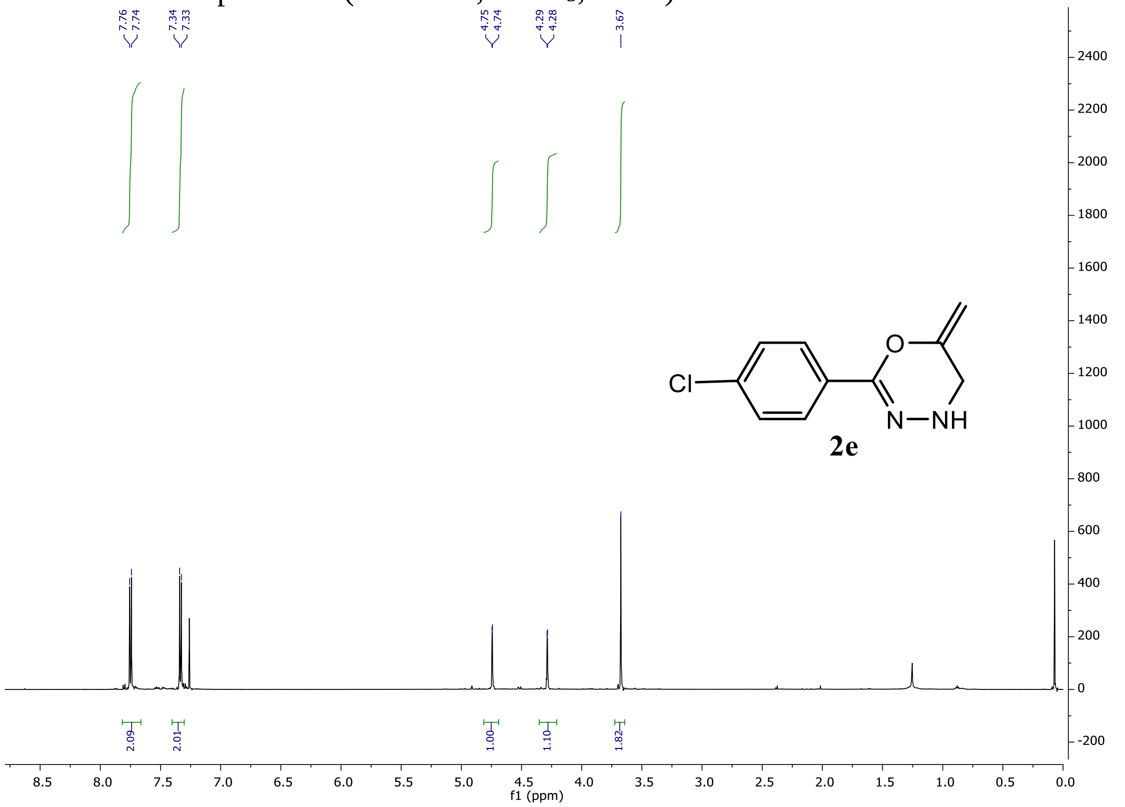

^{13}C NMR of compound **2e** (151 MHz, $CDCl_3$, 25 ºC)

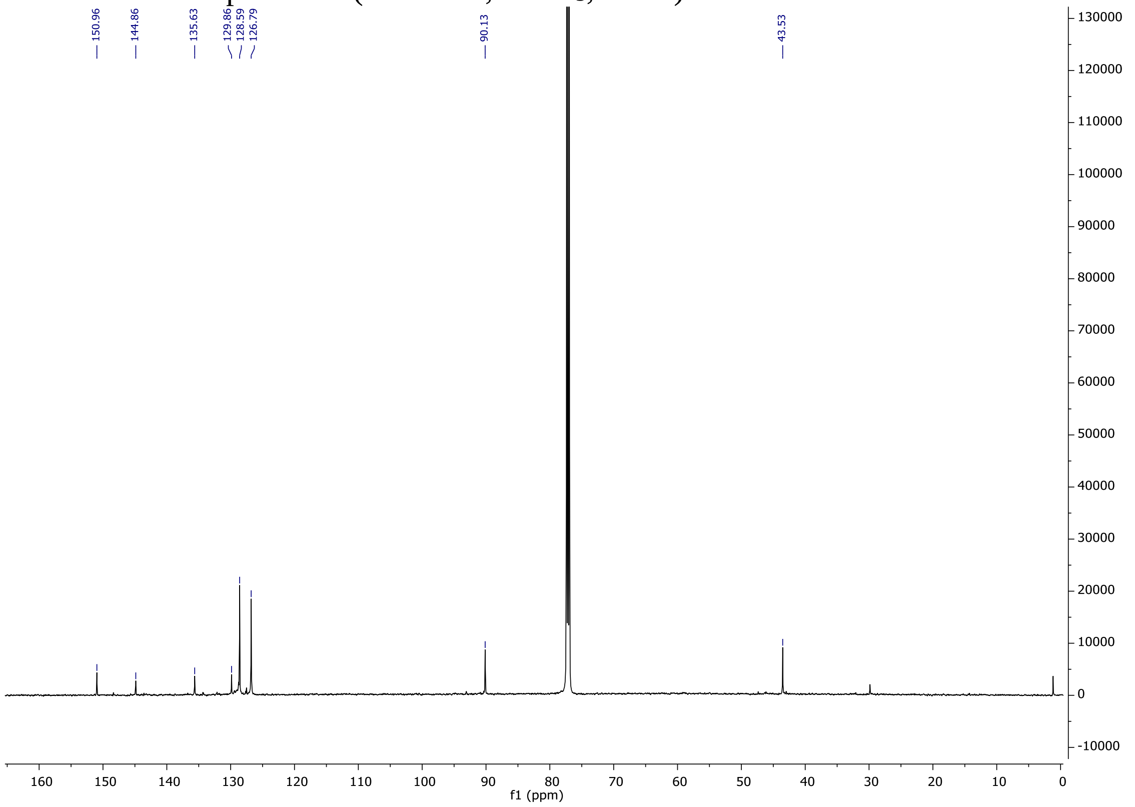

HSQC of compound **2e**

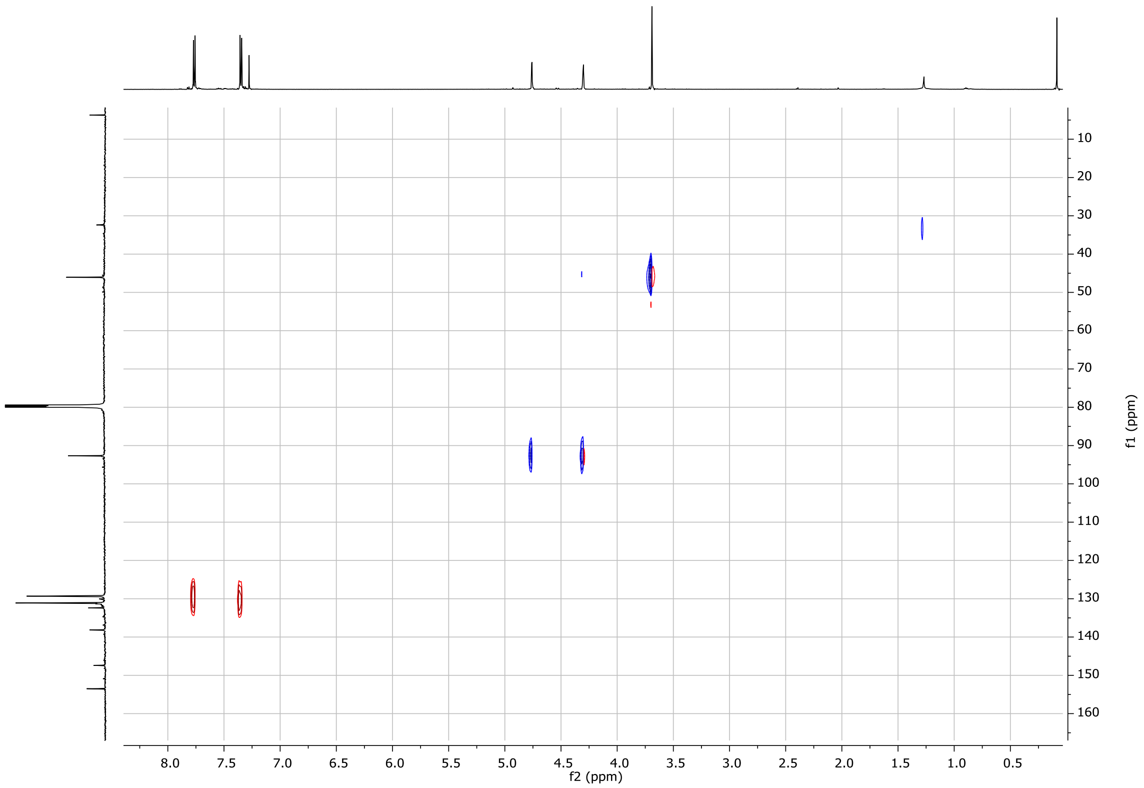

HMBC of compound **2e**

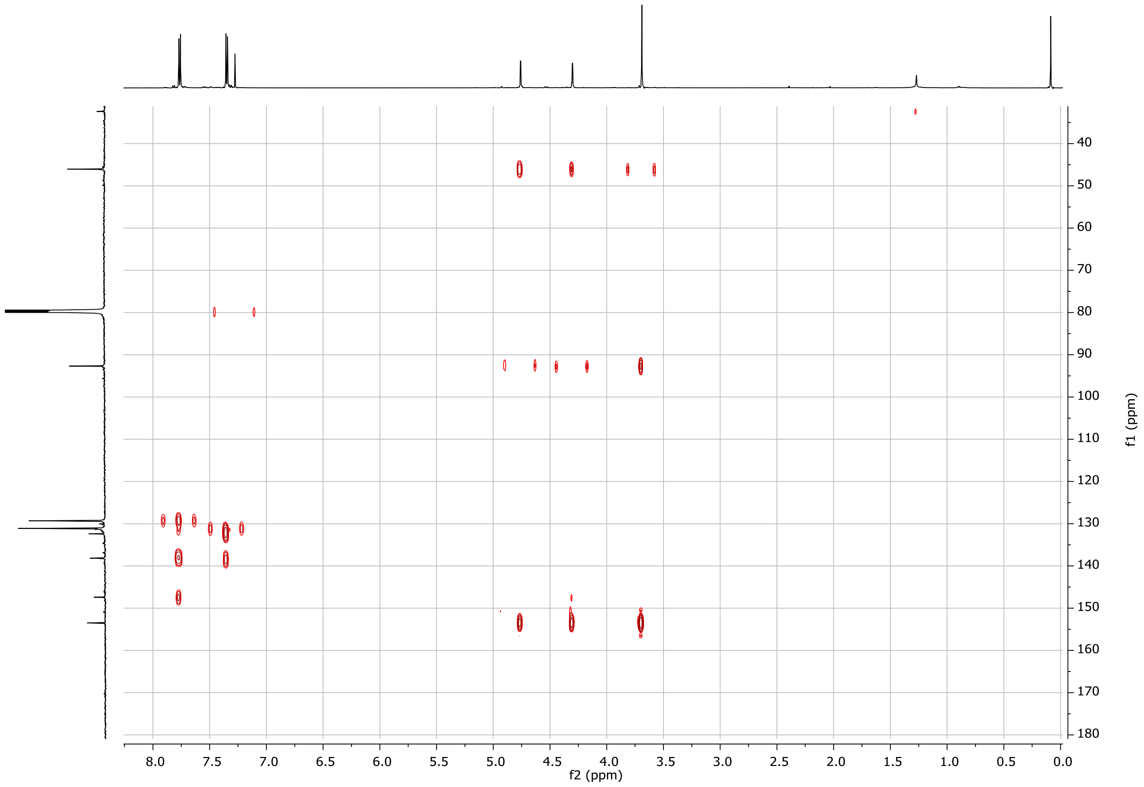

^{1}H NMR of compound **2g** (600 MHz, $CDCl_3$, 25 ºC)

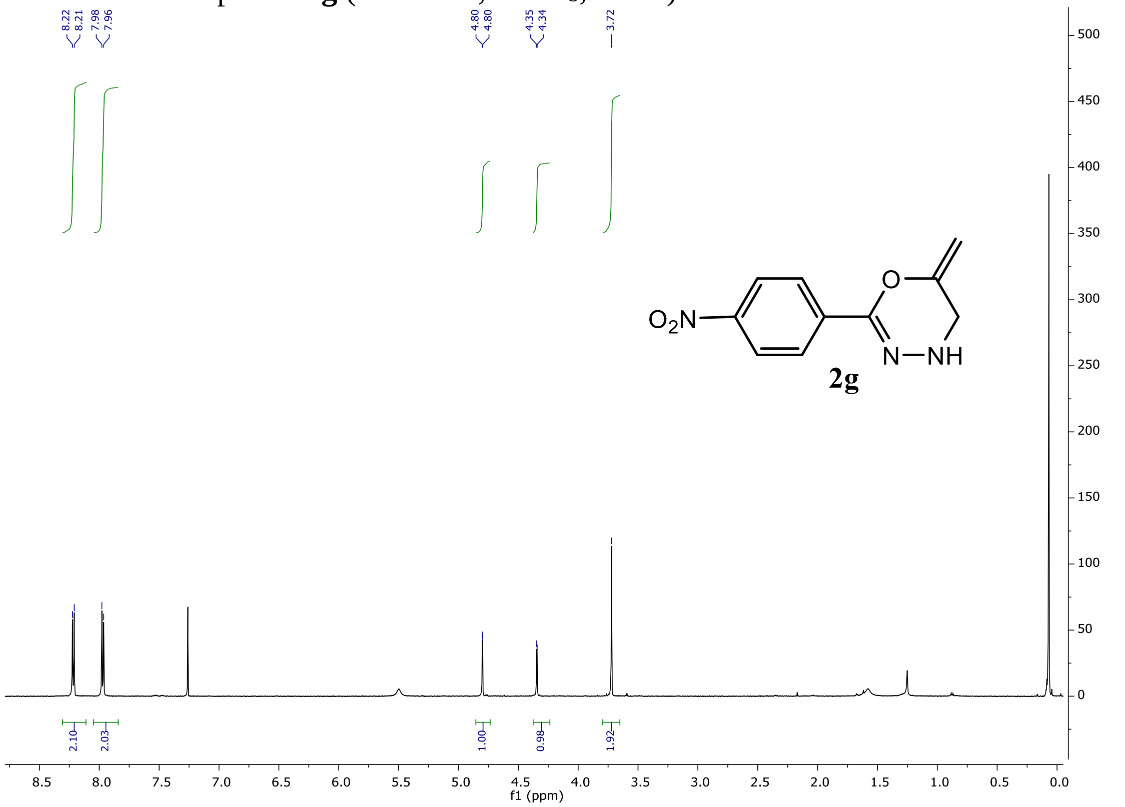

^{13}C NMR of compound **2g** (151 MHz, $CDCl_3$, 25 ºC)

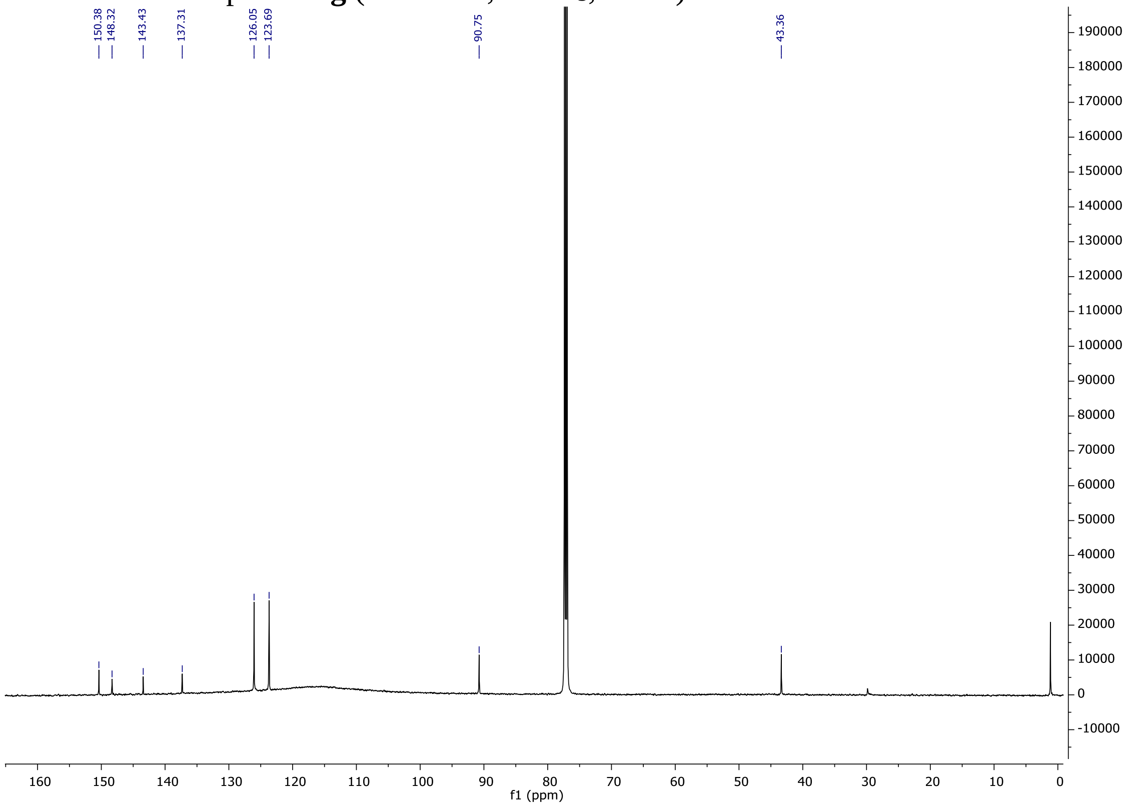

HSQC of compound **2g**

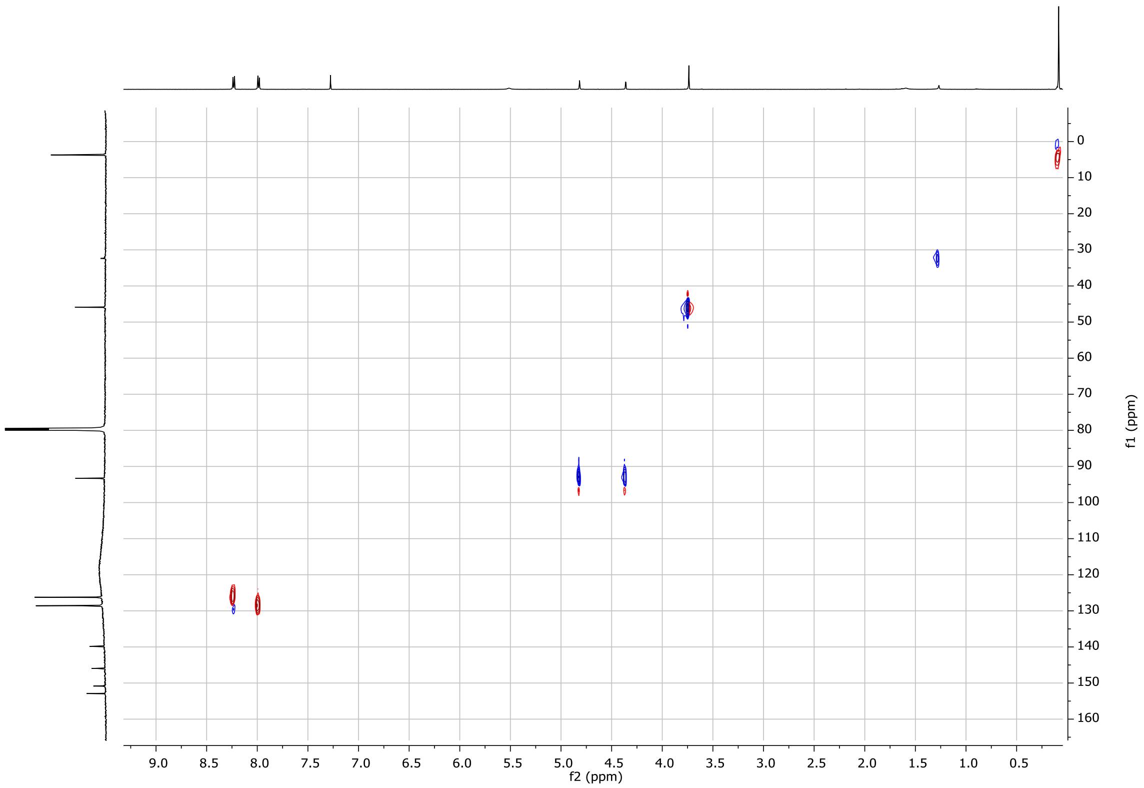

HMBC of compound **2g**

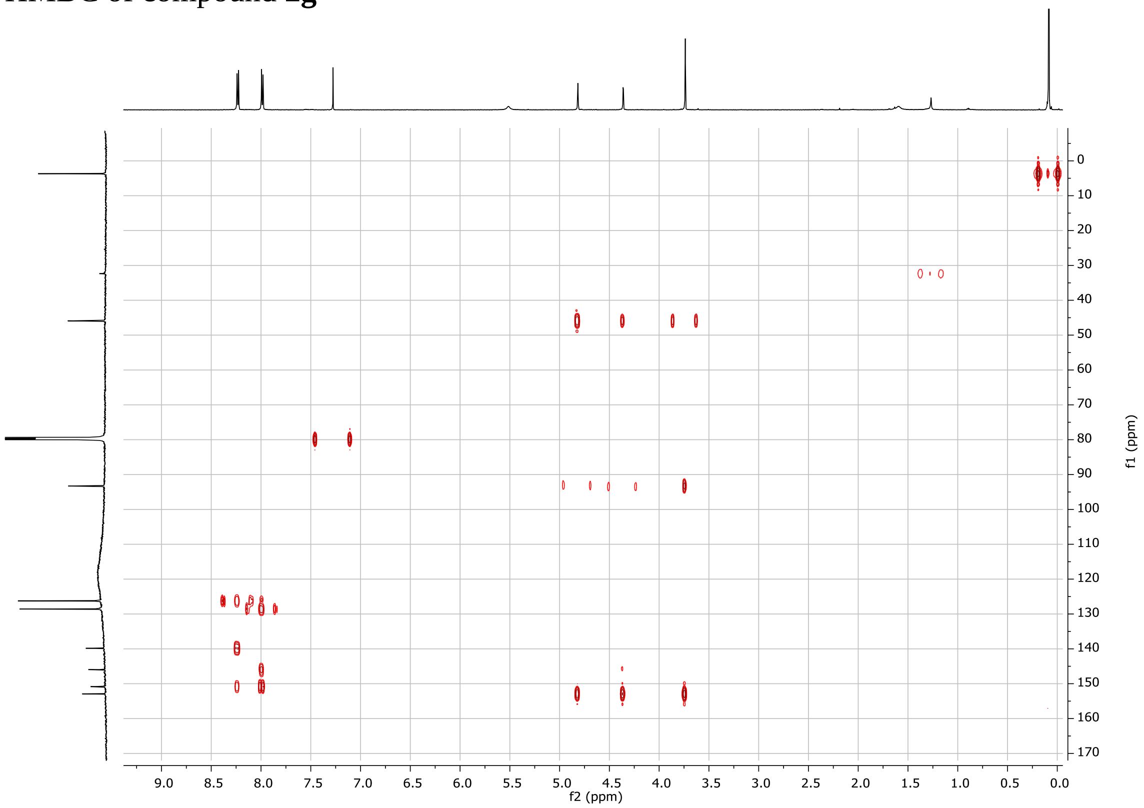

^{1}H NMR of compound **2h** (600 MHz, $CDCl_3$, 25 ºC)

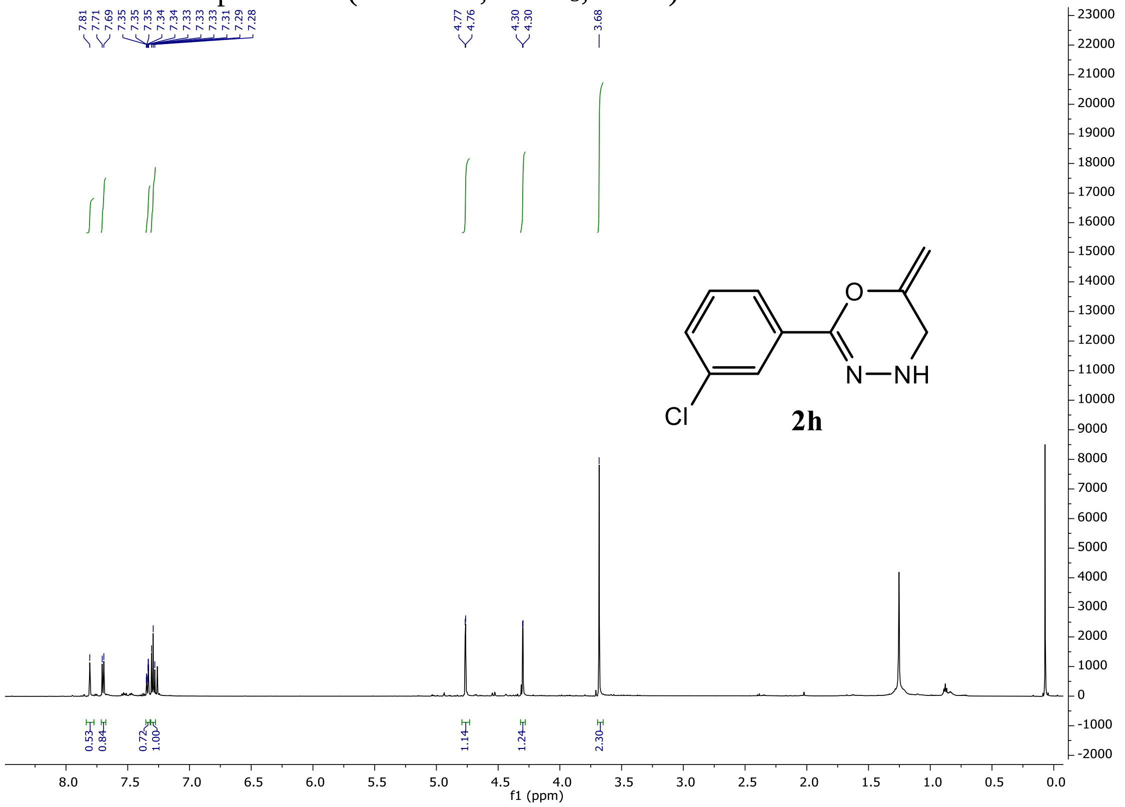

^{1}H NMR of compound **2h** (600 MHz, $CDCl_3$, 25 ºC) shows decomposition without base

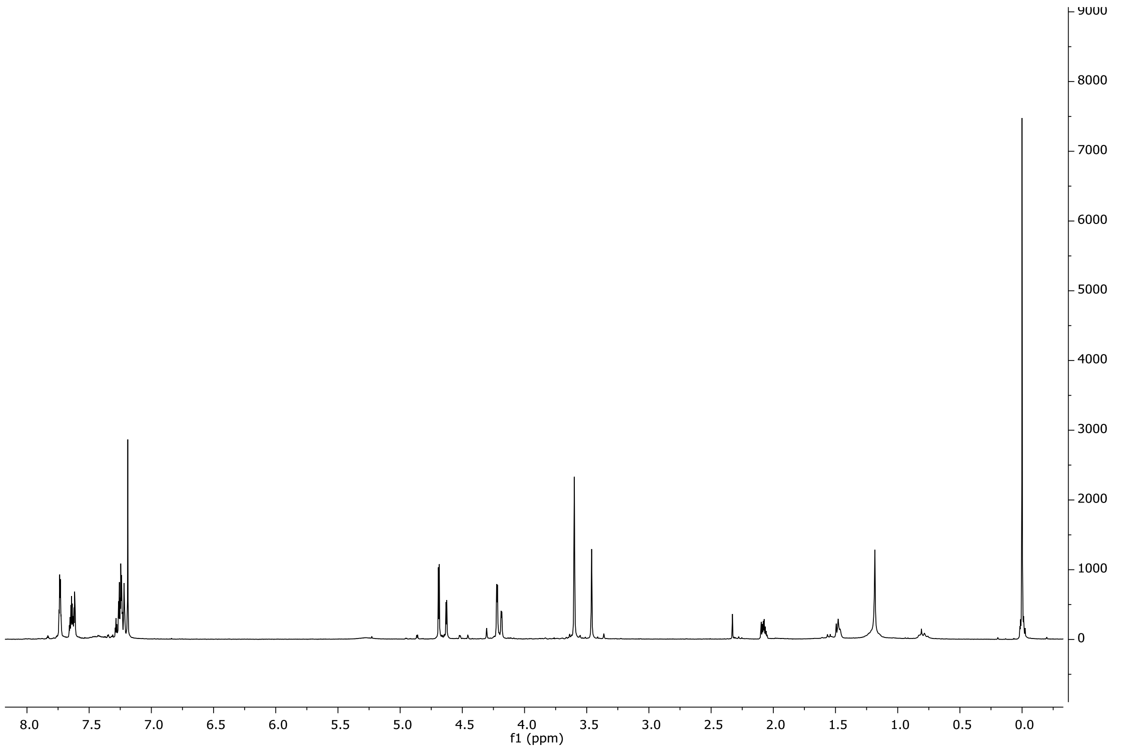

^{13}C NMR of compound **2h** (151 MHz, $CDCl_3$, 25 ºC)

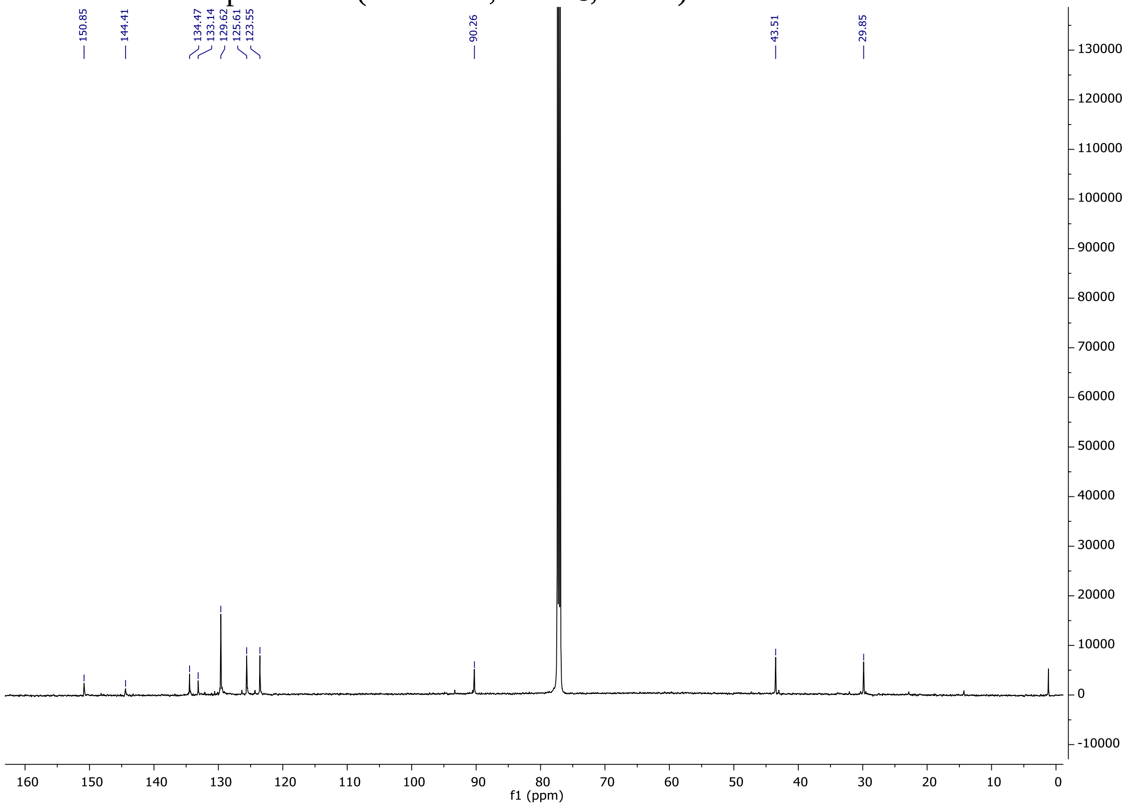

HSQC of compound **2h**

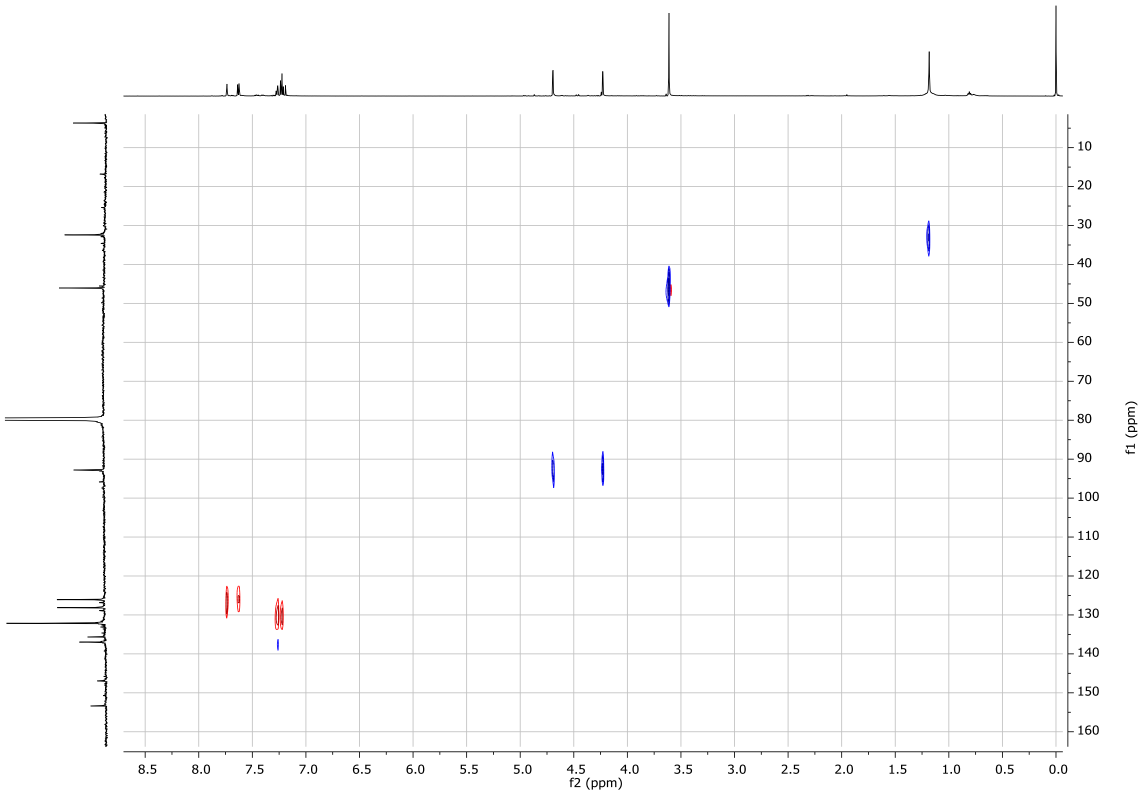

HMBC of compound **2h**

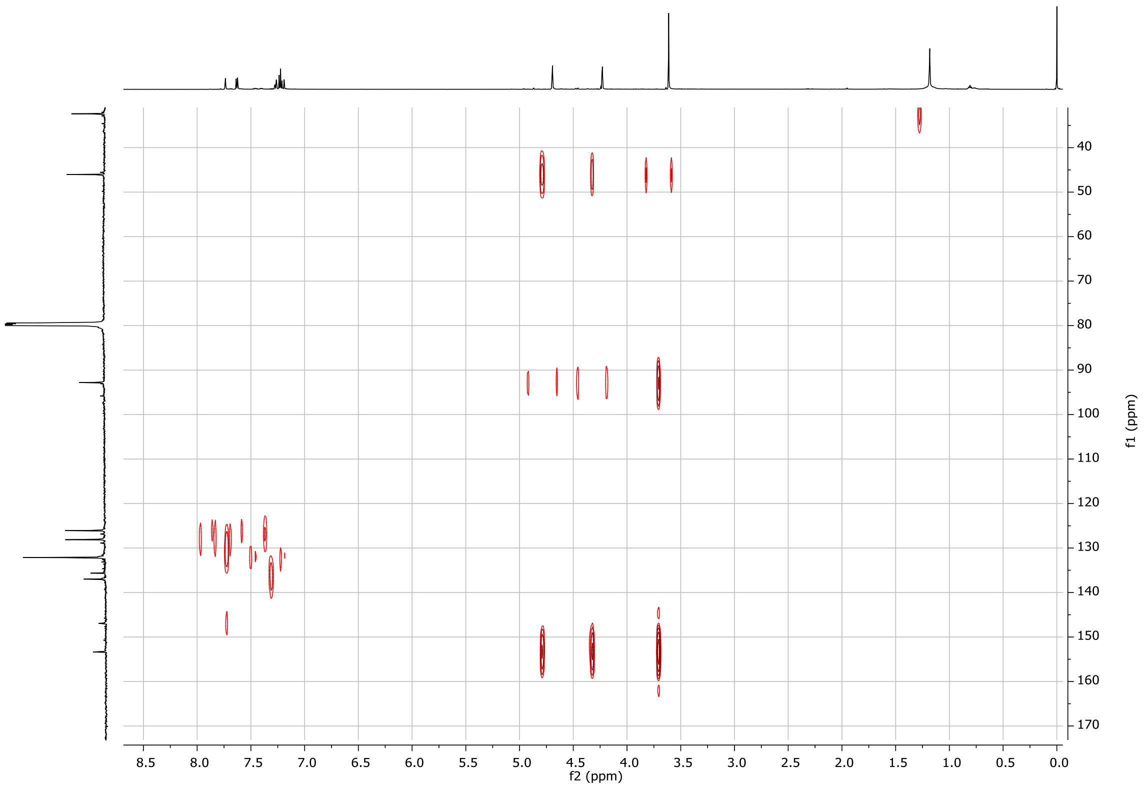

^{1}H NMR of compound **2i** (600 MHz, $CDCl_3$, 25 ºC)

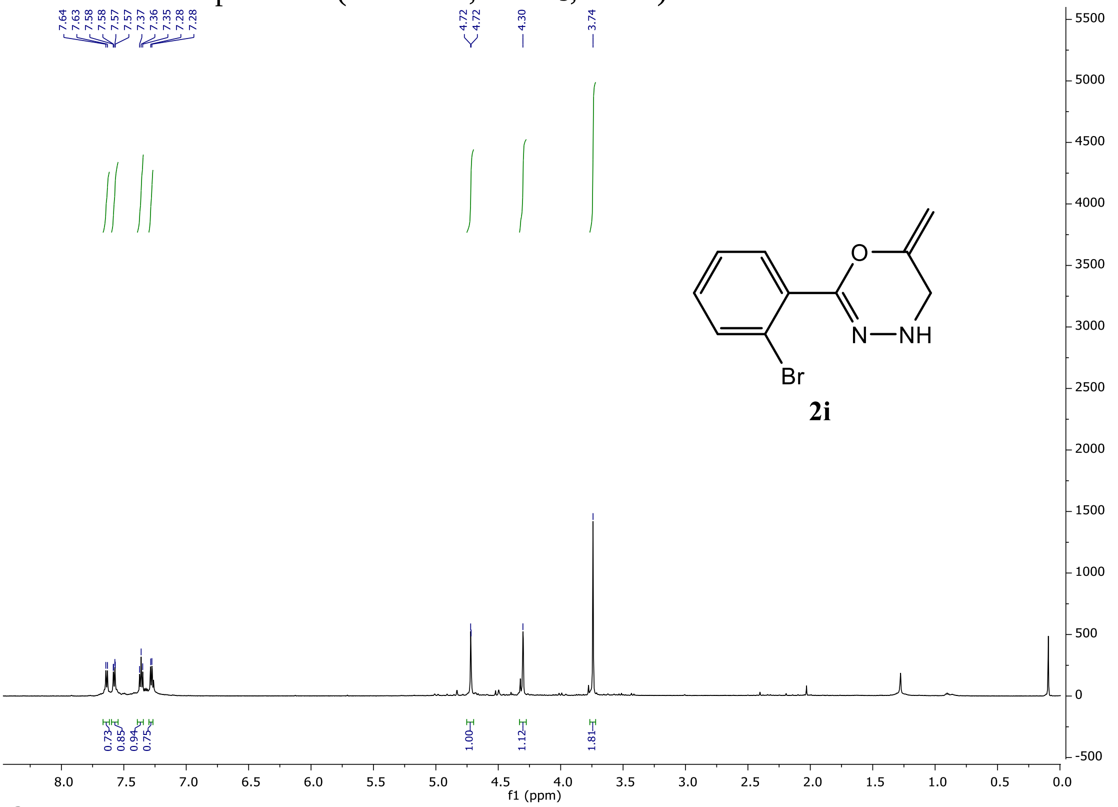

^{13}C NMR of compound **2i** (151 MHz, $CDCl_3$, 25 ºC)

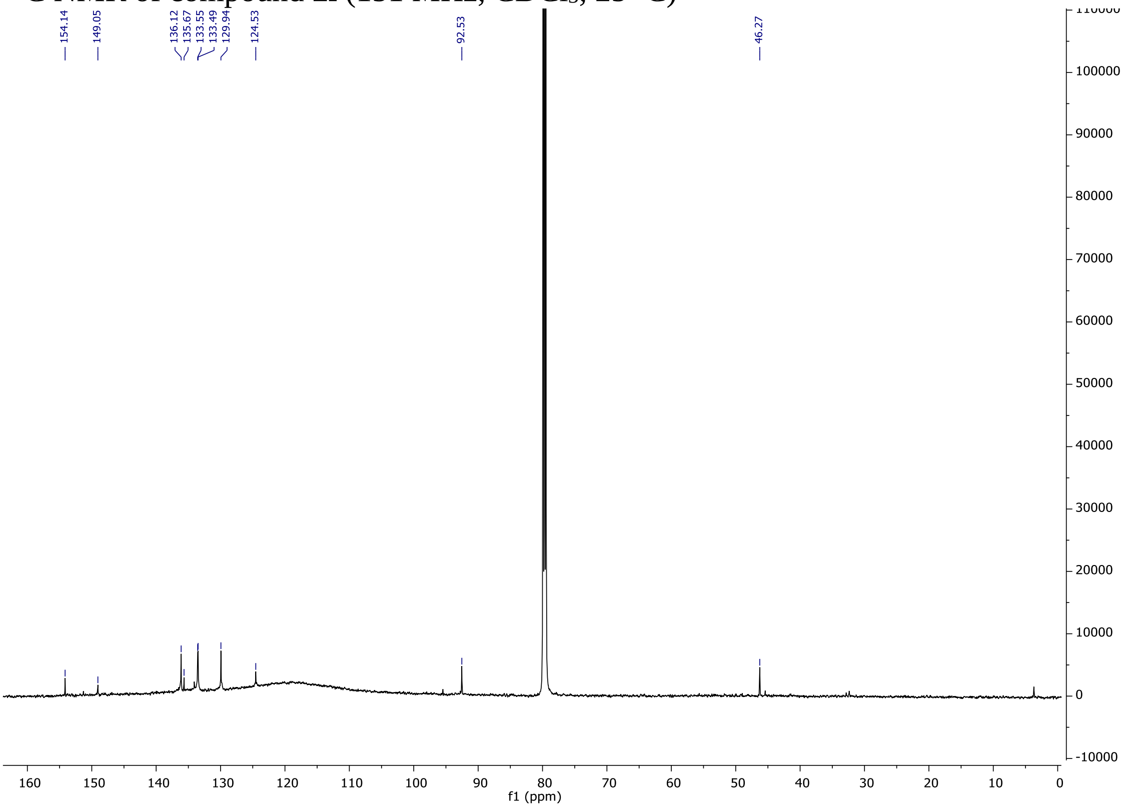

HSQC of compound **2i**

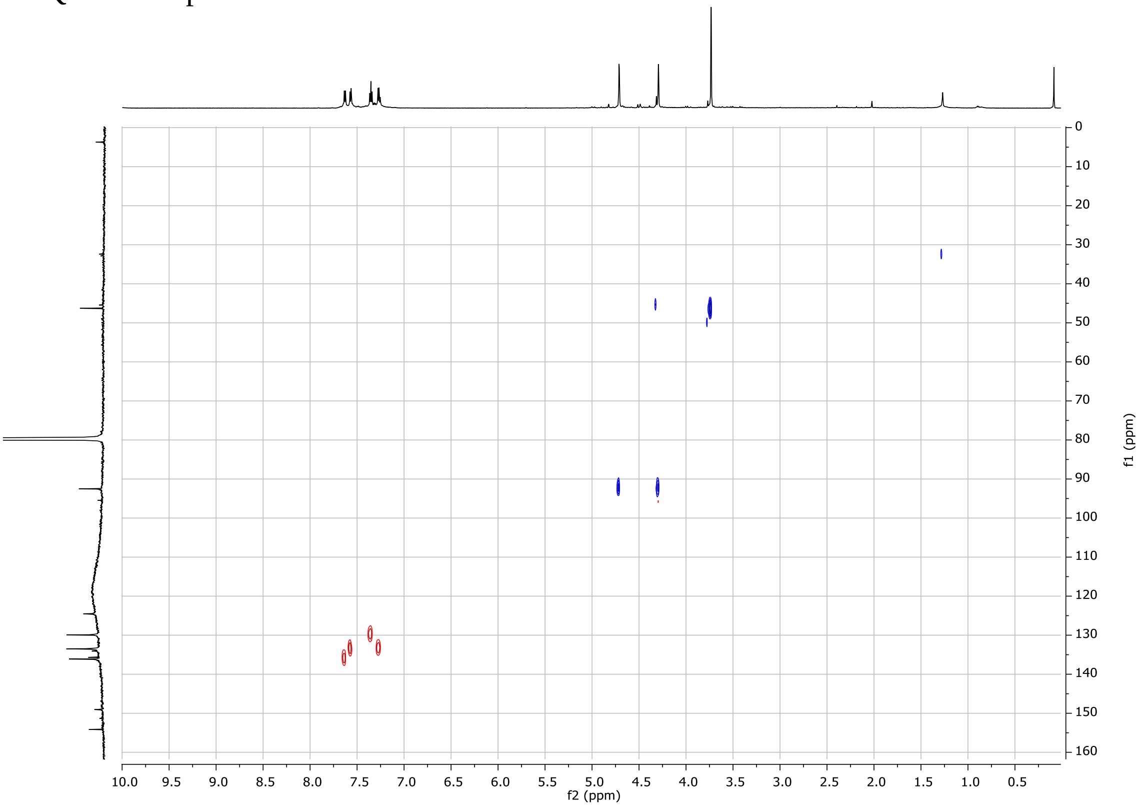

HMBC of compound **2i**

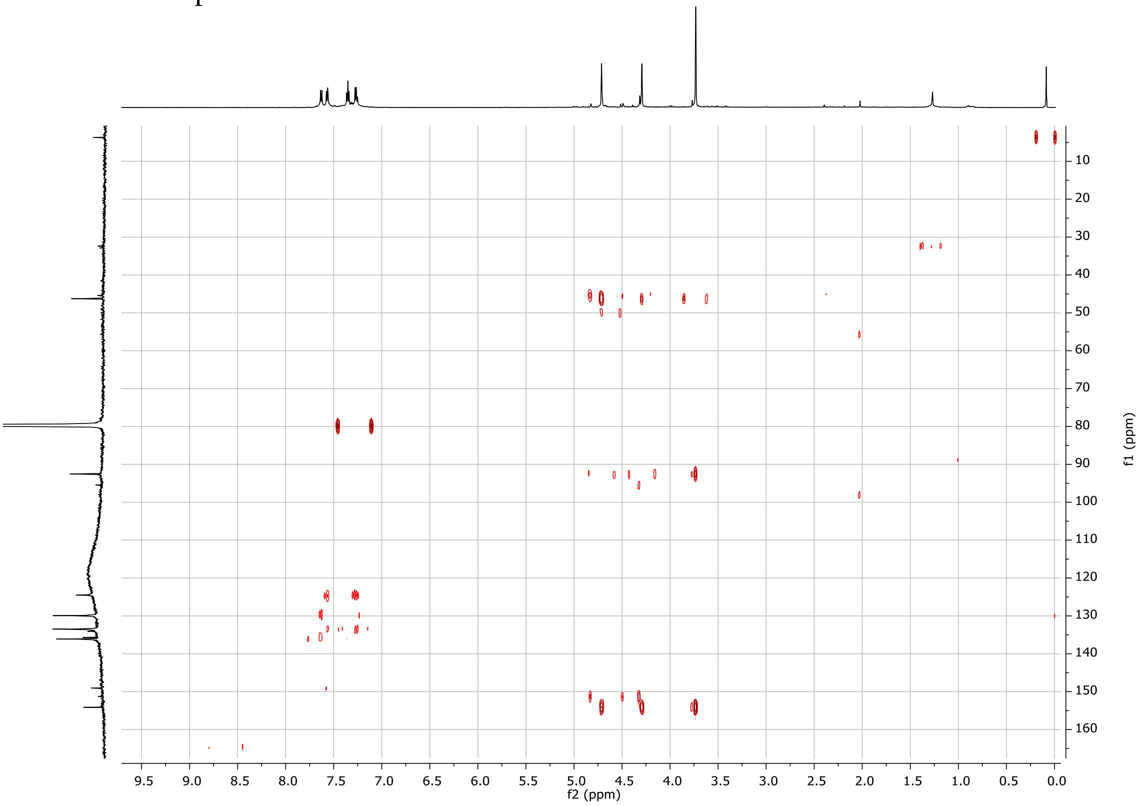

^{1}H NMR of compound **2j** (600 MHz, $CDCl_3$, 25 ºC)

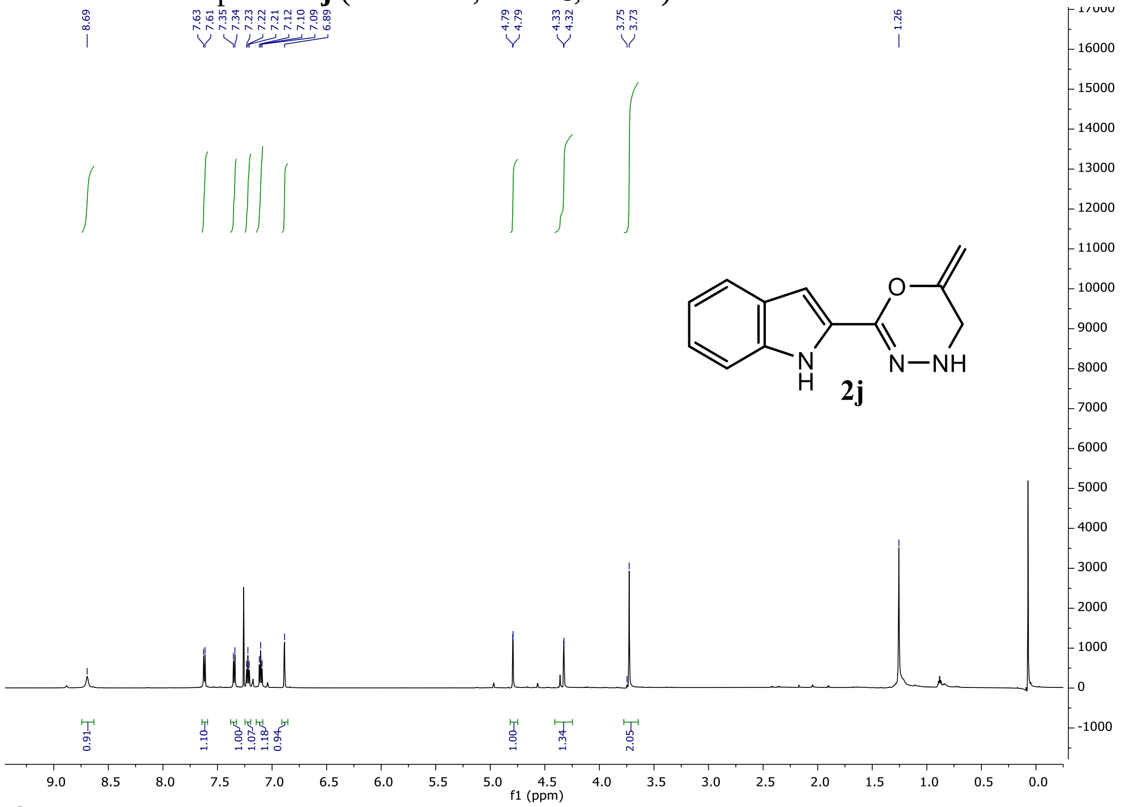

^{13}C NMR of compound **2j** (151 MHz, $CDCl_3$, 25 ºC)

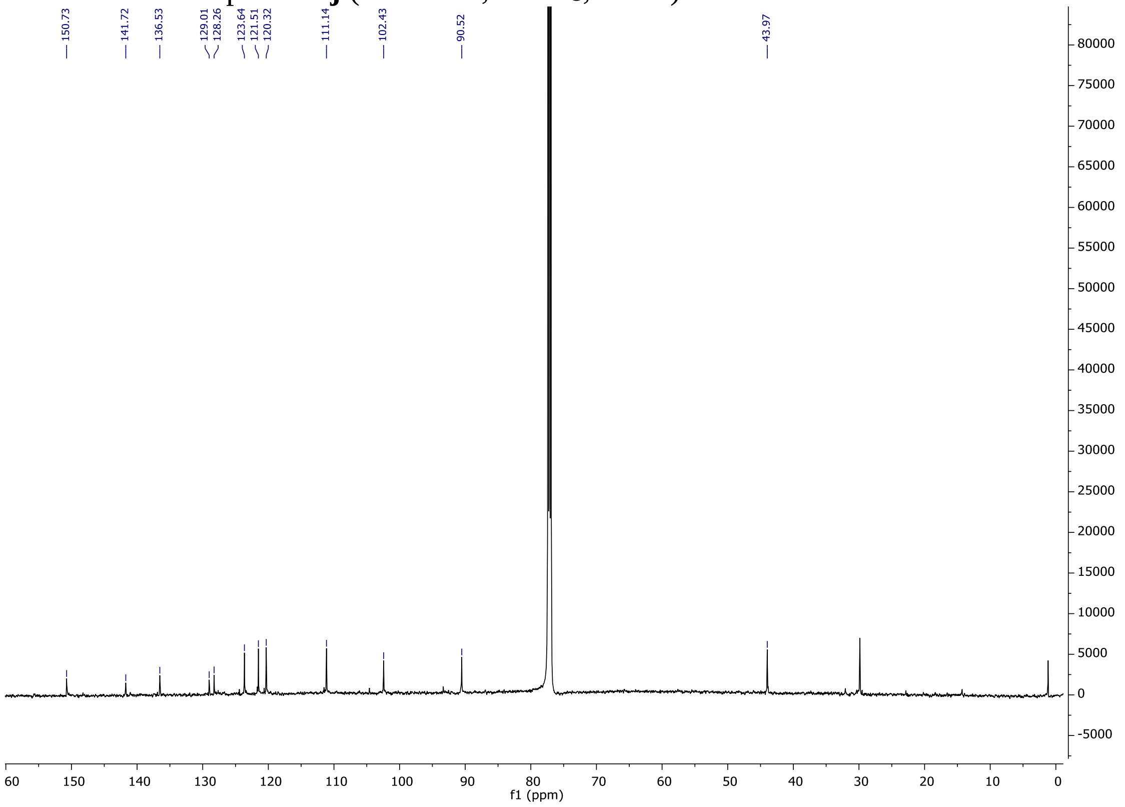

HSQC of compound **2j**

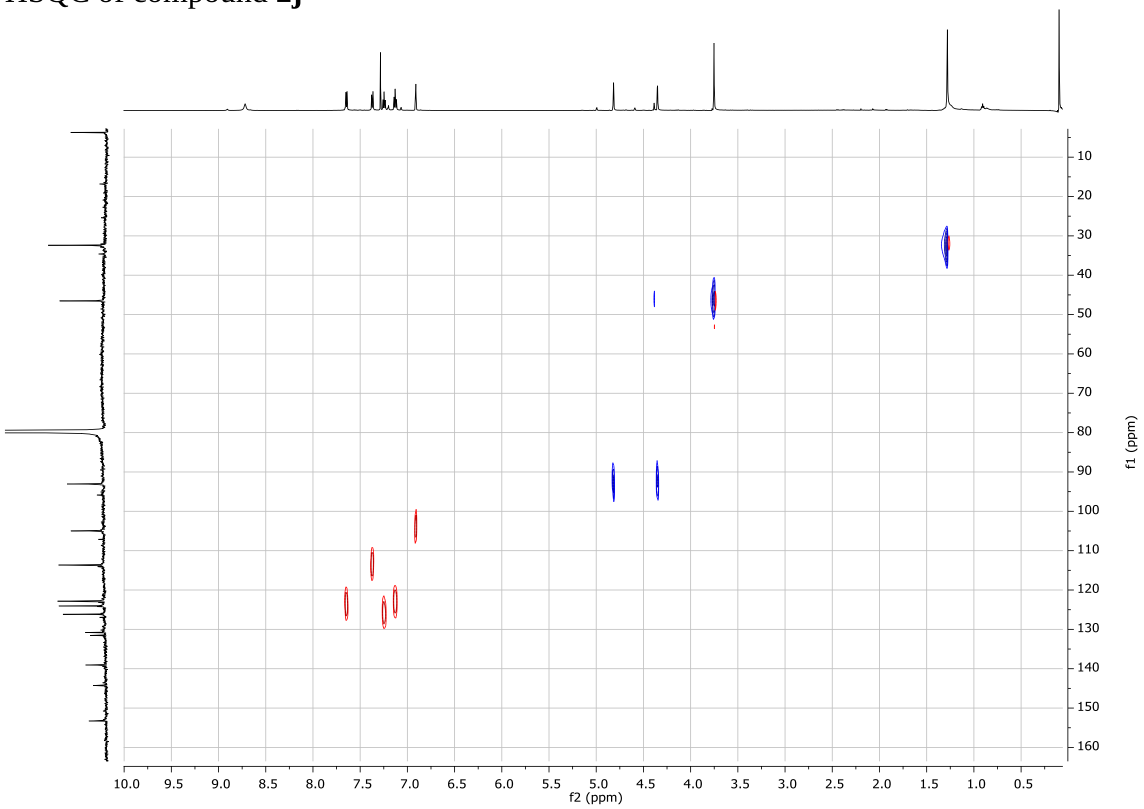

HMBC of compound **2j**

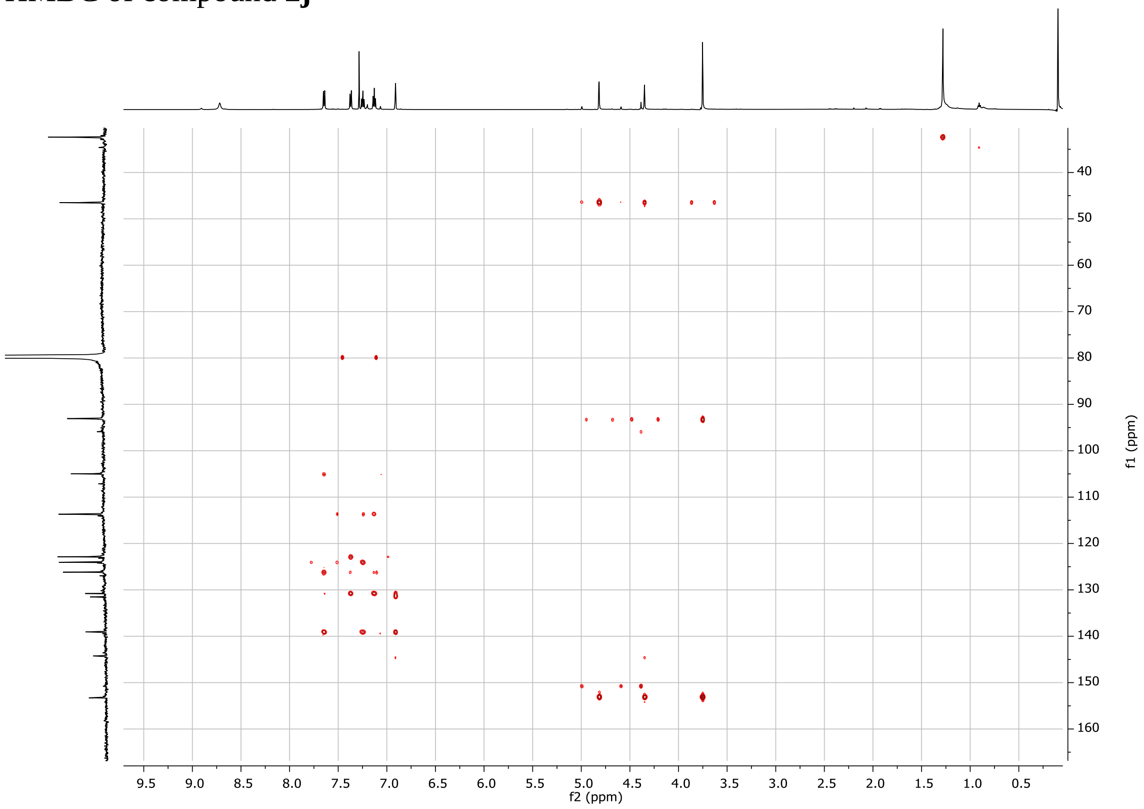

^{1}H NMR of compound **2k** (600 MHz, $CDCl_3$, 25 ºC)

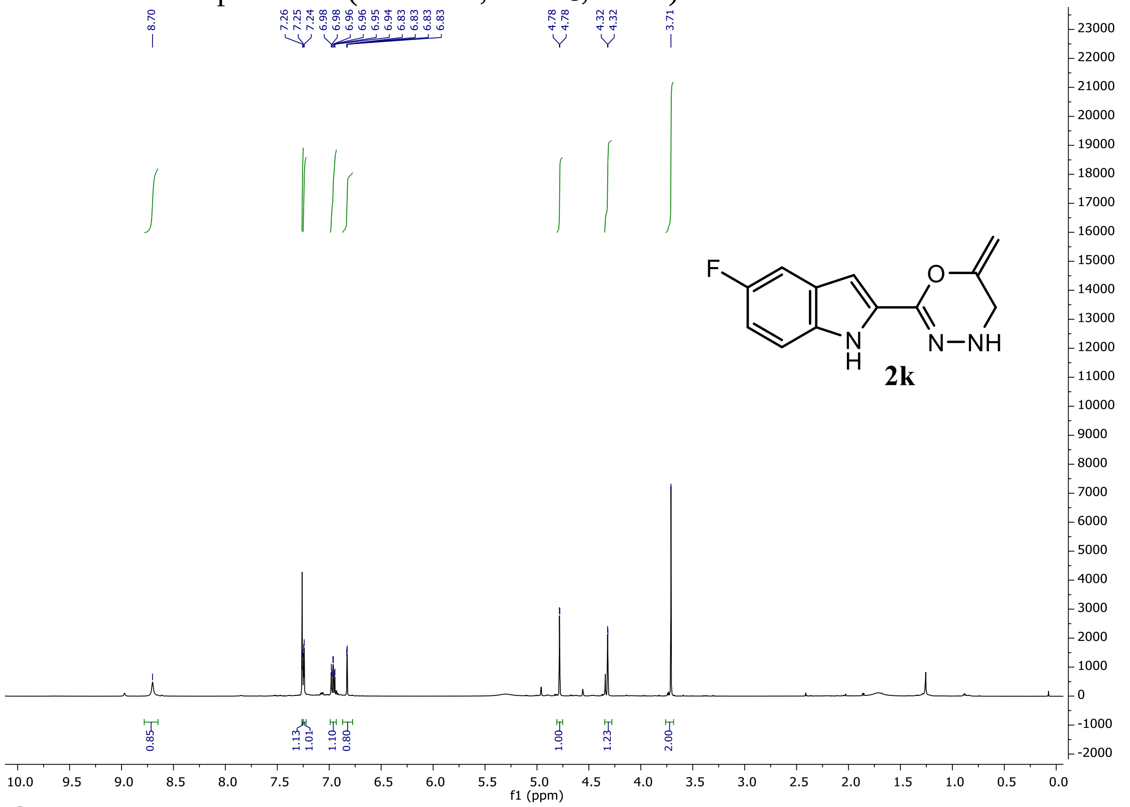

^{13}C NMR of compound **2k** (151 MHz, $CDCl_3$, 25 ºC)

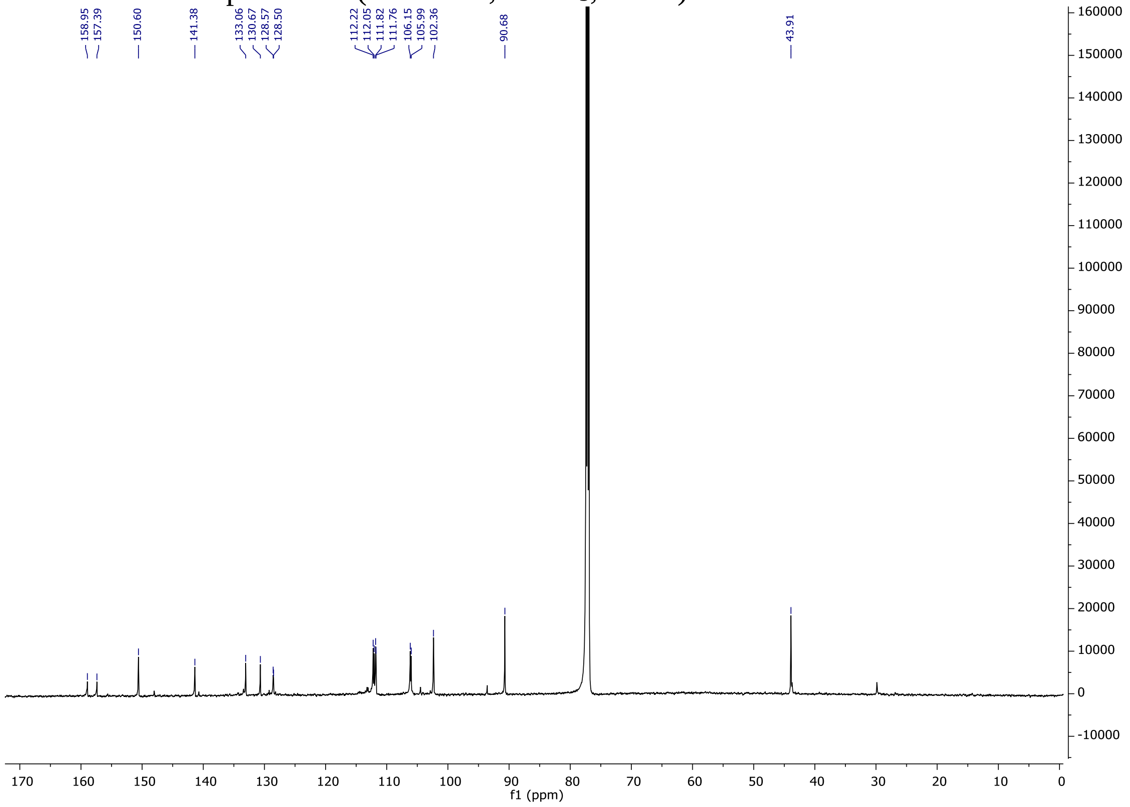

HSQC of compound **2k**

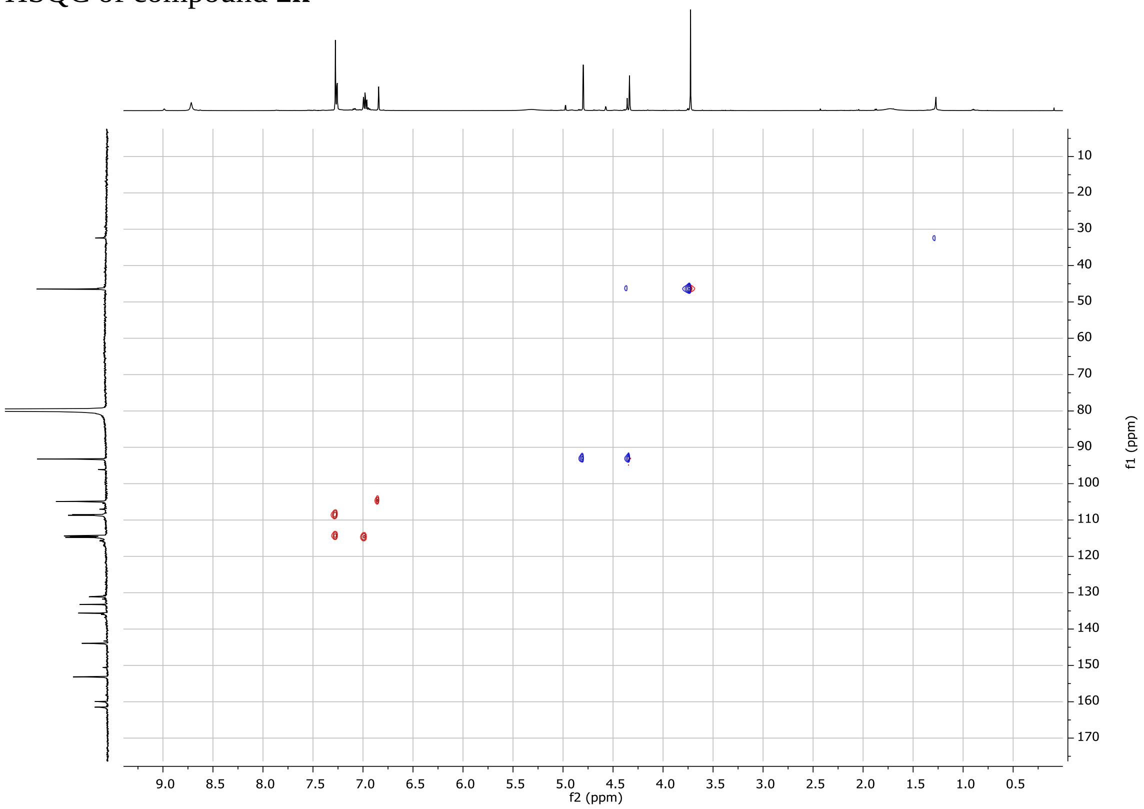

HMBC of compound **2k**

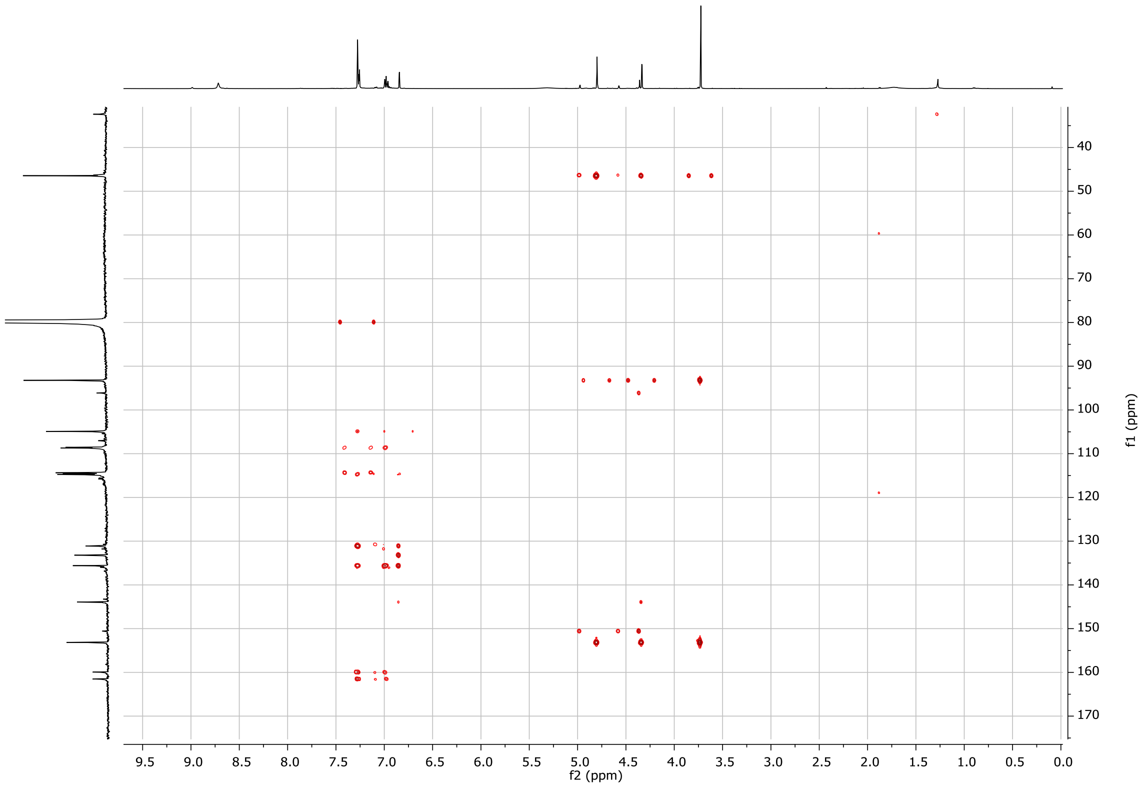

^{1}H NMR of compound **2l** (600 MHz, $CDCl_3$, 25 ºC)

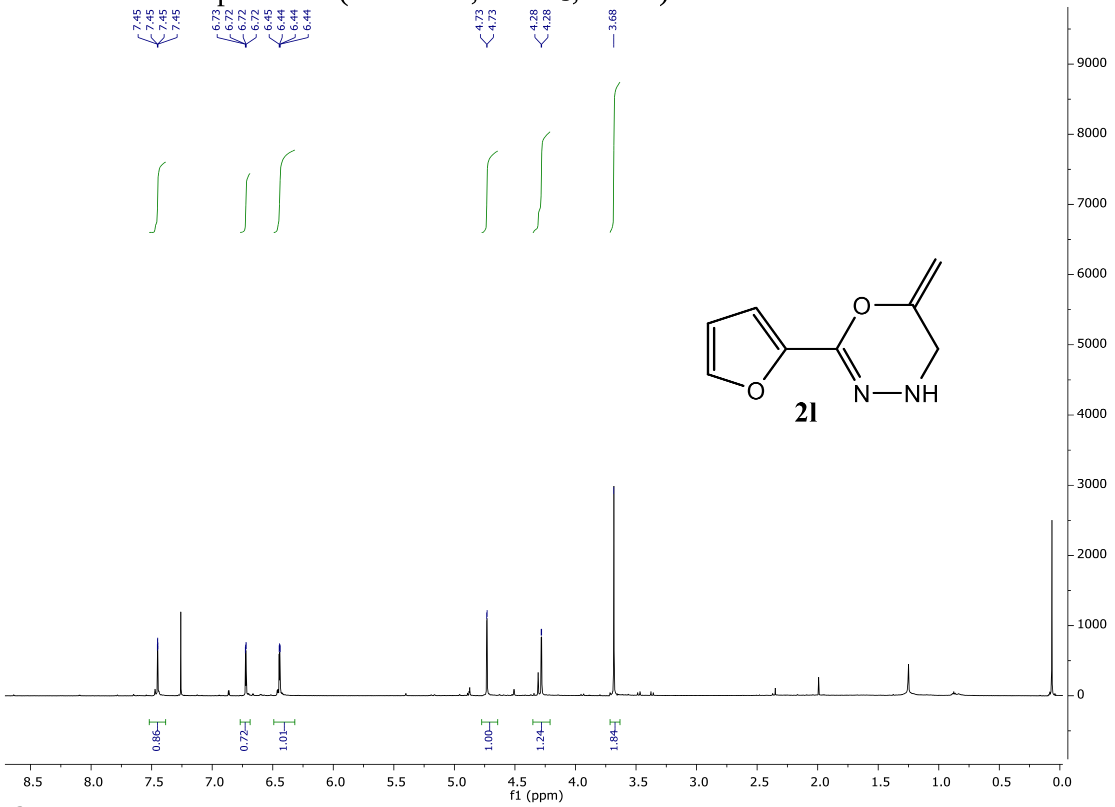

^{13}C NMR of compound **2l** (151 MHz, $CDCl_3$, 25 ºC)

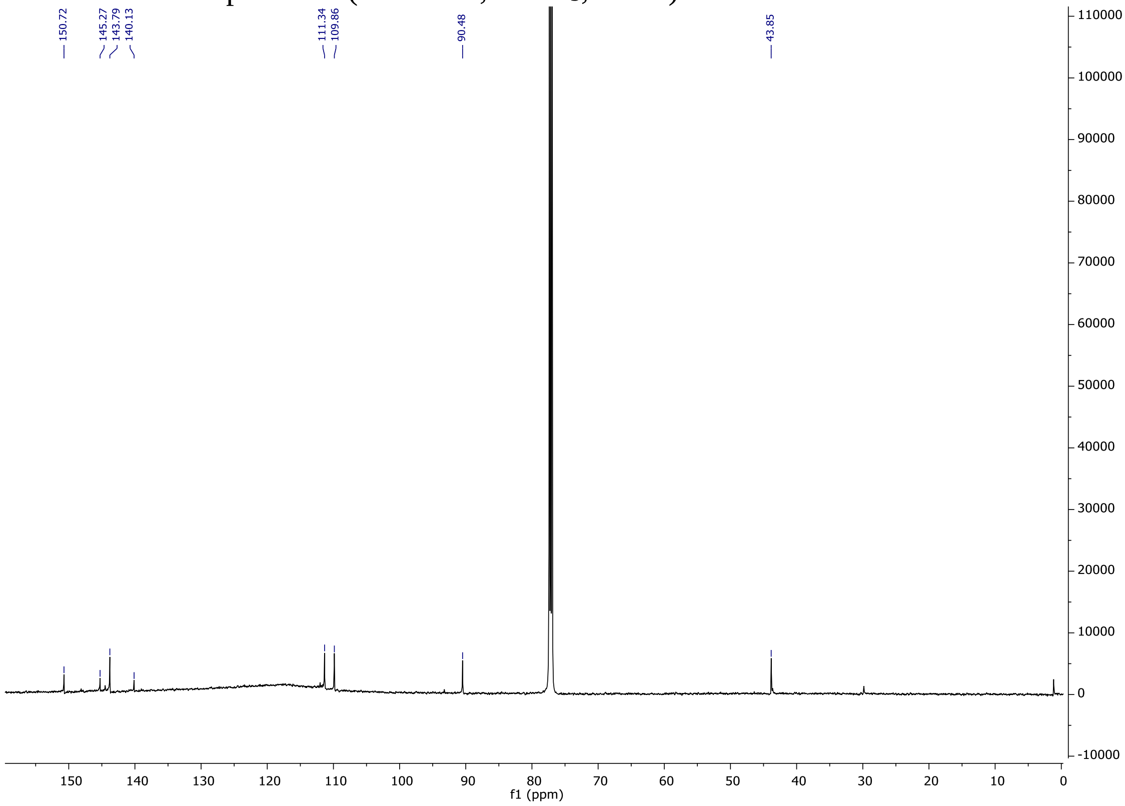

HSQC of compound **2l**

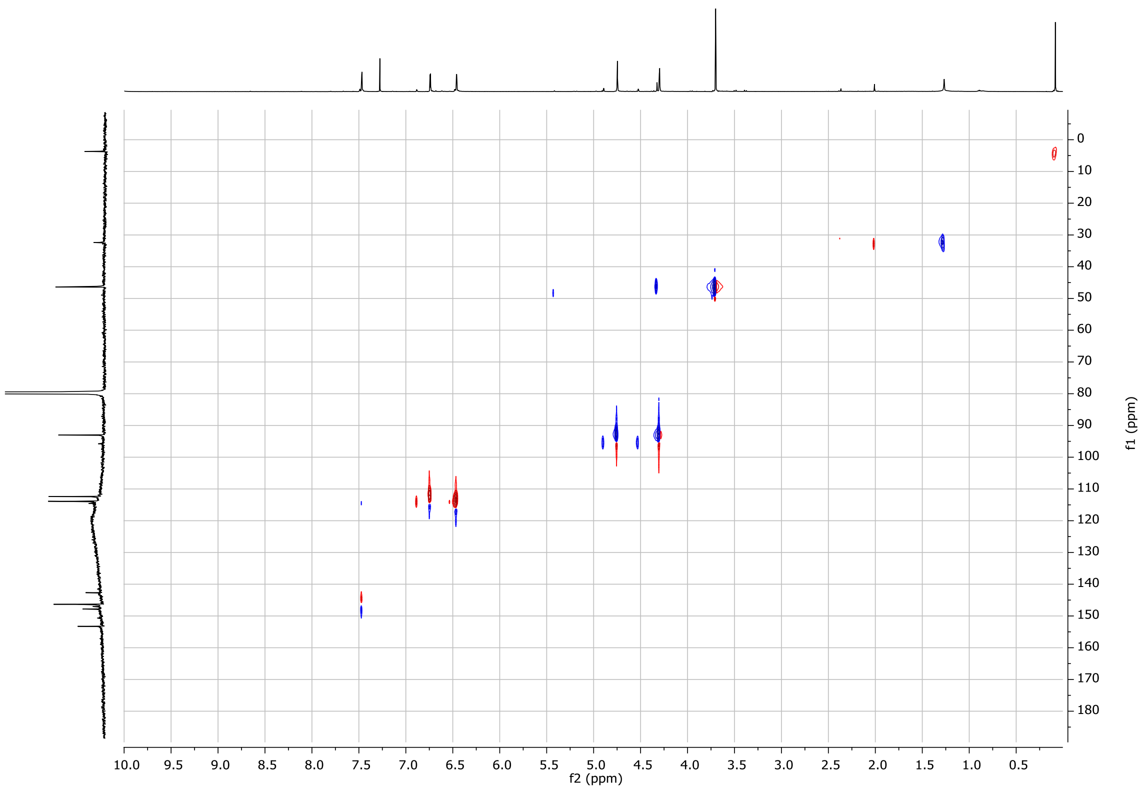

HMBC of compound **2l**

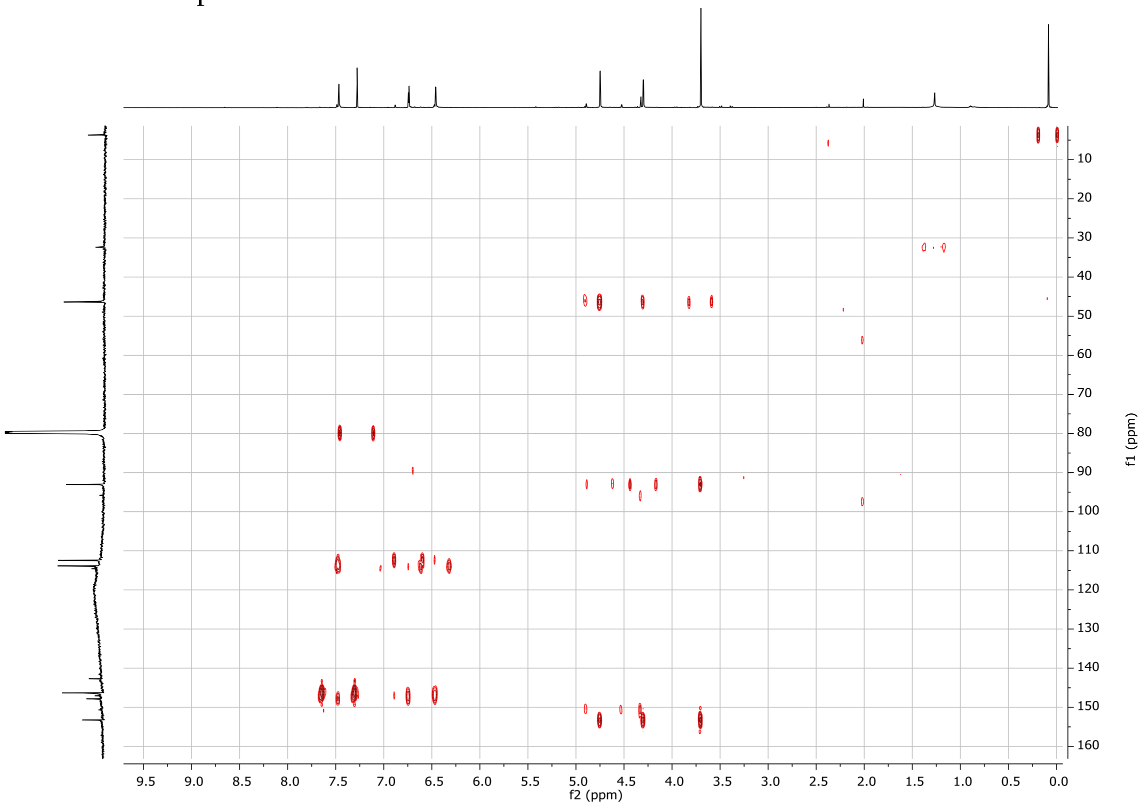

^{1}H NMR of compound **2m** (600 MHz, $CDCl_3$, 25 ºC)

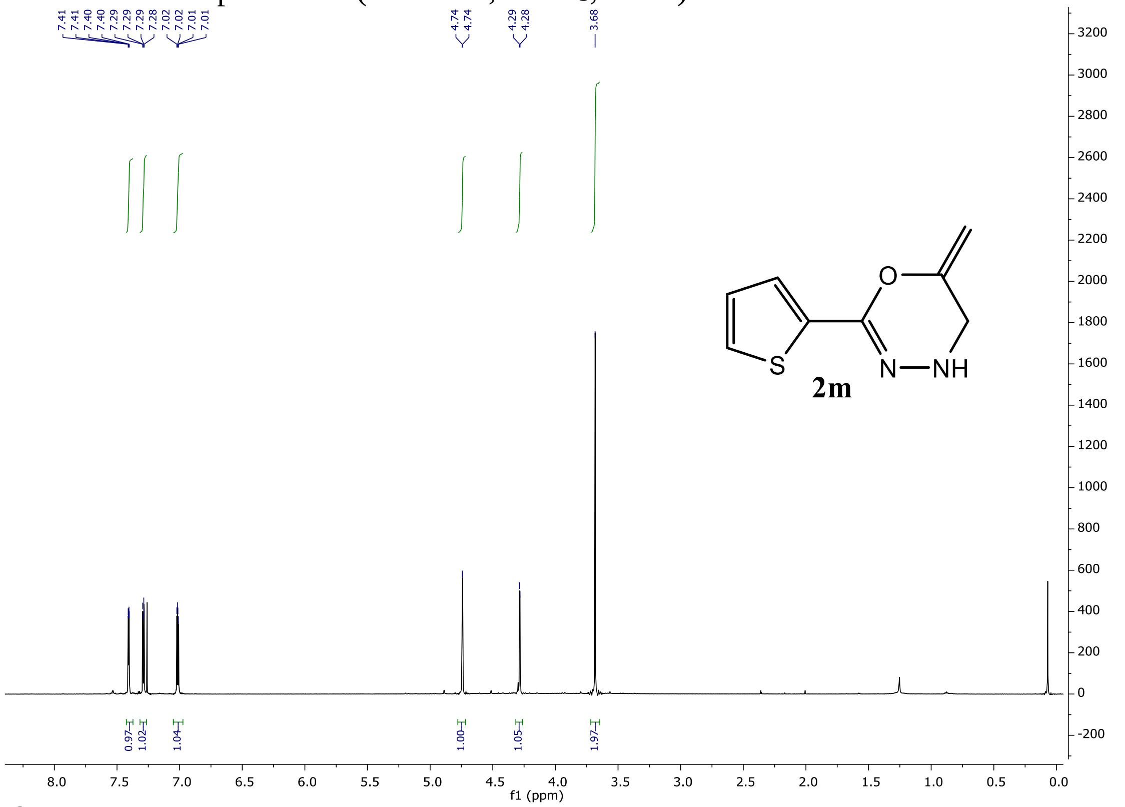

^{13}C NMR of compound **2m** (151 MHz, $CDCl_3$, 25 ºC)

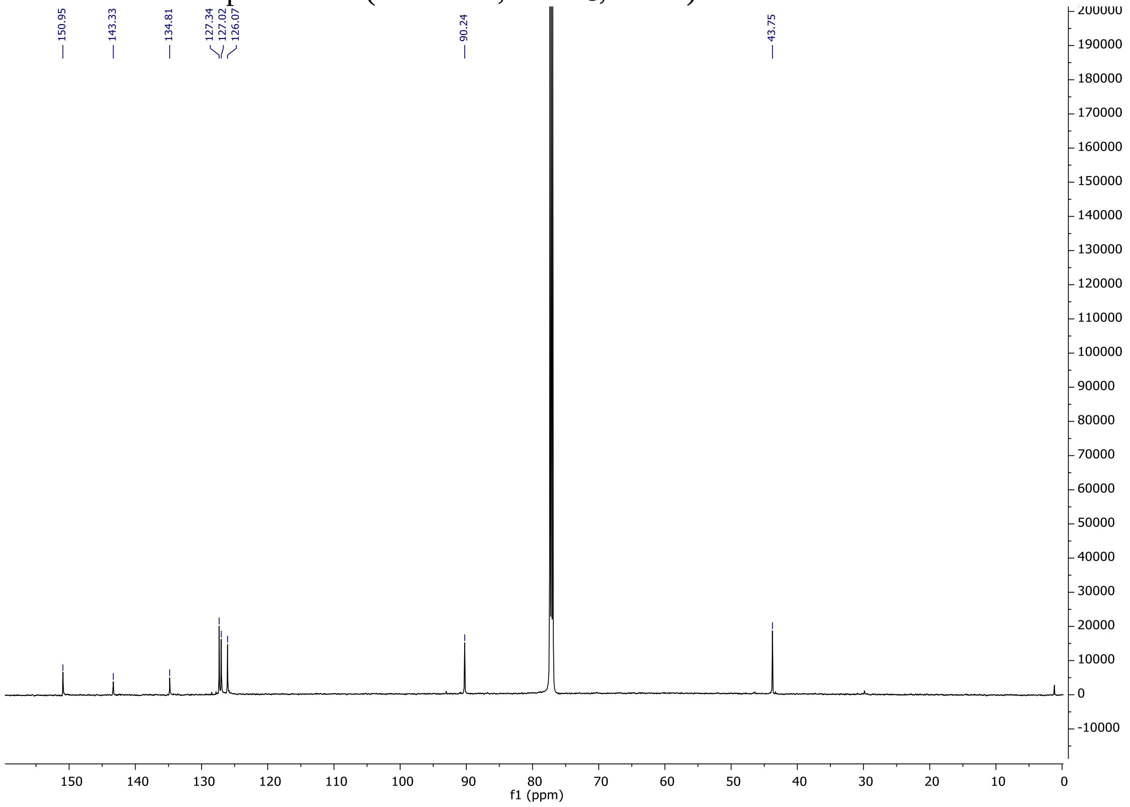

HSQC of compound **2m**

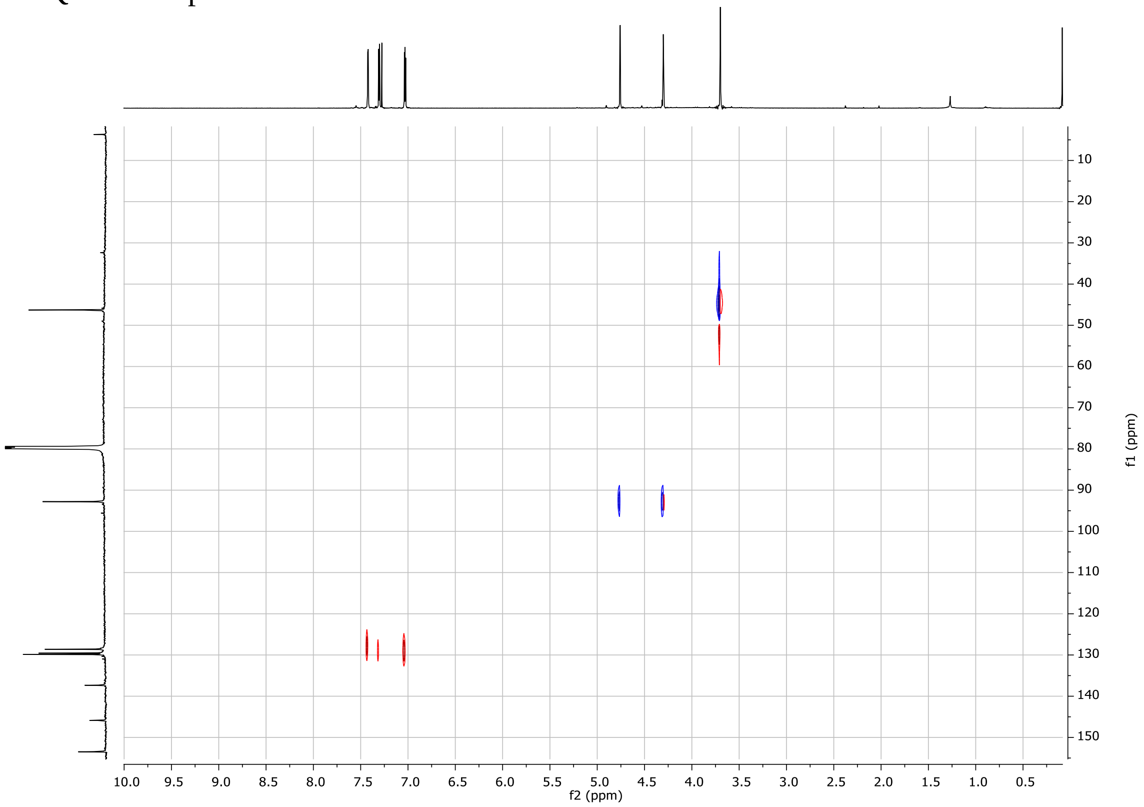

HMBC of compound **2m**

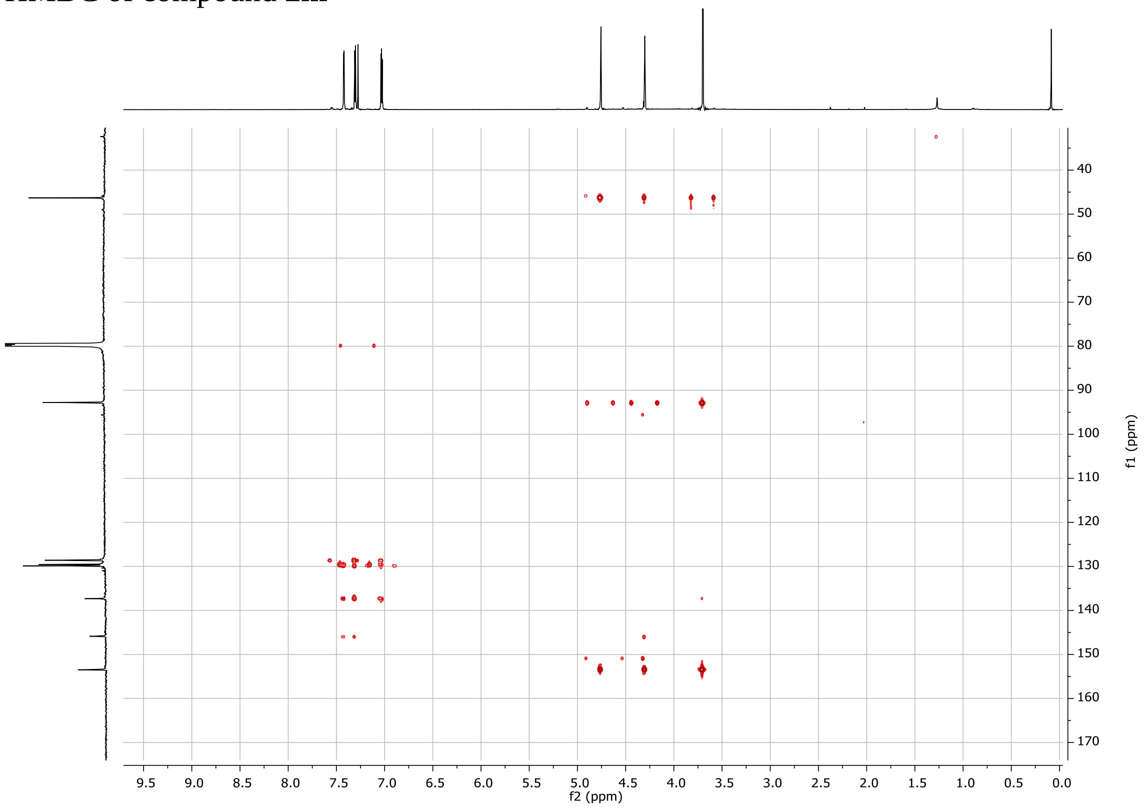

^{1}H NMR of compound **2n** (700 MHz, $CDCl_3$, 25 ºC)

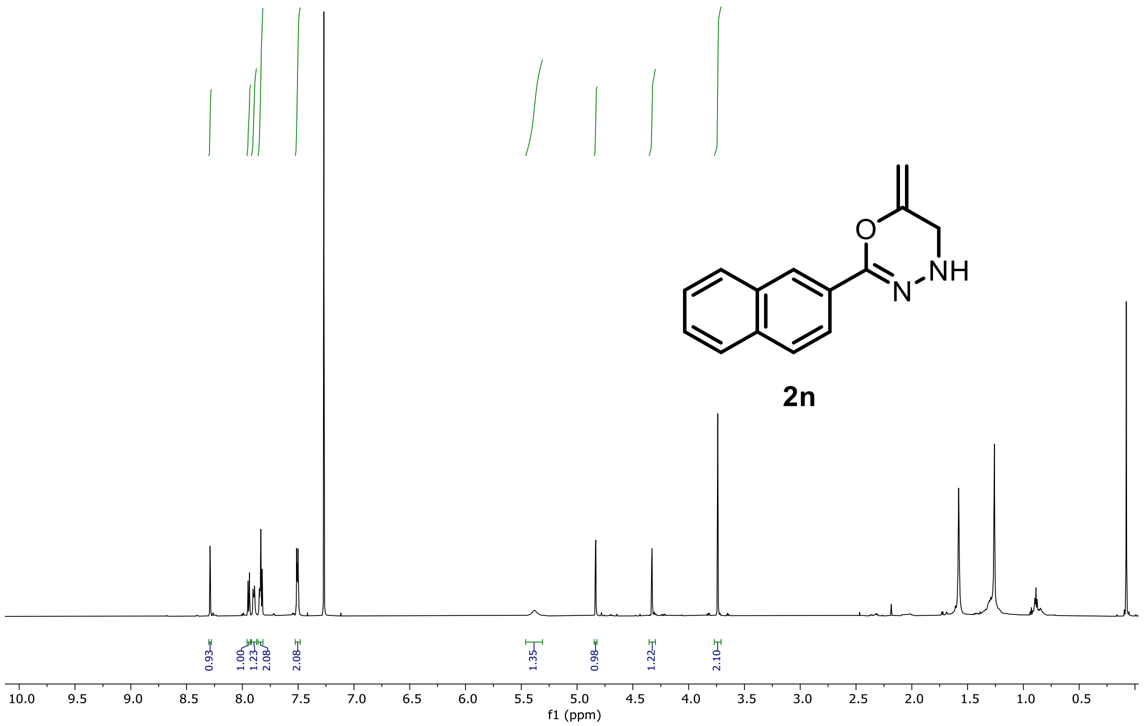

^{13}C NMR of compound **2n** (175 MHz, $CDCl_3$, 25 ºC)

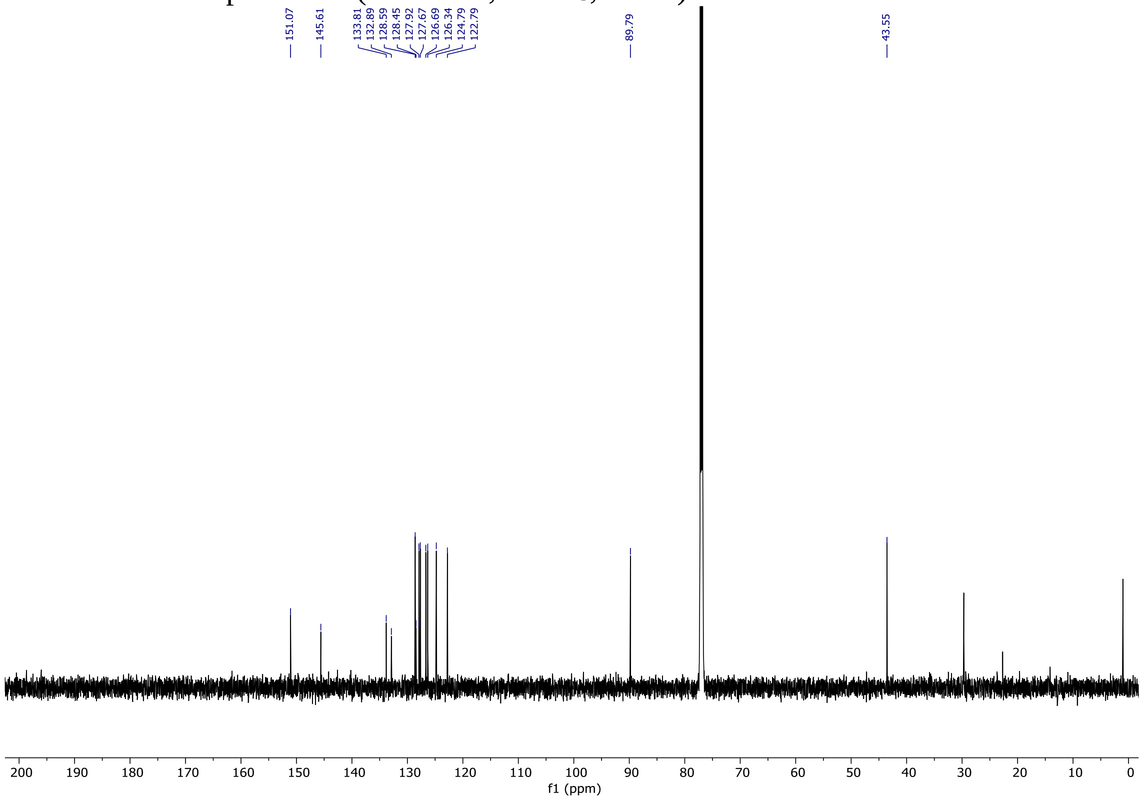

^{1}H NMR of compound **2n-N** (600 MHz, $CDCl_3$, 25 ºC)

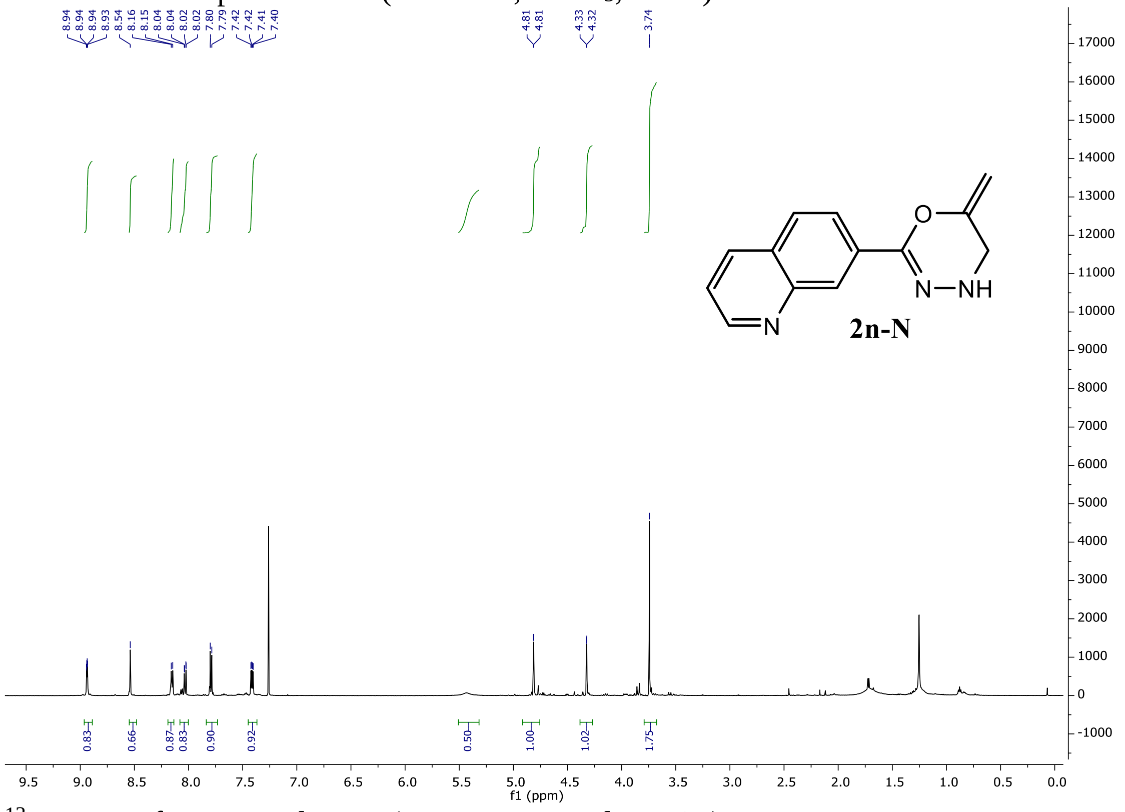

^{13}C NMR of compound **2n-N** (151 MHz, $CDCl_3$, 25 ºC)

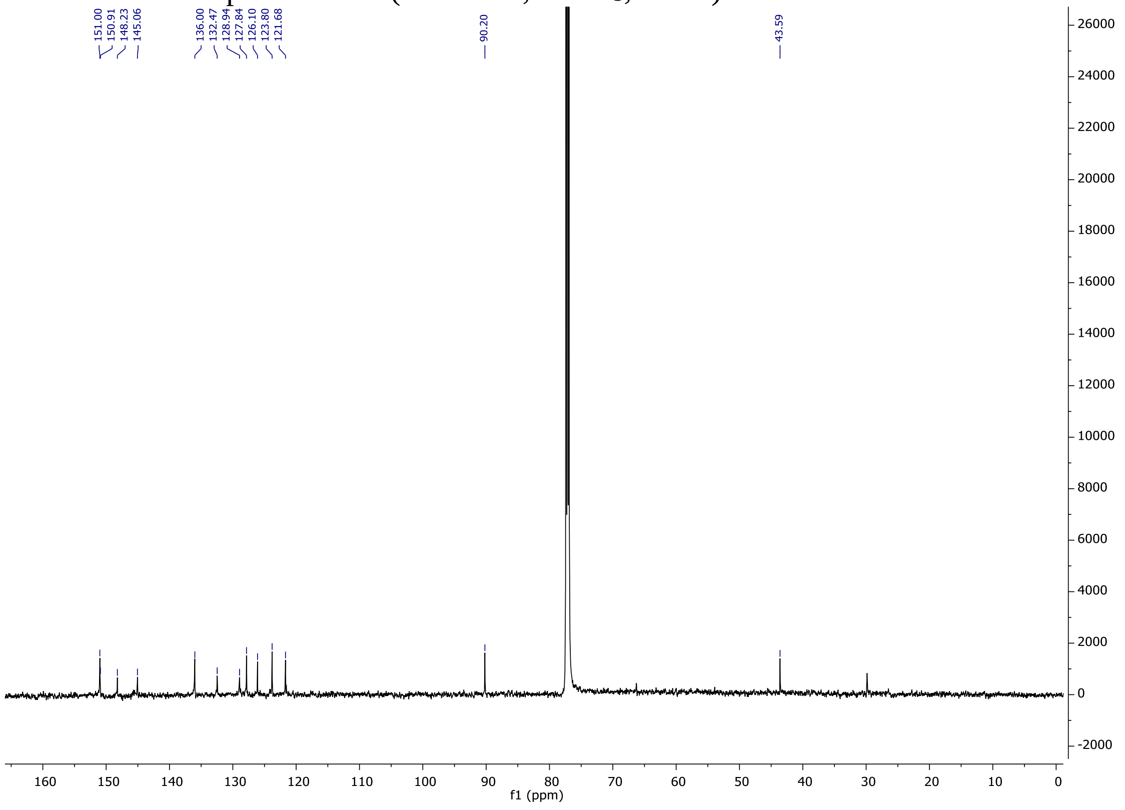

HSQC of compound **2n-N**

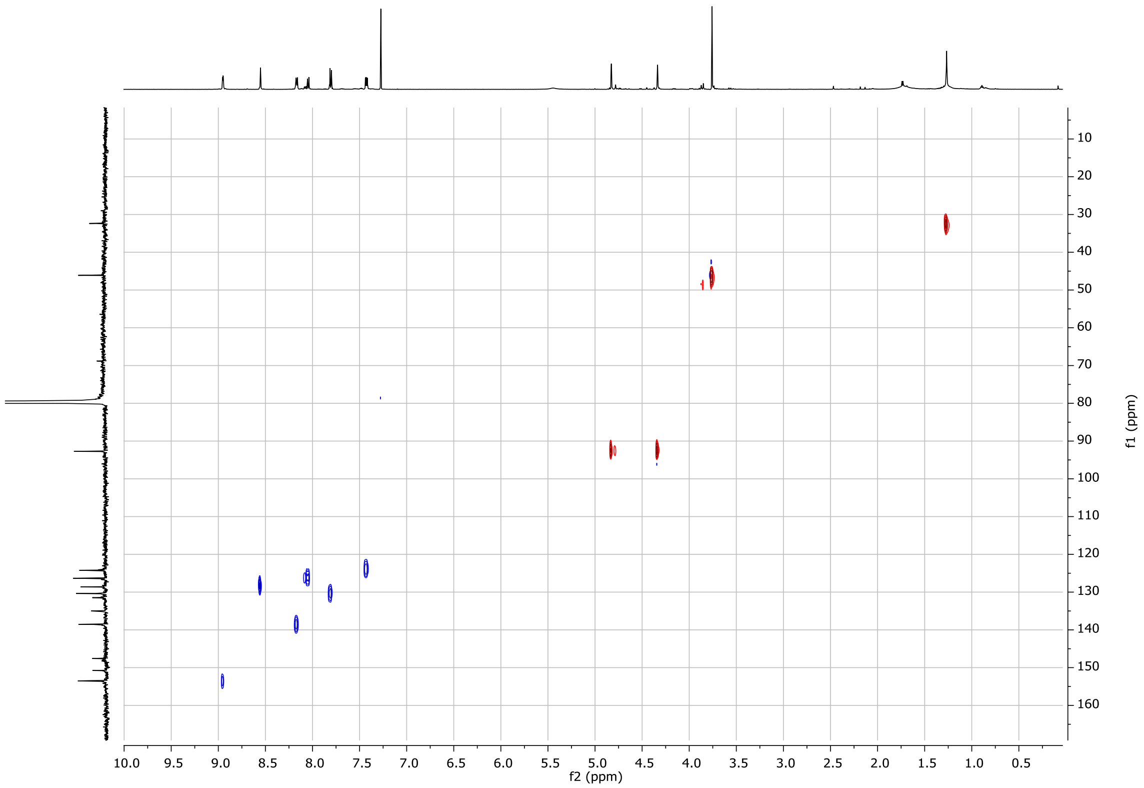

HMBC of compound **2n-N**

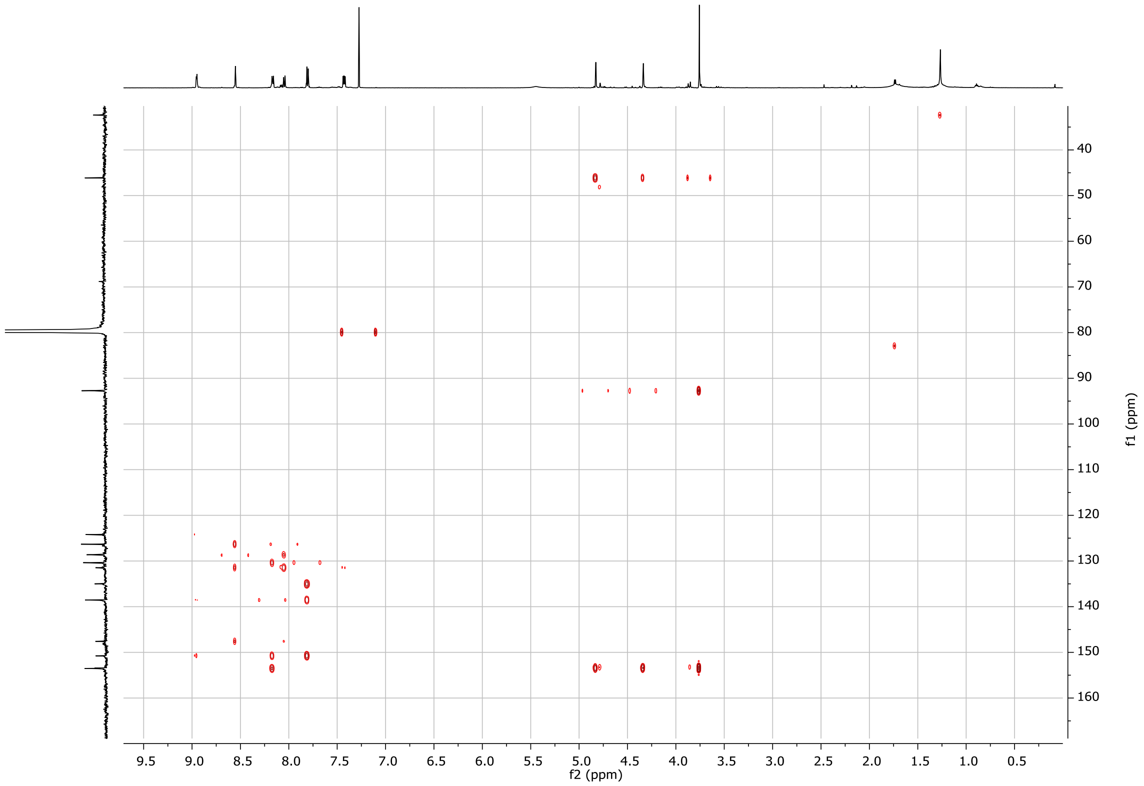

^{1}H NMR of compound **3a** (300 MHz, $CDCl_3$, 25 ºC)

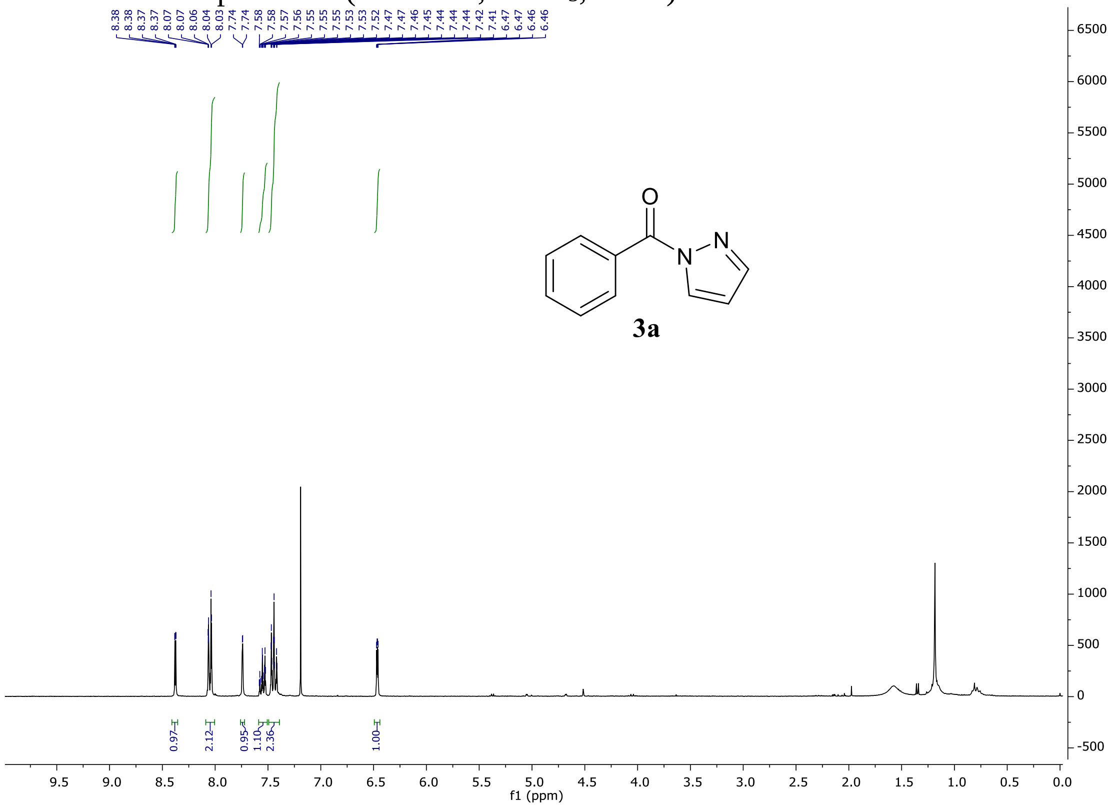

^{13}C NMR of compound **3a** (75 MHz, $CDCl_3$, 25 ºC)

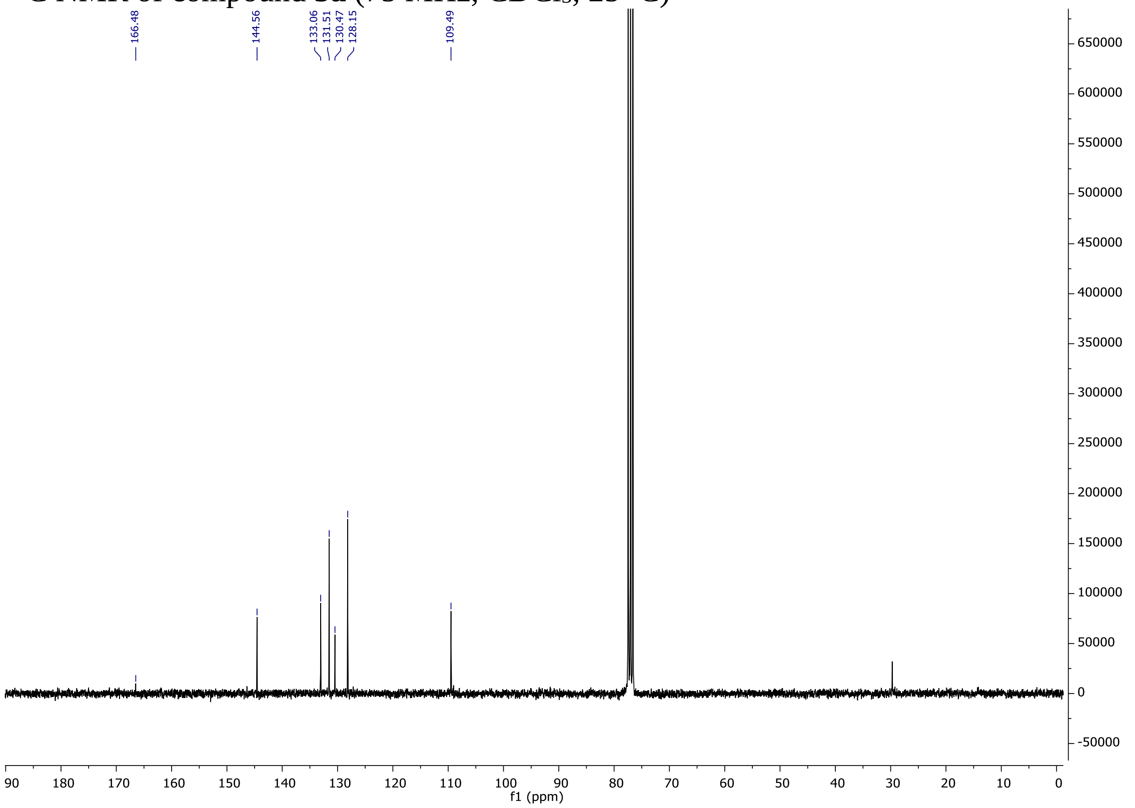

DEPT of compound **3a** (75 MHz, $CDCl_3$, 25 ºC)

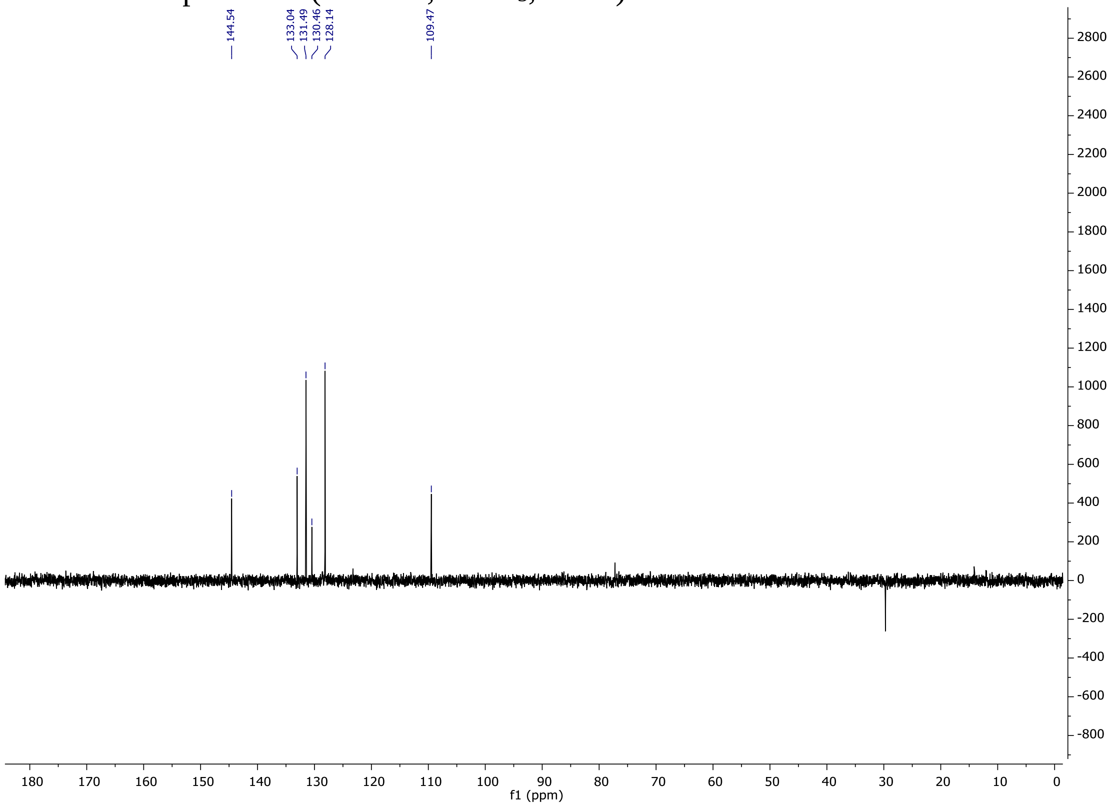

^{1}H NMR of compound **3b** (600 MHz, $CDCl_3$, 25 ºC)

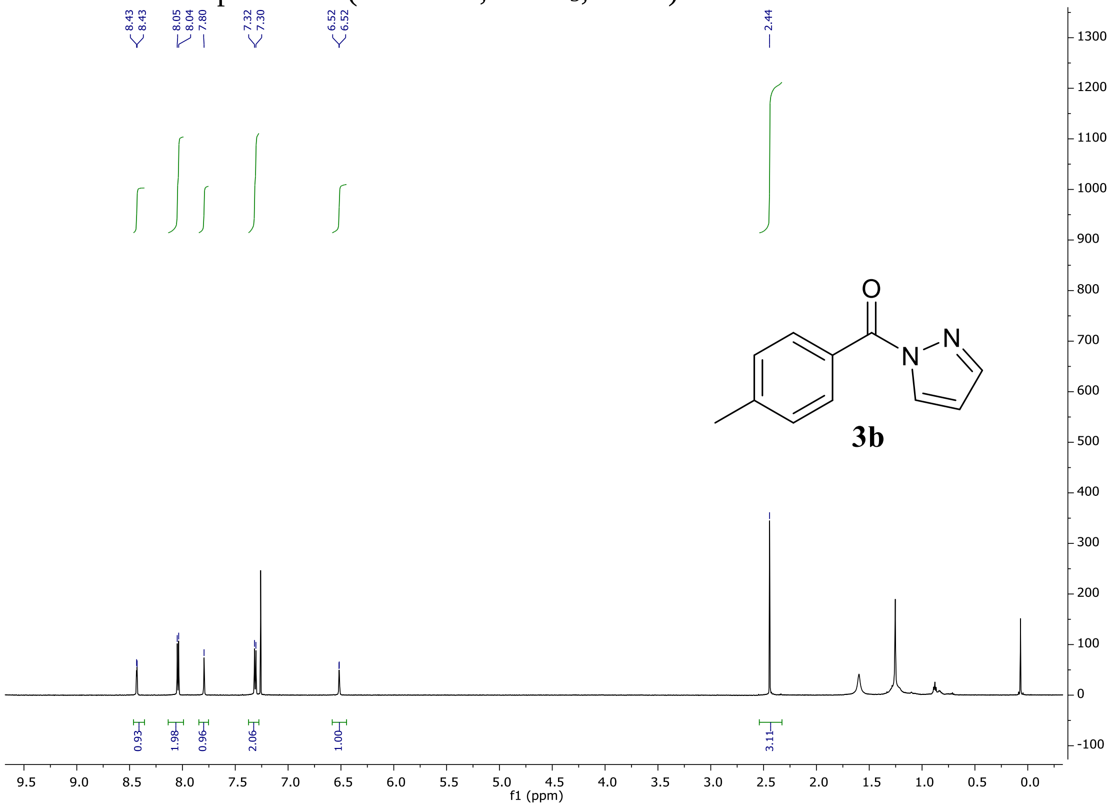

^{13}C NMR of compound **3b** (151 MHz, $CDCl_3$, 25 ºC)

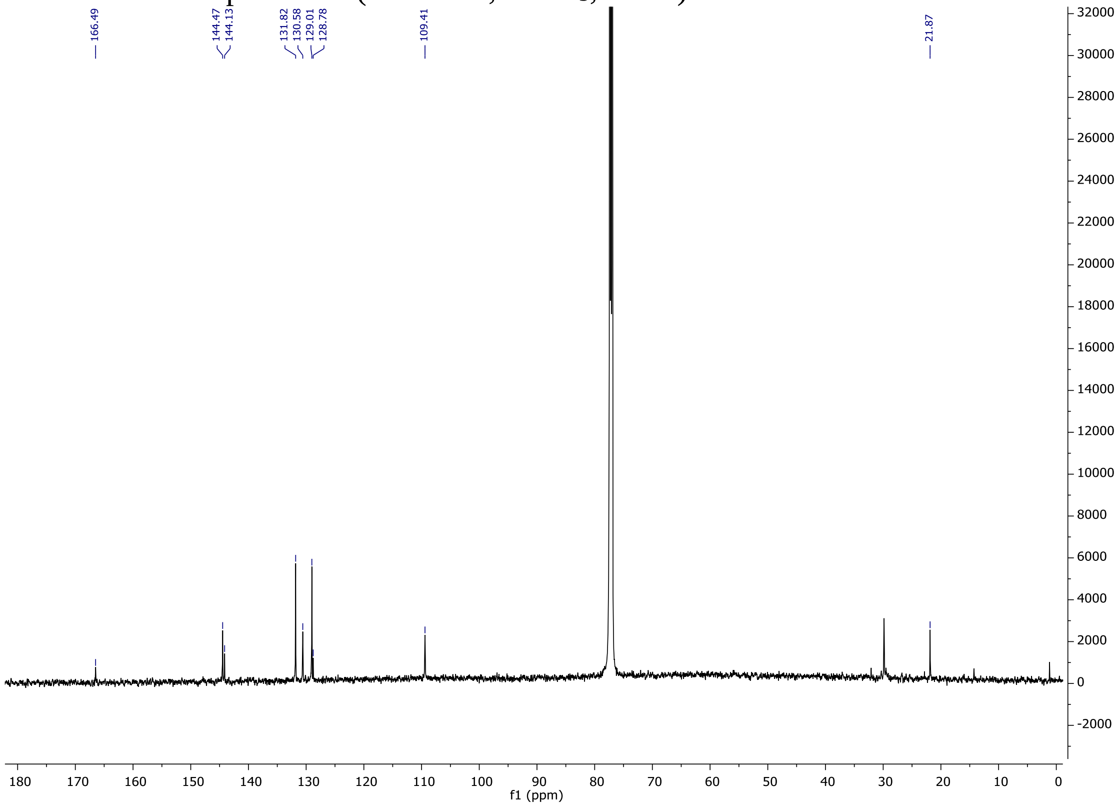

HSQC of compound **3b**

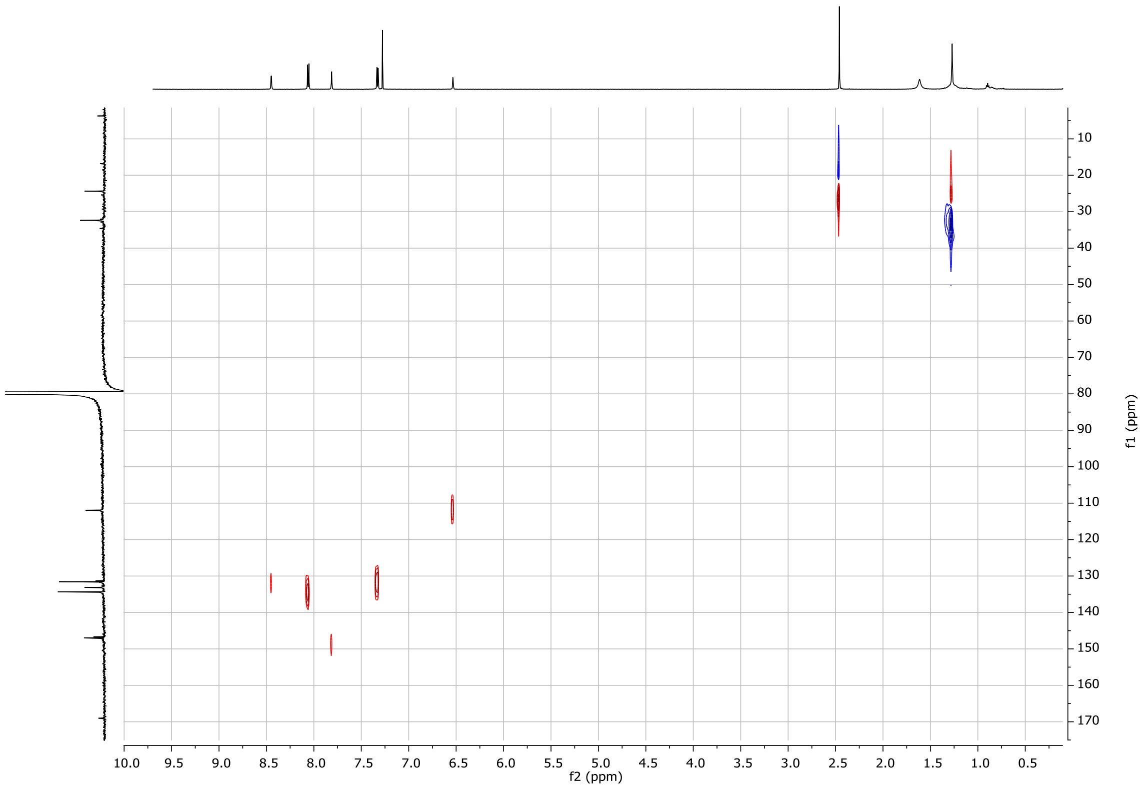

HMBC of compound **3b**

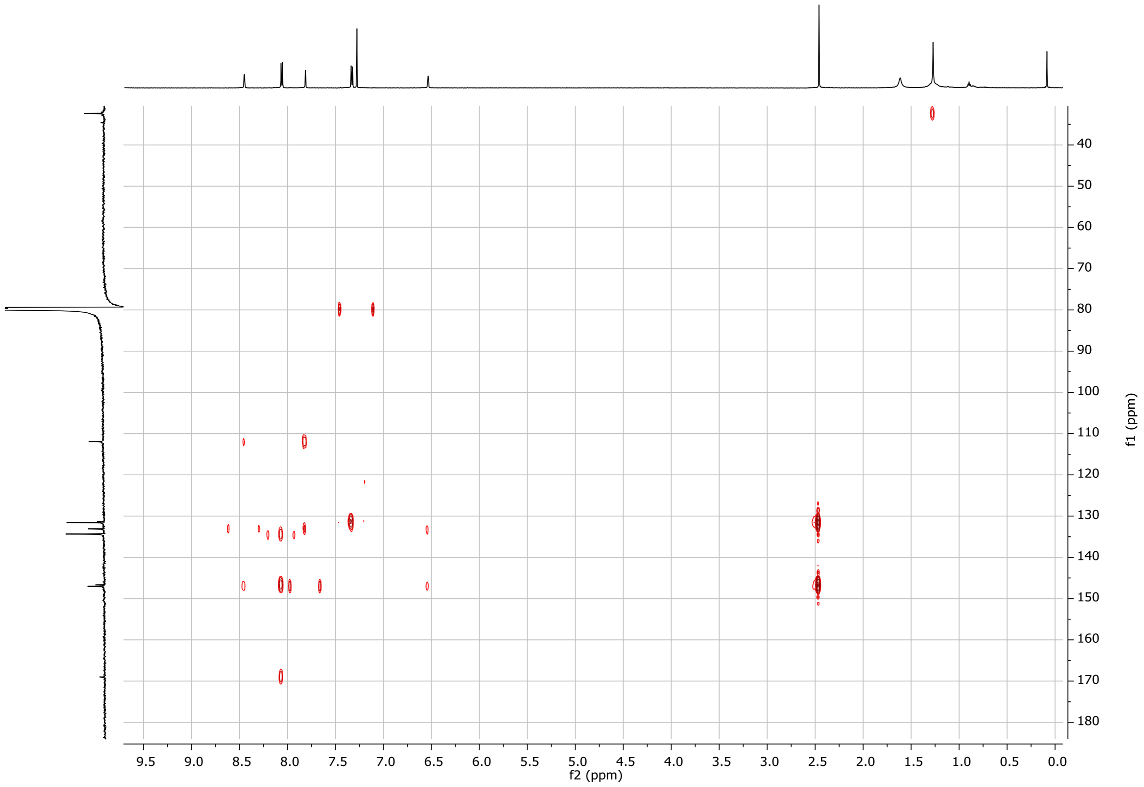

^{1}H NMR of compound **3c** (600 MHz, Acetone d6, 25 ºC)

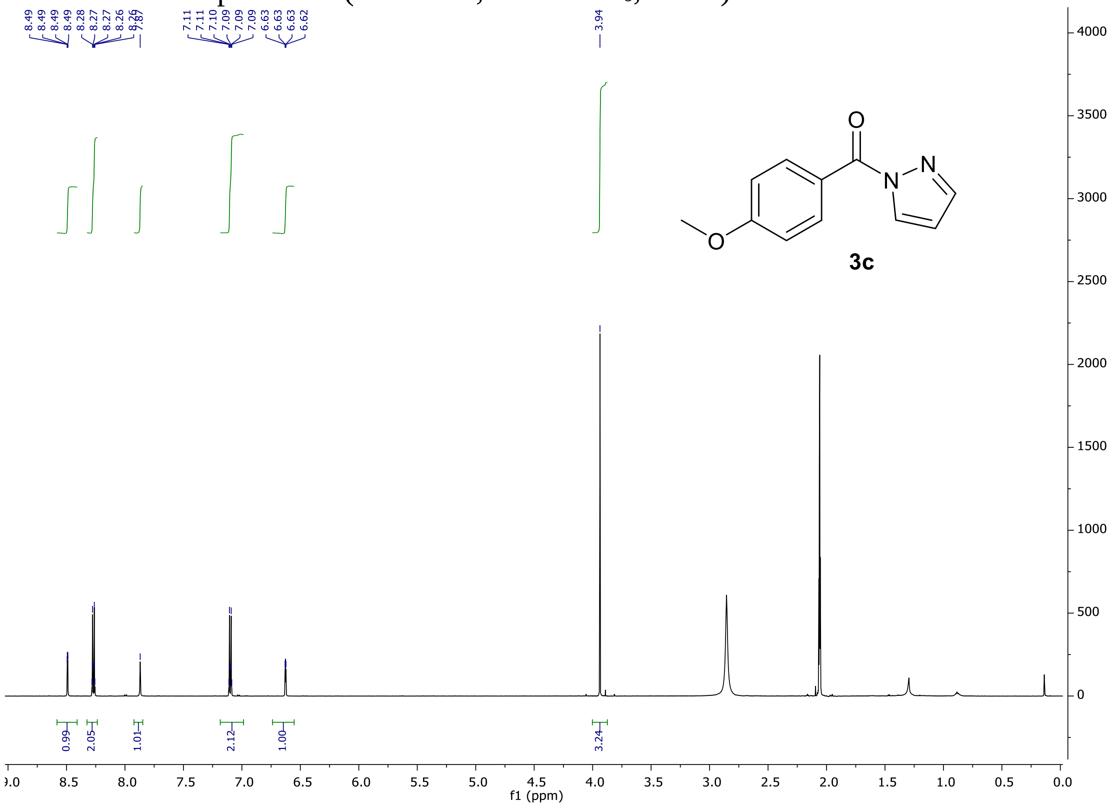

^{13}C NMR of compound **3c** (151 MHz, Acetone d6, 25 ºC)

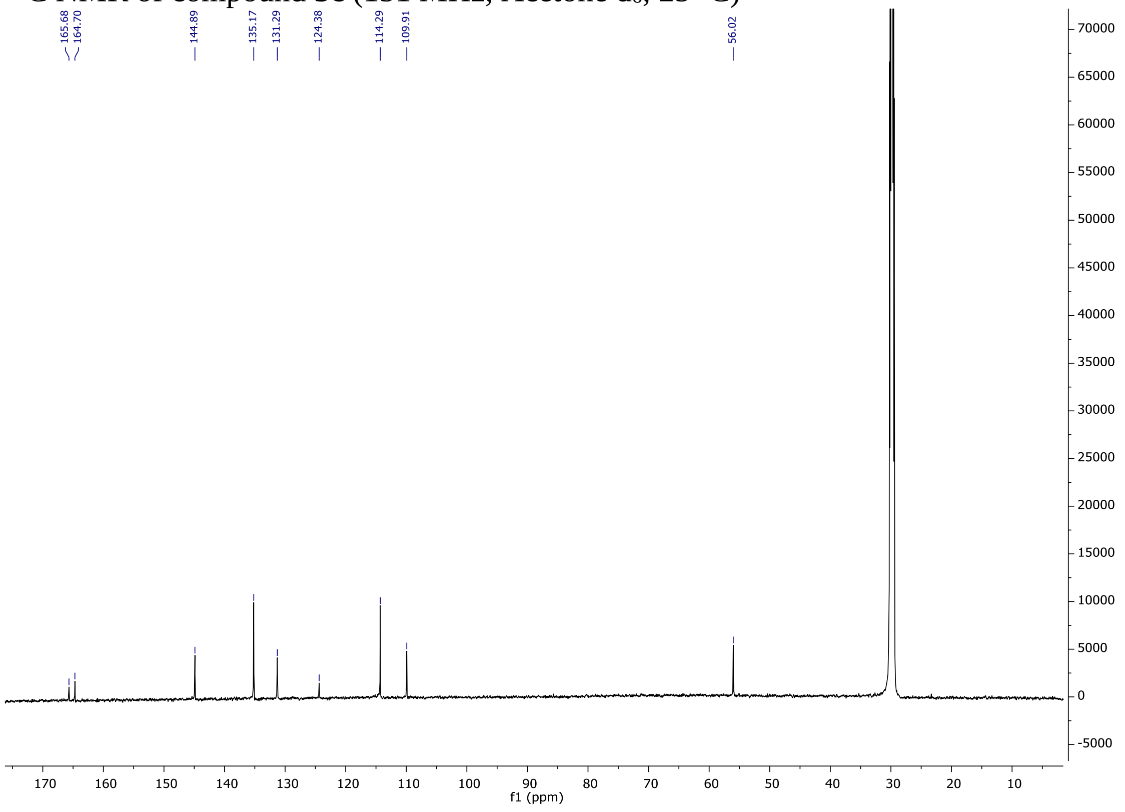

HSQC of compound **3c**

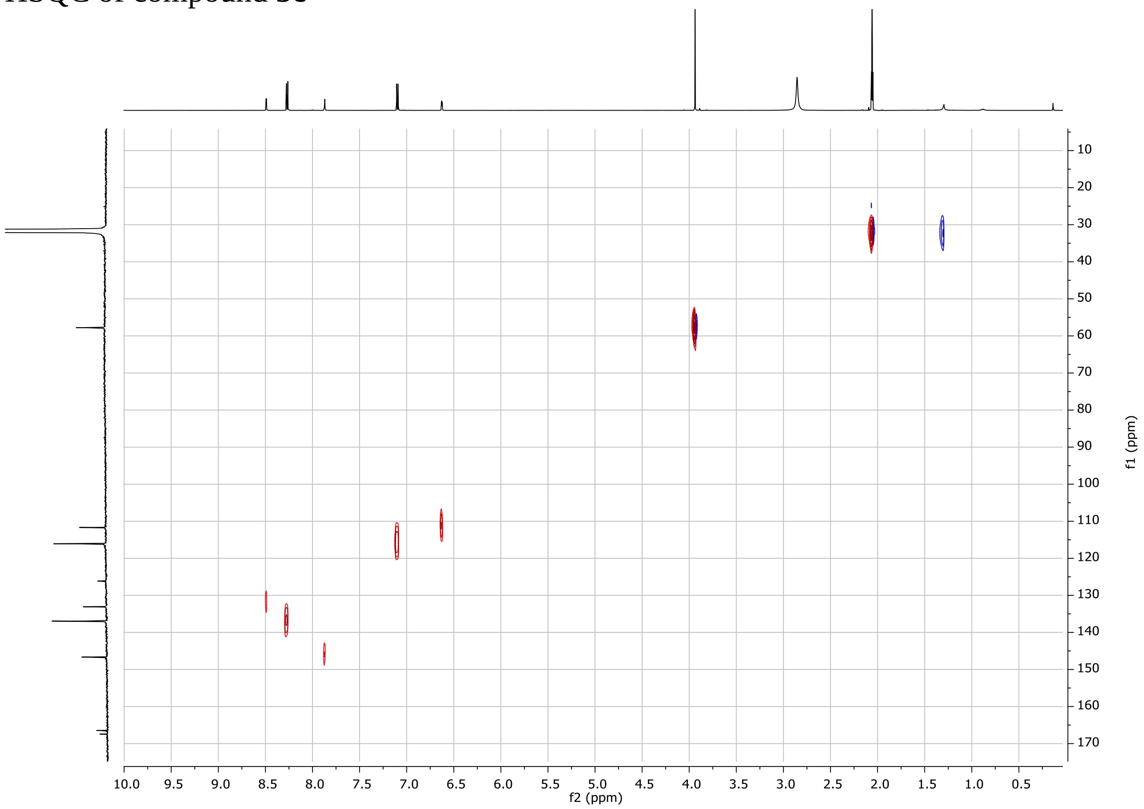

HMBC of compound **3c**

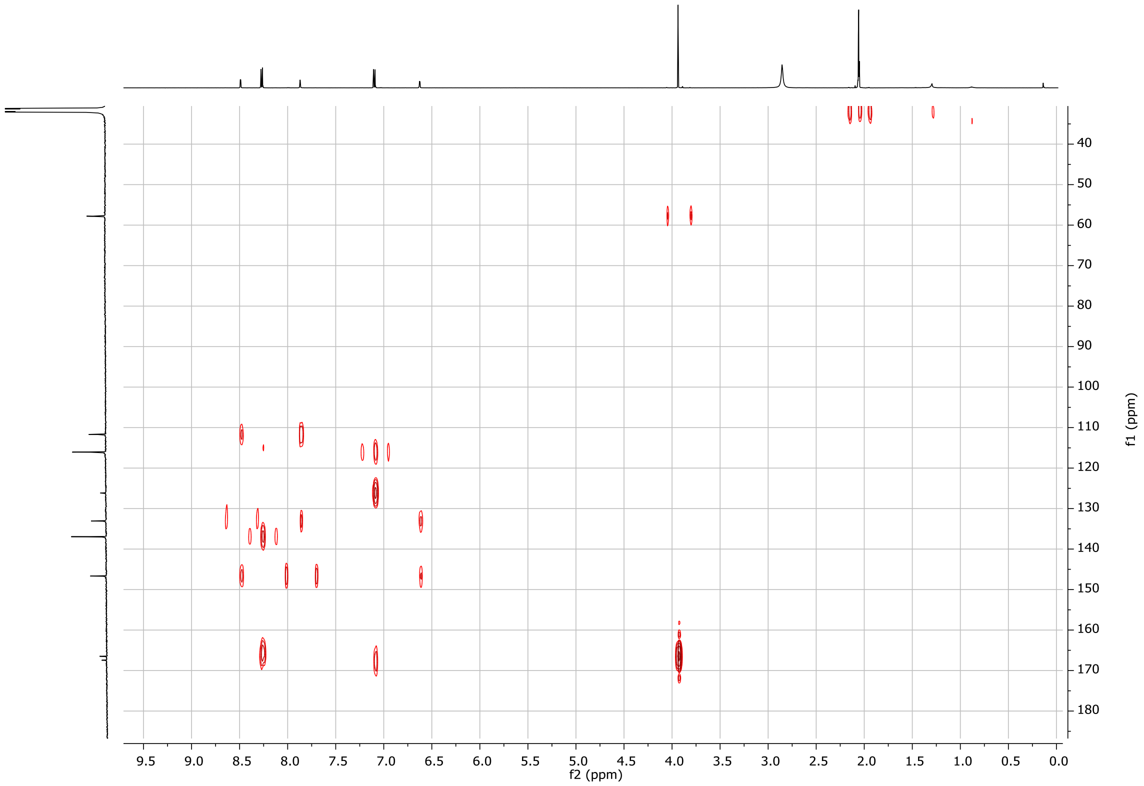

^{1}H NMR of compound **3-N** (300 MHz, $CDCl_3$, 25 ºC)

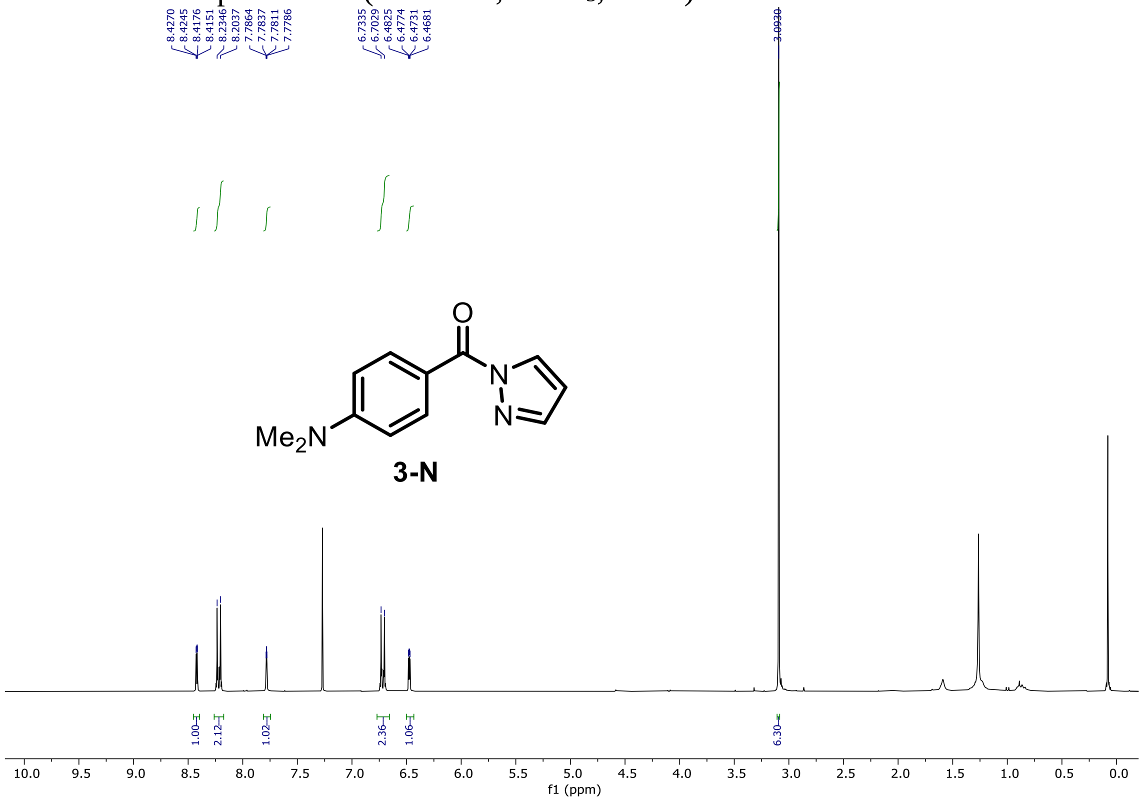

^{13}C NMR of compound **3-N** (75 MHz, $CDCl_3$, 25 ºC)

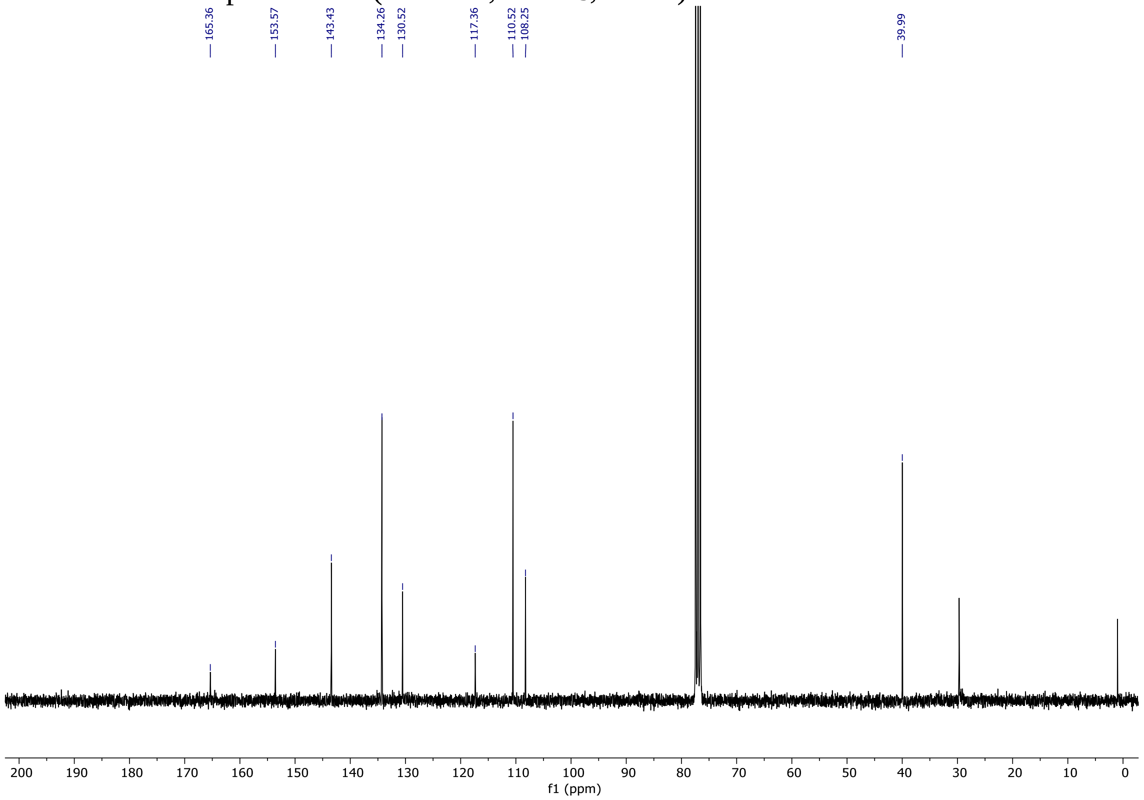

^{1}H NMR of compound **3d** (600 MHz, Acetone d6, 25 ºC)

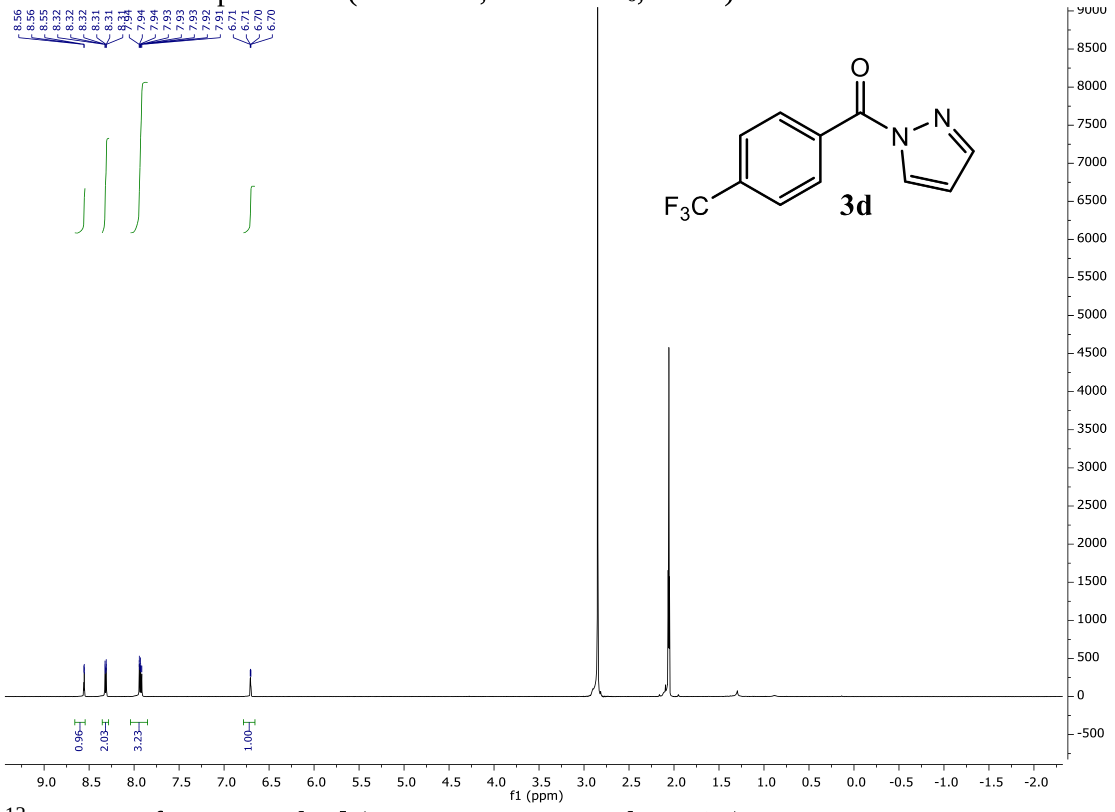

^{13}C NMR of compound **3d** (151 MHz, Acetone d6, 25 ºC)

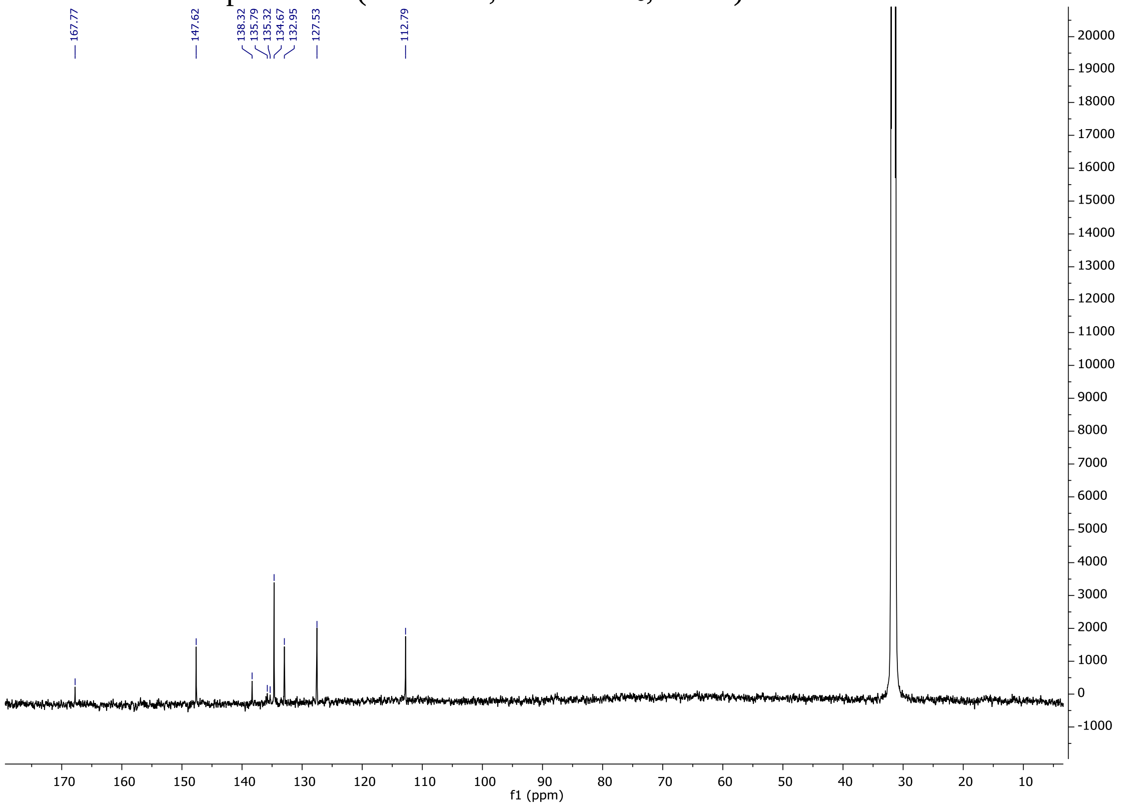

HSQC of compound **3d**

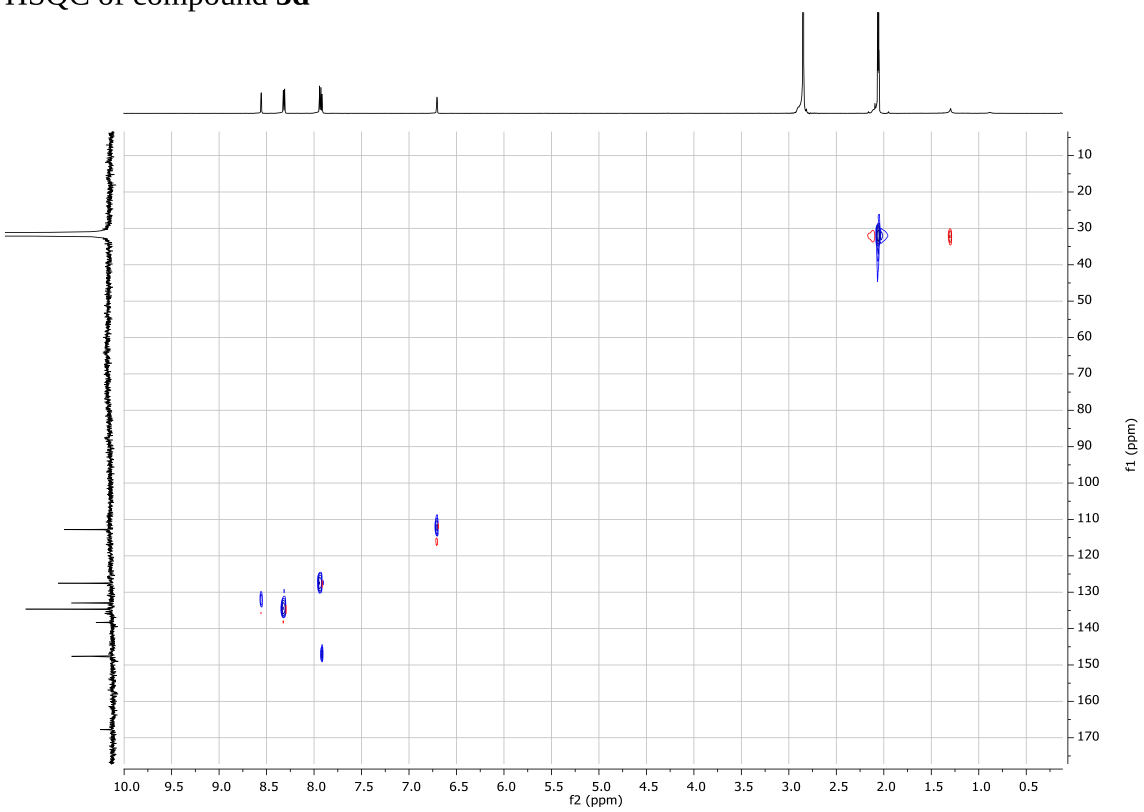

HMBC of compound **3d**

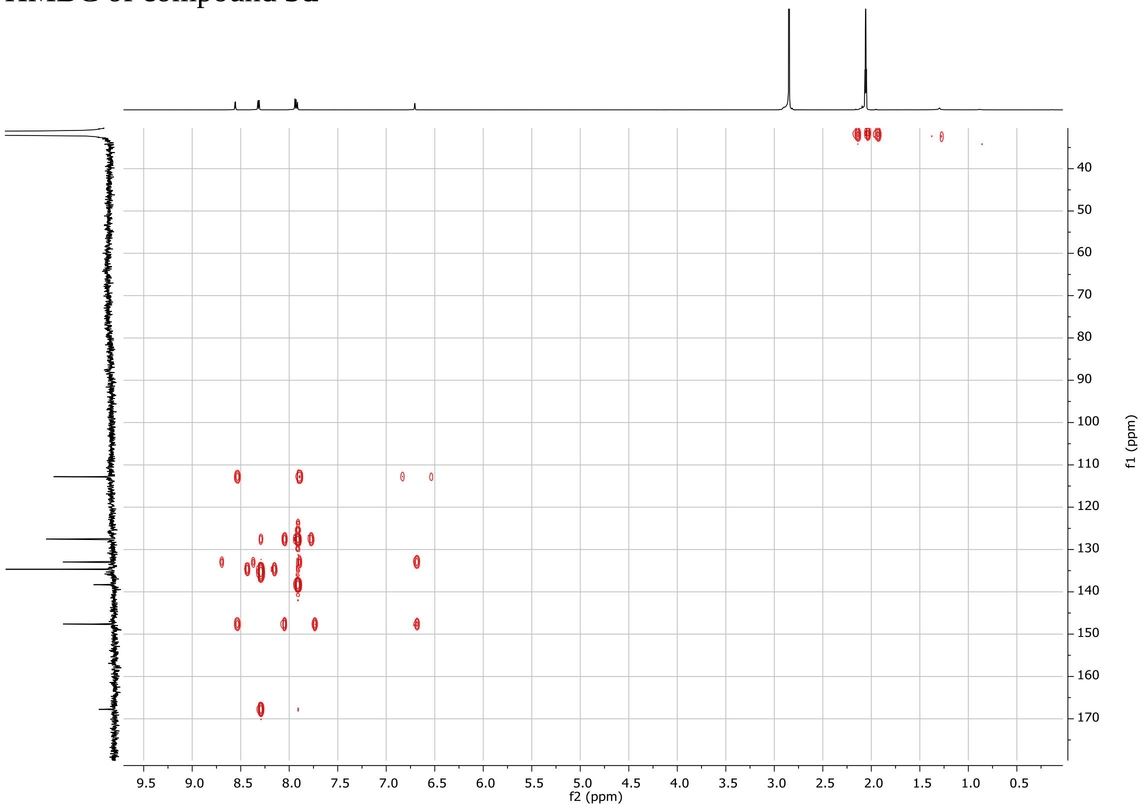

^{1}H NMR of compound **3e** (600 MHz, $CDCl_3$, 25 ºC)

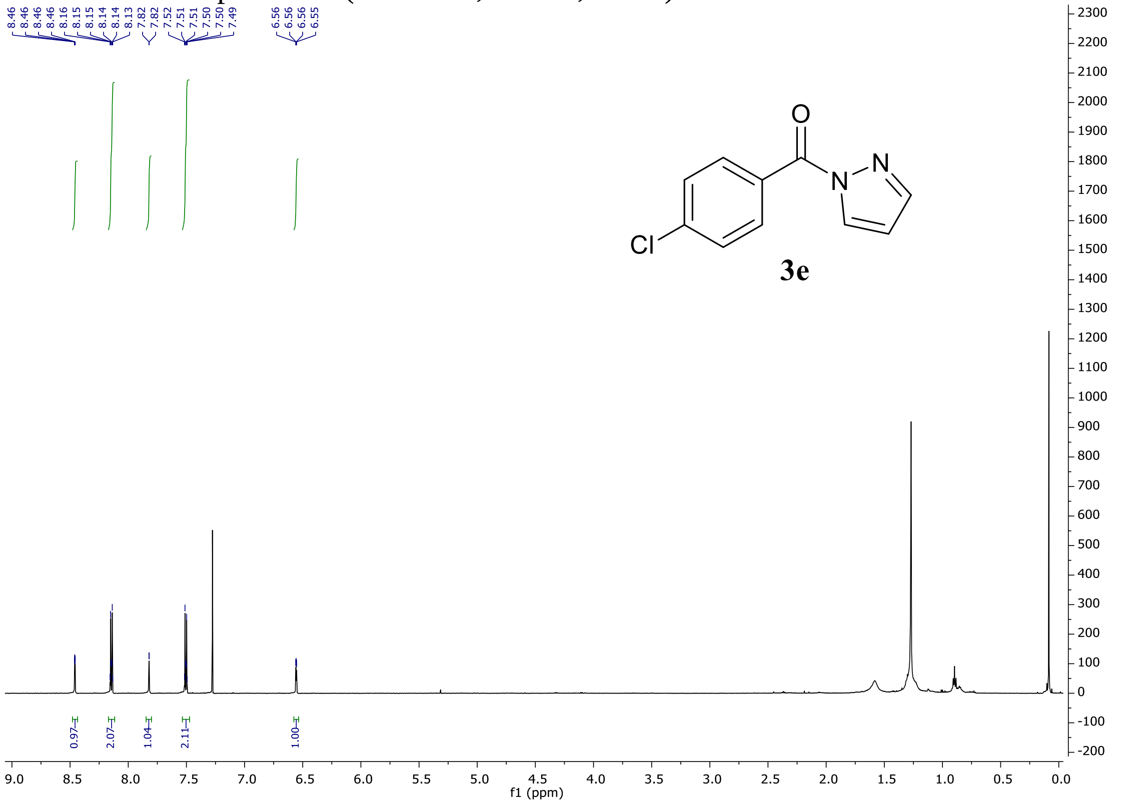

^{13}C NMR of compound **3e** (151 MHz, $CDCl_3$, 25 ºC)

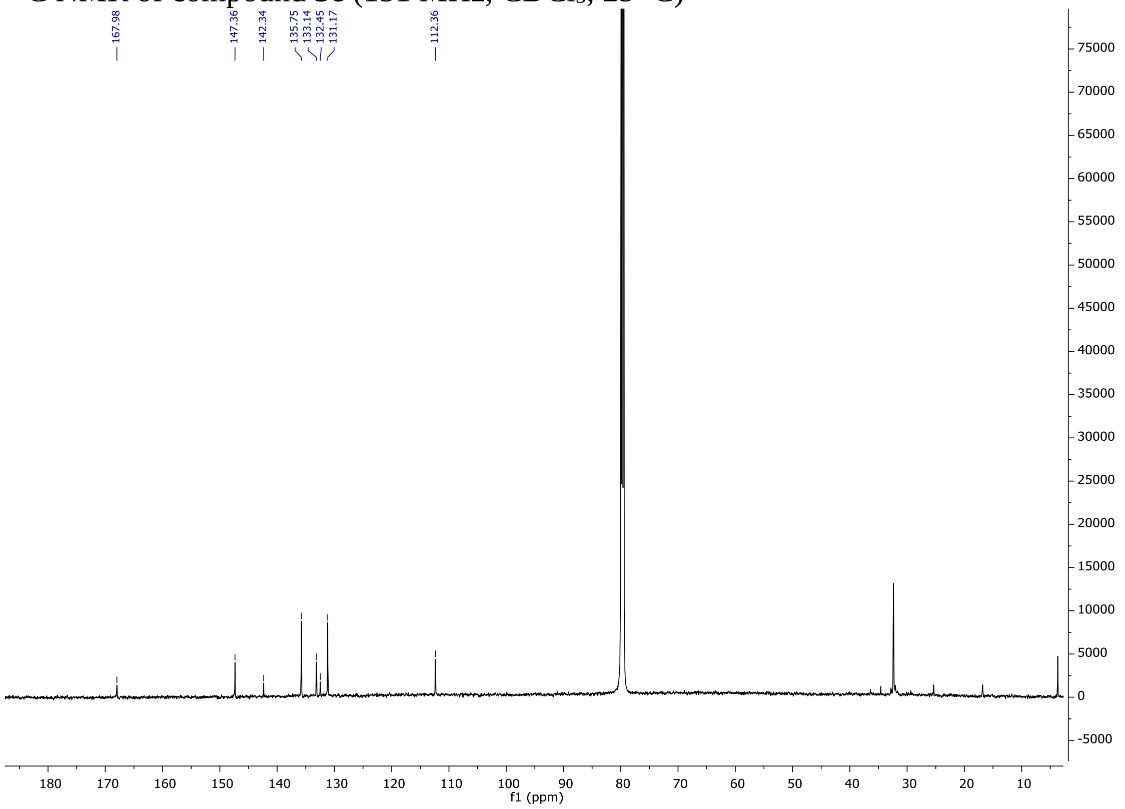

HSQC of compound **3e**

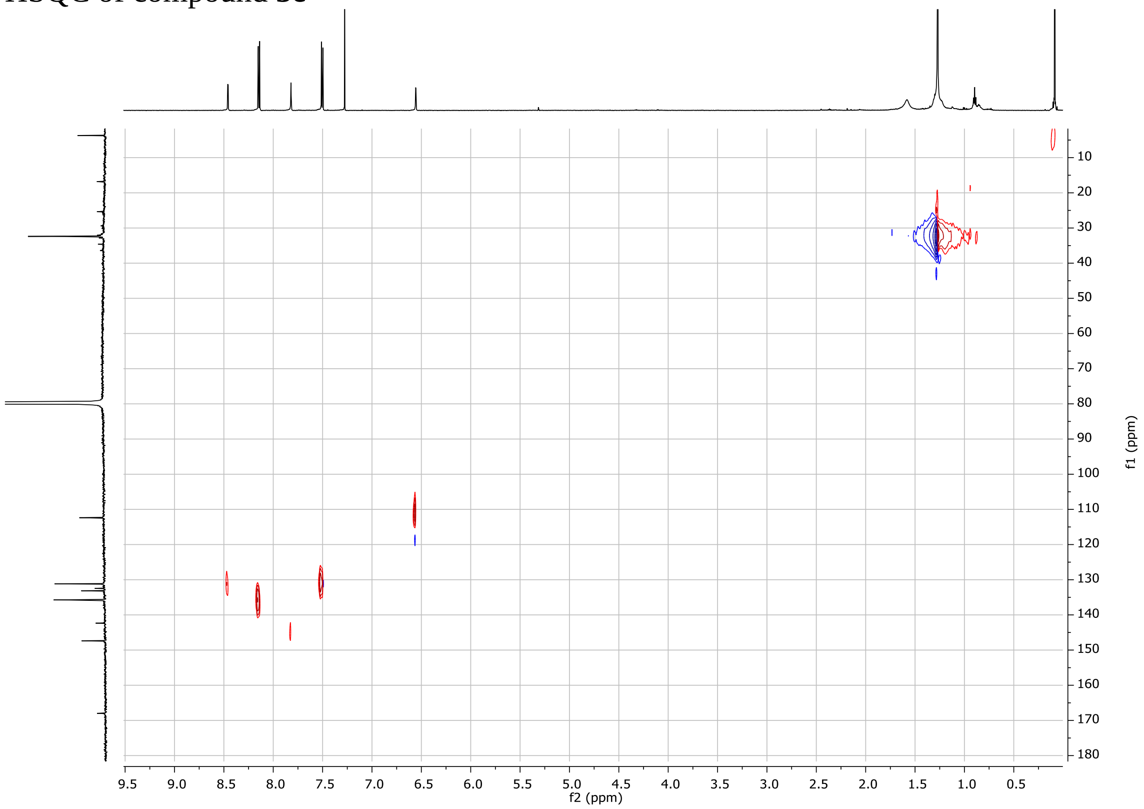

HMBC of compound **3e**

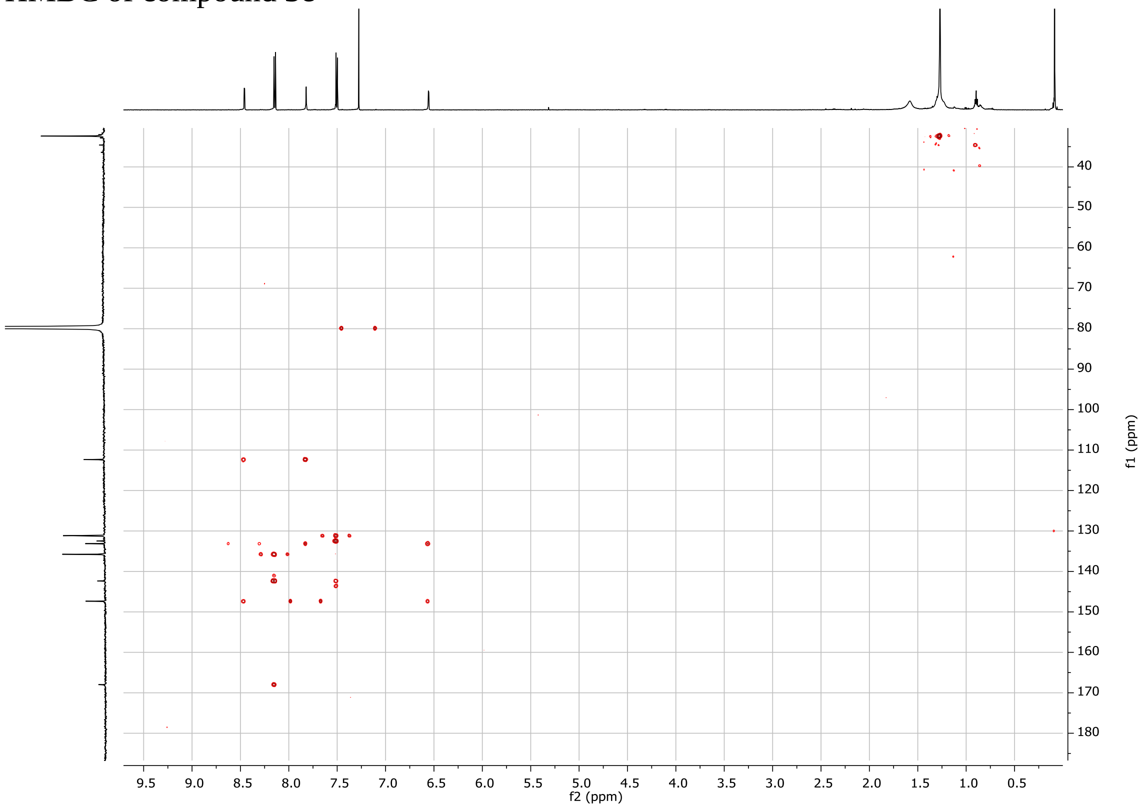

^{1}H NMR of compound **3f** (600 MHz, $CDCl_3$, 25 ºC)

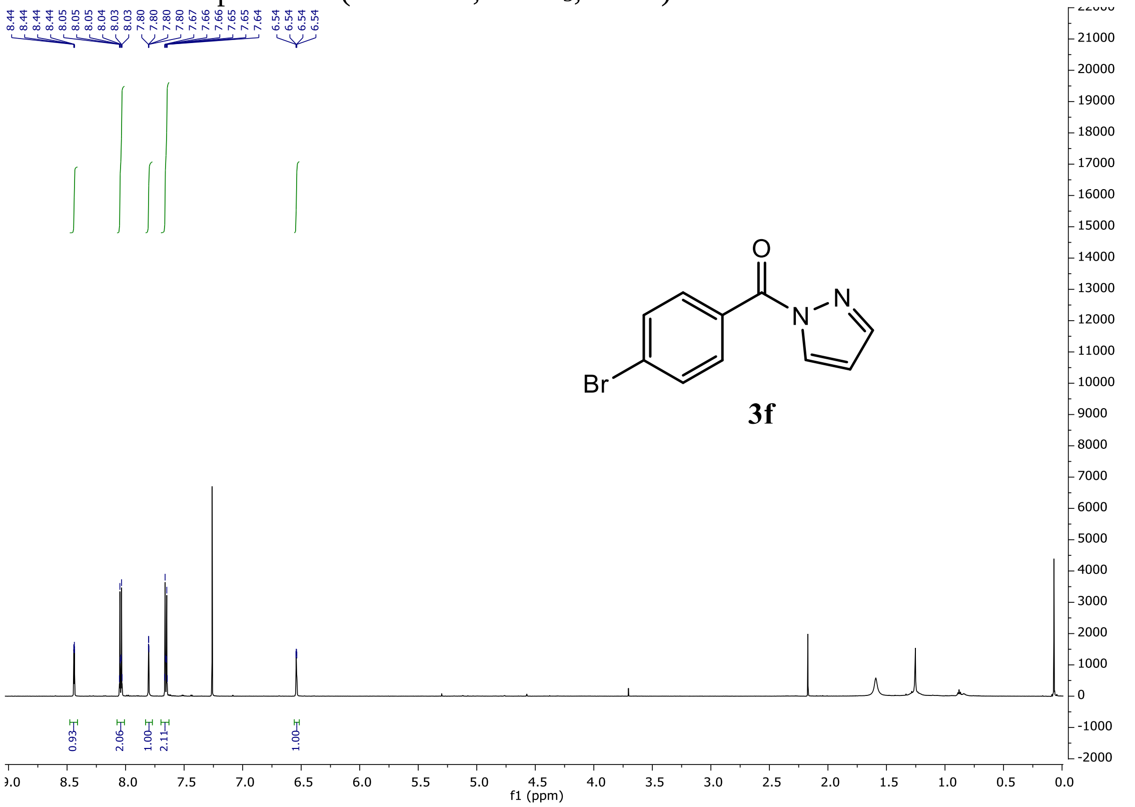

^{13}C NMR of compound **3f** (151 MHz, $CDCl_3$, 25 ºC)

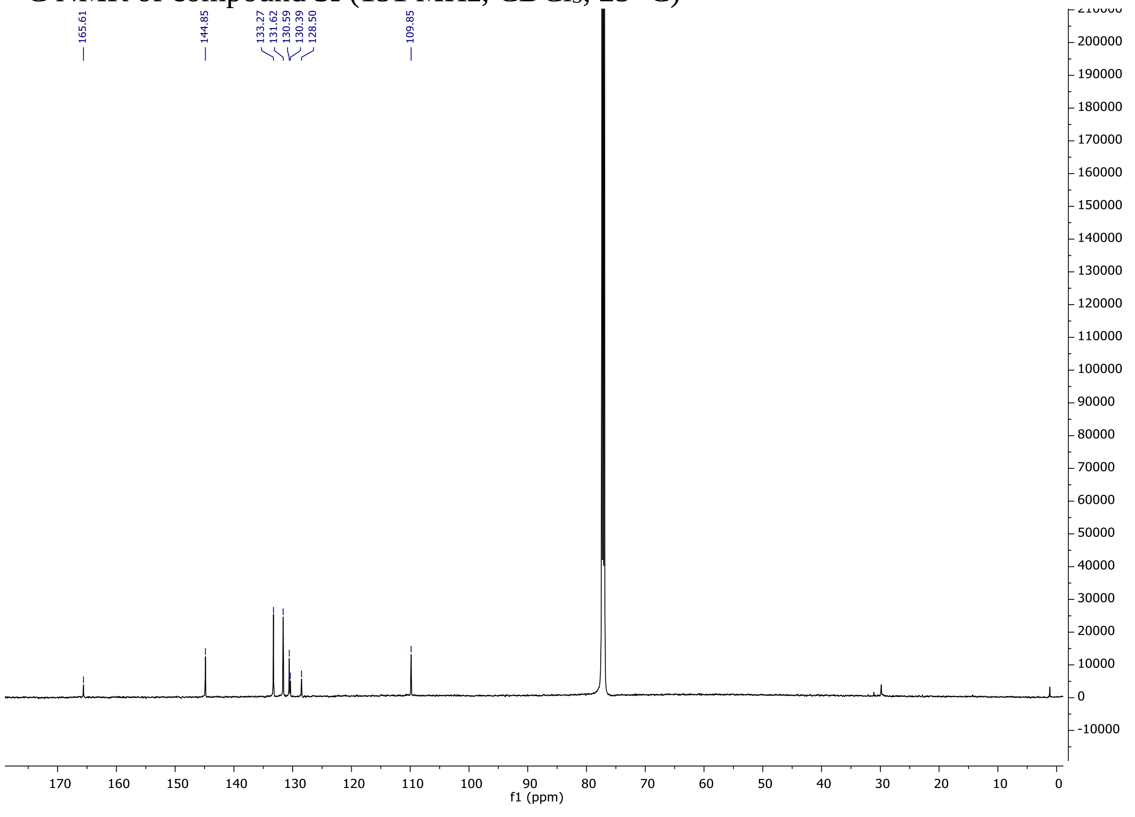

HSQC of compound **3f**

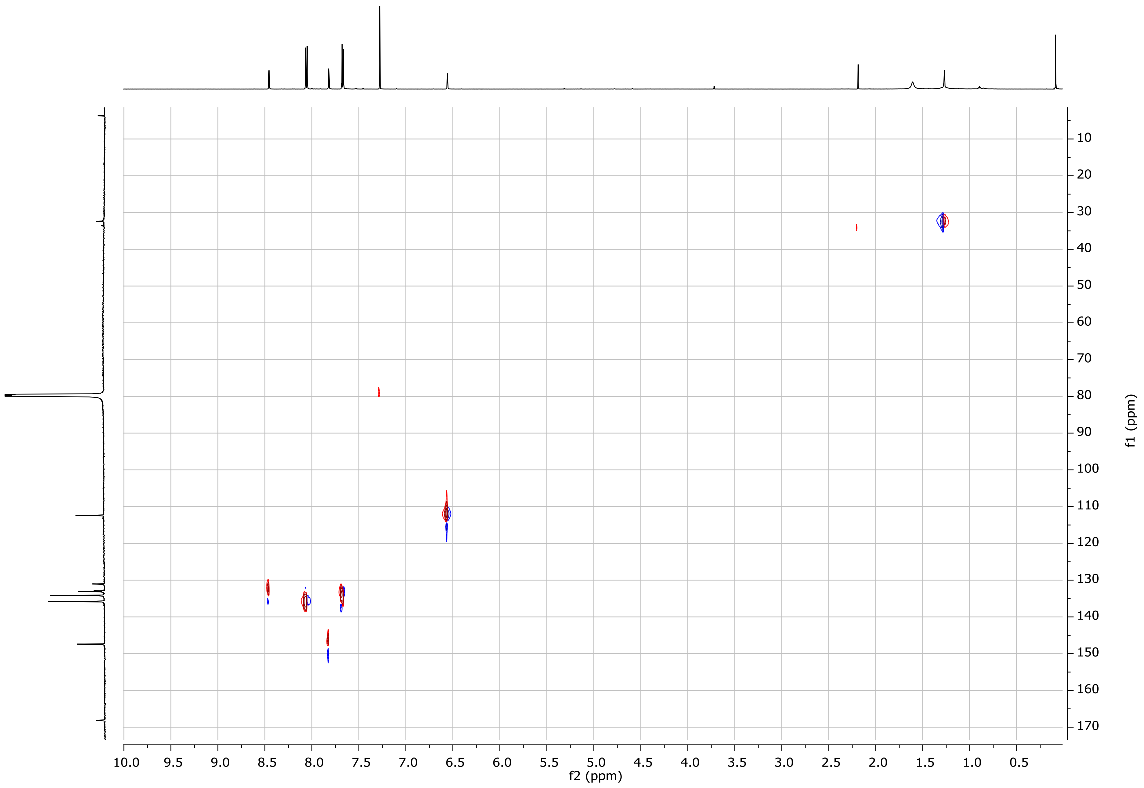

HMBC of compound **3f**

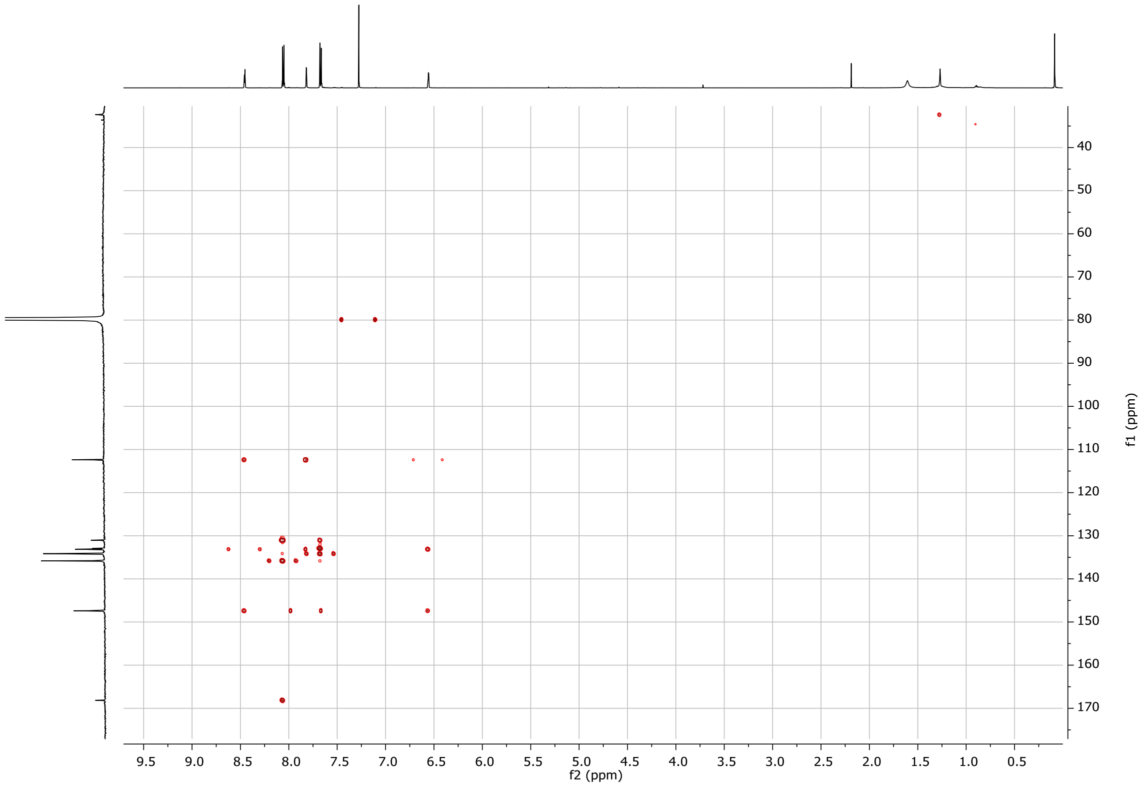

^{1}H NMR of compound **3h** (600 MHz, $CDCl_3$, 25 ºC)

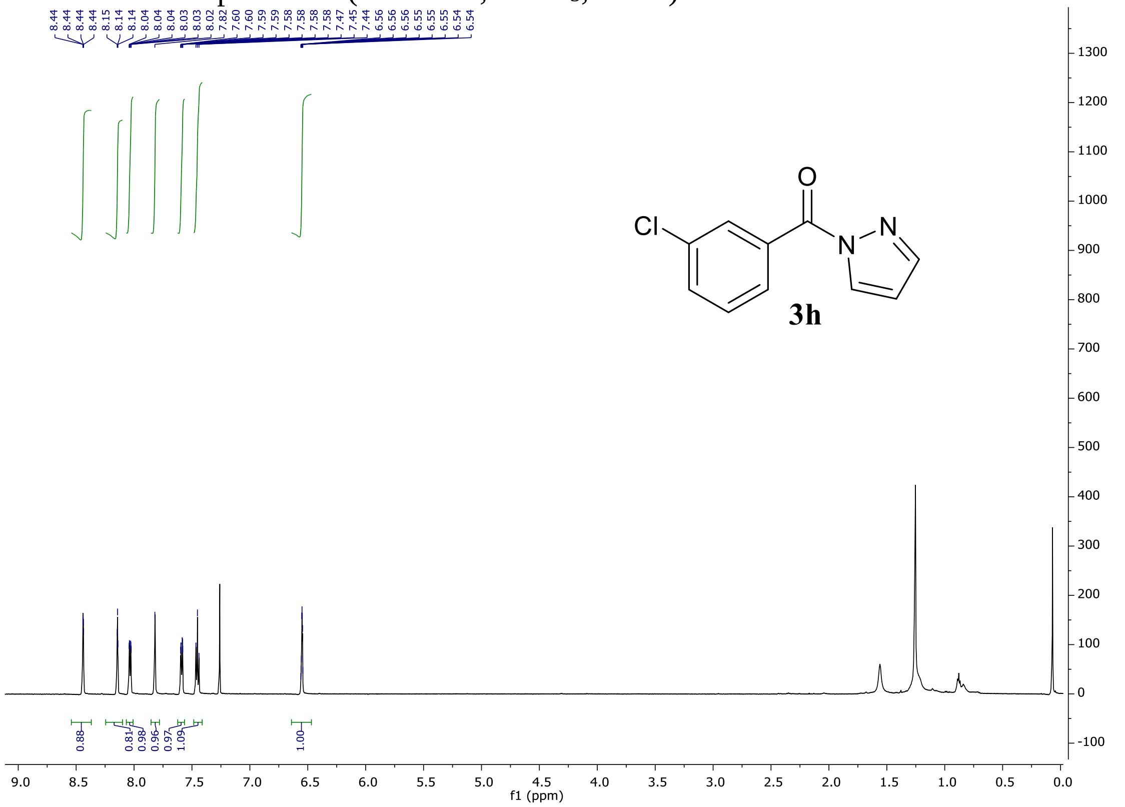

^{13}C NMR of compound **3h** (151 MHz, $CDCl_3$, 25 ºC)

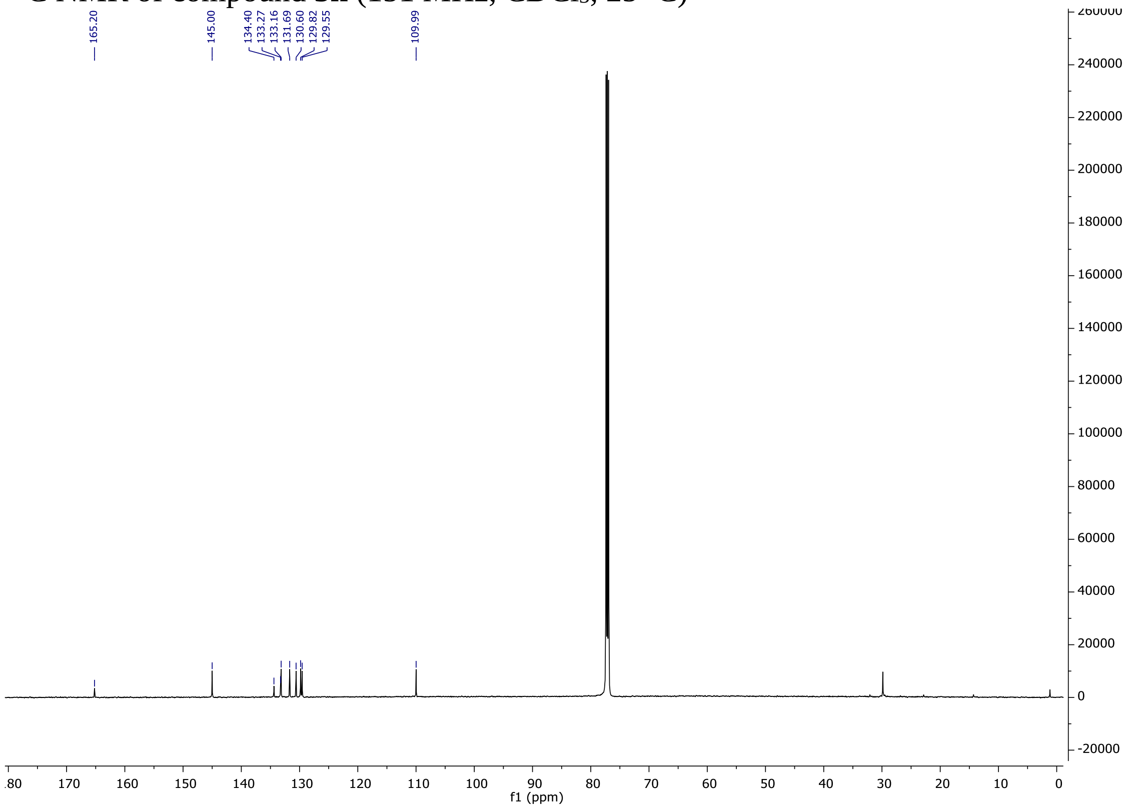

HSQC of compound **3h**

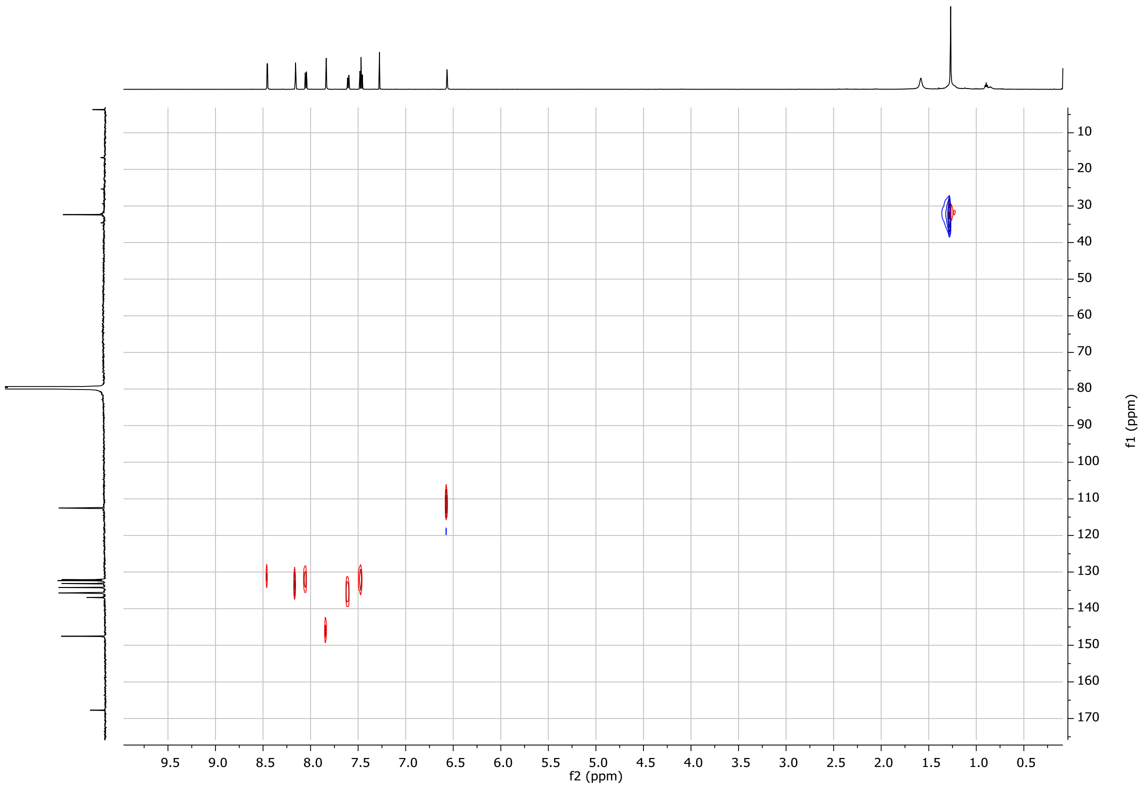

HMBC of compound **3h**

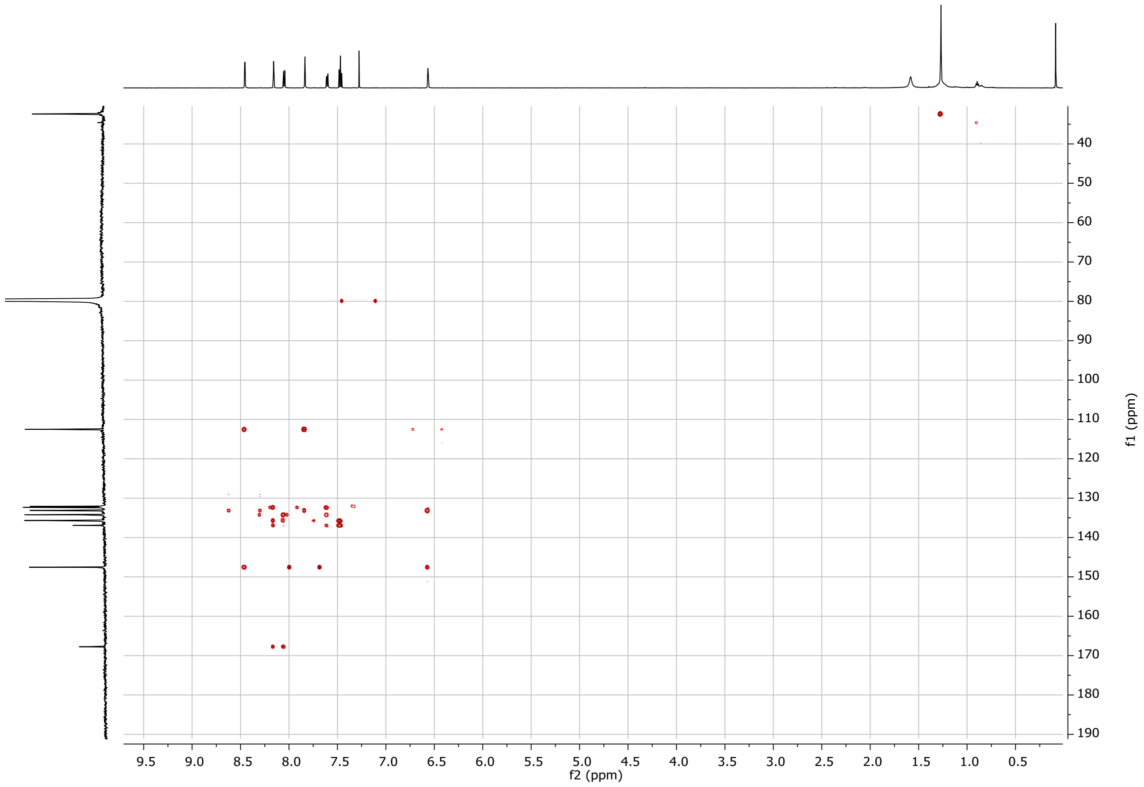

^{1}H NMR of compound **3i** (600 MHz, $CDCl_3$, 25 ºC)

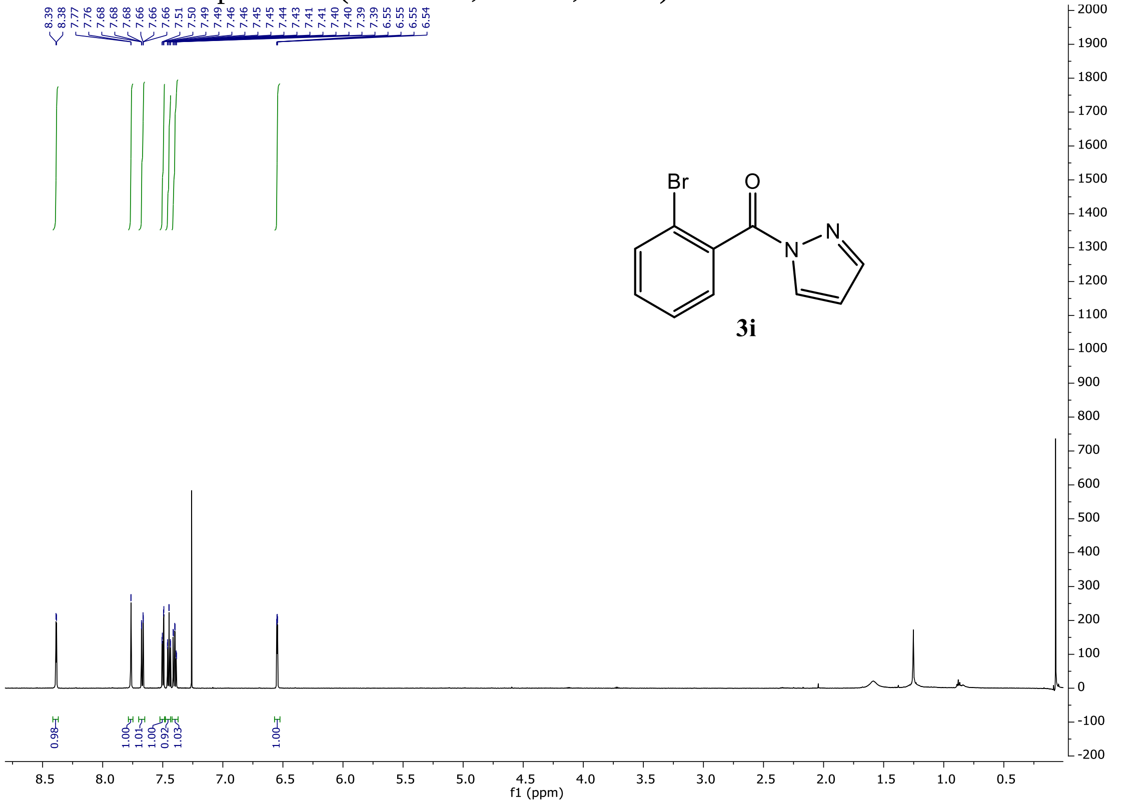

^{13}C NMR of compound **3i** (151 MHz, $CDCl_3$, 25 ºC)

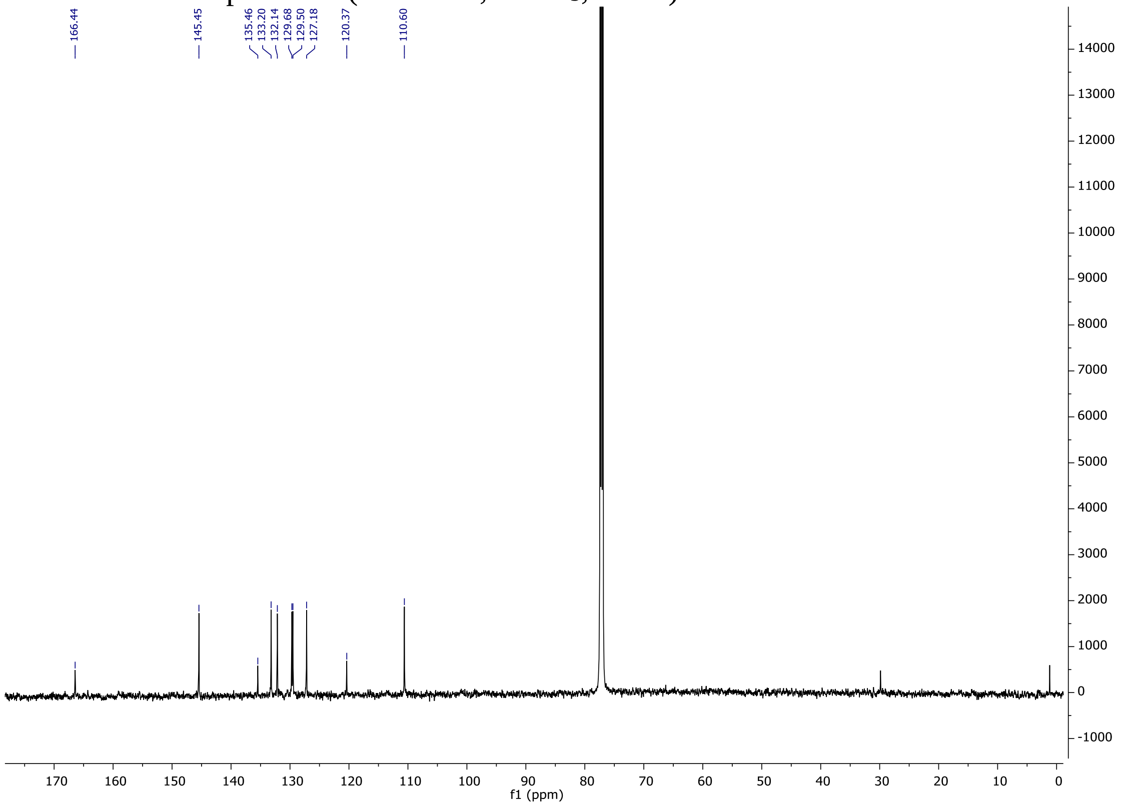

HSQC of compound **3i**

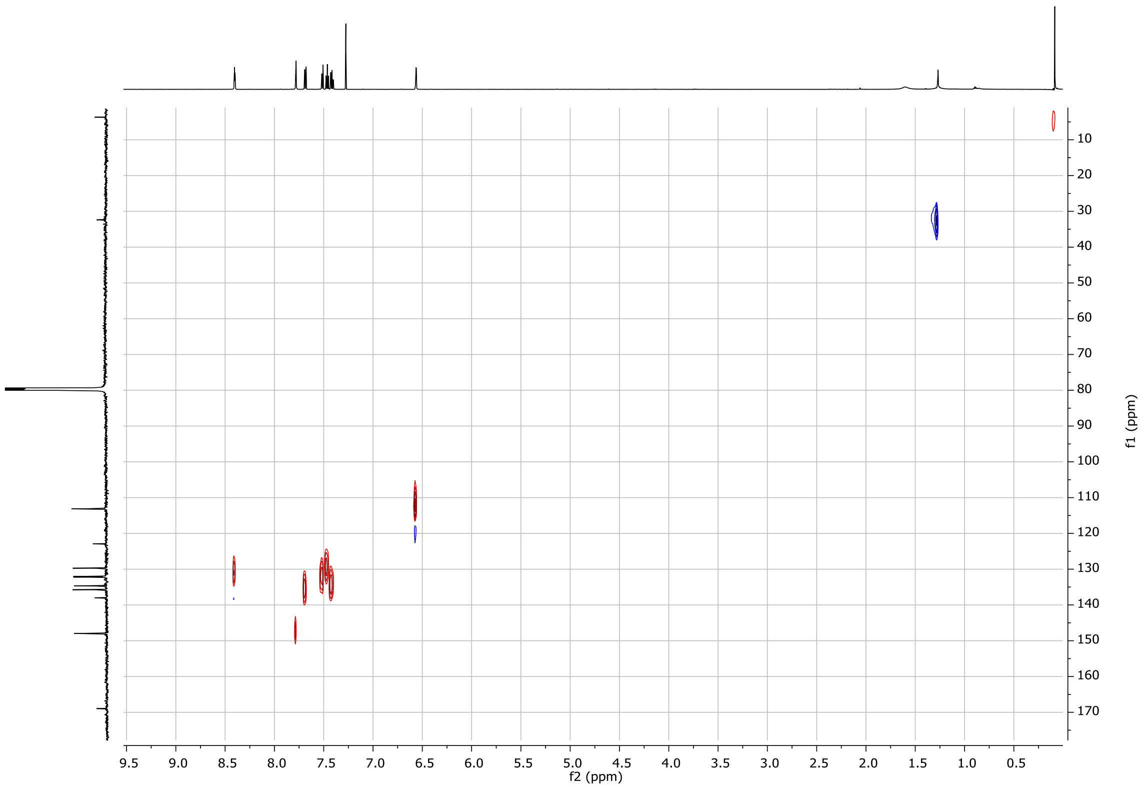

HMBC of compound **3i**

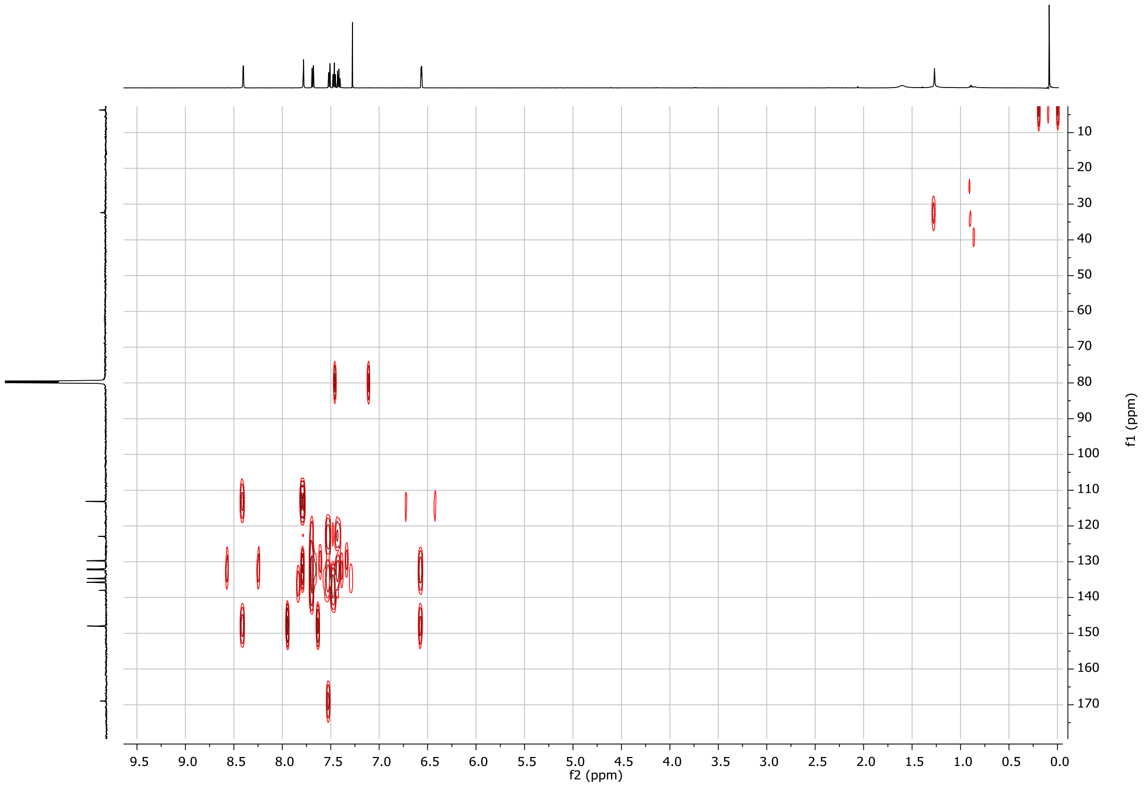

^{1}H NMR of compound **3j** (600 MHz, $CDCl_3$, 25 ºC)

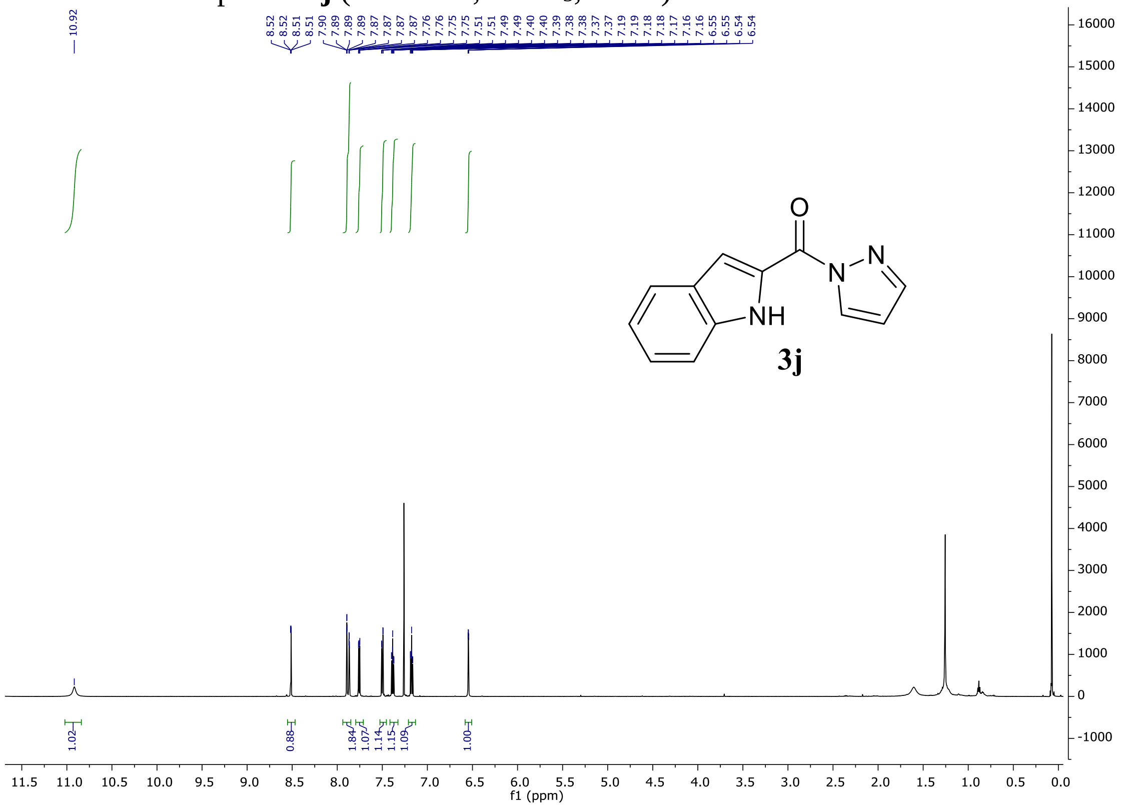

^{13}C NMR of compound **3j** (151 MHz, $CDCl_3$, 25 ºC)

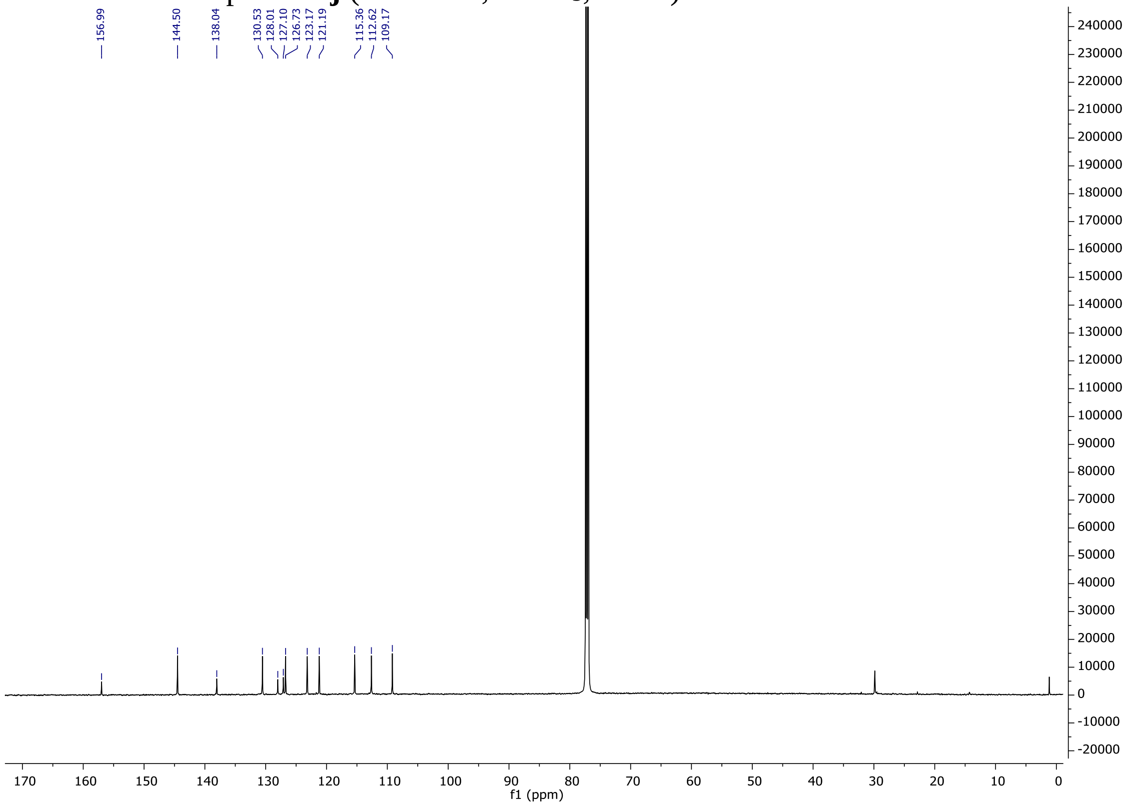

HSQC of compound **3j**

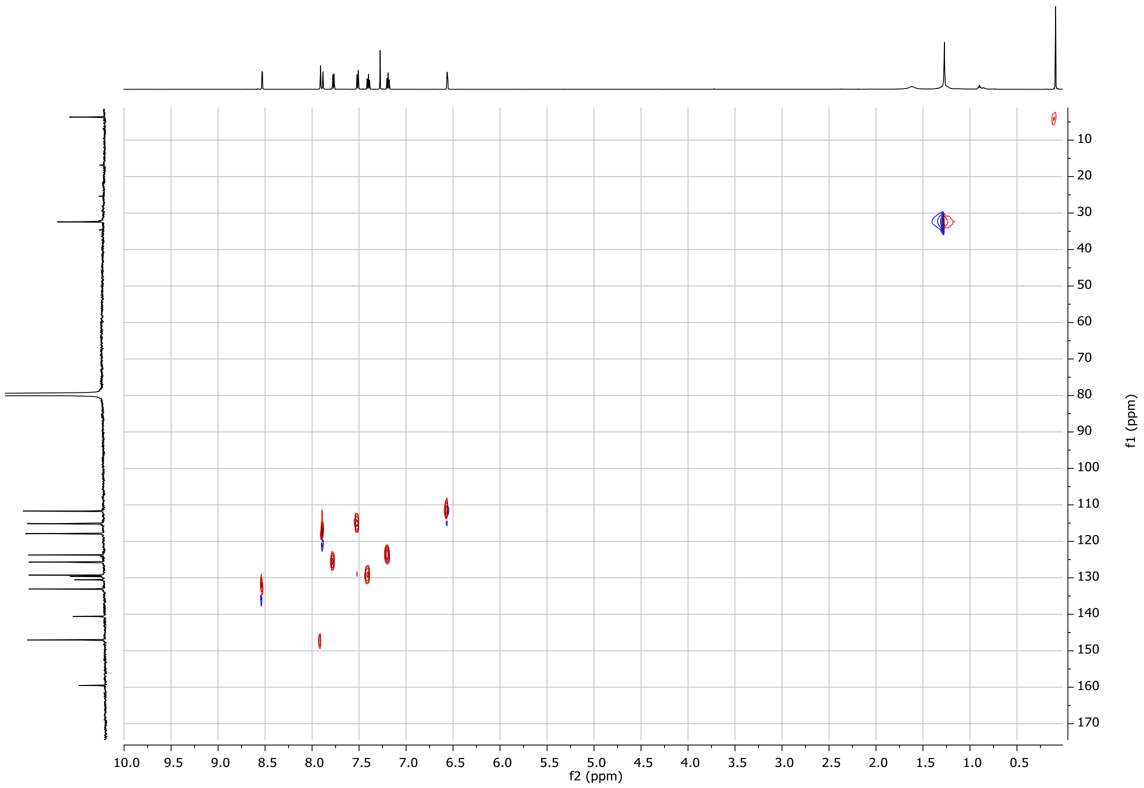

HMBC of compound **3j**

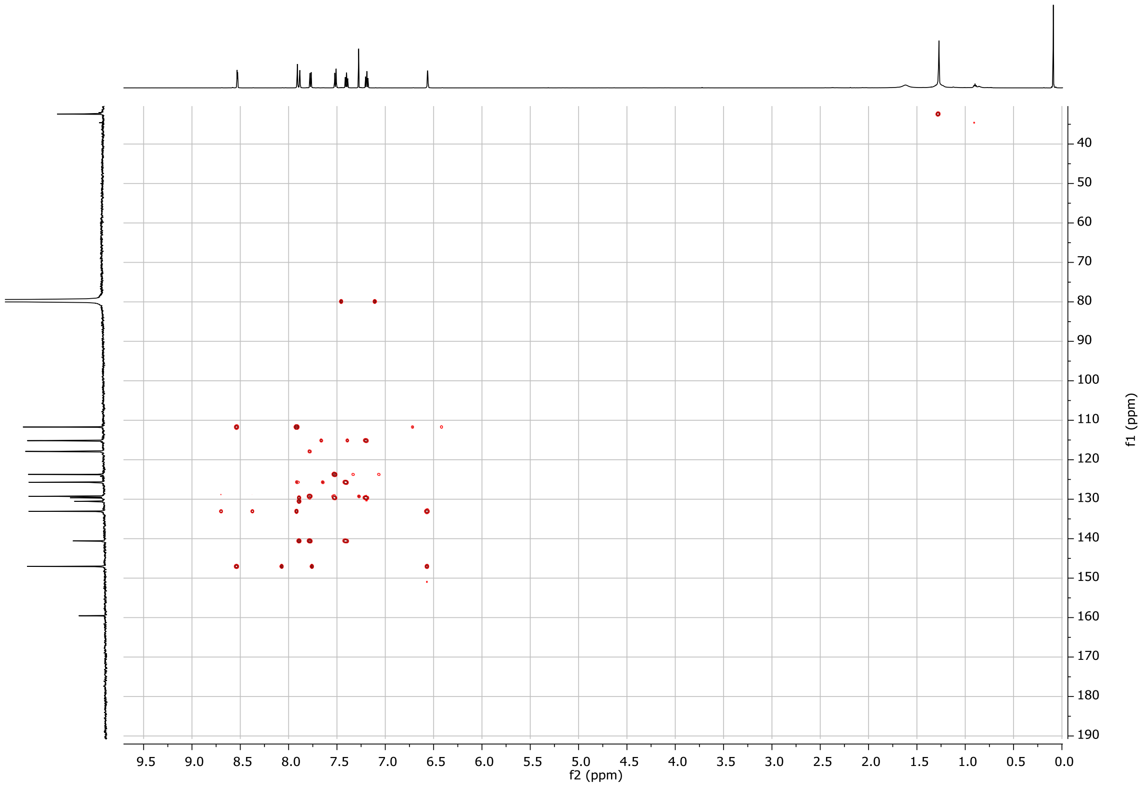

^{1}H NMR of compound **3k** (600 MHz, $CDCl_3$, 25 ºC)

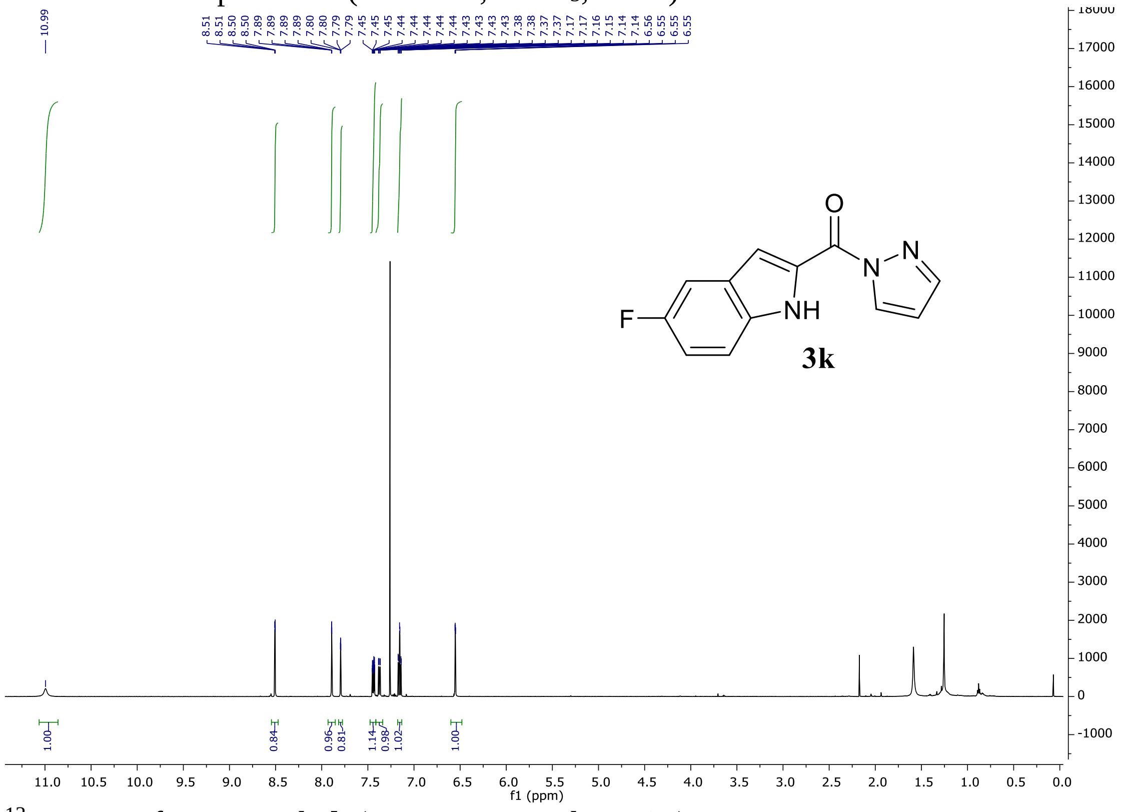

3k

^{13}C NMR of compound **3k** (151 MHz, $CDCl_3$, 25 ºC)

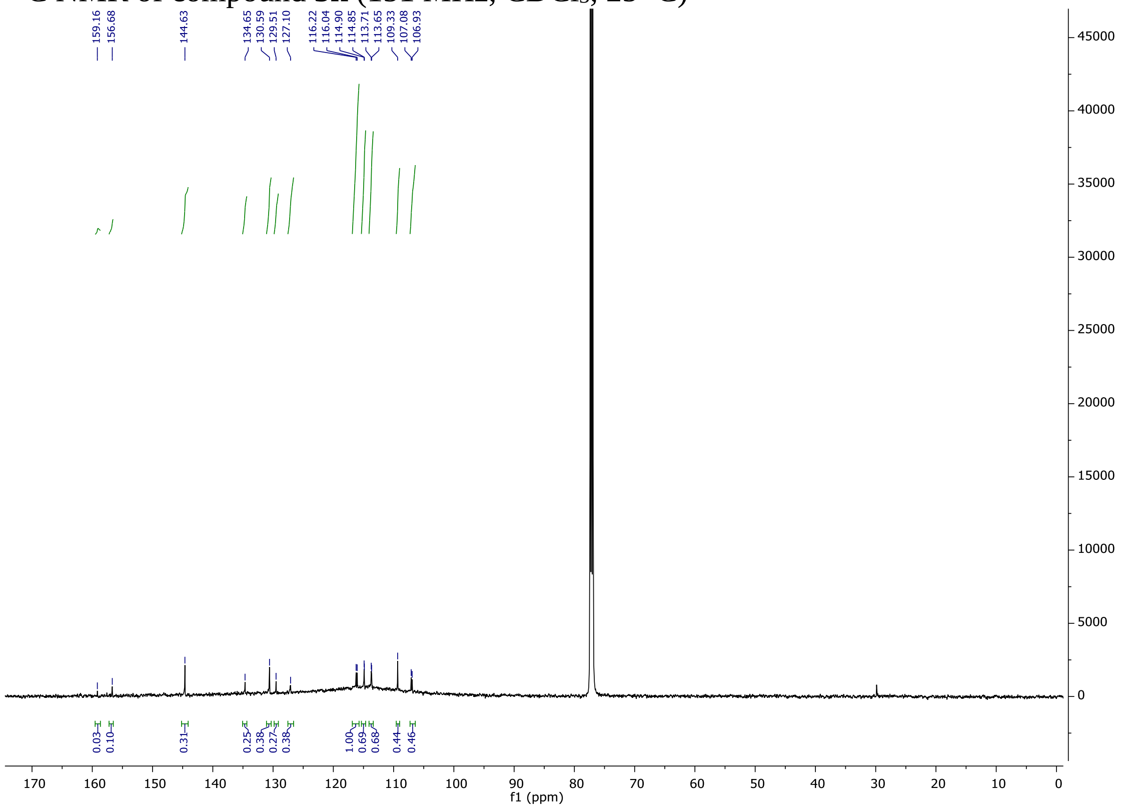

HSQC of compound **3k**

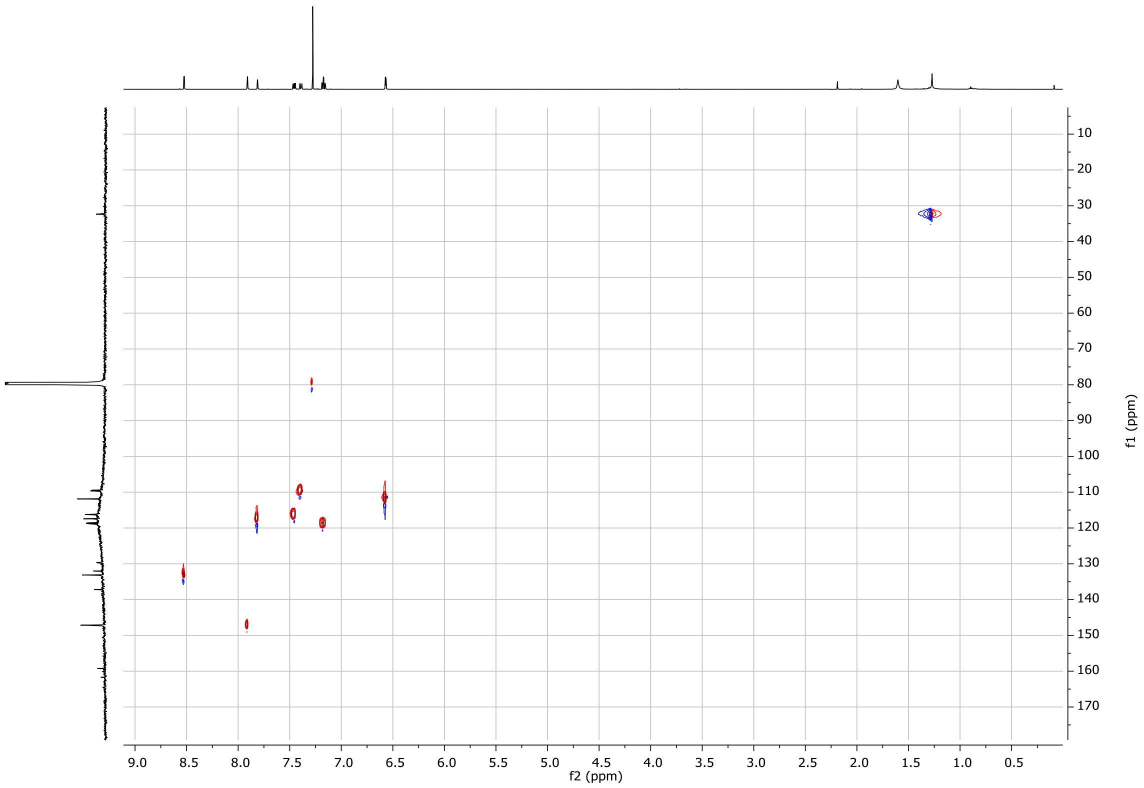

HMBC of compound **3k**

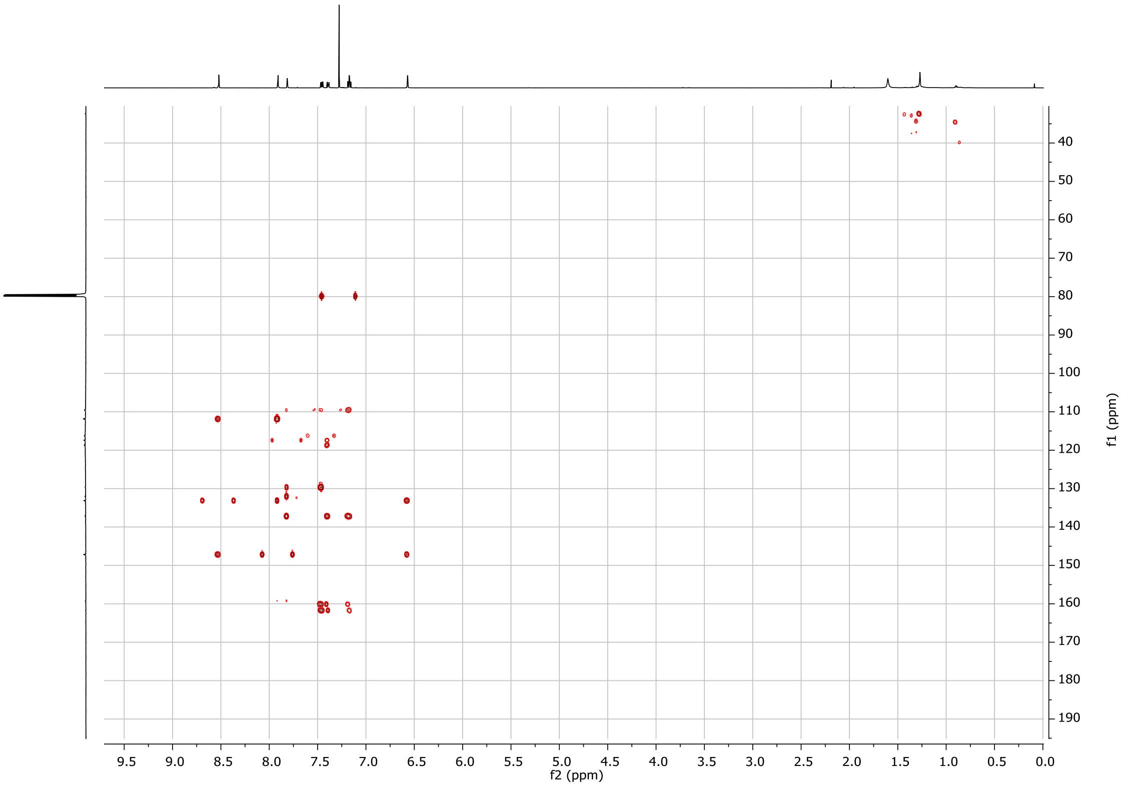

^{1}H NMR of compound **3l** (300 MHz, $CDCl_3$, 25 ºC)

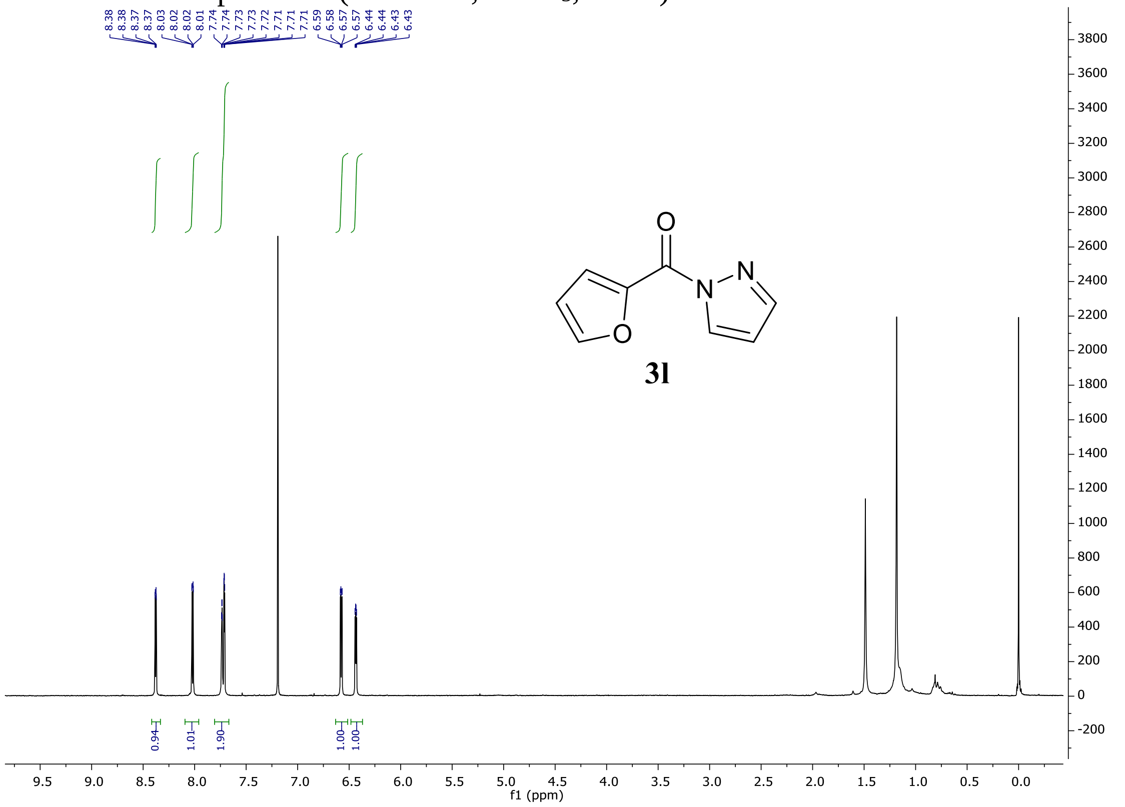

^{13}C NMR of compound **3l** (75 MHz, $CDCl_3$, 25 ºC)

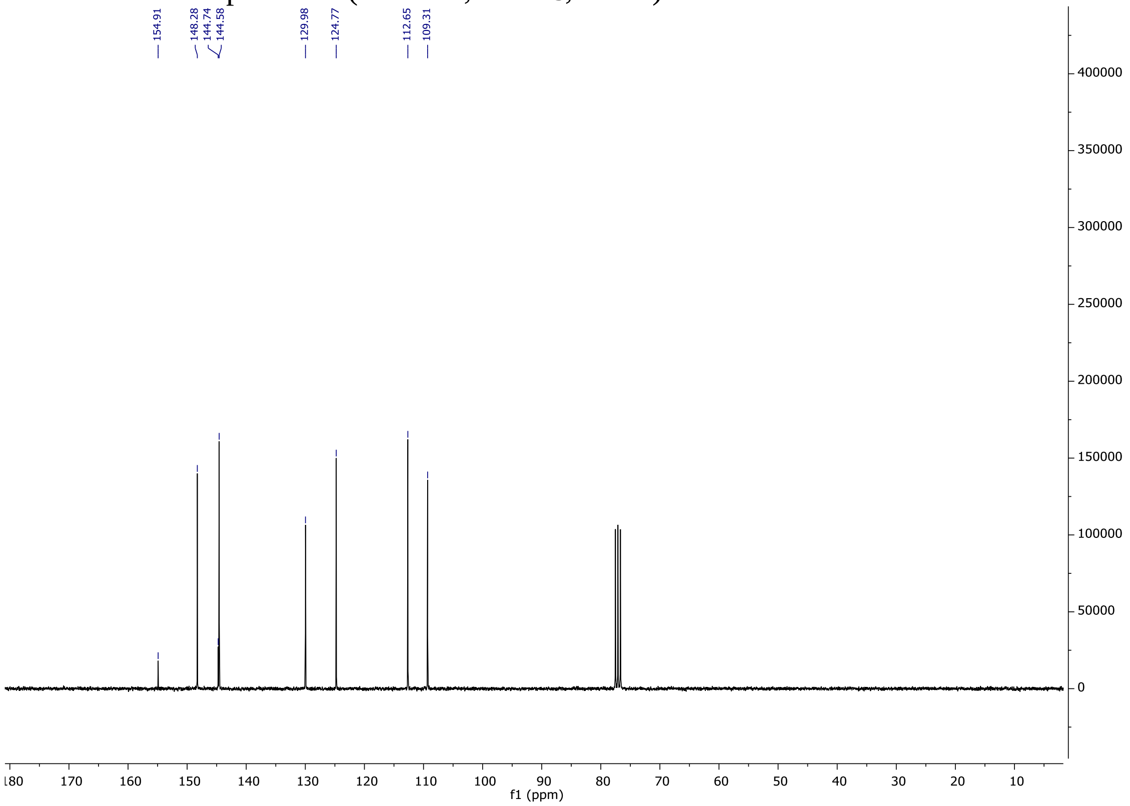

^{1}H NMR of compound **3m** (600 MHz, $CDCl_3$, 25 ºC)

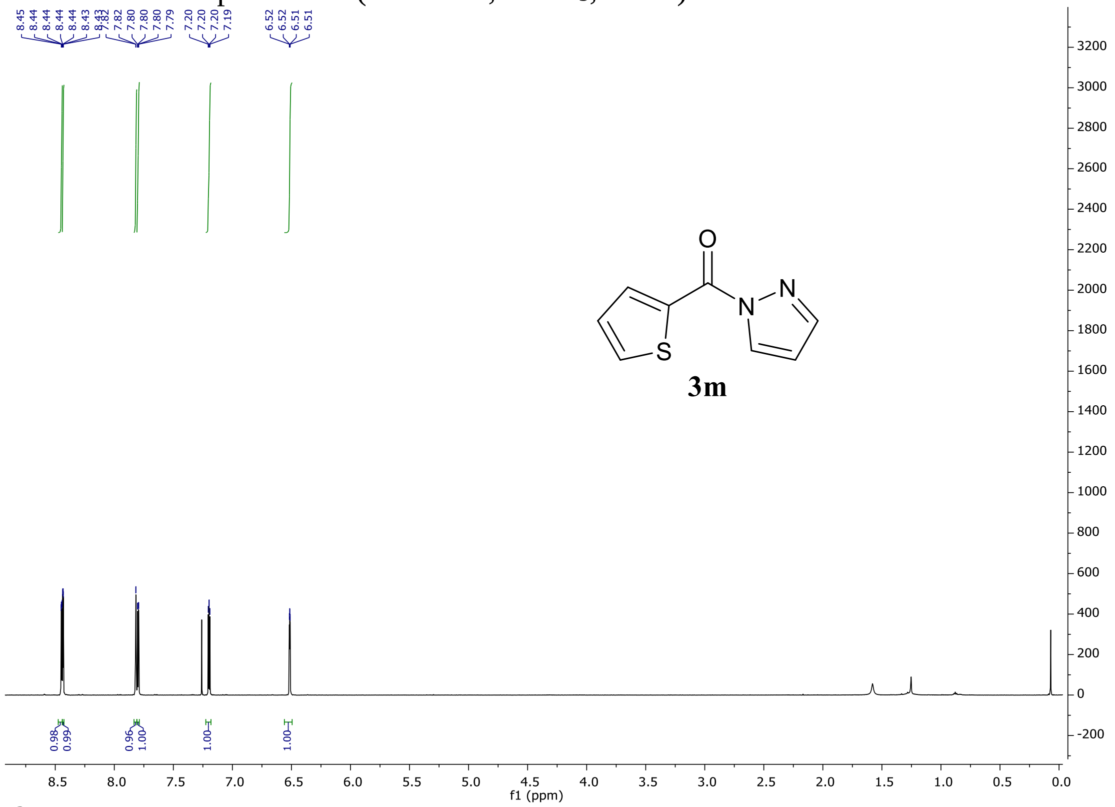

^{13}C NMR of compound **3m** (151 MHz, $CDCl_3$, 25 ºC)

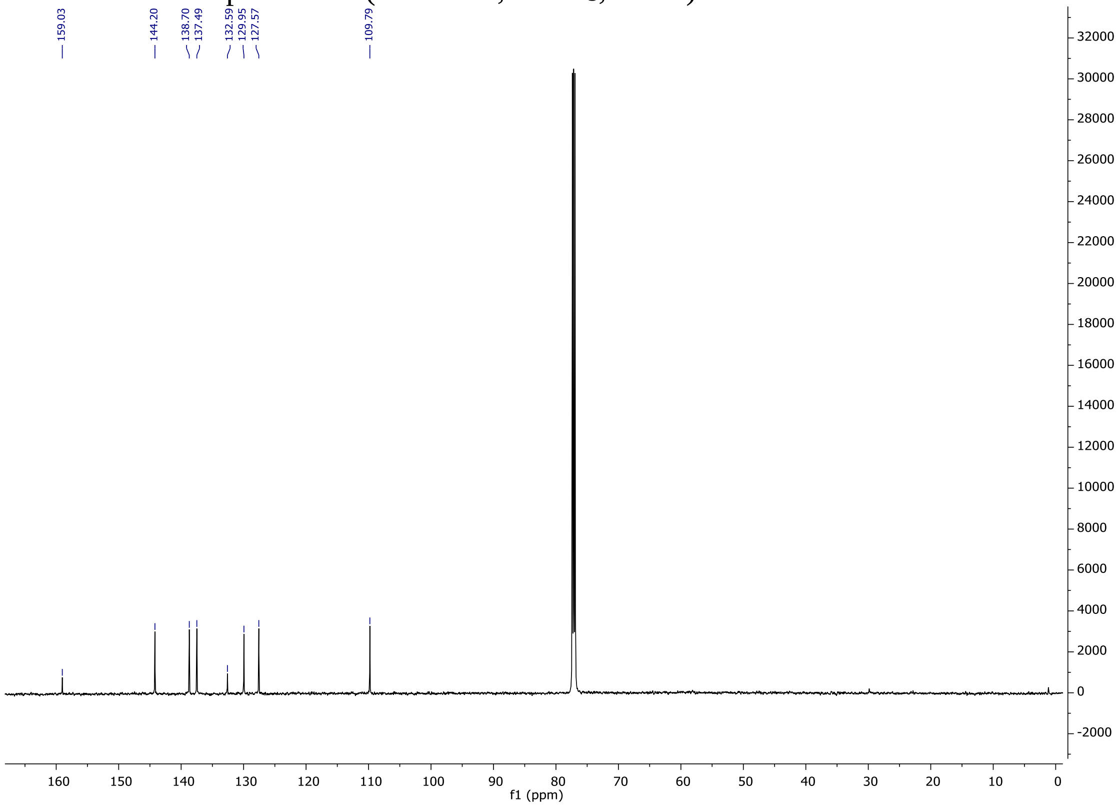

HSQC of compound **3m**

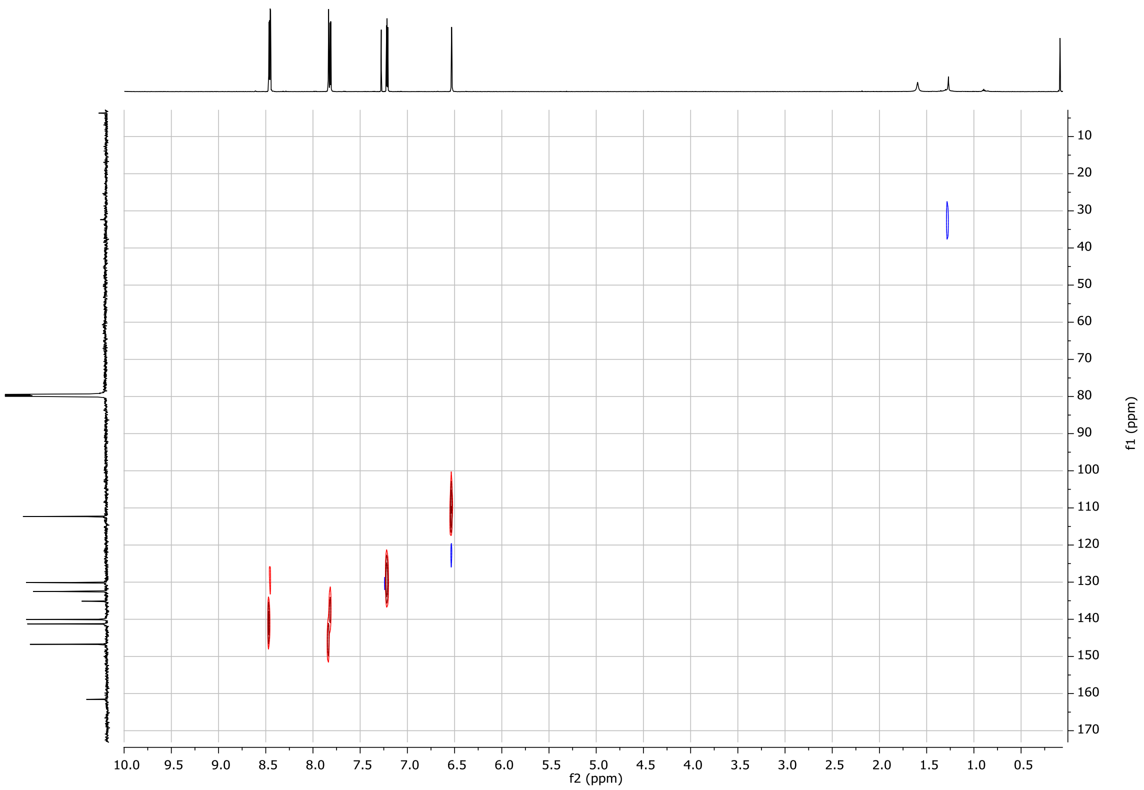

HMBC of compound **3m**

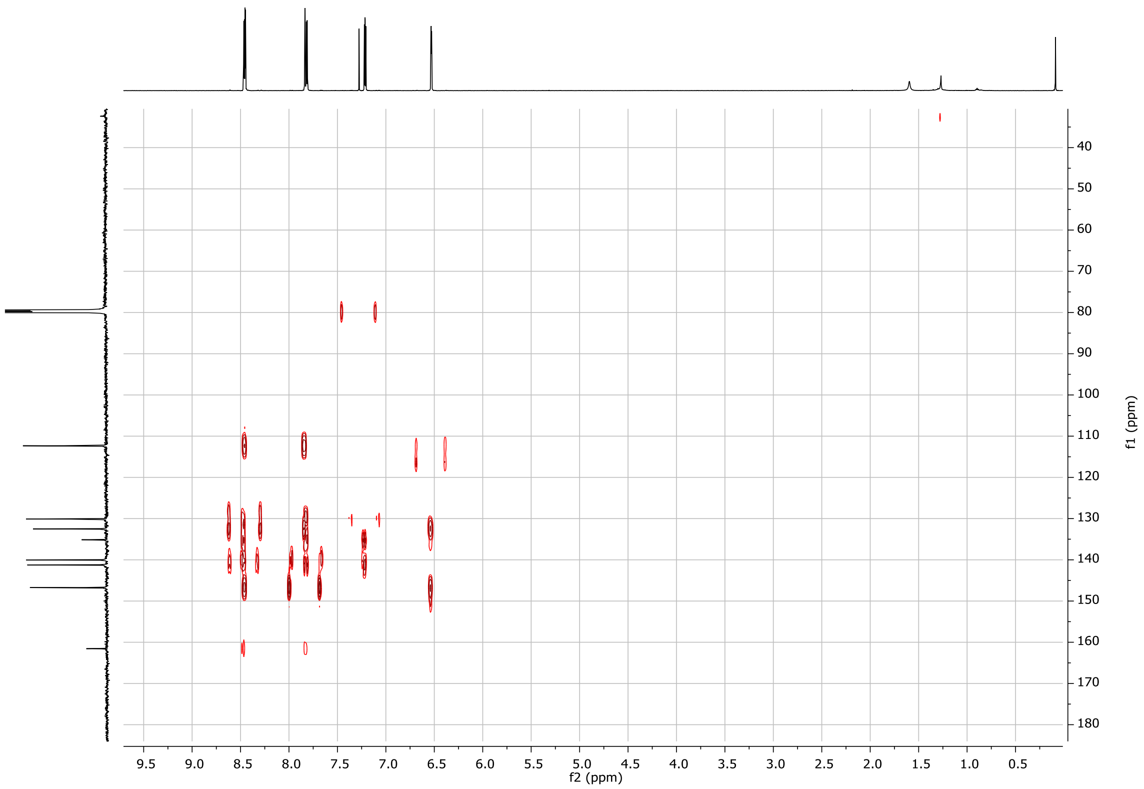

^{1}H NMR of compound **3n** (300 MHz, $CDCl_3$, 25 ºC)

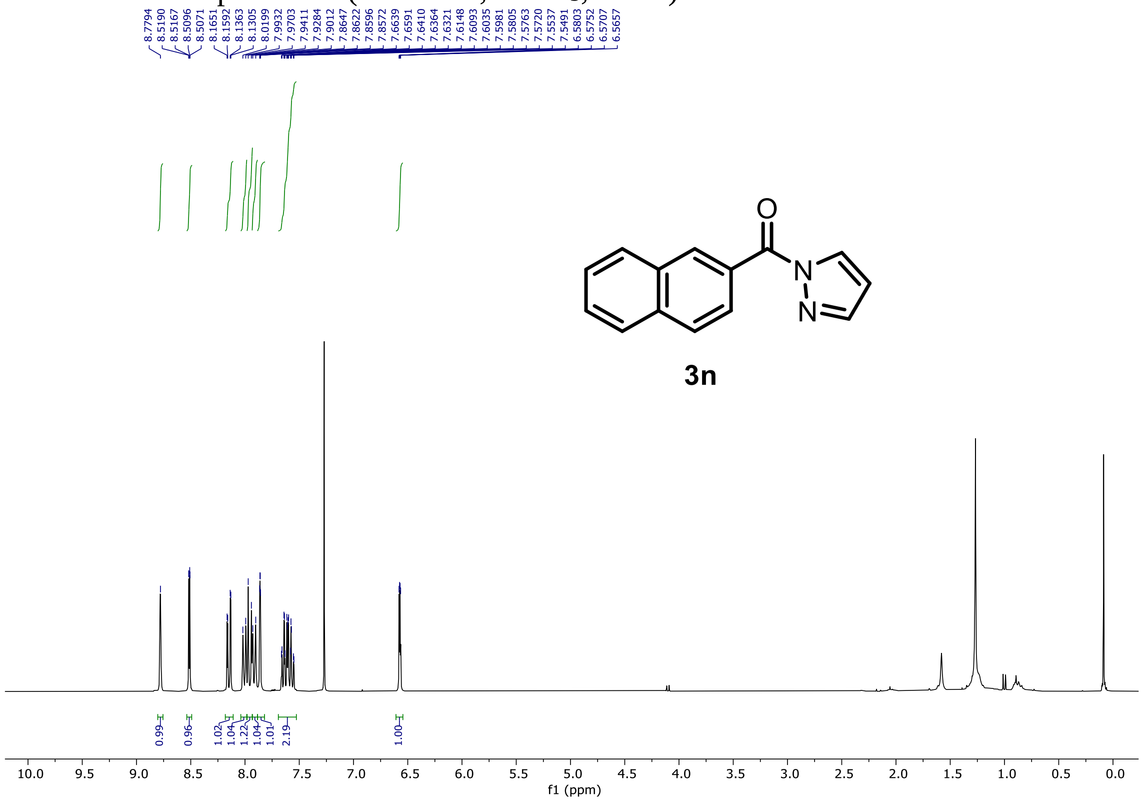

^{13}C NMR of compound **3n** (75 MHz, $CDCl_3$, 25 ºC)

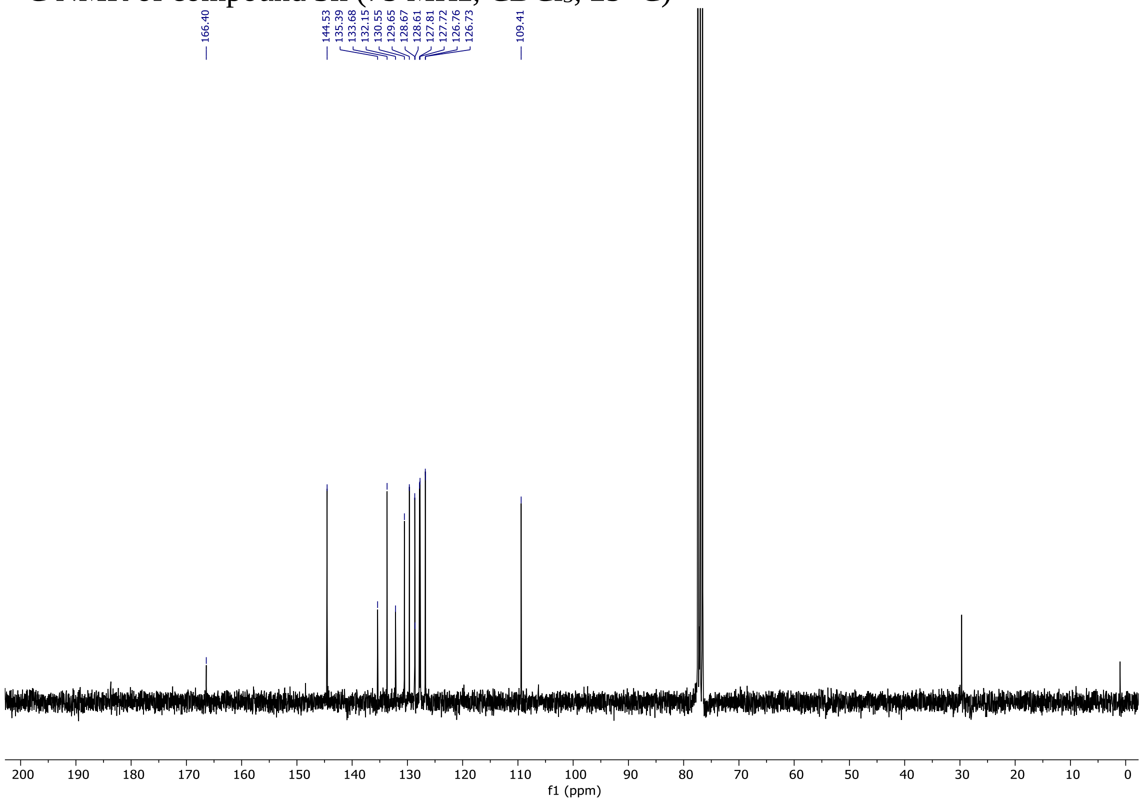

^{1}H NMR of compound **3p** (600 MHz, $CDCl_3$, 25 ºC)

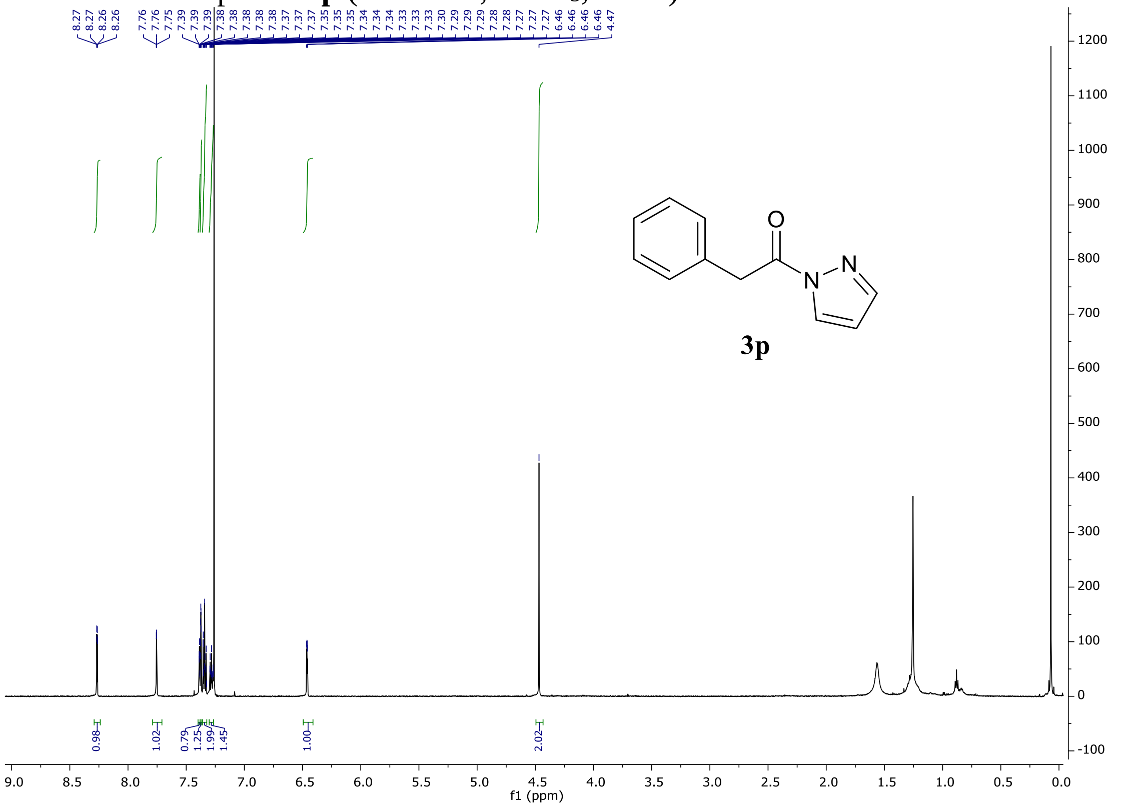

^{13}C NMR of compound **3p** (151 MHz, $CDCl_3$, 25 ºC)

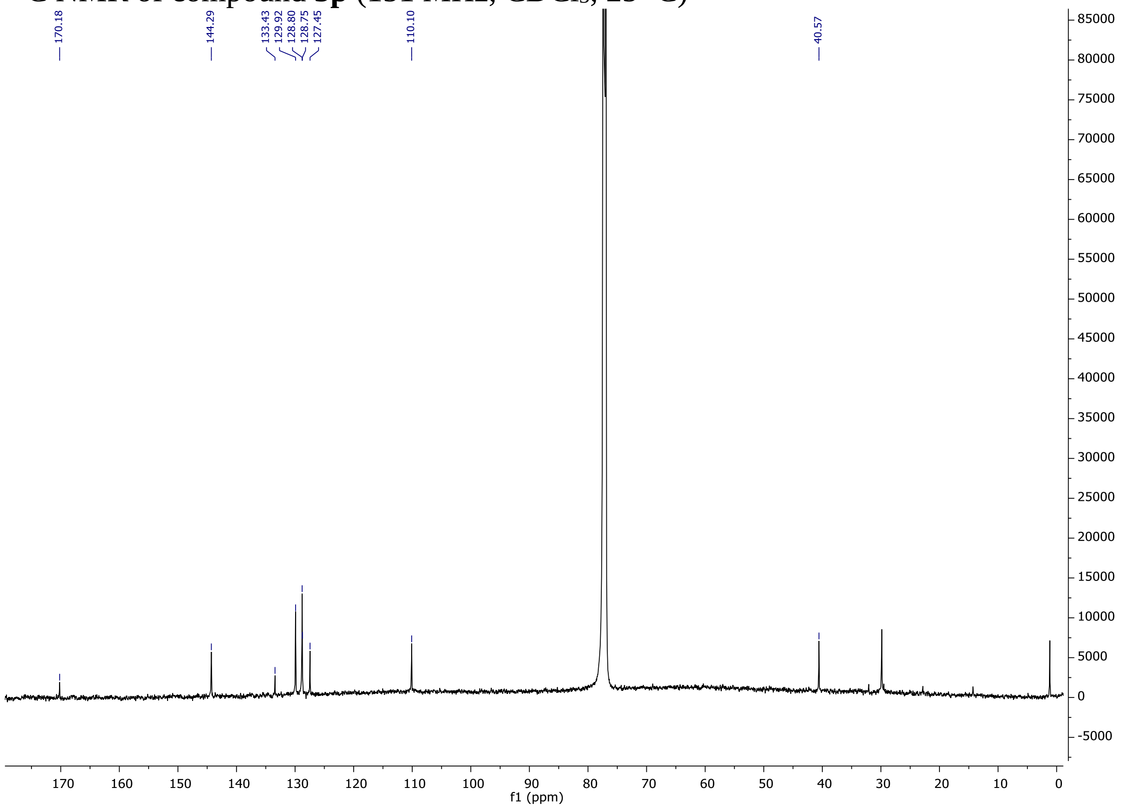

HSQC of compound **3p**

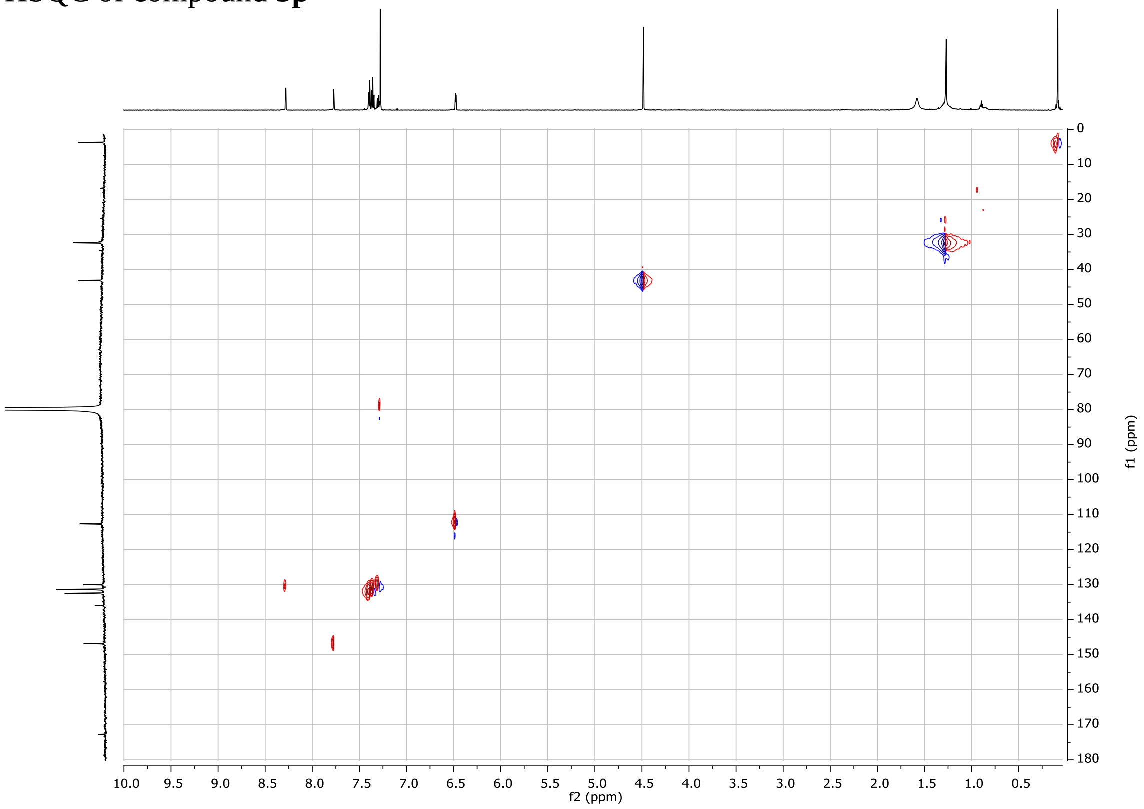

HMBC of compound **3p**

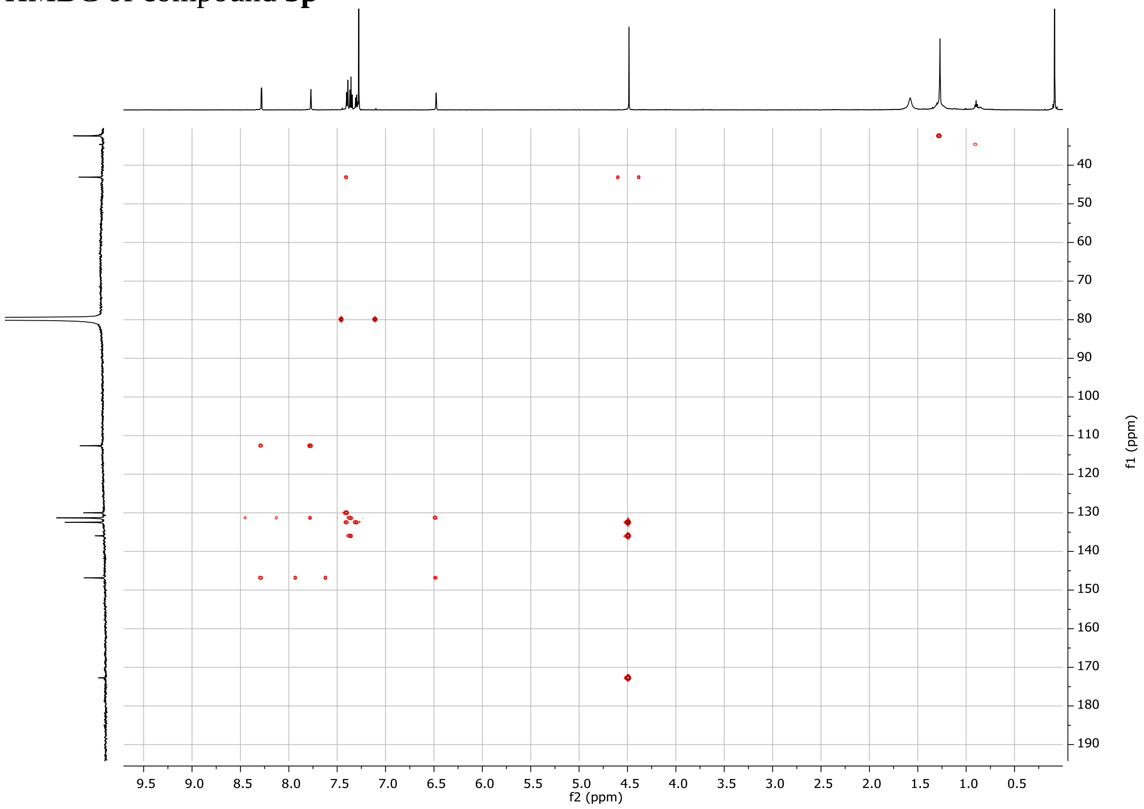

^{1}H NMR of compound **3q** (600 MHz, $CDCl_3$, 25 ºC)

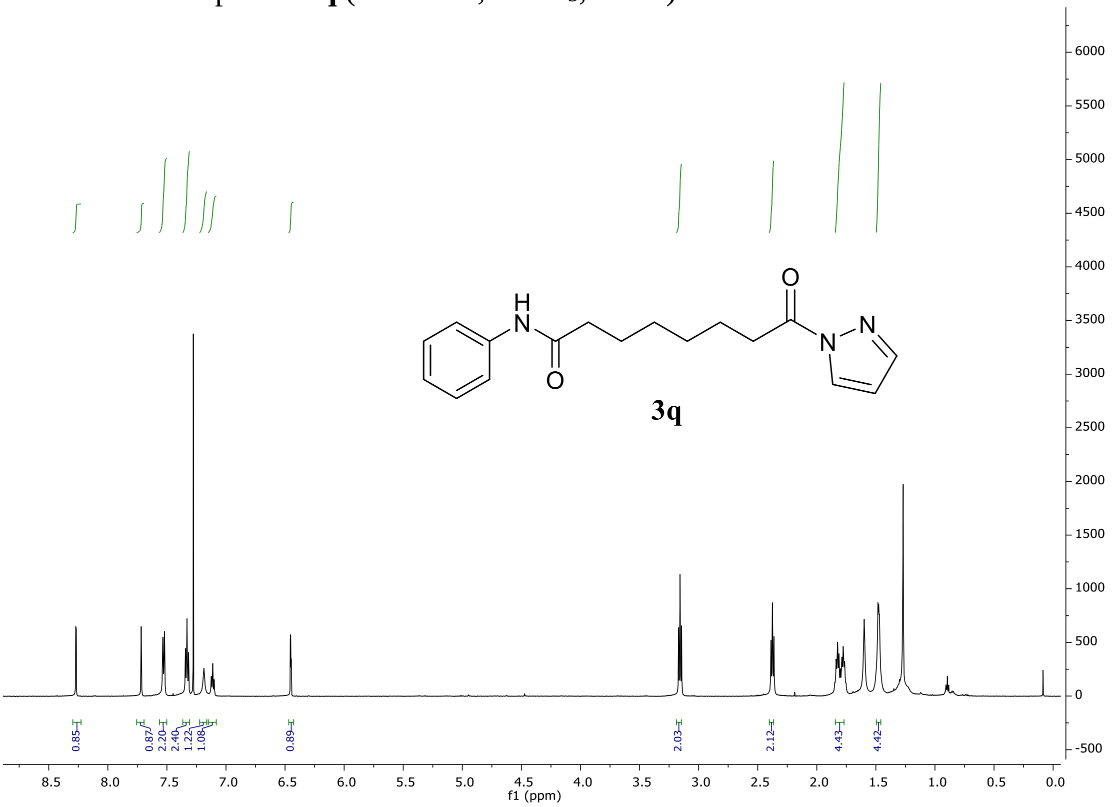

^{13}C NMR of compound **3q** (151 MHz, $CDCl_3$, 25 ºC)

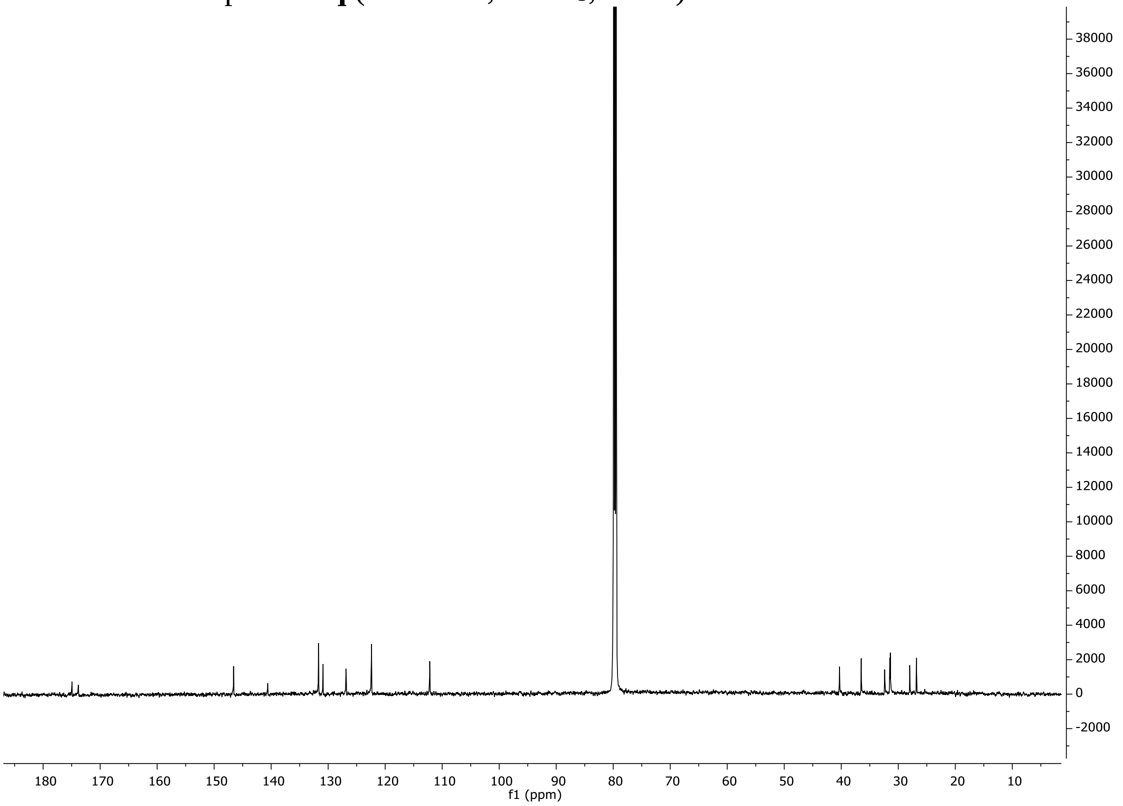

HSQC of compound **3q**

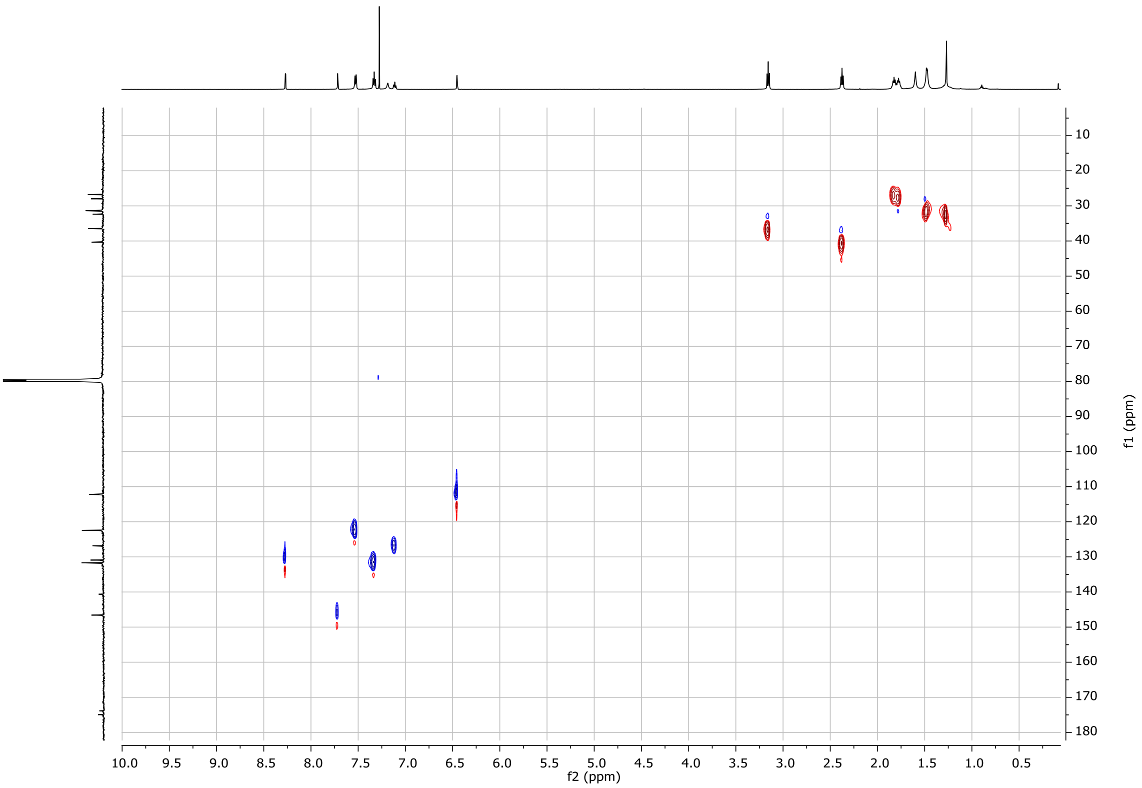

HMBC of compound **3q**

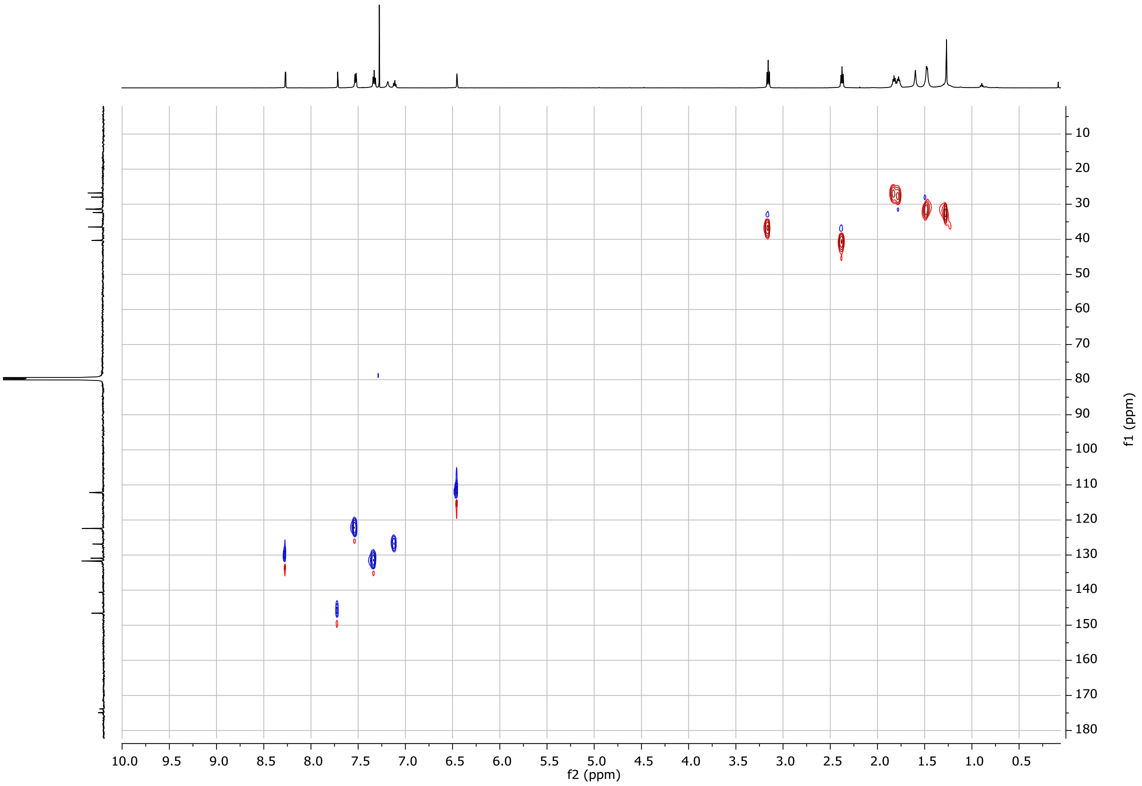

^{1}H NMR of compound **4** (600 MHz, $CDCl_3$, 25 ºC)

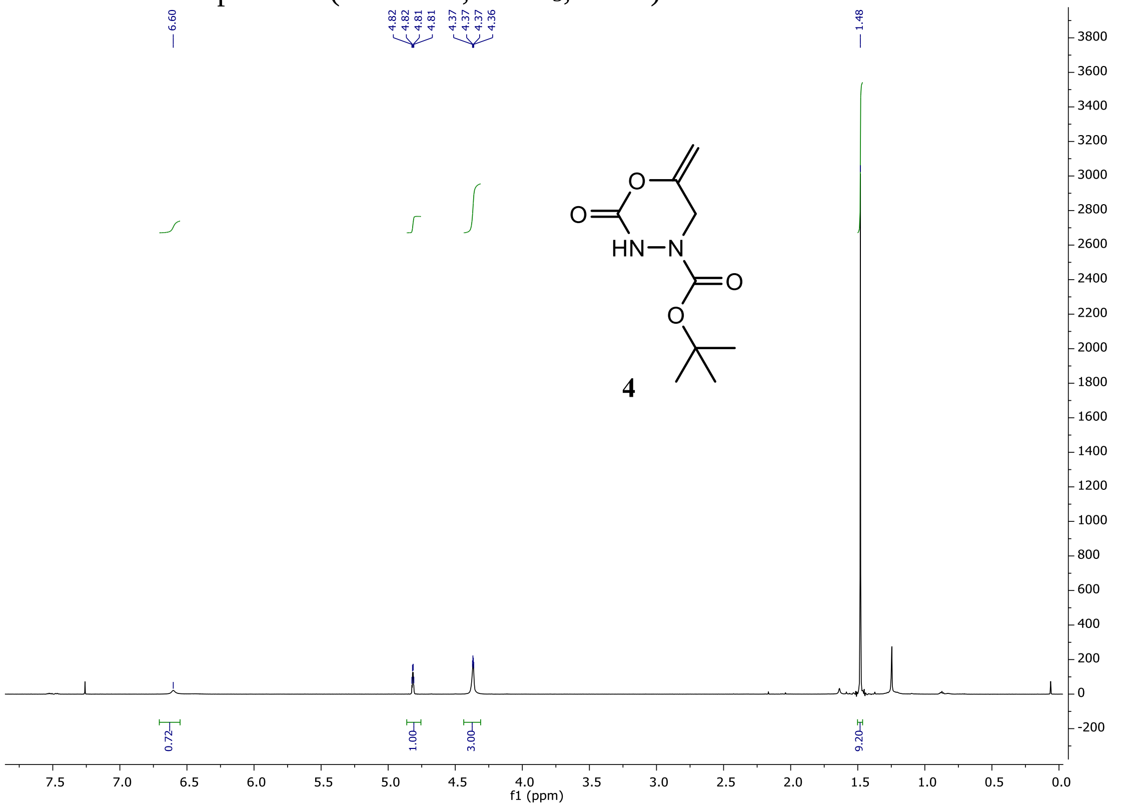

^{13}C NMR of compound **4** (151 MHz, $CDCl_3$, 25 ºC)

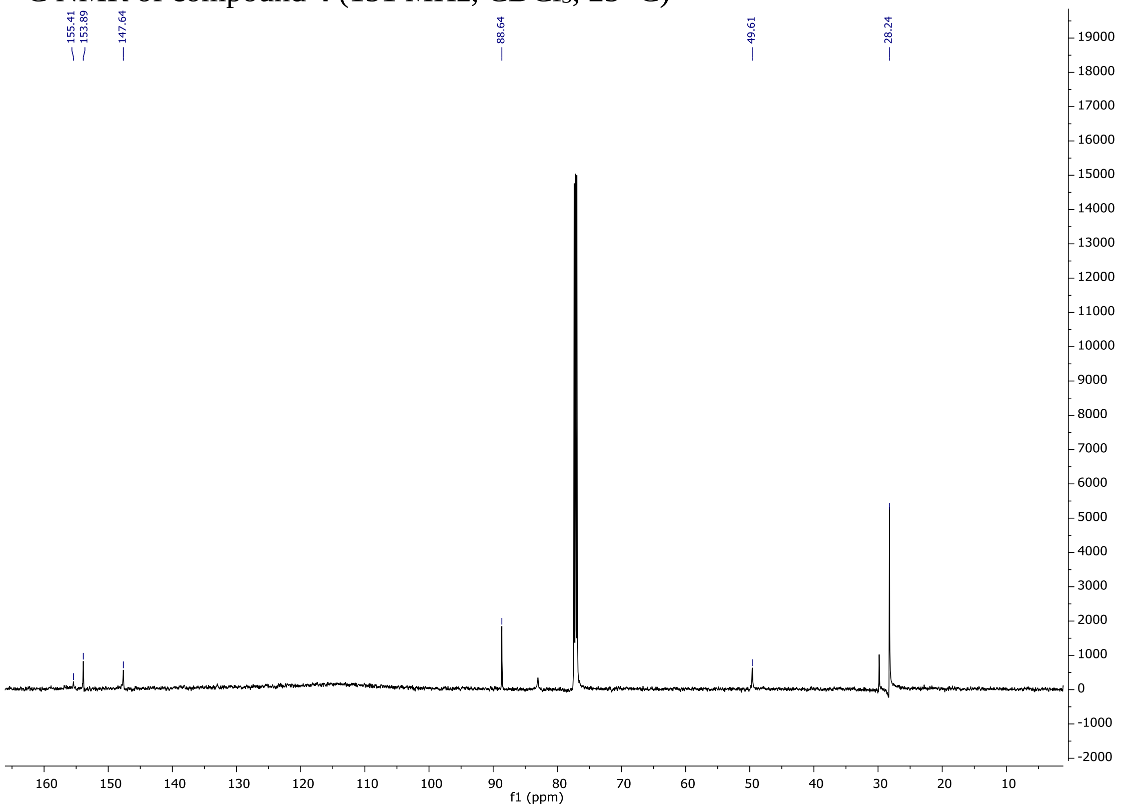

HSQC of compound **4**

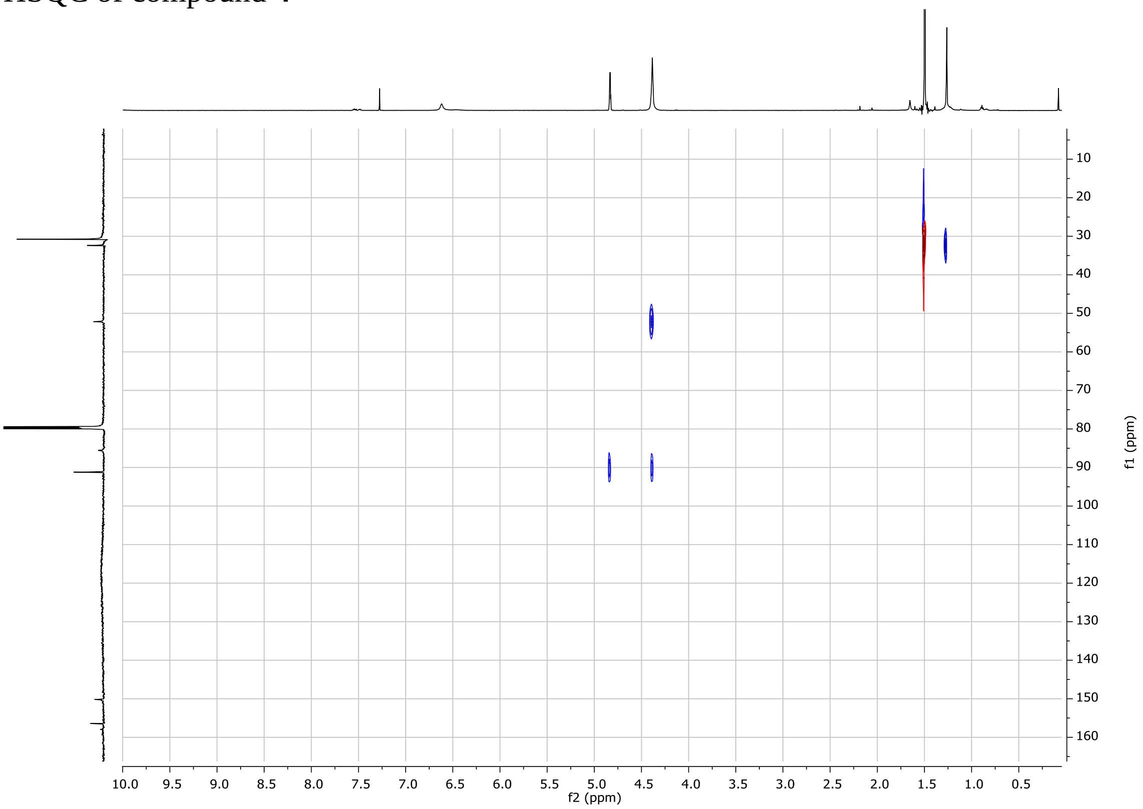

HMBC of compound **4**

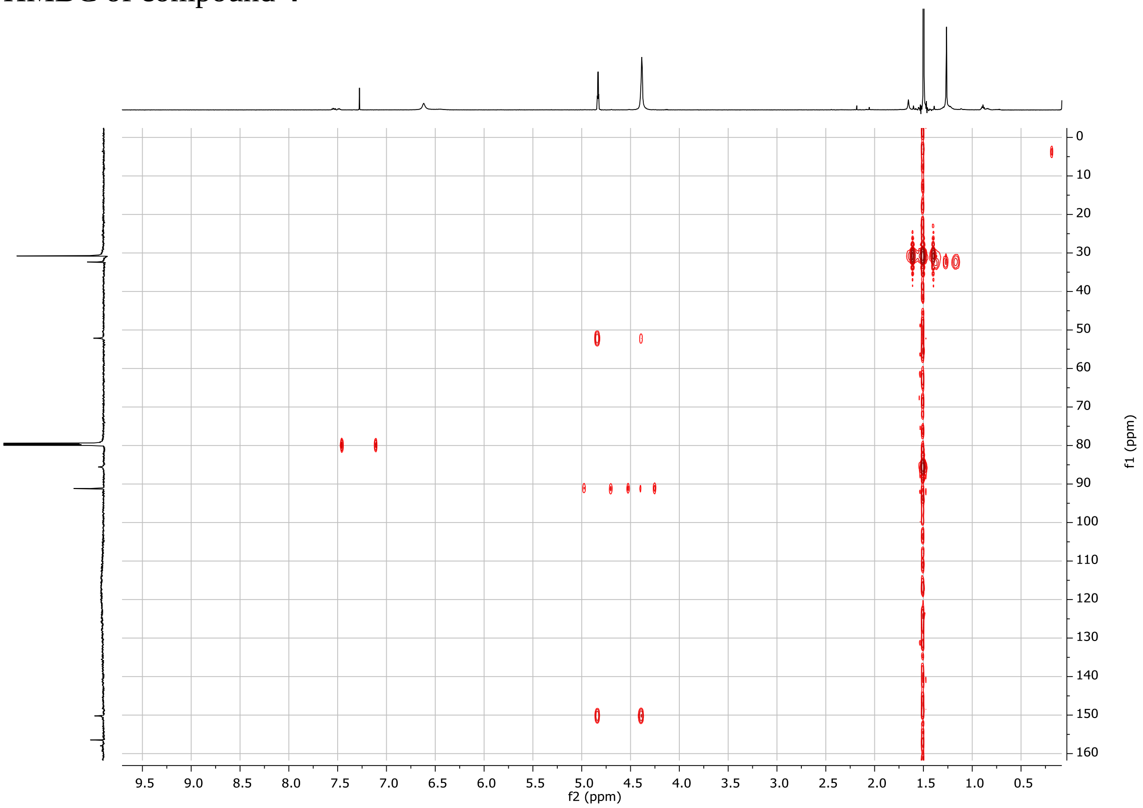

^{1}H NMR of compound **2g-BrBz** (600 MHz, $CDCl_3$, 25 ºC)

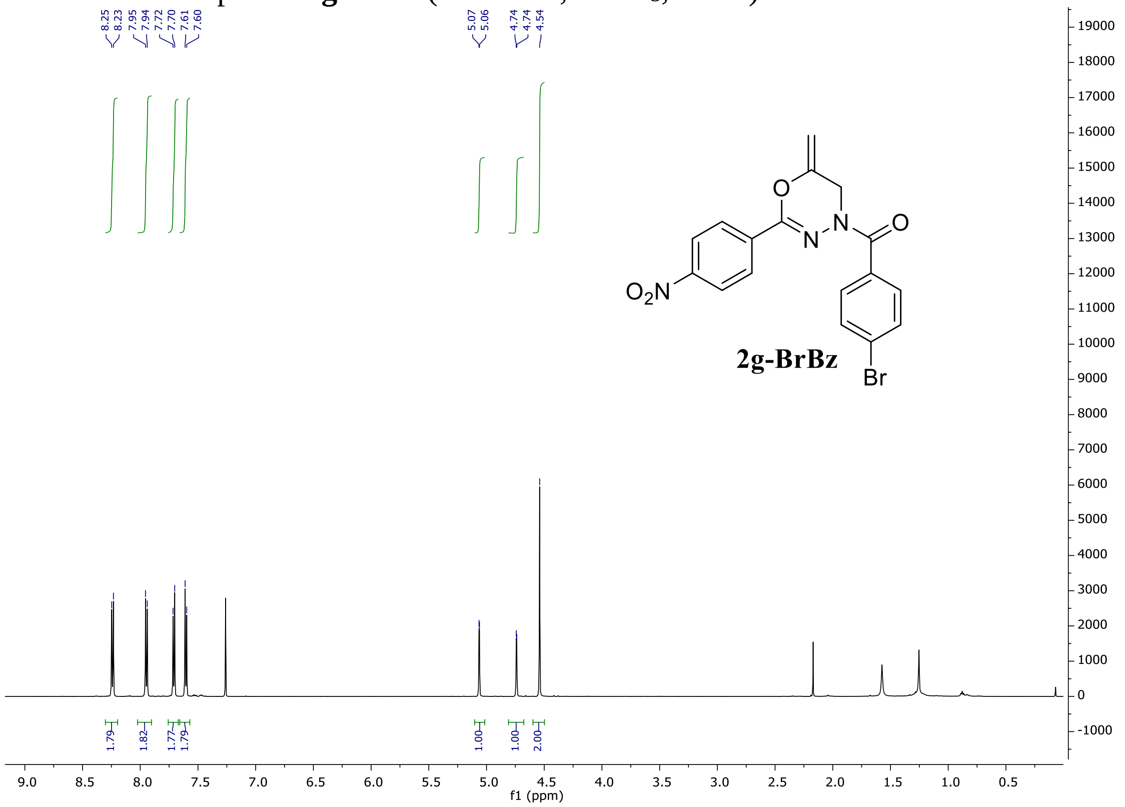

^{13}C NMR of compound **2g-BrBz** (151 MHz, $CDCl_3$, 25 ºC)

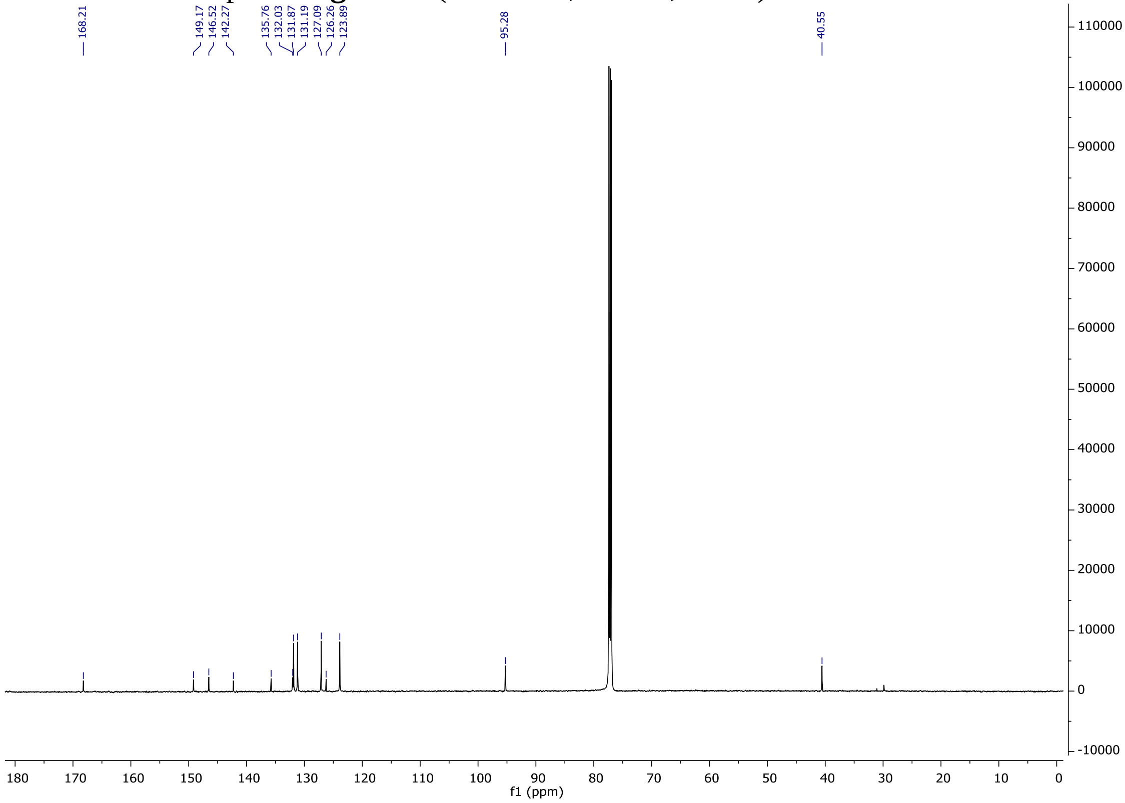

HSQC of compound **2g-BrBz**

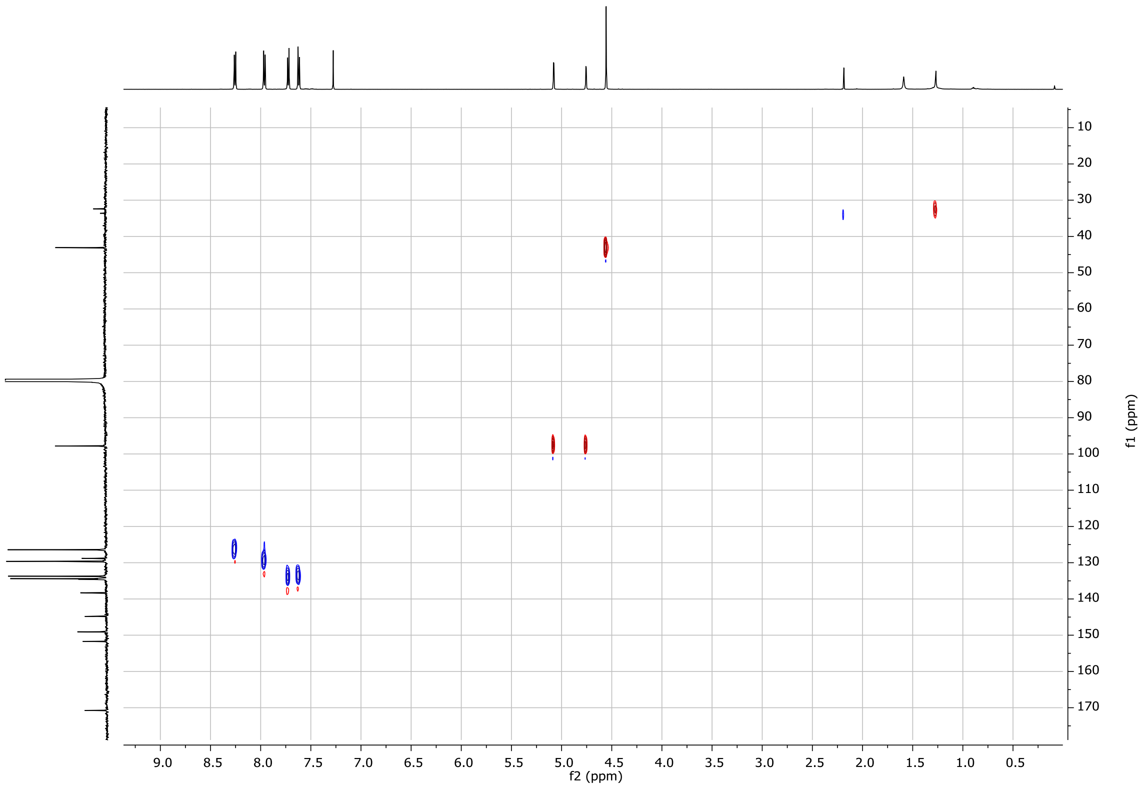

HMBC of compound **2g-BrBz**

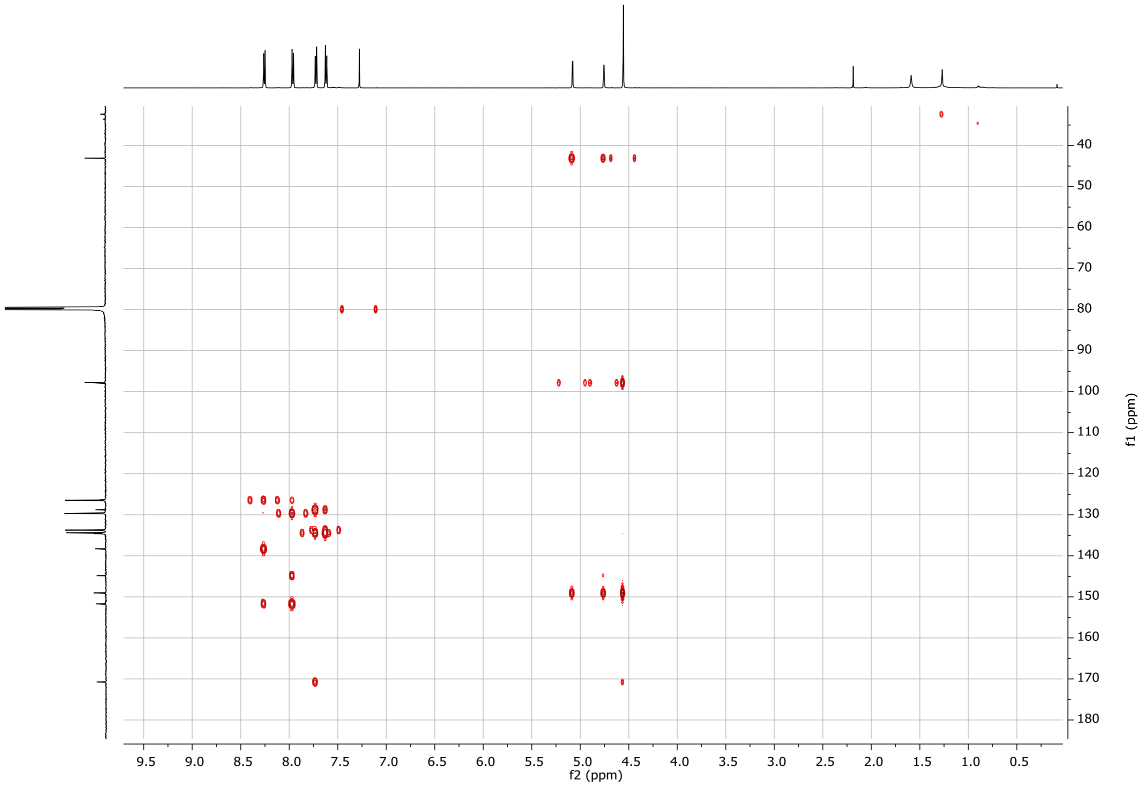

^{1}H NMR of compound **2g-Ac** (600 MHz, $CDCl_3$, 25 ºC)

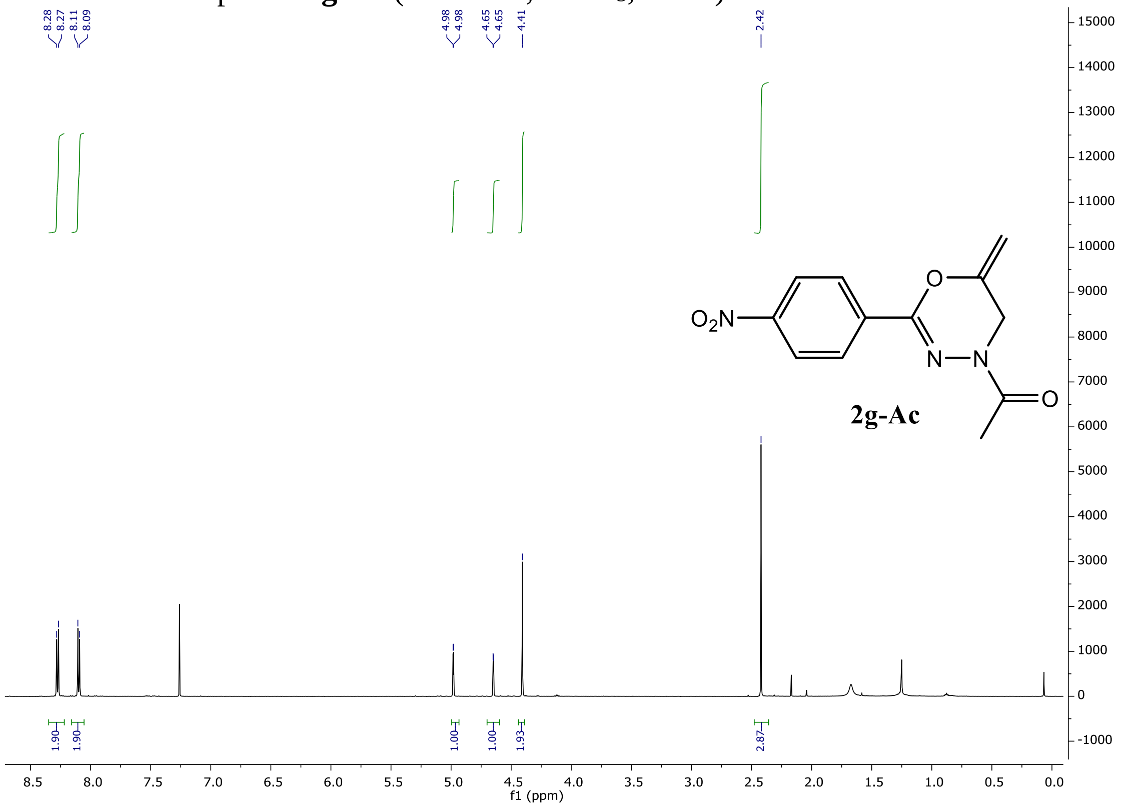

^{13}C NMR of compound **2g-Ac** (151 MHz, $CDCl_3$, 25 ºC)

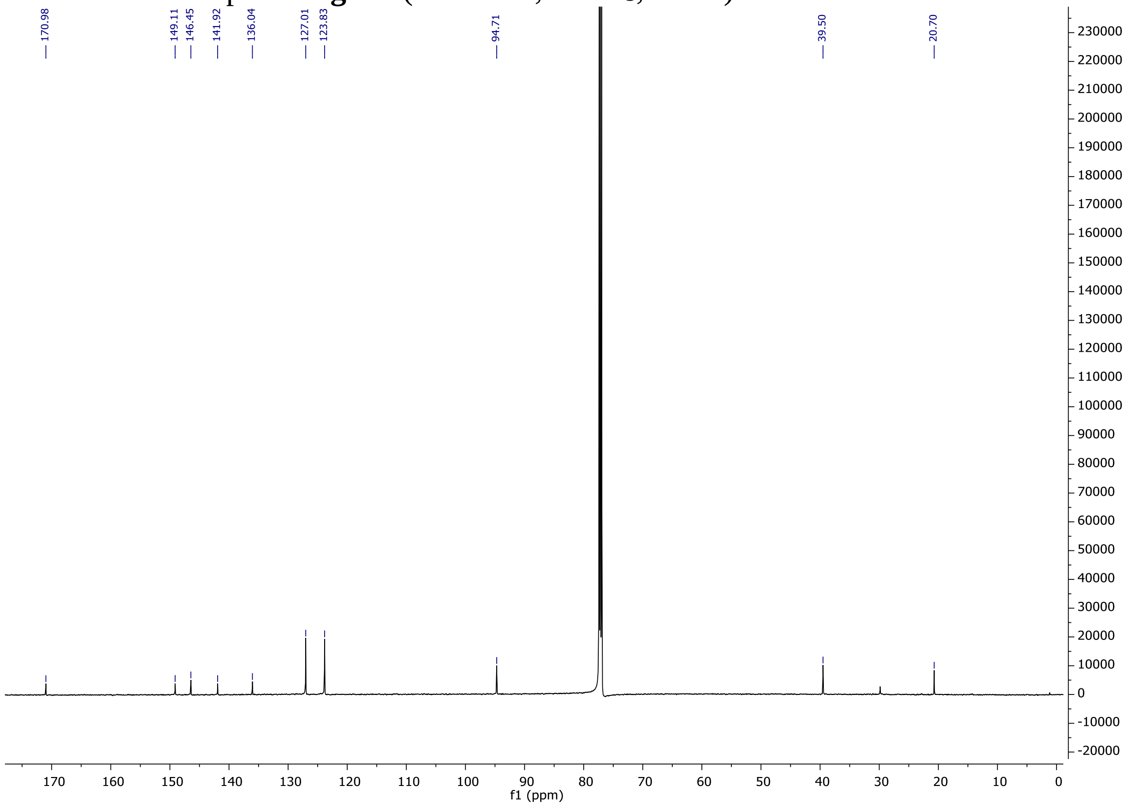

HSQC of compound **2g-Ac**

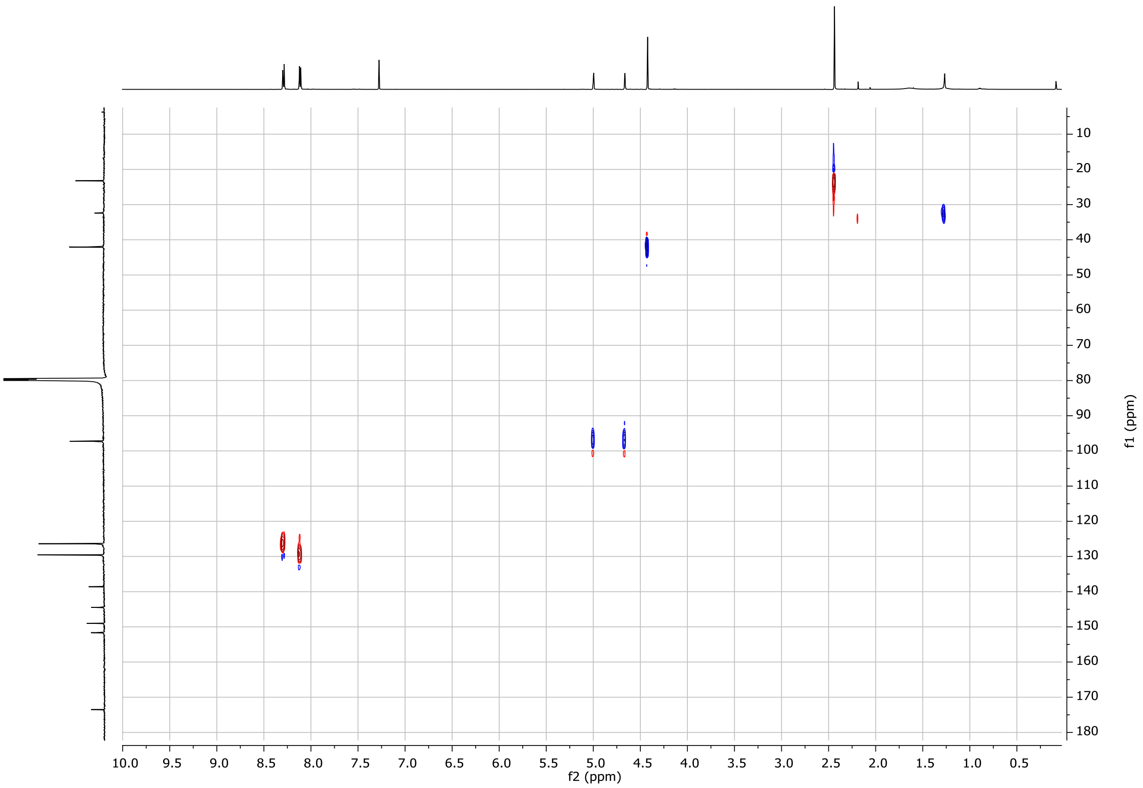

HMBC of compound **2g-Ac**

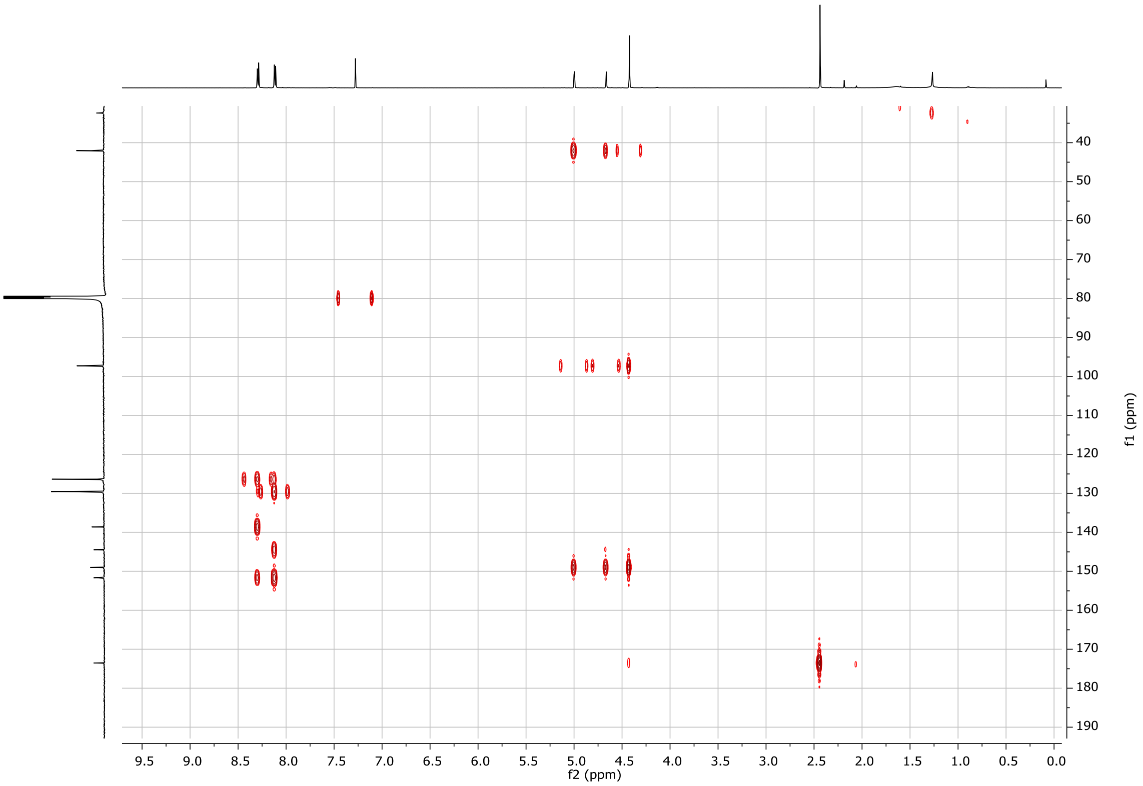

^{1}H NMR of compound **2o-Ac** (600 MHz, acetone d_6, 25 ºC)

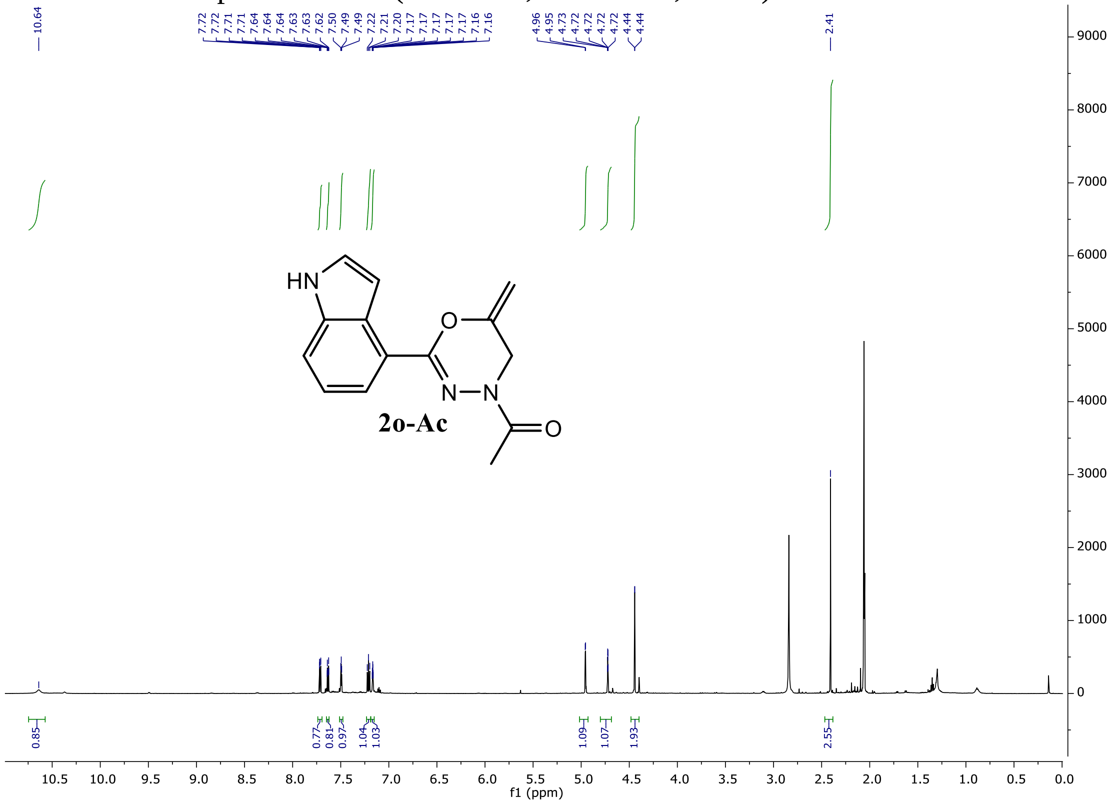

^{13}C NMR of compound **2o-Ac** (151 MHz, acetone d_6, 25 ºC)

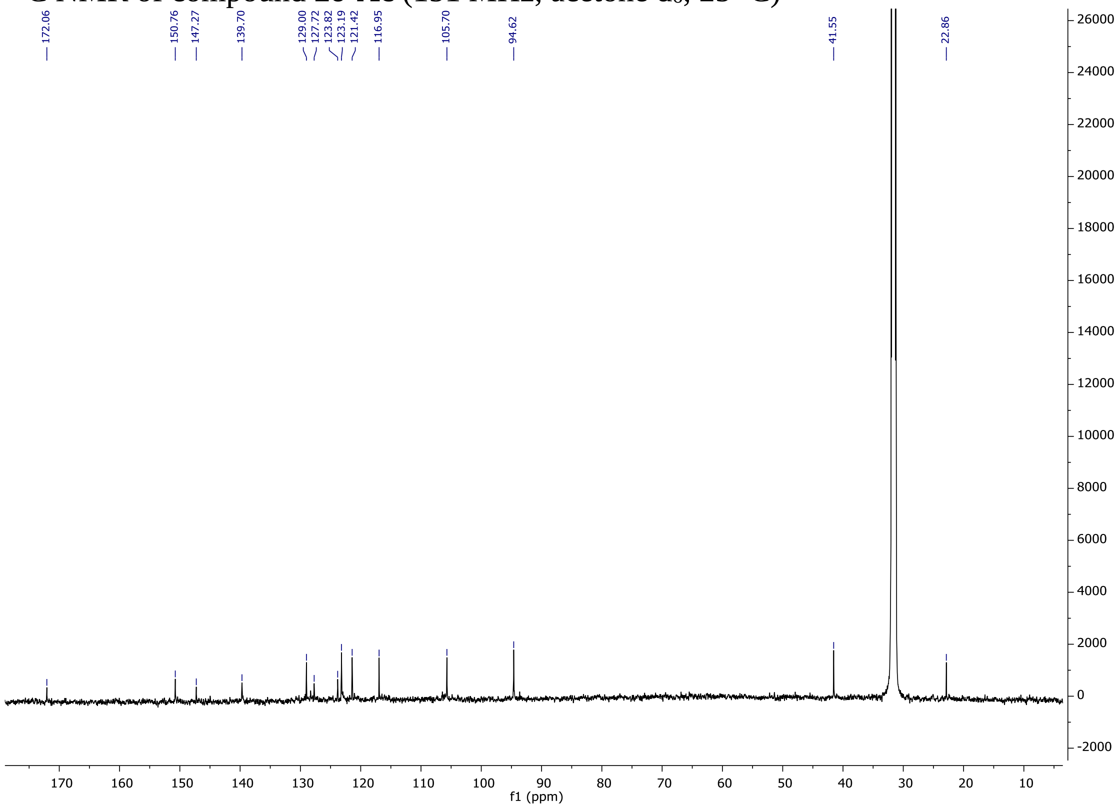

HSQC of compound **2o-Ac**

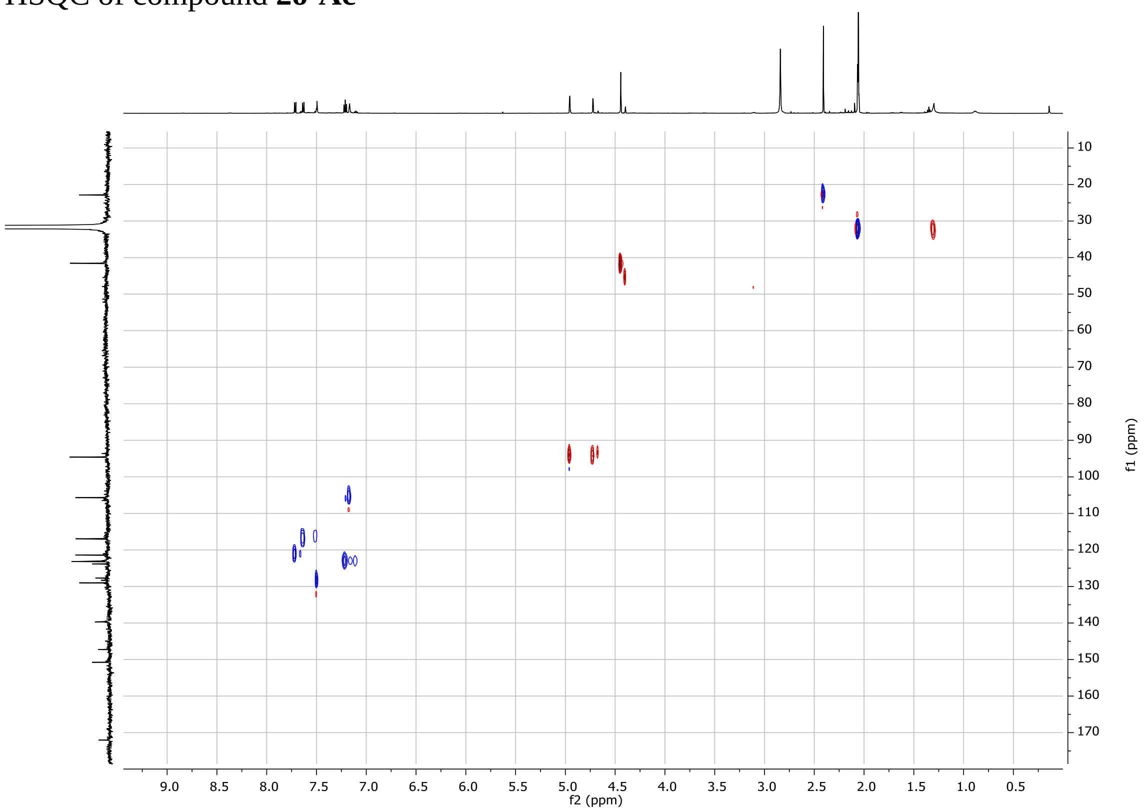

HMBC of compound **2o-Ac**

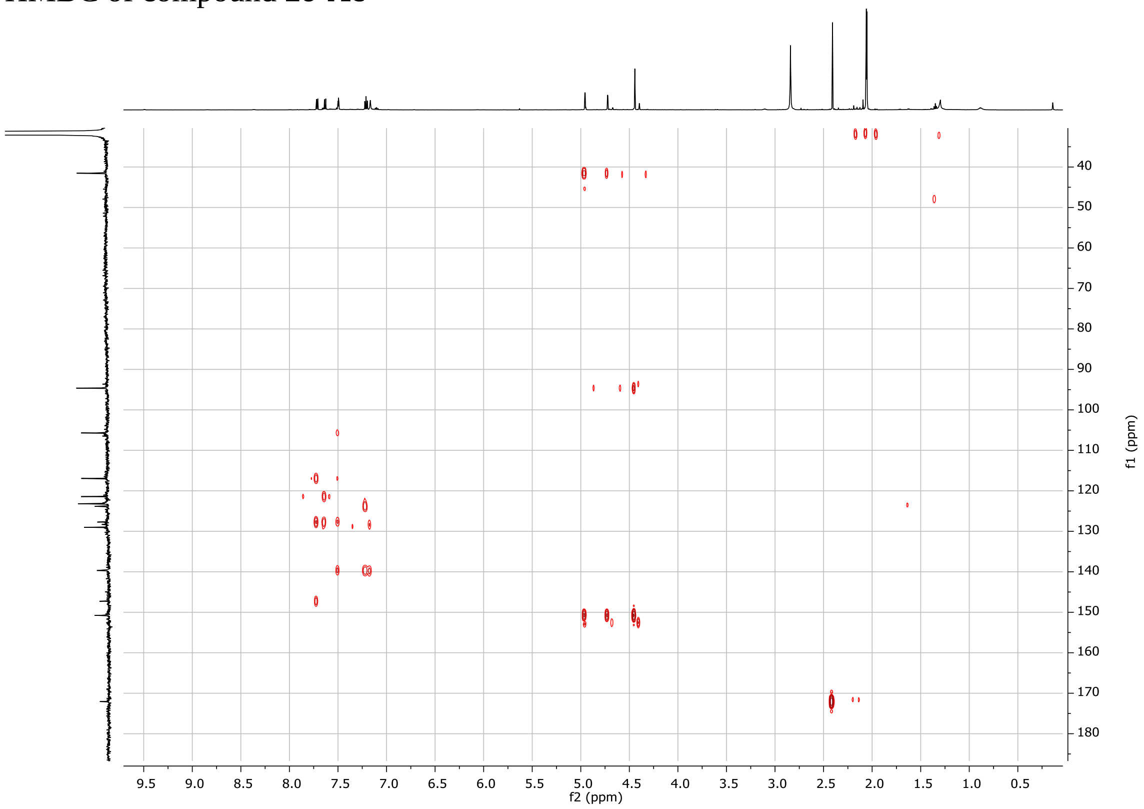

^{1}H NMR of compound **2p-Ac** (600 MHz, $CDCl_3$, 25 ºC)

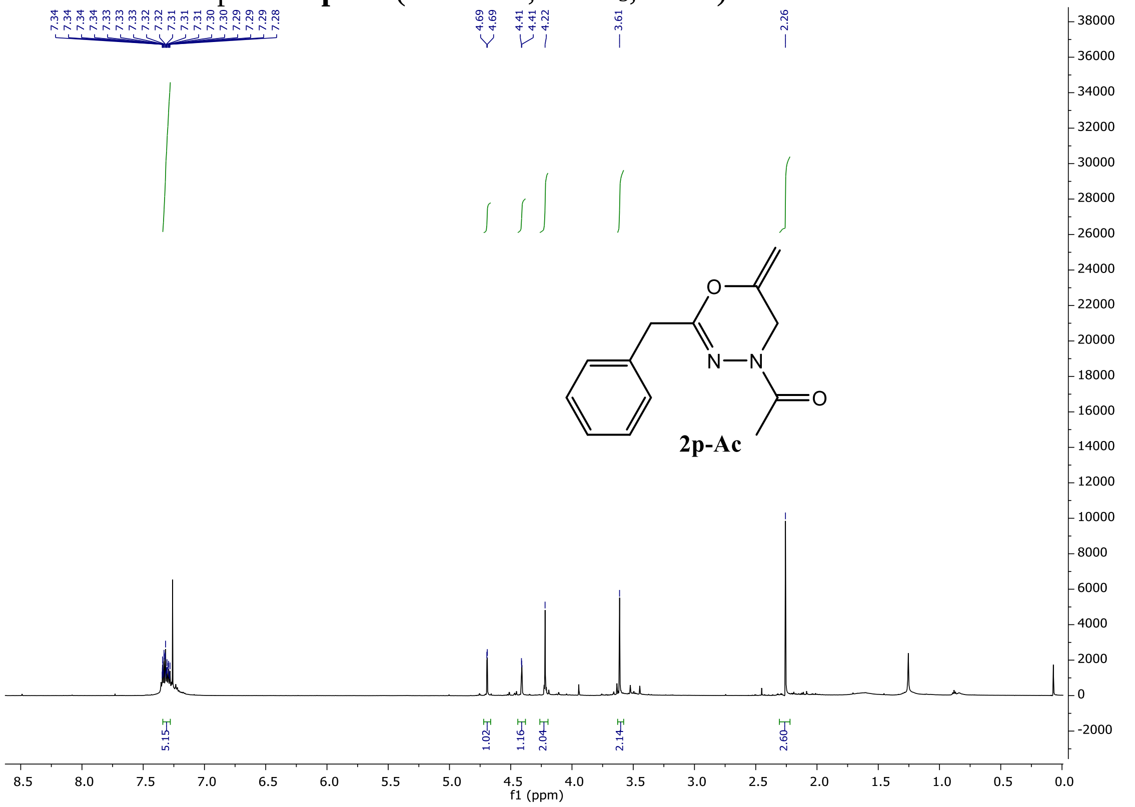

^{1}H NMR of compound **2p** (600 MHz, $CDCl_3$, 25 ºC) showing decomposition without acetylation

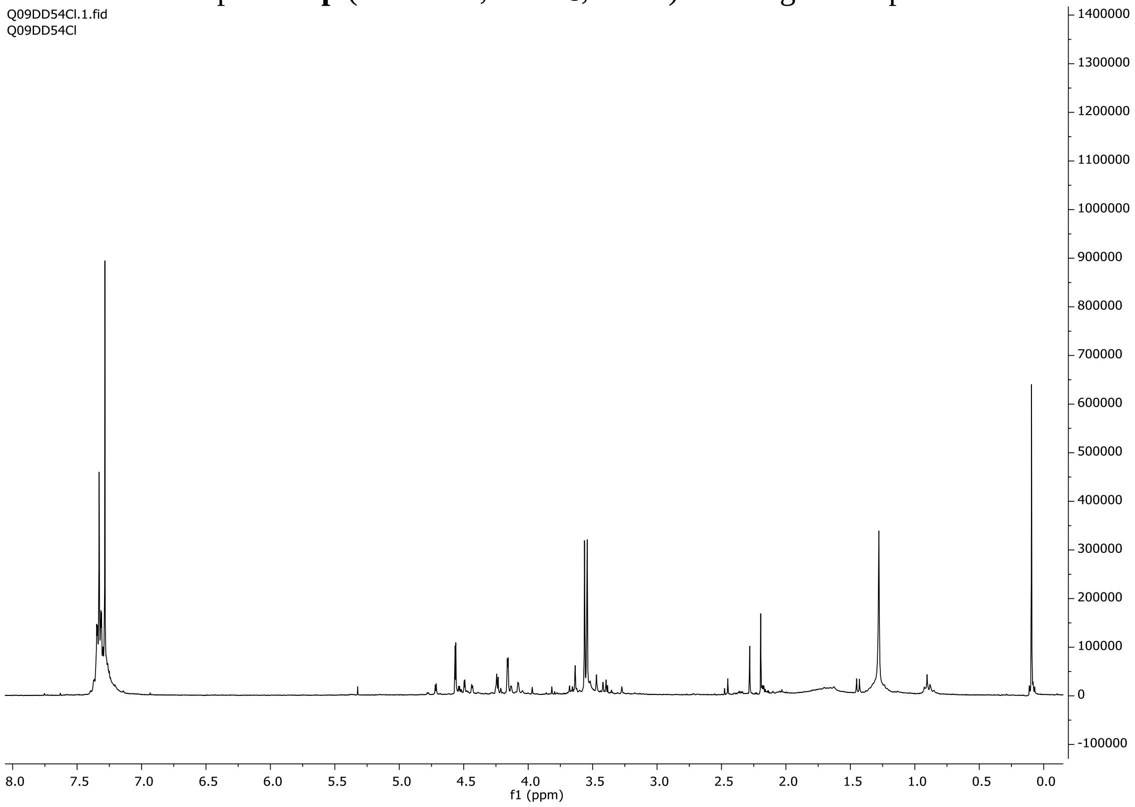

^{13}C NMR of compound **2p-Ac** (151 MHz, $CDCl_3$, 25 ºC)

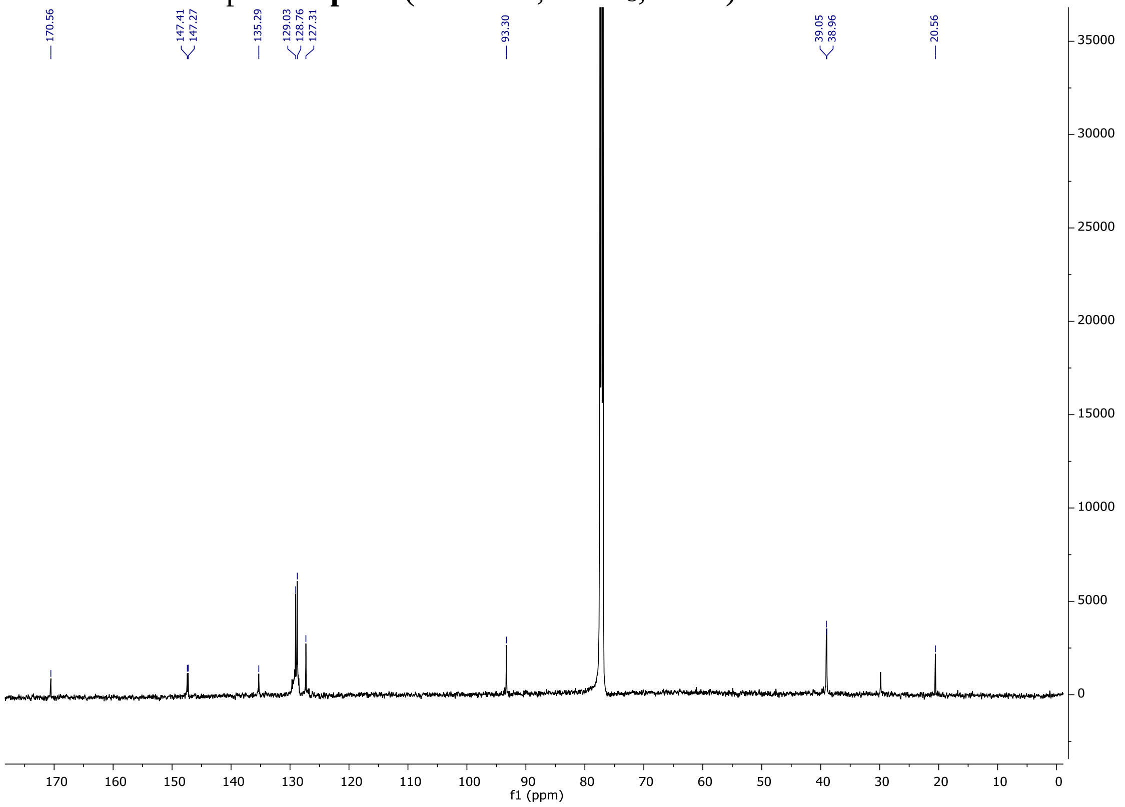

HSQC of compound **2p-Ac**

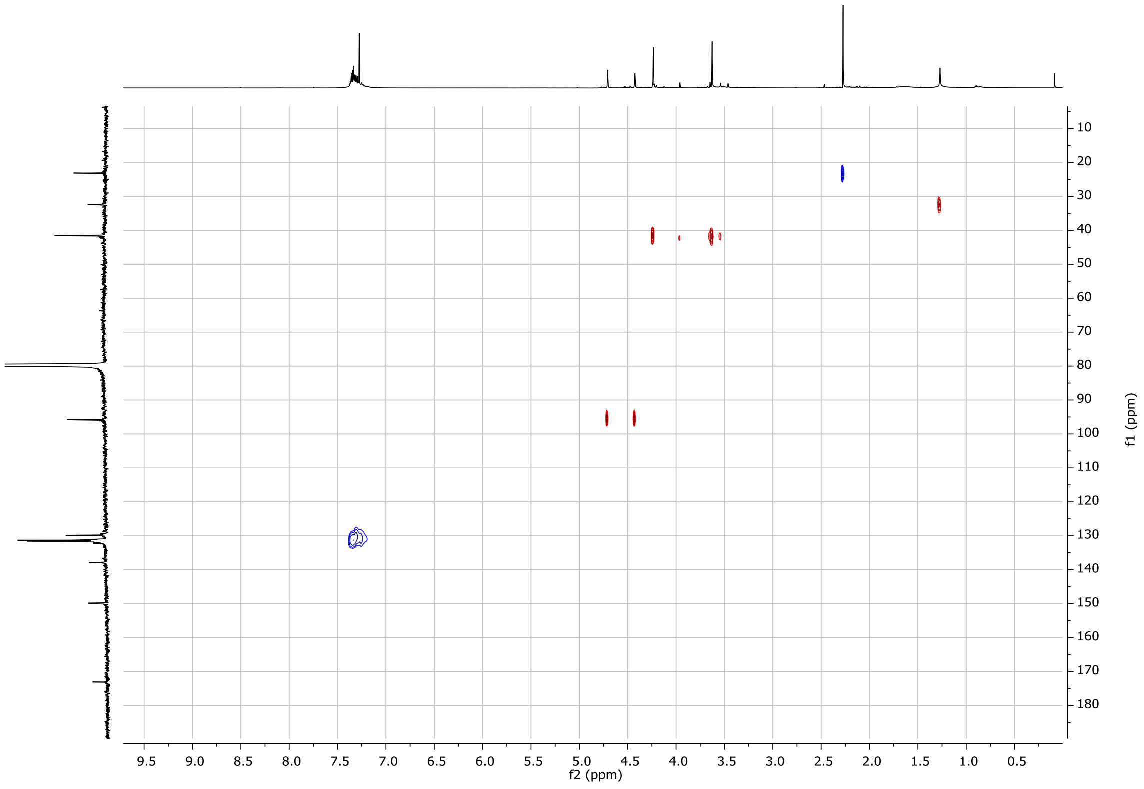

HMBC of compound **2p-Ac**

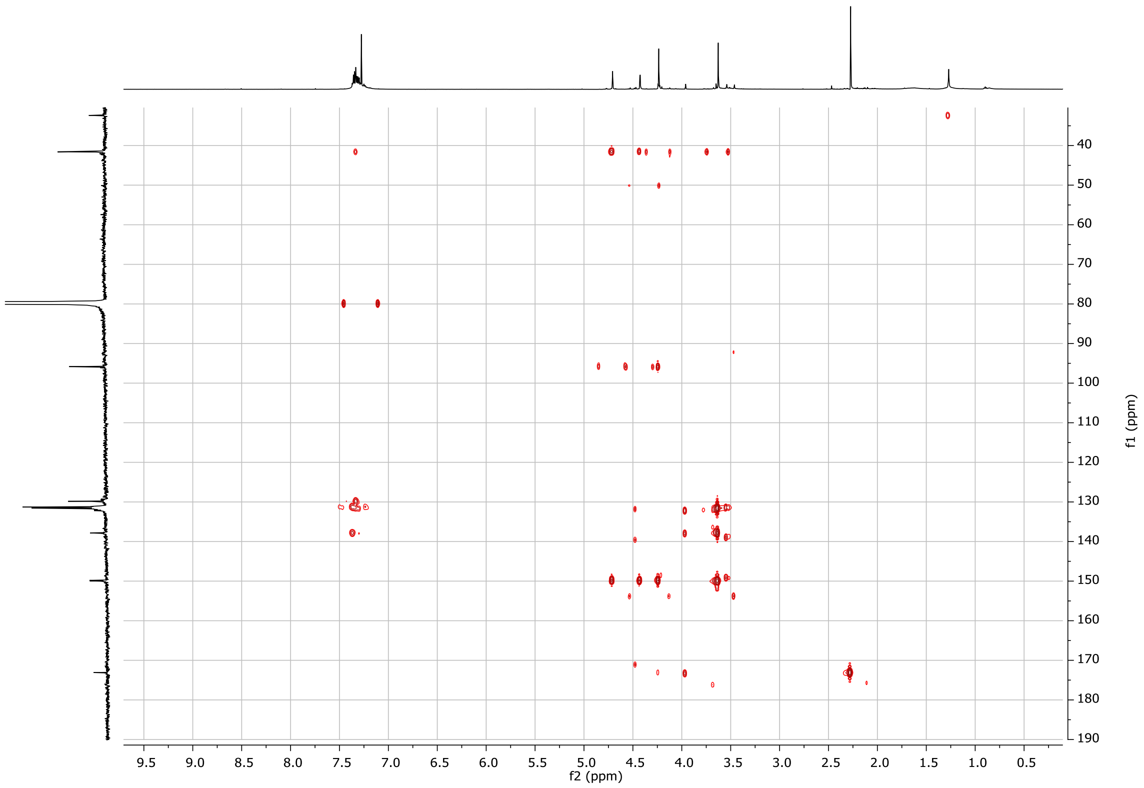

^{1}H NMR of compound **2q-Ac** (600 MHz, $CDCl_3$, 25 ºC)

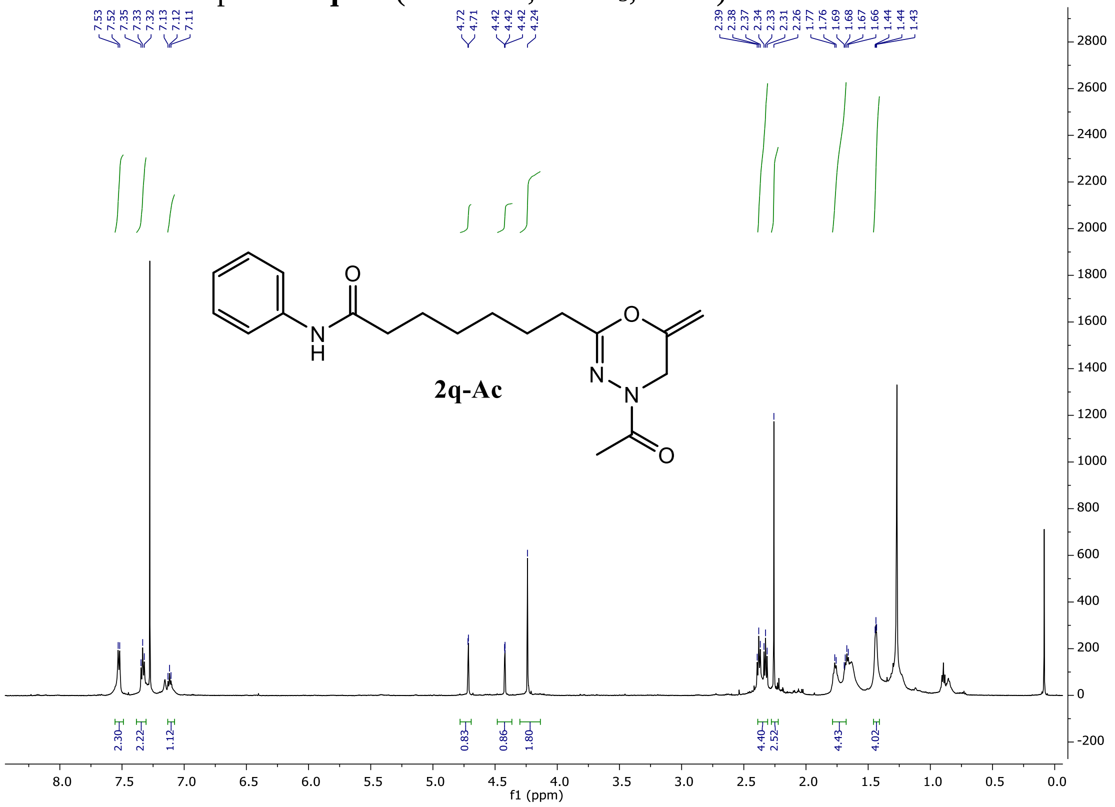

^{1}H NMR of compound **2q** (600 MHz, $CDCl_3$, 25 ºC) showing decomposition without acetylation

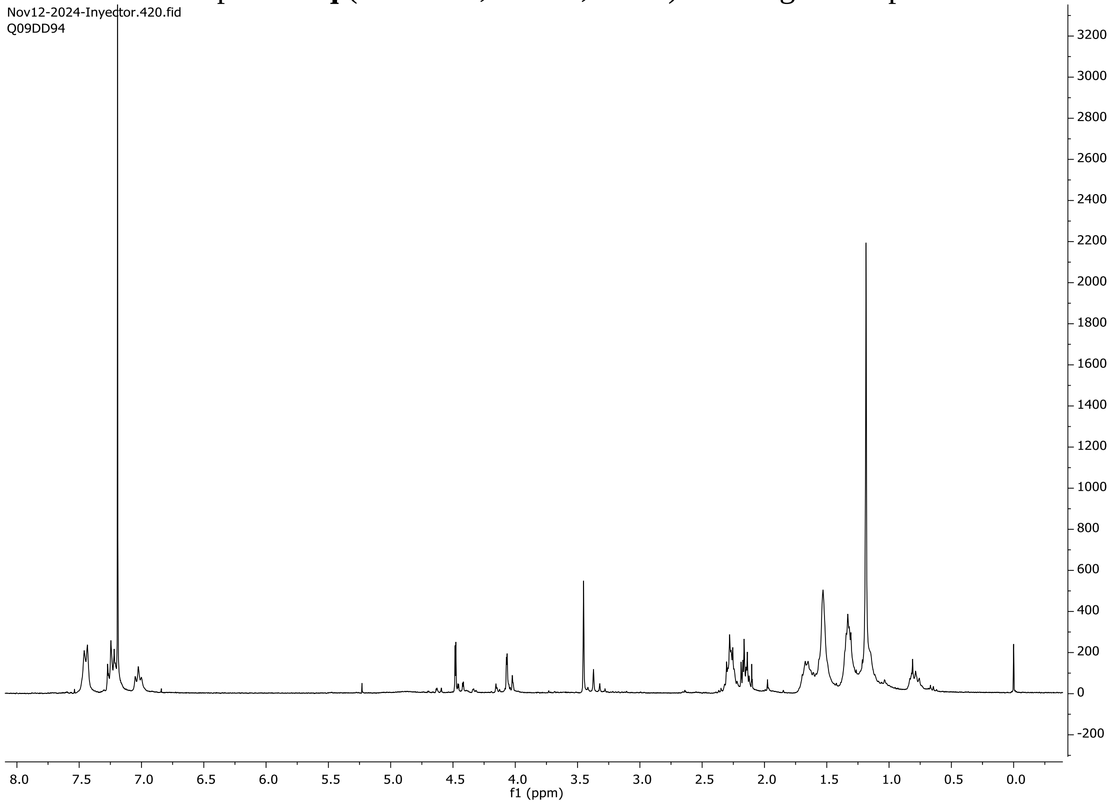

^{13}C NMR of compound **2q-Ac** (151 MHz, $CDCl_3$, 25 ºC)

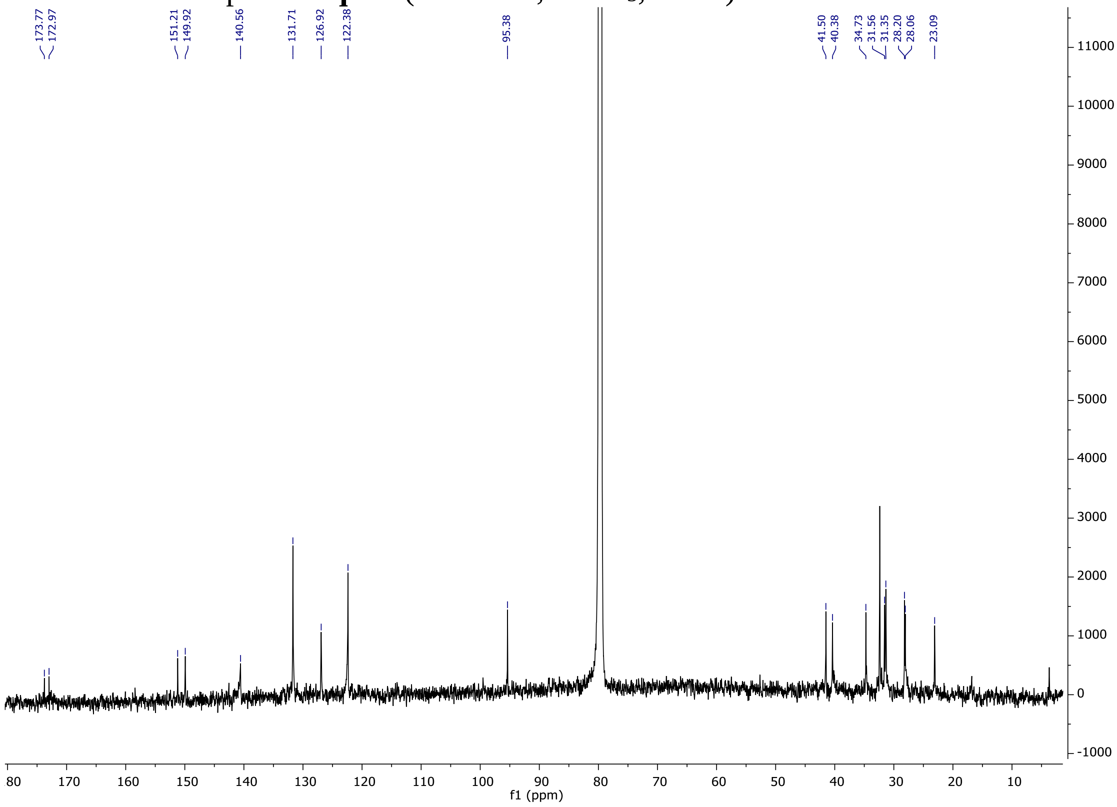

HSQC of compound **2q-Ac**

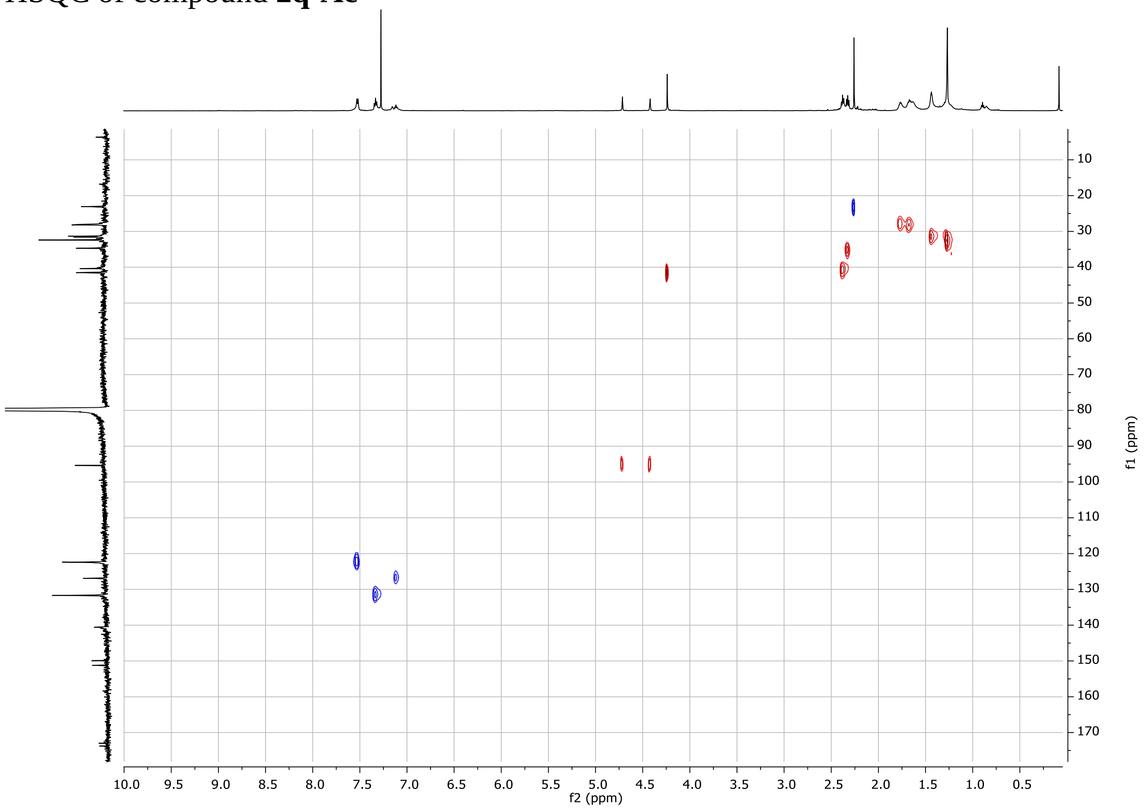

^{1}H NMR of compound **4g** (600 MHz, $CDCl_3$, 25 ºC)

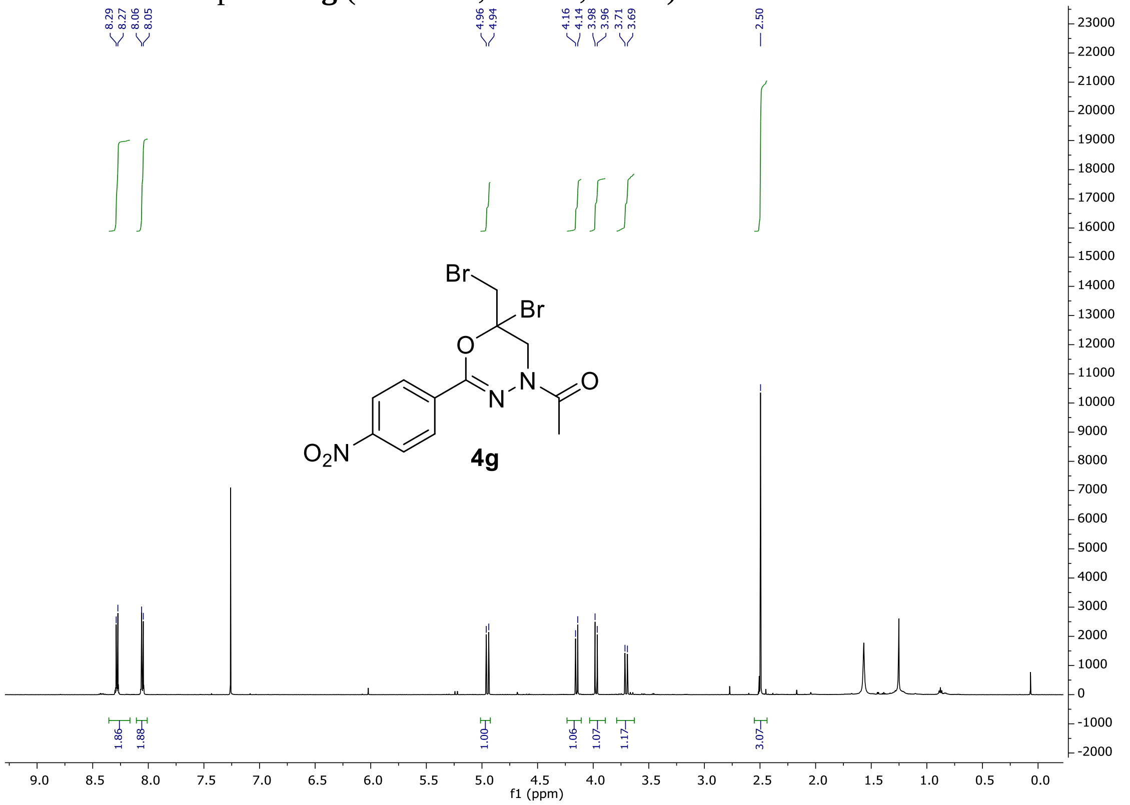

^{13}C NMR of compound **4g** (151 MHz, $CDCl_3$, 25 ºC)

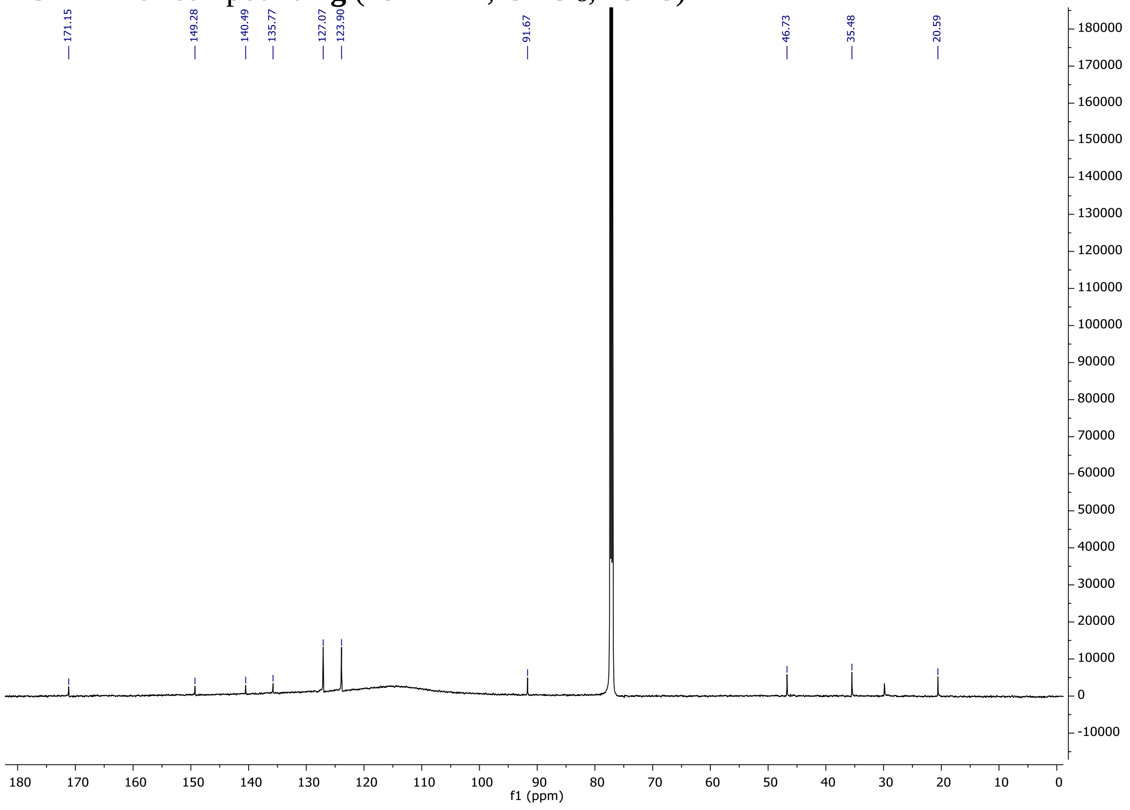

HSQC of compound **4g**

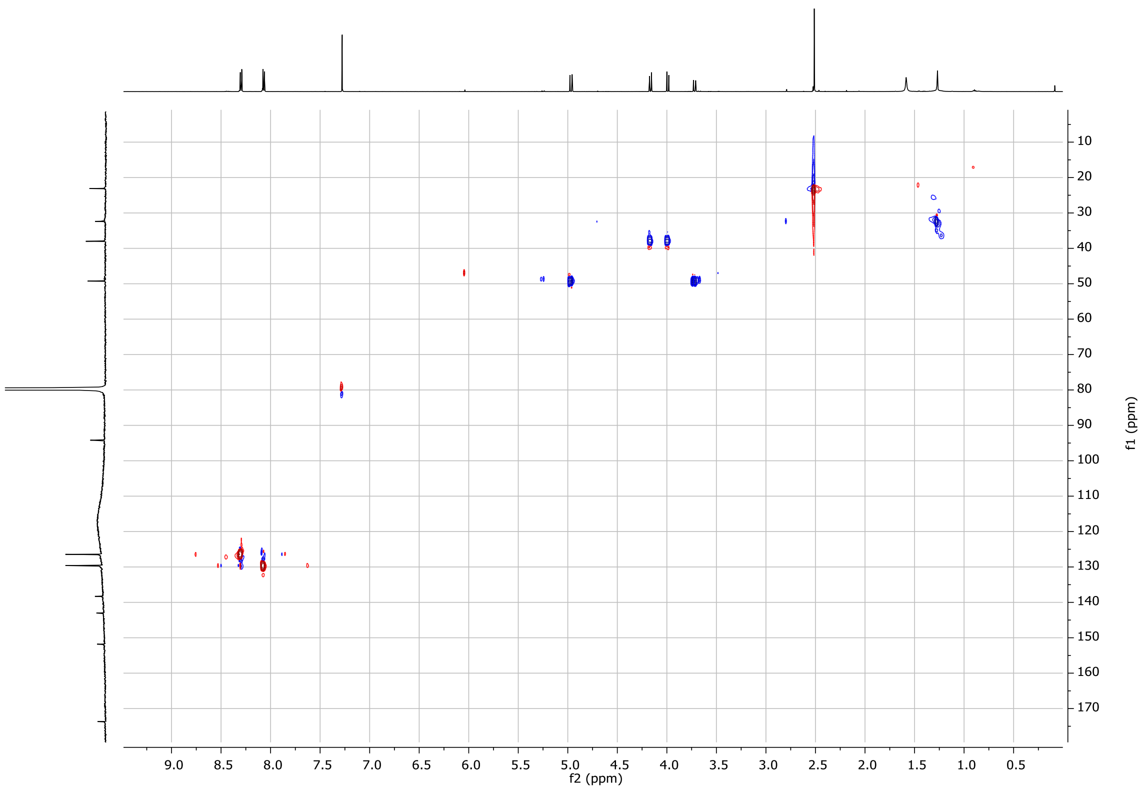

HMBC of compound **4g**

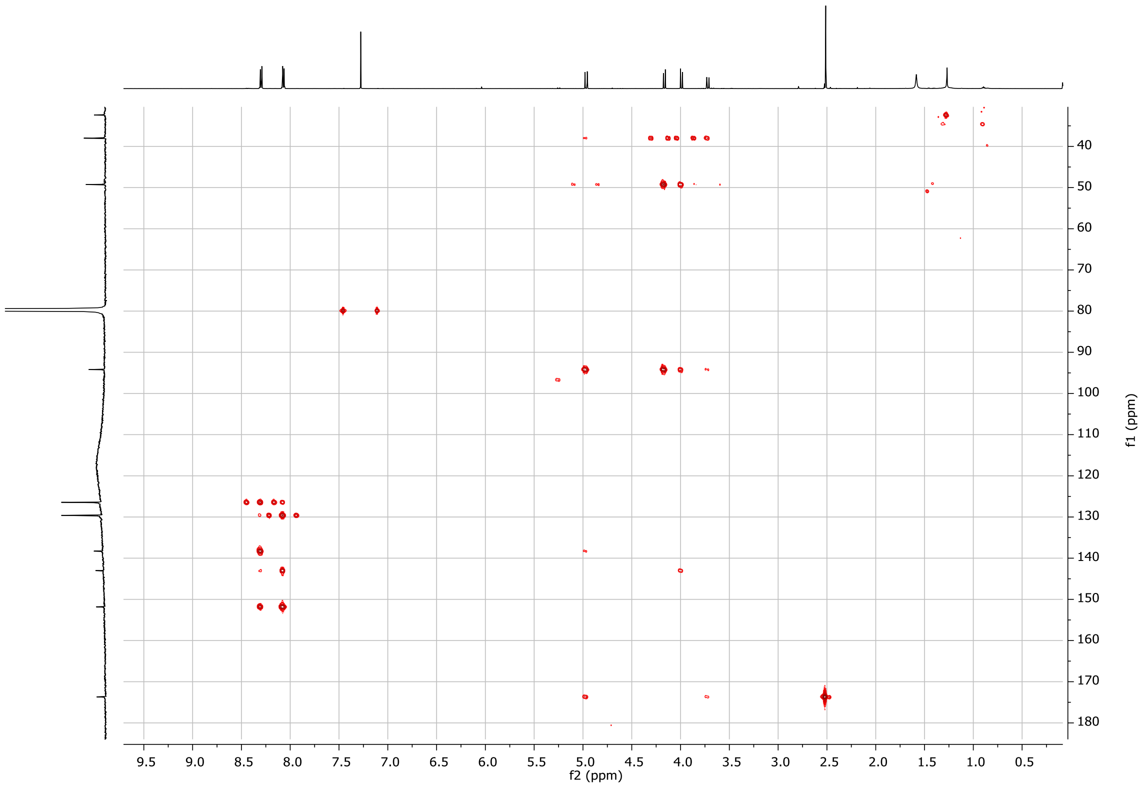

^{1}H NMR of compound **5g** (600 MHz, $CDCl_3$, 25 ºC)

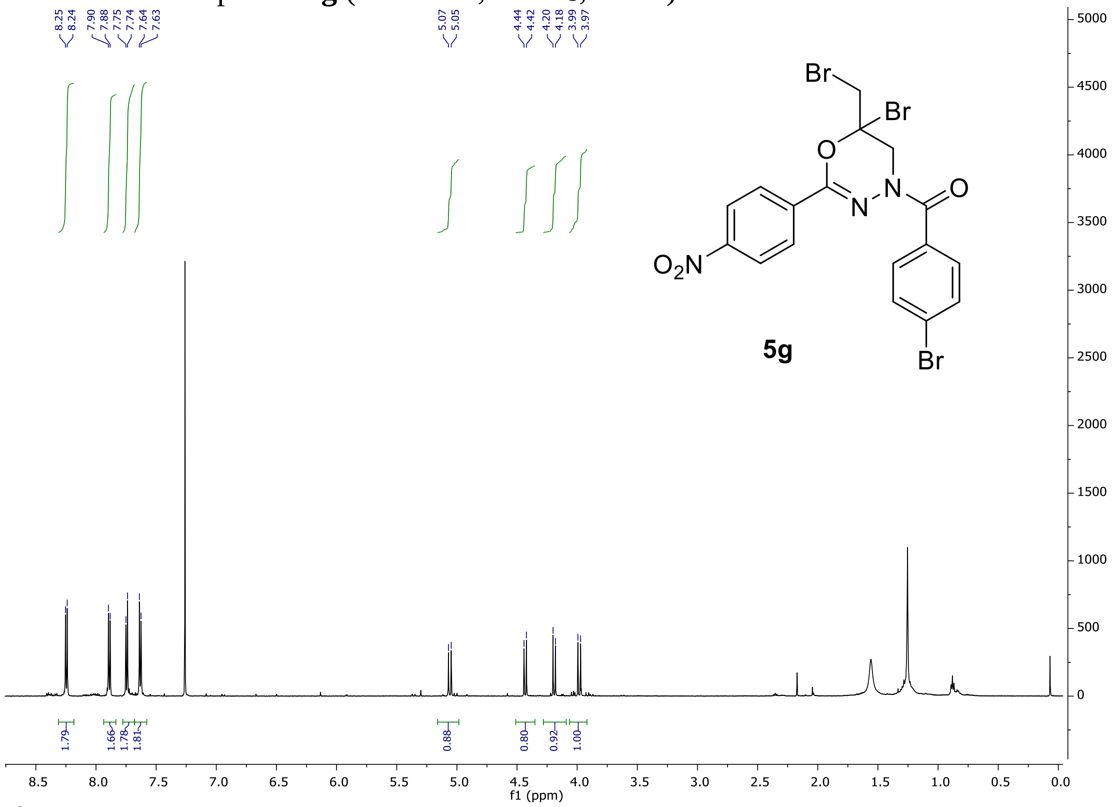

^{13}C NMR of compound **5g** (151 MHz, $CDCl_3$, 25 ºC)

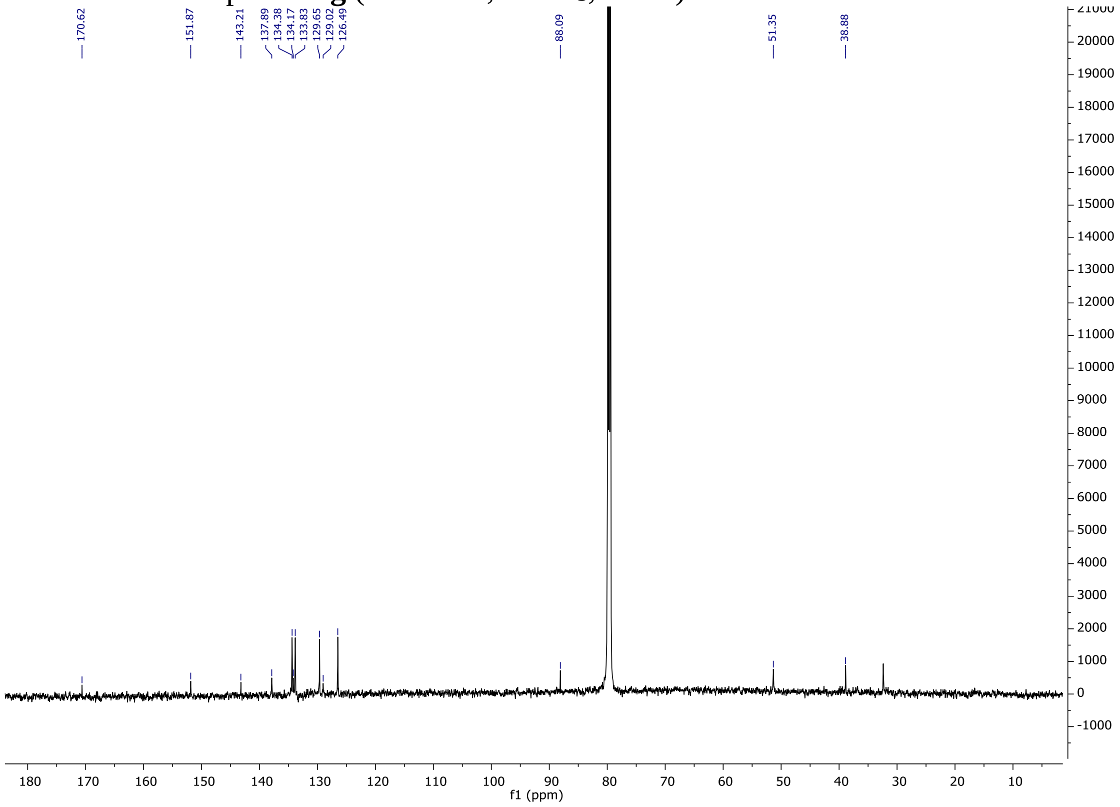

HSQC of compound **5g**

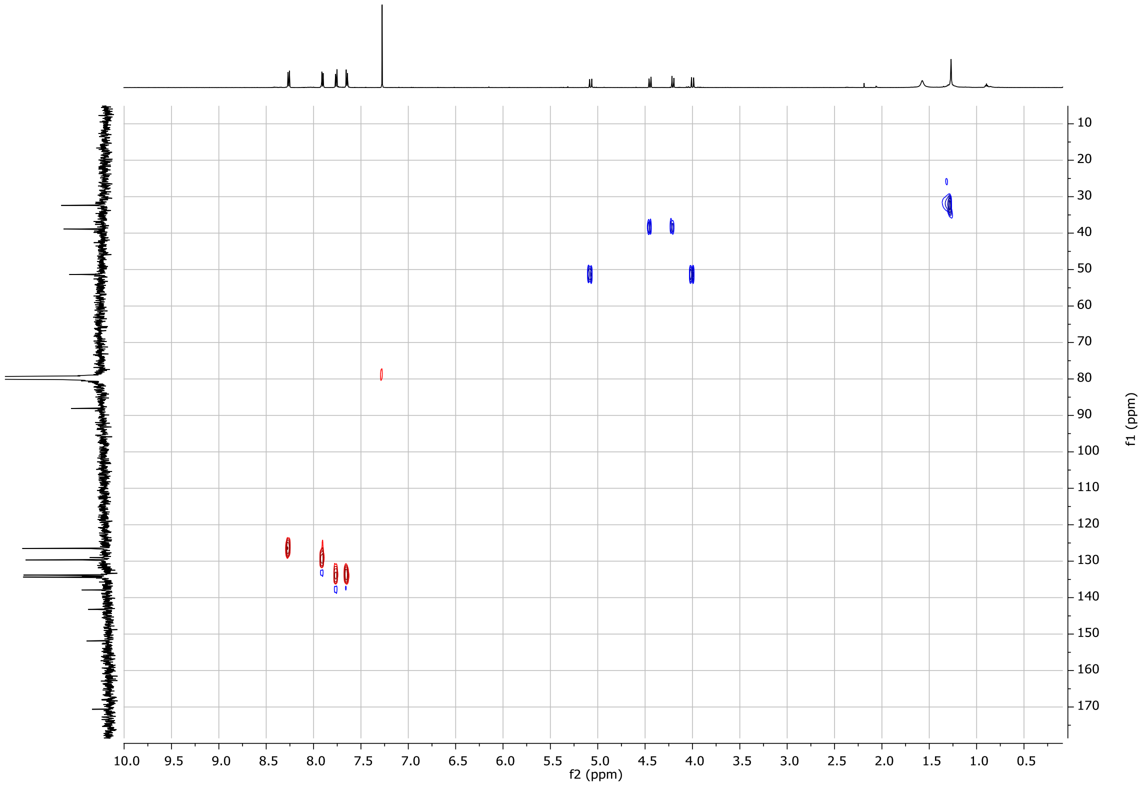

HMBC of compound **5g**

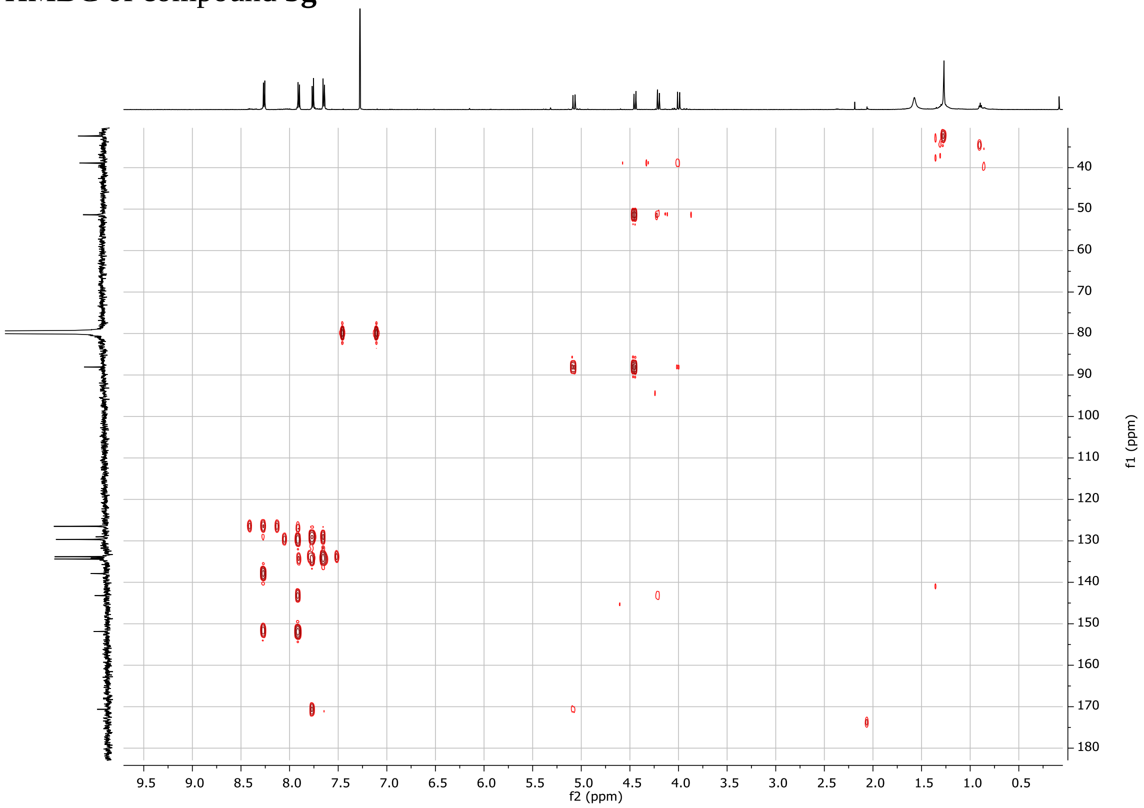

^{1}H NMR of compound **5g-rot** (600 MHz, $CDCl_3$, 25 ºC)

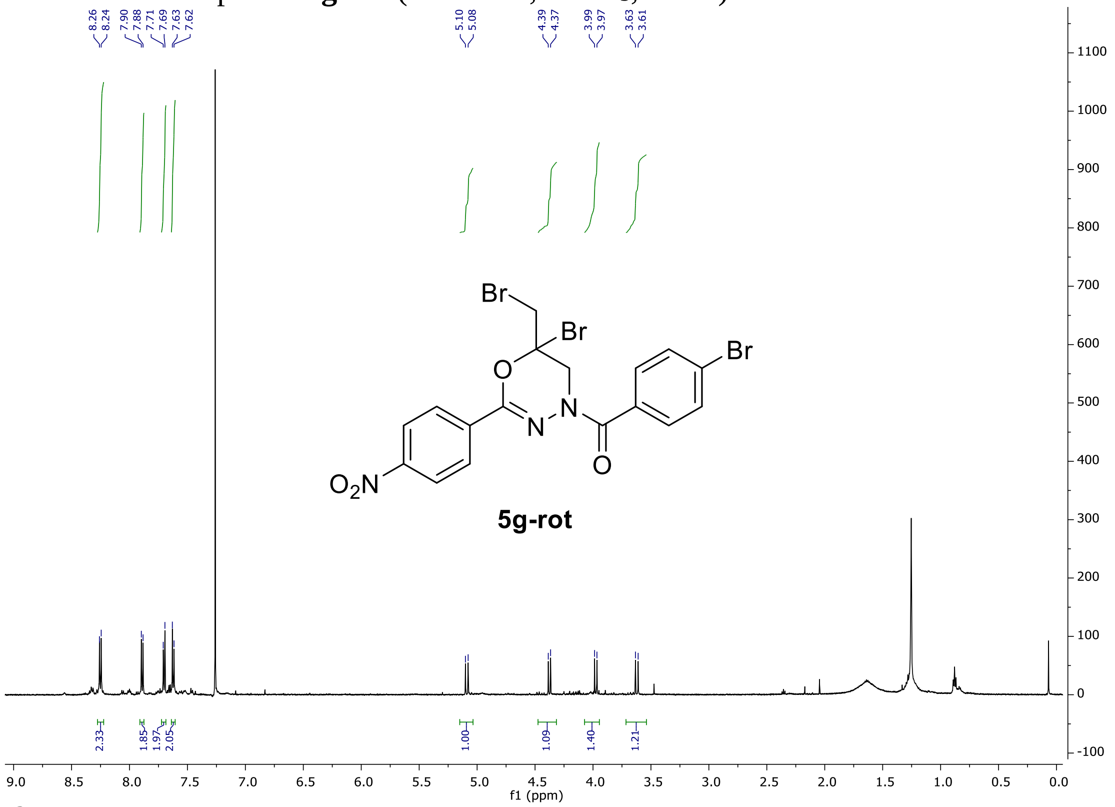

^{13}C NMR of compound **5g-rot** (151 MHz, $CDCl_3$, 25 ºC)

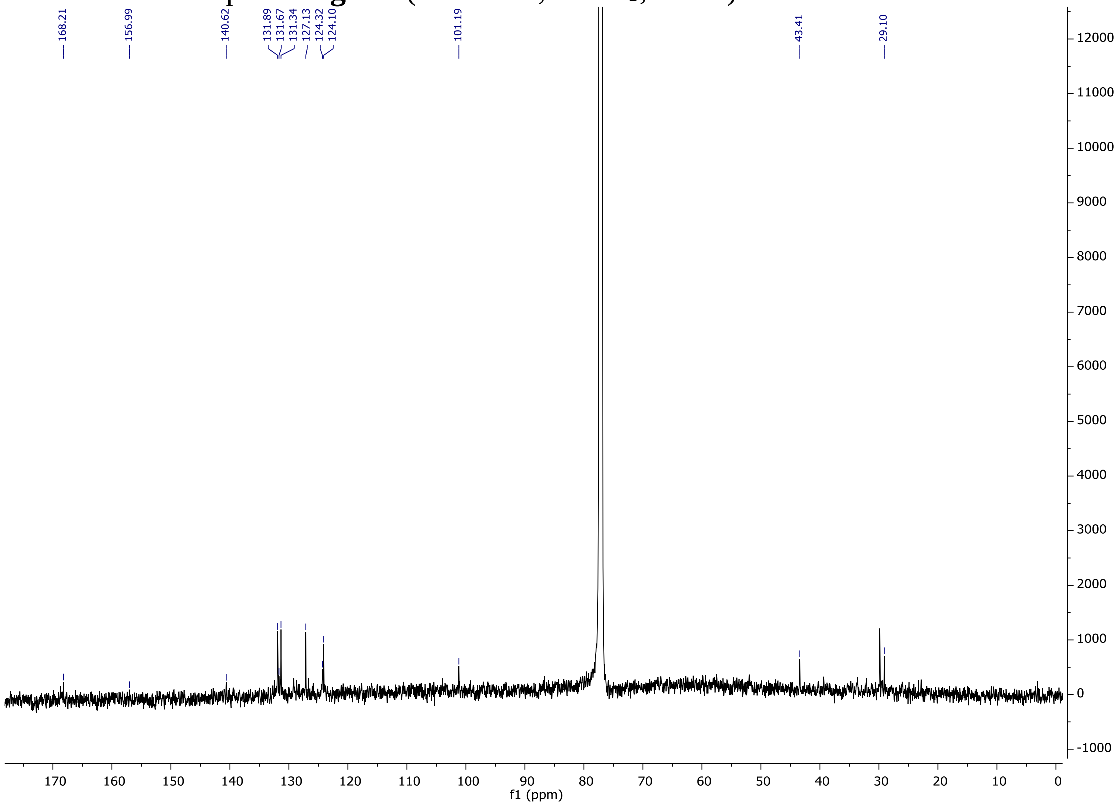

HSQC of compound **5g-rot**

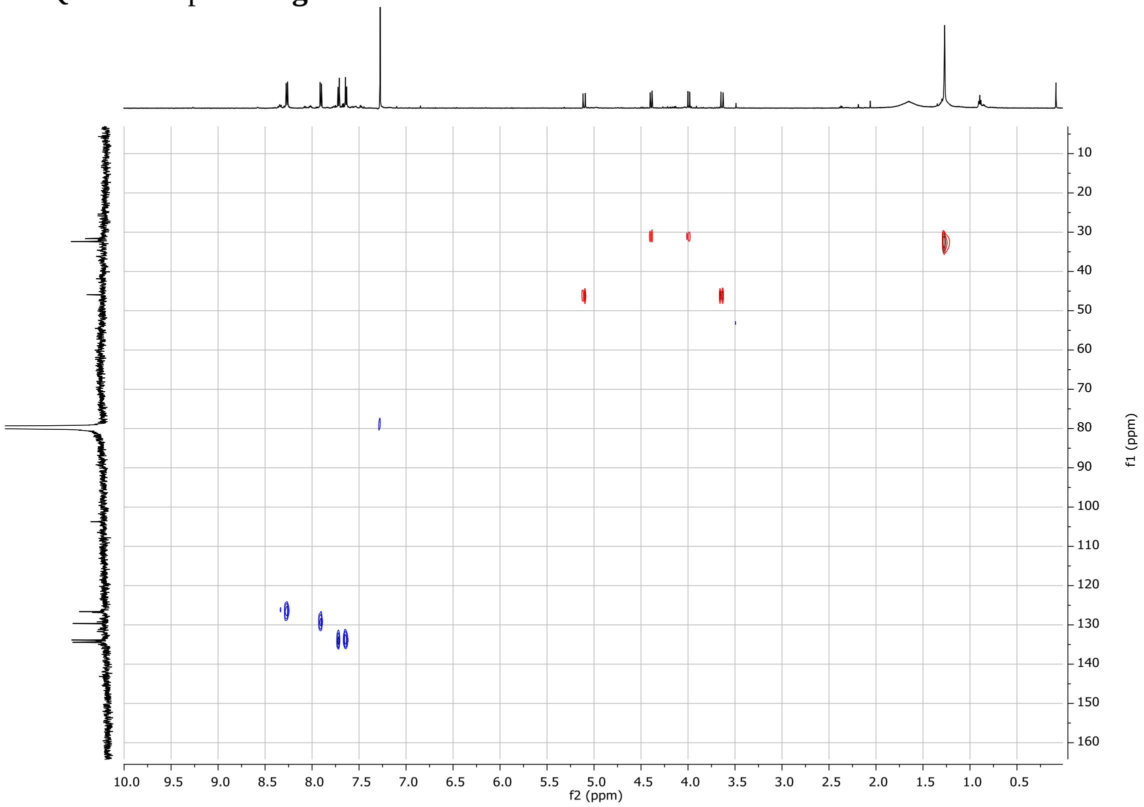

HMBC of compound **5g-rot**

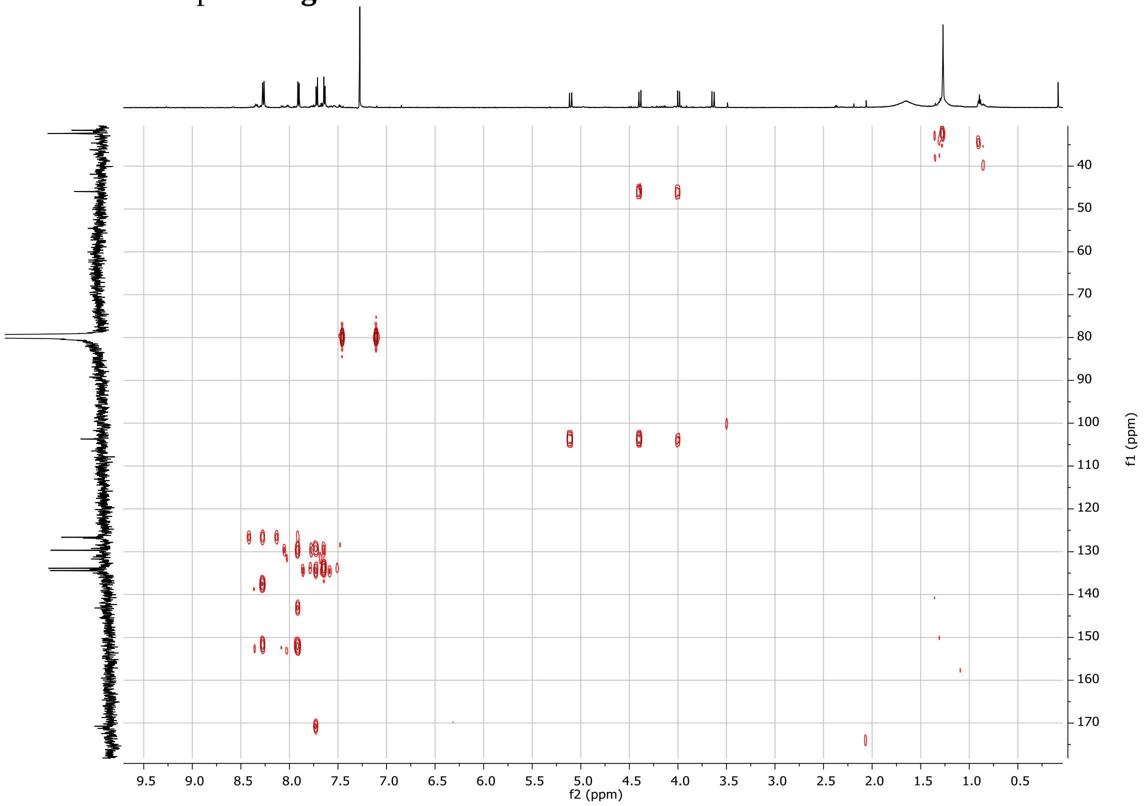

Supplement: Supplementary file 1 [file ol5c03069_si_001.pdf]
